# Applications of Machine Learning for Early Diagnosis and Prognosis of Chronic Kidney Disease: Current Evidence

**DOI:** 10.3390/diagnostics16152354

**Published:** 2026-07-27

**Authors:** Leon Van de Putte, Marijn M. Speeckaert

**Affiliations:** 1 Faculty of Medicine and Health Sciences, Ghent University, 9000 Ghent, Belgium; leon.vandeputte@ugent.be; 2 Department of Nephrology, Ghent University Hospital, 9000 Ghent, Belgium; 3 Research Foundation-Flanders (FWO), 1000 Brussels, Belgium

**Keywords:** artificial intelligence, machine learning, CKD, diagnosis, prognosis

## Abstract

In recent years, interest in machine learning applications has grown rapidly, particularly in the medical domain, where large amounts of data are available for training these models. This review focuses on the potential of machine learning for early diagnosis and prognosis of chronic kidney disease (CKD) by examining the most recent literature. Articles published from 2016 to 2025 were collected from online databases such as PubMed, Web of Science, and Embase. After abstract and full-text screening, 57 articles were included in the results section. Machine learning was applied to clinical and laboratory data, medical imaging, urine samples, retinal images, and at-home measurements to diagnose CKD and predict CKD progression and related complications. Although many studies reported high discriminatory performance, the evidence base was dominated by retrospective, single-center, and methodologically heterogeneous studies, with frequent high-risk-of-bias findings and limited external validation. Furthermore, most published models are not yet sufficiently validated for clinical deployment. Before these tools can be adopted in routine care, prospective, multicenter studies are required that report calibration and clinical utility, adhere to established reporting standards, and demonstrate added value over the current standard of care.

## 1. Introduction

Chronic kidney disease (CKD) is defined as abnormalities in kidney structure or function lasting three months or longer, regardless of cause. These diseases, with different causes, are grouped into this category because of common mechanisms that damage kidney function or structure. This is also notable in the therapy of CKD, which is frequently independent of the primary cause of kidney function decline [1]. The current threshold for CKD, based on glomerular filtration rate (GFR), is <60 mL/min/1.73 m^2^ [2]. The 2024 guidelines from Kidney Disease: Improving Global Outcomes (KDIGO) classify CKD by cause, GFR, and albuminuria, specifically the urine albumin-to-creatinine ratio (ACR). Other markers of CKD include urine sediment abnormalities, persistent hematuria, electrolyte and other abnormalities due to tubular disorders, histologic or imaging abnormalities, and a history of kidney transplantation [3].

According to an analysis of the Global Burden of Disease Study in 2017 [4], the estimated prevalence of all-stage CKD approached 700 million, exceeding the prevalence of diabetes, osteoarthritis, COPD, asthma, or depressive disorders. In 2017, CKD was the twelfth most frequent cause of death worldwide, with 1.2 million deaths. That number is projected to rise to 2.2–4.0 million by 2024. Dong et al. [5] projected that the global incidence rate per 100,000 population will increase from 234.72 in 2020 to 246.36 in 2030, while the absolute number of deaths will rise from 1,483,812 to 1,924,240 over the same period. In high- to middle-socio-demographic index (SDI) regions, aging was reported as the primary driver of the rise in CKD incidence, whereas in low- to middle-low SDI regions, population growth was the primary driver [5]. The 2024 KDIGO guidelines also highlighted the disparity between the burden of CKD and the availability of adequate healthcare infrastructure in low- and middle-income countries, as well as insufficient access to kidney replacement therapy (KRT) [3].

CKD is caused by two essential mechanisms [6]. The first is an initial trigger that causes kidney damage, such as an injury or an inflammatory event. The second is a compensatory mechanism involving hypertrophy and hyperfiltration in nephrons unaffected by the initial trigger. Hormones, cytokines, and growth factors drive this second mechanism, which ultimately results in sclerosis and a decline in kidney function [6]. Both hypertension and diabetes are considered the most frequent risk factors for CKD [2]. Hypertension is both a cause and an effect of CKD: systemic hypertension increases glomerular capillary pressure, while the afferent arteriole loses its ability to self-regulate, leading to vascular distortion and continued hyperfiltration [6,7]. In diabetic nephropathy, hyperglycemia increases growth factors and advanced glycation end products, which induce hyperfiltration, followed by morphological changes in the glomerulus and ultimately fibrosis, sclerosis, and increased cardiovascular risk [6,8].

Early-stage CKD is often asymptomatic, making it difficult for both patients and clinicians to recognize or detect [1,2]. As CKD progresses, symptoms such as fatigue, nausea, vomiting, anorexia, insomnia, and edema become more frequent [2]. A meta-analysis by Fletcher et al. [9] found that the prevalence of bone and joint pain, fatigue, memory difficulties, muscle cramps, itching, restless legs, and numerous other symptoms was significantly higher in patients with CKD than in the healthy population. The rise in uremic toxins due to declining kidney function is believed to account for several of these symptoms, and additional mechanisms include anemia, vitamin D deficiency, and hyperphosphatemia [10].

Because CKD can be detected with relatively inexpensive tools, universal screening has been repeatedly questioned. However, economic analyses have shown that screening the general population is not cost-effective, and no randomized controlled trial (RCT) has demonstrated improved outcomes from screening asymptomatic people [1,2]. Current guidelines recommend screening people at high risk, such as those with hypertension, diabetes mellitus, cardiovascular disease, or those older than 60 years. Key diagnostic instruments include GFR estimation, measurement of albuminuria, microscopic examination of urine sediment, and imaging. Ultrasound and color Doppler imaging (US-CDI) are the first-choice imaging techniques when CKD is suspected, while percutaneous biopsy remains available to confirm the diagnosis or etiology [11,12].

There is currently no treatment that can cure CKD or reverse existing kidney damage. At present, treatment options include conservative pharmacological and non-pharmacological interventions, renal replacement therapy (RRT), or kidney transplantation, depending on the severity of kidney function loss [13]. Non-pharmacological measures include limiting sodium and protein intake, engaging in at least 150 min of physical activity per week, smoking cessation, and weight management [2,3,13]. Multiple pharmacotherapies can slow the progression of kidney disease, with key targets including the renin–angiotensin–aldosterone system (RAAS), which is targeted by ACE inhibitors (ACEIs) and angiotensin receptor blockers (ARBs). More recently, sodium-glucose cotransporter-2 (SGLT-2) inhibitors have emerged as the most prominent first-line agents for reducing CKD progression and cardiovascular risk, independent of their glycemic effect. The 2024 KDIGO guidelines recommend initiating an SGLT-2 inhibitor in CKD patients with an eGFR ≥ 20 mL/min/1.73 m^2^ and a urine ACR ≥ 200 mg/g, or in patients with heart failure [3,13].

Artificial intelligence (AI) can be defined as a branch of computer science that seeks to understand and build intelligent entities, often implemented as software programs, or, more broadly, as a field focused on automating intellectual tasks typically performed by human beings [14,15]. Machine Learning (ML) is a subset of AI that uses algorithms to identify patterns in data. In ML, programs are trained on data and learn by finding patterns in large datasets, in contrast to classical programming, where a computer is given a predefined algorithm [14,15,16]. Deep Learning (DL) is a further subset of ML that employs artificial neural networks with multiple layers to identify patterns in data [15].

Depending on the task, different evaluation metrics are used to assess an ML model’s performance. Common issues include underfitting (high bias, poor performance on training data) and overfitting (high variance, poor performance on new test data), which necessitate finding the right bias-variance trade-off [16,17]. In binary classification, the receiver operating characteristic (ROC) curve and its area under the curve (AUROC) are commonly used performance metrics, whereas for unbalanced datasets, the area under the precision-recall curve (AUPR) is preferred. For datasets too small for a separate validation set, k-fold cross-validation provides a robust alternative [17].

**Figure 1:**
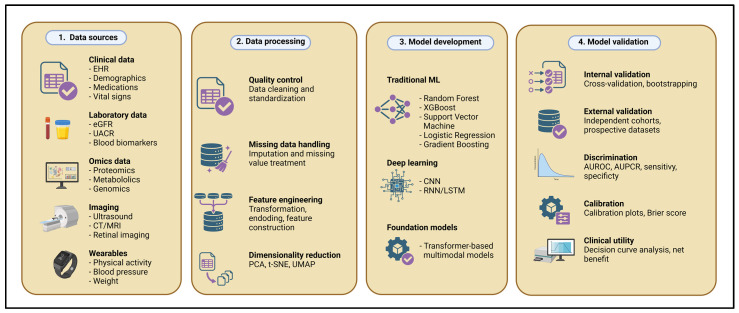
Methodological workflow for machine learning applications in chronic kidney disease. Clinical, laboratory, omics, imaging, and wearable data undergo preprocessing before model development using classical machine learning, deep learning, or foundation models. Model performance is then evaluated using internal or external validation, discrimination, calibration, and clinical utility assessment. Abbreviations: EHR, electronic health record; eGFR, estimated glomerular filtration rate; UACR, urine albumin-to-creatinine ratio; CT, computed tomography; MRI, magnetic resonance imaging; PCA, principal component analysis; t-SNE, t-distributed stochastic neighbor embedding; UMAP, uniform manifold approximation and projection; ML, machine learning; XGBoost, Extreme Gradient Boosting; CNN, convolutional neural network; RNN, recurrent neural network; LSTM, long short-term memory; AUROC, area under the receiver operating characteristic curve; AUPCR, area under the precision-recall curve.

Despite advances in CKD management, early diagnosis and accurate prediction of disease progression remain significant clinical challenges [1,2,3]. One reason ML continues to attract increasing attention is that it can uncover hidden patterns in large datasets, which can help us group patients by risk level or even by the type of treatment to be given [17,18]. At present, only a limited number of studies are available, and all of them have notable methodological limitations. For example, they have mainly relied on data from a single center in the past, used different outcome definitions, rarely tested the model’s performance outside the training data, and mostly relied on data from very few patients. So, it is impossible to know whether most such models will work well in real clinical practice or yield incorrect results. That can only be assessed by conducting multicenter, prospective validation studies. The aim of this article is to review the literature on the use of AI for diagnosing and predicting the course of kidney disease from multiple angles, focusing on methods, validation, and potential for clinical use. The central research question is: What are the current applications of ML in the diagnosis and prognosis of CKD? Figure 1 summarizes the methodological workflow for machine learning applications in CKD, from multimodal data acquisition and preprocessing to model development and validation, providing the methodological framework for the studies discussed throughout this review.

## 2. Materials and Methods

For this review, multiple electronic databases were used to identify relevant publications: PubMed, Embase, and Web of Science. A structured literature search was performed using predefined search strings combining terms related to “chronic kidney disease”, “machine learning”, “artificial intelligence”, “diagnosis”, and “prognosis”. Boolean operators (AND/OR) were used to refine the search. The literature search was completed on 8 April 2025. To allow the literature search to be fully reproduced, the complete search strategy for each database (PubMed, Embase, and Web of Science) is reported verbatim in Supplementary Table S1, including the full query with exact syntax, all field tags and controlled vocabulary (MeSH and Emtree), the Boolean operators used, every limit and filter applied (the 1 January 2016 to 8 April 2025 publication-date range and any language or article-type restrictions), and the number of records retrieved. Free-text searches were limited to the period from 1 January 2016, to 8 April 2025, to avoid missing articles not yet indexed by MeSH or Emtree. No language restrictions were applied. This yielded 1738 articles. After removing duplicates using EndNote, 1267 articles remained. Titles and abstracts were screened against predefined eligibility criteria. Potentially eligible studies were then assessed in full text. Eligibility was determined using the predefined inclusion and exclusion criteria described below. Information from each included study was extracted using a standardized extraction form that documented study design, study population, data source, machine learning methodology, clinical application, outcome definition, validation strategy, performance measures, and principal findings relevant to this review’s objectives. After title and abstract screening, excluding reviews, case reports, editorials, and articles not relevant to the research question, 210 articles remained for full-text screening. From this group, 57 articles were selected based on relevance to the research question. Inclusion criteria were: (1) studies applying ML or deep learning techniques; (2) studies focusing on chronic kidney disease diagnosis, staging, or prognosis; and (3) studies conducted in human populations. Exclusion criteria were: (1) studies limited to animal models; (2) studies focusing exclusively on end-stage renal disease or dialysis without predictive modeling; (3) studies not involving a diagnostic or prognostic ML application; and (4) non-original articles such as reviews, editorials, or case reports. Articles were also excluded if CKD was not the primary clinical focus or if machine learning was not central to the diagnostic or prognostic objective.

This review was conducted as a narrative review and, therefore, was not prospectively registered. Nevertheless, predefined eligibility criteria, a structured literature search, and transparent study selection procedures were established before screening began. To improve reporting transparency, relevant elements of the PRISMA 2020 reporting guideline, including the study selection flow diagram, were incorporated where appropriate. The PRISMA 2020 flow diagram is provided in Supplementary Figure S1.

Methodological quality was evaluated using established assessment frameworks. The reporting quality of prediction model studies was assessed according to the TRIPOD-AI Statement [19]. The risk of bias and applicability were evaluated using PROBAST(+AI). For each included study, the assessment considered the four methodological domains of participants, predictors, outcome, and analysis, after which an overall judgment of risk of bias and applicability was assigned in accordance with the published guidance [20]. Detailed PROBAST(+AI) assessment forms for all included studies are provided in Supplementary Table S1.

Due to substantial heterogeneity across study populations, predictor variables, machine learning methods, outcome definitions, validation strategies, and reported performance measures, a quantitative meta-analysis was deemed inappropriate, and the findings were synthesized narratively.

## 3. Results

A total of 57 studies met the eligibility criteria and were included in this review. Of these, 47 focused primarily on diagnostic applications and 10 on prognostic applications. Diagnostic studies were grouped by primary data modality, including clinical and laboratory data, radiological imaging, urine analysis, histopathology, at-home monitoring, and retinal imaging. Prognostic studies were organized by the clinical outcome being predicted, namely CKD progression, CKD-related complications, or mortality. Due to substantial heterogeneity in study populations, outcome definitions, machine learning algorithms, validation strategies, and reported performance measures, the results are synthesized narratively by clinical application rather than by direct comparison of performance metrics.

### 3.1. Diagnosis

#### 3.1.1. Blood Analysis and Clinical Examination

Blood-based and routinely collected clinical variables formed the largest category of diagnostic studies. These investigations addressed four principal tasks: CKD detection, CKD staging, prediction of incident CKD, and differentiation of CKD etiology. Although reported discrimination was generally high, considerable heterogeneity remained across cohort sizes, predictor selection, validation strategies, and outcome definitions.

Several studies evaluated ML models for detecting CKD using demographic, clinical, and laboratory features. Chen et al. [19] tested three models, k-nearest neighbor (KNN), support vector machine (SVM), and Soft independent modeling by class analogy (SIMCA), in 386 patients, achieving up to 99.0% accuracy with the SVM. This figure was obtained from a small, single cohort using internal validation only, without an independent external test set, and should therefore not be read as generalizable discrimination. Subsequent studies using the publicly available UCI dataset of 400 patients confirmed strong performance. Polat et al. [21] reported an AUROC of up to 0.988 using an SVM with feature selection (again on the 400-patient UCI benchmark, with feature selection performed on the same data and no external validation), and Singh et al. [22] reported 100% accuracy using a neural network on the UCI dataset. Although noteworthy, this result should be interpreted cautiously because it was obtained from a relatively small, publicly available dataset that has been extensively reused in machine learning research. Consequently, the reported performance may not be representative of independent clinical populations. Studies reporting exceptionally high or near-perfect performance should be evaluated in light of several methodological considerations, including dataset size, class balance, potential data leakage, reuse of benchmark datasets, validation strategy, and the absence of independent external validation. These factors may increase the risk that reported performance is optimistic rather than generalizable to routine clinical practice. The methodological criteria used to interpret studies reporting exceptionally high predictive performance are summarized in Supplementary Table S2. Vásquez-Morales et al. [23] demonstrated, on a much larger dataset of 40,000 Colombian patients, that NN outperformed SVM and random forests (RFs), achieving 95% accuracy and an AUROC of 0.98. Beyond binary CKD detection, Ilyas et al. [24] classified patients into individual CKD stages using decision tree (DT) and RF models, while Koch-Nogueira et al. [25] applied XGBoost and DT models to detect CKD in children, achieving AUROCs of up to 0.945.

Several studies moved beyond detection to risk prediction in longitudinal cohorts. Lapi et al. [26] followed nearly 1 million patients over 3 years and compared 7 ML models, finding that LightGBM achieved the highest AUROC of 88.9%. Yoshizaki et al. [27] followed over 30,000 patients for 6 years to predict the risk of reaching an eGFR < 60 mL/min/1.73 m^2^ or developing proteinuria, with the best-performing models achieving AUROCs up to 0.941. Further studies distinguished among CKD etiologies: Zhang et al. [28] differentiated diabetic nephropathy from non-diabetic nephropathy with an AUROC of 0.920, while Glazyrin et al. [29] combined spectrometry with ML to separate CKD etiologies with an accuracy of 87.5%. Notably, two studies comparing ML models to established GFR equations found mixed results: Zhao et al. [30] showed that an ensemble model had significantly lower P30 accuracy than the CKD-EPI formula (58.9% vs. 74.1%), while Lanot et al. [31] similarly reported that RF models fell short, with P10 accuracy below 60%.

Overall, studies using clinical and laboratory variables have consistently demonstrated strong diagnostic performance, relying primarily on routinely available data. Nevertheless, studies directly comparing ML with established clinical equations have shown that ML does not consistently outperform conventional approaches, underscoring that increasing algorithmic complexity alone does not guarantee superior clinical performance.

#### 3.1.2. Radiology

Radiological studies primarily evaluated ultrasound, followed by MRI and CT. Compared with blood-based models, imaging studies more often focused on tissue characterization, fibrosis assessment, disease staging, and image segmentation, reflecting the structural information available from medical imaging. Although several imaging studies reported AUROC values above 0.90, many were based on relatively small retrospective cohorts with limited external validation, and the reported performance should therefore be interpreted with caution. Ultrasonography (US) is the first-line imaging modality for evaluating CKD, and multiple studies have explored its integration with ML. Chen et al. [32] demonstrated that an SVM model could classify CKD stages from US images with accuracies up to 75.95% per kidney side, while Tian et al. [33] showed that a convolutional neural network (CNN) trained on 4365 US images outperformed both junior and senior physicians in CKD detection (AUROC 0.918 for the model vs. 0.869 for senior physicians, *p* < 0.001). Importantly, when classifying US images into the correct CKD stage, the model also outperformed senior physicians in early stages: the model’s AUROC was 0.781 in stage 1, 0.880 in stage 2, and 0.905 in stage 3, compared with 0.506, 0.586, and 0.796, respectively, for the senior group [33]. Weaver et al. [34] specifically examined the prediction of CKD progression in children with posterior urethral valves using a hybrid model that combined US image-derived features and clinical data, achieving an AUROC of 0.85 over 5 years in this pediatric population.

Several studies have investigated ML models to predict renal fibrosis using US data, potentially reducing the need for invasive kidney biopsies. Qin et al. [35] reported an AUROC of 0.86 for a multimodal US deep learning model, which was not statistically different from a clinical model based on eGFR and 24 h-proteinuria (AUROC 0.80). Notably, a SHAP analysis in this study showed that superb microvascular imaging accounted for 65.39% of the model’s output impact, compared with 27.51% for strain elastography and only 7.10% for classical gray-scale ultrasound. Chang et al. [36] combined US images with patient biomarkers and reported an AUROC of 0.96 using a logistic regression model, outperforming models trained on either data source alone. In additional studies, combined models incorporating shear-wave elastography achieved AUROCs of 0.77–0.86 [37,38,39].

MRI-based studies demonstrated additional applications. Yoruk et al. [40] used an RF algorithm to segment contrast-enhanced MRI images of children’s kidneys and then calculated eGFR from the segmentation output, achieving an F1-score of 0.93 for whole-kidney segmentation and a correlation of ρ = 0.99 with manual methods. Mo et al. [41] combined four segmentation methods with an SVM classifier to classify kidneys into normal function, mild-to-moderate impairment, and severe impairment on T2-weighted MRI, with the best-performing method achieving an AUROC of 0.938 for severe impairment. Bin Islam et al. [42] compared nine DL models for classifying healthy kidneys from CKD kidneys based on HASTE MRI sequences, reporting accuracies up to 94.38% and F1-scores up to 0.9438. In CT imaging, Fu et al. [43] presented a deep learning model for segmenting renal cysts, achieving a Dice Similarity Coefficient of 96.25%. Lee et al. [44] applied XGBoost to high-resolution peripheral quantitative CT images at four skeletal locations, including the distal and diaphyseal radius and tibia, achieving AUROCs of 0.98–0.99 for classifying patients with CKD stages 3–5, highlighting an unexpected yet promising application of bone imaging in CKD detection.

Collectively, these findings suggest that the greatest contribution of ML in renal imaging may lie in quantitative image interpretation and tissue characterization rather than simply replacing radiologist interpretation.

#### 3.1.3. Urine Analysis

Urine-based ML applications combined conventional urinalysis with advanced analytical techniques, including proteomics, mass spectrometry, and Raman spectroscopy. These studies illustrate the broad spectrum of urinary biomarkers that may support CKD detection, staging, and etiological classification. Reported discrimination was generally high; however, heterogeneous analytical platforms, limited external validation, and small study populations reduce confidence in the generalizability of these findings. Urine-based ML applications ranged from conventional dipstick data to advanced analytical techniques, including spectrometry, spectroscopy, and biosensors. Jang et al. [45] developed XGBoost models using five urine dipstick features, along with age and sex, for two eGFR thresholds (60 and 45 mL/min/1.73 m^2^), achieving AUROCs of 0.91–0.95 across two independent external test sets from a health checkup center and the general population. However, performance dropped notably in patients aged 65 and older, with both models failing to exceed an AUROC of 0.80 in that subgroup. Mavrogeorgis et al. [46] used an SVM to differentiate among CKD etiologies using capillary electrophoresis and mass spectrometry data, achieving at least 95.56% accuracy in binary classification between diabetic nephropathy and healthy controls without dimensionality reduction, and 87.15% in multiclass classification across four groups (healthy, diabetic nephropathy, vasculitis, and IgA nephropathy) in the external test set.

Wang et al. [47] classified urine Surface-Enhanced Raman Spectroscopy (SERS) data into the correct CKD stage using several ML models, with RF achieving the highest accuracy of 97.58%. However, its F1-score of 0.8535 was lower than that of the DT model (0.9852), illustrating that no single metric is sufficient for complete model evaluation. Iftikhar et al. [48] compared nine models on laboratory urine features from Pakistani patients, with SVM variants consistently outperforming the others across three training-to-testing ratios, achieving up to 91.71% accuracy. Taken together, these findings suggest that urine data, analyzed using a wide range of analytical and ML techniques, may support both CKD detection and differentiation of etiology.

#### 3.1.4. Histopathology

Unlike previous diagnostic modalities, histopathological studies primarily focused on extracting prognostic and functional information from digitized kidney biopsy specimens rather than establishing the initial diagnosis. ML models appear capable of extracting diagnostic and prognostic value from kidney biopsy images. Most studies were conducted in selected biopsy cohorts with limited sample sizes, restricting the generalizability of the reported performance. Kolachalama et al. [49] developed six CNN models trained on trichrome-stained kidney biopsy images for tasks including eGFR stage prediction, serum creatinine classification, proteinuria classification, and 1-, 3-, and 5-year survival prediction. The CNNs outperformed a pathologist-estimated fibrosis score (PEFS)-based classifier across all tasks, with AUROCs of 0.912 for creatinine classification, 0.867 for proteinuria at diagnosis, and 0.904 for 5-year survival prediction. For eGFR stage classification, the CNN achieved an accuracy of 0.649 and a Cohen’s κ of 0.519, compared with 0.345 accuracy and a κ of 0.051 for the PEFS-based model, highlighting the potential for considerably greater discriminating ability of image-based DL compared with conventional pathologist scoring.

Mendapara [50] used ML to identify differentially expressed genes between CKD and healthy renal tissue in the Gene Expression Omnibus (GEO) database. Using differential expression analysis and Lasso regression, 35 differentially expressed genes were identified, and six key genes, DUSP1, GADD45B, IFI30, IFI44L, ATF3, and LYZ, were selected to train a final RF classifier. The model achieved an AUROC of 0.913 and an accuracy of 89.83% in an external validation set, demonstrating that ML can identify novel disease-relevant genes and incorporate them into a predictive model.

Another group of researchers applied unsupervised learning methods to histopathological biopsy images from the C-PROBE cohort, first using a bag-of-words model [51] and then extending it with spatial graph analysis via a deep graph convolutional neural network [52]. The bag-of-words model, combining imaging and clinical features, achieved AUROCs of 0.91 for baseline eGFR prediction and 0.80 for one-year eGFR decline. Incorporating spatial relationships in the subsequent study improved these figures to 0.9563 and 0.8549, respectively, demonstrating how increasingly sophisticated ML architectures can extract additional predictive signal from the same underlying data.

These studies demonstrate that deep learning can identify complex histopathological patterns that go beyond conventional semiquantitative pathological scoring systems.

#### 3.1.5. At-Home Diagnosis

Metherall et al. [53] examined the use of ML for CKD diagnosis based solely on at-home measurements. The dataset was divided into three feature groups—at-home, monitoring, and laboratory features—and RF and ANN models were trained separately on each group. The RF model achieved 92.5% accuracy and an AUROC of 0.965 using only at-home-measurable features, with hypertension and diabetes showing the highest mean entropy importance in this group. With monitoring or laboratory features, both models reached accuracies exceeding 99%. Two further studies examined the use of ML to interpret smartphone photographs of urinary dipsticks [54,55]. Bhatt et al. [54] reported that a KNN model achieved up to 96% accuracy on the test set for predicting albumin concentration categories from dipstick images, with performance declining when the lighting source or smartphone brand was varied, suggesting the need for further robustness testing. Xu et al. [55] demonstrated AUROCs of 0.941–1.0 for different dipstick features using an RF model under optimal smartphone conditions in an independent test set of 187 dipsticks. These studies collectively provide a potentially valuable tool for early CKD screening in settings with limited access to clinical facilities, a particularly relevant perspective given the global burden of CKD in low- and middle-income countries.

These findings suggest that ML-assisted home monitoring may complement, rather than replace, conventional clinical assessment, especially in underserved settings with limited access to laboratory testing.

#### 3.1.6. Retinal Imaging

Retinal imaging is among the most innovative non-invasive diagnostic approaches identified in this review. The biological rationale is supported by the shared microvascular and developmental characteristics of the retina and kidney. Sabanayagam et al. [56] were the first to propose using a DL algorithm to detect CKD from retinal photographs, drawing on the known shared developmental, physiological, and pathological pathways between the eye and the kidney. They developed three models (image-only, clinical risk factors-only, and a hybrid model) and tested them on two independent external datasets from China and Singapore, achieving AUROCs greater than 0.80 on both datasets. Zhang et al. [57] extended this concept by developing models not only for CKD detection but also for predicting the future risk of developing CKD in asymptomatic patients from retinal images, achieving AUROCs of 0.885 and 0.870 for CKD detection in two test sets, and a C-index of 0.719 for the combined fundus and clinical data model in CKD risk prediction. Dong et al. [58] used DL on retinal images for the more specific task of classifying CKD patients as having diabetic nephropathy versus non-diabetic nephropathy, achieving AUROCs of 0.989 on a prospective dataset and 0.932 on a multicenter dataset. In this study, a clinically significant increase of more than 20% in average diagnostic accuracy, from 70.4% to 90.9%, was observed when the ML model’s visualization maps were used to assist clinicians. Bhak et al. [59] combined retinal image DL with urine dipstick data in a multimodal model that achieved an AUROC of 0.94 in the internal test set and 0.88 in the external test set. An et al. [60] further explored whether using a two-factor eGFR formula (creatinine and cystatin C combined) rather than the single creatinine-based formula as the reference standard would change the performance of these DL models, finding a statistically significant advantage only when eGFR-based labels were used, but no significant difference when ICD-10 codes for CKD were used as the reference.

Compared with several other imaging modalities, retinal imaging studies more often included geographically independent external validation cohorts, thereby increasing confidence in the reported diagnostic performance. Nevertheless, prospective implementation studies remain scarce.

### 3.2. Prognosis

Prognostic studies primarily evaluated longitudinal outcomes and were categorized into two major types: predicting CKD progression and predicting CKD-related complications or mortality. In contrast to diagnostic studies, prognostic investigations generally relied on longitudinal electronic health records and repeated laboratory measurements rather than cross-sectional datasets. Although discrimination was frequently favorable, substantial heterogeneity in outcome definitions and relatively limited prospective external validation complicate direct comparison between models and limit conclusions regarding clinical implementation.

#### 3.2.1. CKD Progression

The prognosis section primarily addresses the risk of CKD progression. Multiple studies have used proteinuria as a surrogate for CKD progression. Xiao et al. [61] reported that XGBoost achieved the highest accuracy (83%), while logistic regression achieved the highest AUROC (0.873) among nine models. Lu et al. [62] reported that an ensemble model combining XGBoost, NN, and logistic regression achieved the highest AUROC of 0.856 across eight compared models. Reddy et al. [63] explored prediction of kidney failure based on eGFR slope and clinical features, training models on an Australian dataset and externally validating on a Japanese dataset, highlighting the importance of generalizability: the RF model achieved an AUROC of 0.928 in external validation. Shih et al. [64] predicted CKD progression to stage 3 in over 50,000 Taiwanese patients, with an auto-ML scheme achieving the highest AUROC of 0.94, and identified the combination of age and creatinine as the most important feature pair.

For predicting kidney failure within a specified time period, multiple studies reported high model performance. Liang et al. [65] showed that an NN achieved the highest AUROC of 0.8991 for three-year kidney failure prediction among eight models. Bellocchio et al. [66] showed that their Naïve Bayes classifier for six-month kidney failure prediction outperformed the established Kidney Failure Risk Equation (KFRE) by 0.149 (*p* = 0.0013) and also outperformed expert clinicians for 24-month prediction (AUROC 0.96 vs. average expert AUROC of 0.79). Segal et al. [67] demonstrated that XGBoost achieved an AUC of 0.93 for predicting kidney failure within 6 months in a large commercial insurance database of nearly 27,000 patients. Tangri et al. [68] externally validated the ML Klinrisk RF model on data from two major canagliflozin trials, reporting AUROCs of 0.81 to 0.88 across one- to three-year prediction horizons. Further studies examined CKD progression in specific patient groups, such as patients with type 2 diabetes [69], where XGBoost reached an AUROC of 0.953, and patients with IgA nephropathy [70], where a recurrent neural network (RNN) combining baseline and time-dependent data achieved a c-statistic of 0.93.

Overall, predicting CKD progression consistently yielded AUROC values above 0.85 across several independent cohorts. However, variation in endpoint definitions and limited external validation reduce direct comparability and may affect generalizability.

#### 3.2.2. Complications and Mortality

Several studies examined the prediction of specific complications in patients with CKD. Chang et al. [71] demonstrated that XGBoost predicted hyperkalemia (K+ > 5.5 mEq/L) with an AUROC of 0.876 and an accuracy of 93.3%, outperforming two nephrologists who achieved AUROCs of 0.745 and 0.741. Lu et al. [72] predicted sarcopenia in patients with CKD stage 3+ using five ML models, with the Gradient Boosting Machine (GBM) achieving the highest AUROC of 0.923. Hsu et al. [73] predicted osteoporosis in more than 6000 CKD patients, with the ANN model achieving the highest AUROC of 0.930. In the postoperative setting, Oh et al. [74] predicted CKD after renal cell carcinoma surgery in nearly 4400 patients, with Gradient Boosting reaching an AUROC of 0.826. For cardiovascular disease, Zhu et al. [75] trained seven models on data from 8944 CKD patients, with XGBoost achieving the highest AUROC of 0.893. Tran et al. [76] predicted all-cause mortality in non-dialysis stage 4–5 CKD patients, finding that an optimized Naïve Bayes model reached an AUROC of 0.81. Collectively, prognostic ML applications extended beyond predicting kidney failure to include metabolic complications, cardiovascular disease, postoperative renal dysfunction, osteoporosis, sarcopenia, and mortality. Although discrimination was generally favorable, relatively few studies reported calibration, clinical utility analyses, or prospective validation, limiting conclusions about readiness for routine clinical implementation (Table 1(a,b)).

### 3.3. Summary of Risk-of-Bias Assessment

The methodological quality of the included prediction-model studies was evaluated using the PROBAST(+AI) framework [20]. Overall, the assessment showed that methodological limitations were common across the current literature. The Analysis domain had the highest frequency of high risk of bias, primarily due to inadequate handling of missing data, insufficient assessment of model overfitting, limited reporting of calibration, and the absence of external validation. The Participants and Outcome domains also raised frequent concerns, owing to the predominant use of retrospective single-center cohorts and publicly available datasets with limited information on patient selection and outcome ascertainment. By contrast, the Predictors domain generally had fewer concerns because most studies used routinely available clinical, laboratory, or imaging variables that were consistently defined and clinically relevant (Table 2).

**Figure 2:**
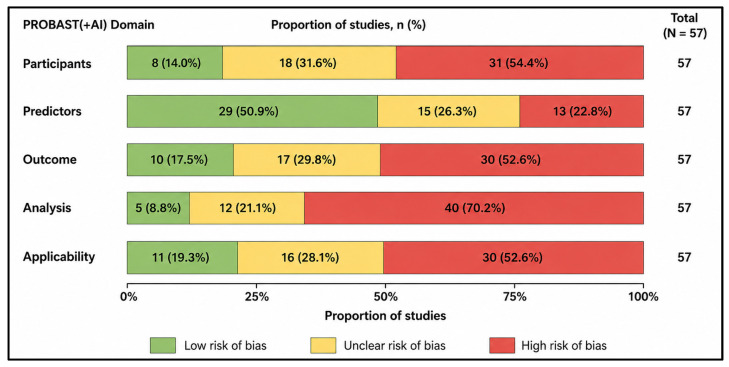
Domain-level summary of the PROBAST(+AI) risk-of-bias assessment across the included studies (*n* = 57). Horizontal stacked bars show the proportion of studies judged to have low (green), unclear (yellow), or high (red) risk of bias for each PROBAST(+AI) domain (Participants, Predictors, Outcome, and Analysis) and for applicability. Detailed study-level PROBAST(+AI) assessments are provided in the Supplementary Materials.

Overall, only a minority of studies met most PROBAST(+AI) criteria across all domains, supporting a cautious interpretation of the frequently reported high predictive performance. These findings indicate that the current evidence base is more limited by methodological quality and generalizability than by algorithmic performance alone. Figure 2 summarizes the domain-level PROBAST(+AI) assessments across all included studies, while the complete study-specific assessments are provided in the Supplementary Materials.

## 4. Discussion

### 4.1. Diagnosis

The screening methodology of the 2024 KDIGO guidelines recommends using eGFR or ACR to detect CKD early [3]. Imaging techniques are not commonly used for baseline CKD diagnosis and are reserved for further evaluation and determination of etiology [3,11]. Studies in this review suggest the potential of combining ML with imaging techniques to diagnose CKD, with accuracies reaching 94.38%, though performance can be considerably worse in some cases [32,33,34,35,36,37,38,39,40,41,42,43,44]. In the study by Tian et al. [33], the ML model outperformed senior clinicians in classifying US images of patients with early-stage CKD. Furthermore, multiple studies have demonstrated that ML, combined with imaging, may predict the extent of kidney fibrosis without the need for invasive biopsy [11,35,36,37,38,39]. Given that invasive biopsy procedures can result in severe complications, including infection or death, the use of ML and imaging as an additional or replacement tool might be more convenient for both patients and physicians [11]. Even when a biopsy is still required, ML shows potential for a variety of subsequent analytical tasks, including evaluating fibrosis, predicting eGFR, and predicting survival [49,51,52].

This review also presented less conventional diagnostic methods for CKD, including retinal imaging and at-home measurements [53,54,55,56,57,58,59,60]. Because the eye and the kidney share developmental, physiological, and pathological pathways, and because patients with retinal microvascular pathology are more likely to have CKD, using retinal images as input for ML models has shown compelling results, with AUROCs consistently exceeding 0.80 in external validation sets [56]. Dong et al. [58] specifically demonstrated a clinically significant increase of more than 20% in average detection accuracy when the ML model aided nephrologists. At-home diagnostic tools, particularly the analysis of smartphone photographs of urinary dipsticks, represent another promising avenue for regions with limited access to hospital or physician care [53,54,55].

The studies examining blood results and clinical data form the largest group in the diagnosis section and cover the widest array of tasks, ranging from basic CKD detection to staging, risk prediction, etiology differentiation, and detection in specific subpopulations [19,20,21,22,23,24,25,26,27,28,29,77]. Accuracies ranged from 57.6% to 100% [22,25,26,27,28,29,30,31]. Nevertheless, it remains important to critically evaluate the added value of these ML applications compared with non-ML methods. Zhao et al. [30] showed that the Asian-modified CKD-EPI equation significantly outperformed an ML model for GFR estimation, and Lanot et al. [31] similarly found that RF models for eGFR estimation yielded relatively low P10 accuracies below 60%. These results demonstrate that applying ML does not automatically lead to better outcomes and may even perform worse than established non-ML methods.

### 4.2. Prognosis

Predicting CKD progression with ML achieved high accuracy and AUROC across model types and outcome definitions [61,62,63,64,65,66,67,68,69,70]. A particularly noteworthy approach was used by Reddy et al. [63], who trained models on an Australian dataset and tested them on a Japanese dataset, underscoring the critical importance of generalizability: a model trained on one population is not necessarily as successful when tested on another. Predicting kidney failure within a defined time frame could be a valuable clinical tool, especially if models can estimate its timing over a long horizon, enabling earlier patient communication and more advanced care planning [65,66,67,68]. Furthermore, predicting specific complications, from hyperkalemia and sarcopenia to cardiovascular disease and all-cause mortality, provides clinicians with additional tools to plan long-term care more accurately. If validated across larger, more diverse datasets, these models could identify which patients would benefit most from specific screening programs, thereby increasing the efficiency of these programs [71,72,73,74,75,76].

### 4.3. Model Performance

A first observation regarding model performance is the robustness of CNNs across different types of image data, consistent with the broader literature indicating that CNNs are well-suited for images because they capture spatial relationships [18]. MLP models also achieved strong results when trained on large, diverse datasets [23,73], consistent with their known strength in handling very large multivariate datasets [15]. More classical models also performed well. In Metherall et al. [53], despite being a simpler model, RF achieved higher accuracy and AUROC than the MLP, possibly due to the smaller dataset used. The consistent success of gradient boosting methods across many studies is also notable. In Xiao et al. [61], for example, XGBoost performed best among nine models, including MLP, across heterogeneous categorical and binary features, consistent with the known strength of gradient boosting as a non-linear classifier on non-linear data [18].

### 4.4. Strengths, Weaknesses, and Recommendations

When the included studies are evaluated using the TRIPOD-AI checklist [19], several reporting patterns emerge. Positively, many studies adequately described their datasets and inclusion criteria, and most clearly defined their prediction outcomes. A significant number of studies used small, retrospective, or publicly available datasets without clearly detailing participant flow or handling missing data. Sample-size justification and handling of missing data were rarely discussed, and fewer than 10% of studies performed external validation using independent data, a key TRIPOD-AI requirement for demonstrating model generalizability. Additionally, the use of different performance metrics across studies significantly limits the ability to compare results.

The structured PROBAST(+AI) assessment further demonstrated that the principal limitations of the current literature are methodological rather than algorithmic. Although many studies reported excellent discrimination, often exceeding an AUROC of 0.90, these results were frequently accompanied by a high risk of bias in the Analysis domain due to inadequate handling of missing data, limited external validation, incomplete calibration assessment, and potential overfitting. Consequently, improvements in study design, reporting quality, and prospective multicenter validation are likely to be at least as important as further advances in machine learning methodology.

Based on the above, several recommendations for future research can be made. First, standardizing outcome measures across studies, for example, by using a consistent definition of kidney failure, would greatly facilitate comparisons. Second, prospective impact studies or RCTs are needed to clarify the clinical impact and added value of ML relative to the standard of care. Third, external and temporal validation of ML models should become standard practice, and multicenter cohorts spanning different countries should be used to increase generalizability. Fourth, the use of multiple data types to build multimodal diagnostic and prognostic models is encouraged. Finally, more transparent reporting of data collection methods is essential, since the quality of the data used to train and test a model is the Achilles’ heel of any ML application [16].

**Figure 3:**
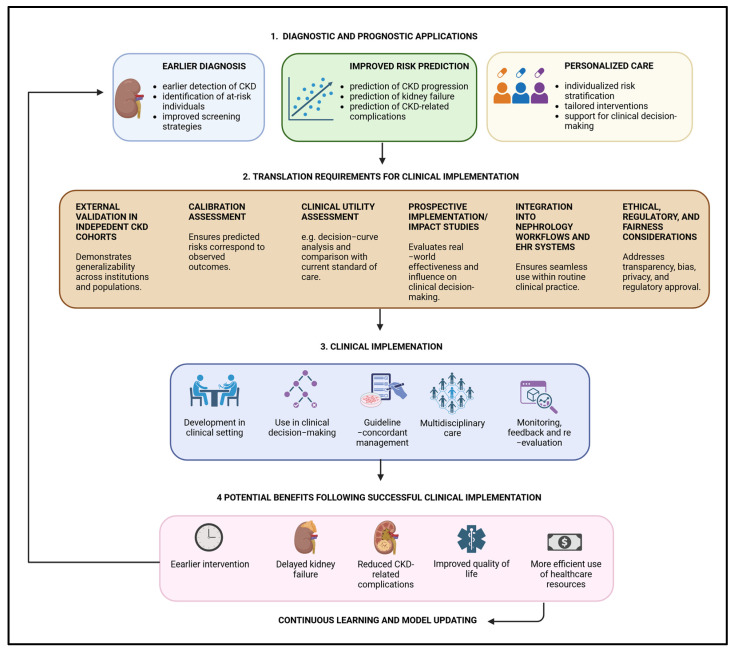
Possible clinical use and translational roadmap of ML in chronic kidney disease. ML may assist in early-stage diagnosis of CKD and can also increase the accuracy of prognosis for disease progression to dialysis or kidney transplantation, as well as for other comorbid diseases. It can also identify high-risk patients (risk stratification) and enable clinicians to make personalized decisions for them. Despite these benefits, for clinicians to use ML models routinely in their care, there must be external validation (i.e., good model performance in a new CKD dataset) across different CKD groups. Models that require calibration (i.e., a good match between predicted probabilities and actual outcomes) must also be demonstrated clinically, meaning that in clinical practice where ML models have been implemented, patient care outcomes have been measured. There should also be integration of AI models into existing healthcare routines and EHR systems. After deployment, the model should be monitored and updated at regular, continuous intervals to maintain its performance. Abbreviations: AI, artificial intelligence; CKD, chronic kidney disease; EHR, electronic health record; ML, machine learning.

Despite promising model performance, the clinical translation of ML in CKD remains limited, as few studies have demonstrated real-world impact, cost-effectiveness, or integration into routine clinical workflows, highlighting a critical gap between algorithm development and clinical implementation (Figure 3). A limited number of studies assessed whether ML-assisted predictions improve clinical decision-making using approaches such as decision-curve analysis or prospective impact evaluation. To close this gap, evaluations should pair discrimination with calibration, decision-curve analysis, and prospective, multicenter implementation studies that test whether ML adds incremental value beyond established risk models and translates into better patient outcomes.

Overall, the evidence presented in this review demonstrates that model performance on numerous machine learning challenges in CKD to date is encouraging, but clinical implementation is limited by methodological bias and a lack of external validation, which hampers the translation of evidence. Most investigations rely on retrospective samples, single-center data, and limited external validation across diverse populations and healthcare environments. Few studies focus on calibration and clinical utility metrics beyond the AUROC, further limiting interpretability. We will only be able to transition from algorithm development to clinical practice through prospective, multicenter studies that are transparently reported to TRIPOD-AI standards and focus on patient-centered metrics. Improving outcome measures with clinical relevance and integrating them with traditional clinical decision tools will ensure that ML strategies add value as a supplement or reinforcement of pre-existing chronic disease setups, rather than merely as a replacement.

### 4.5. PROBAST Analysis

Risk of bias and applicability were assessed for all 57 publications using the PROBAST(+AI) tool [20], with each model evaluated across the four domains of participants, predictors, outcome, and analysis. Overall, methodological quality was poor: 49 of 57 publications were judged to be at high risk of bias, only 7 at low risk, and 1 at unclear risk. The analysis domain was the most frequent source of concern, closely followed by the outcome domain. Recurring flaws included the absence of external validation, reliance on small or single-center cohorts, uncorrected class imbalance, feature selection performed outside the cross-validation fold, and inadequate handling of missing data, most often simple mean imputation or unexplained case deletion. A substantial subset of models, particularly those developed on the publicly available UCI dataset, also produced outcomes that were not defined according to accepted clinical (KDIGO) criteria [3]. Several showed outright circularity or data leakage, in which the outcome was derived deterministically from variables also used as predictors, inflating reported accuracy, occasionally to implausible values approaching 100%. Applicability concerns were similarly widespread, rated highly in 38 of 55 studies for development and 36 of 57 for evaluation, reflecting non-representative populations, single-scanner imaging pipelines, and outcomes that were poorly matched to the intended clinical use. Only six studies achieved a low rating across all applicable domains, generally by employing large, well-characterized cohorts with appropriate validation and clinically grounded endpoints. These findings indicate that, despite encouraging reported performance, the great majority of ML models for CKD are currently at high risk of bias and not yet ready for clinical deployment. The full PROBAST analysis for each article is available in the supplementary materials.

### 4.6. Studies with Possibly Overfitted Models

Several studies in this review reported discrimination or accuracy approaching perfect classification, including an accuracy of up to 100% [22], an AUROC of up to 0.988 [21], an AUROC approaching 1.00 in the at-home and wearable monitoring category [53], and AUROCs of 0.941–1.00 [53] for dipstick-derived features. In a clinical context as heterogeneous as CKD, such near-perfect or perfect discrimination is biologically implausible. It should be interpreted with caution, as it most often signals data leakage, severe overfitting, or evaluation on data that are not independent of the training set, rather than genuine real-world performance. To make this concern explicit, we summarize below, for each study reporting an AUROC of at least 0.99 or an accuracy of 100%, whether external validation was performed, the size and independence of the reported test set, and the resulting risk of overfitting.

Singh et al. [22] reported 100% accuracy for a neural network trained on the publicly available UCI CKD dataset, which included only 400 patients. No external validation was performed, and the reported metric was not derived from an independent, held-out cohort that is demographically and geographically distinct from the training data. A flawless result on such a small, widely reused benchmark should be interpreted cautiously because methodological characteristics, such as the use of small benchmark datasets and limited external validation, may have contributed to the exceptionally high reported performance.

Polat et al. [21] reported an AUROC of up to 0.988 for a support vector machine with feature selection, again using the same 400-patient UCI dataset. The performance was obtained without external validation on an independent cohort, and the small sample size, combined with feature selection performed on the same data, raises a substantial risk of optimistic bias and overfitting. These metrics should therefore be regarded as an upper bound under idealized, single-dataset conditions rather than as an estimate of clinical performance.

Metherall et al. [53] reported accuracies exceeding 99%, with summary-level AUROC approaching 1.00 when monitoring or laboratory features were included, in contrast to the more modest 92.5% accuracy and AUROC of 0.965 obtained from at-home-measurable features alone. No independent external validation cohort was reported. The near-perfect results obtained with laboratory features are particularly suspect for label leakage, because laboratory variables such as serum creatinine are components of the eGFR-based definition of CKD that serves as the outcome label; a model given such features is, in effect, partly answered. These figures are therefore unlikely to reflect the performance achievable from genuinely independent inputs in a screening population.

Xu et al. [55] reported AUROCs of 0.941–1.00 for individual dipstick-derived features using a random forest, evaluated on an independent test set of 187 dipsticks, but only under optimal smartphone conditions. Although the use of a separate test set is a methodological strength, the small size of that set, the restriction to favorable imaging conditions, and the absence of validation across multiple devices, lighting environments, and patient populations all limit confidence in the perfect-discrimination endpoint. A companion study reported performance degradation under varying imaging conditions [52], underscoring that an AUROC of 1.00 is not a credible real-world operating point.

Similarly, Lee et al. [44] reported AUROCs of 0.98–0.99 for classifying CKD stages 3–5 using high-resolution peripheral quantitative CT of the radius and tibia. This pilot-level evidence was derived from a limited cohort with minimal external validation, and the very high discrimination should be considered preliminary until replicated in larger, independent, multicenter samples.

Taken together, these examples illustrate that exceptionally high reported metrics are unlikely to generalize to real-world clinical populations unless supported by appropriate methodological safeguards. The key safeguards are as follows: external validation in cohorts independent of the development data, ideally drawn from different centers, regions, and case mixes; adequately sized and clearly described held-out test sets, with explicit separation among training, validation, and test partitions; the use of k-fold cross-validation when datasets are too small to support a validation set; and vigilance against data leakage, particularly when candidate features overlap with the variables used to define the outcome label. Reported performance that approaches perfect discrimination but lacks these safeguards should be interpreted as a marker of probable overfitting rather than as evidence of clinical readiness.

### 4.7. How Does ML Compare with Existing Clinical Tools?

AUROC and accuracy values are reported throughout this review, but a performance metric is only interpretable when benchmarked against what existing validated clinical tools or experienced clinicians already achieve in the same population. An ML model that reaches an AUROC of 0.85 is informative only if a validated risk score, such as the Kidney Failure Risk Equation (KFRE), or routine clinical judgment performs meaningfully worse on the same task. To clarify where ML adds genuine incremental value and where the evidence remains insufficient to justify replacing simpler, validated alternatives, Table 3 summarizes all head-to-head comparisons identified in this review, reporting for each study the ML model and its performance, the comparator tool or clinical benchmark, and the magnitude of any observed difference.

This review also has several limitations. First, as a narrative review, it does not provide the quantitative synthesis of a systematic review or meta-analysis, and the conclusions are therefore based on qualitative interpretation of the available evidence. Second, although predefined eligibility criteria, a structured literature search, and standardized data extraction procedures were used, the absence of prospective protocol registration may have increased the risk of selection or reporting bias. Third, study selection, data extraction, and methodological assessment were performed by the review authors and may therefore be subject to residual reviewer judgment despite the use of standardized assessment frameworks. Finally, substantial heterogeneity in study populations, predictor variables, machine learning methods, outcome definitions, validation strategies, and reported performance metrics precluded direct quantitative comparison between studies.

## 5. Conclusions

Although the included studies demonstrate potential for ML, important limitations and unresolved questions remain, and much more research is needed to demonstrate its comprehensive real-world clinical utility. The accompanying PROBAST(+AI) assessment further demonstrated that many published prediction models remain at moderate to high risk of bias, particularly due to limitations in study design, external validation, and analytical methods. The significant interest in this topic is not without reason [17]. The studies included in this review represent early-stage developments that highlight the breadth of potential ML applications in CKD. Translating them into routine nephrology practice will hinge on methodological rigor, external validation, transparent reporting, and prospective clinical studies rather than on algorithmic novelty alone.

## Figures and Tables

**Table 1:** (**a**) Overview of machine learning applications in chronic kidney disease diagnosis. (**b**) Overview of machine learning applications in chronic kidney disease prognosis.

(a)									
Data Modality	Domain	Representative Studies	Population/Dataset	ML Approaches	Primary Clinical Task	Representative Performance *	External Validation	Main Strengths	Major Limitations/Translational Barriers
Clinical and laboratory data	Demographics, eGFR, UACR, comorbidities, laboratory biomarkers	Chen et al. [19], Polat et al. [20], Singh et al. [77], Vásquez-Morales et al. [21], Yoshizaki et al. [25]	386 patients to >1,000,000 individuals	SVM, RF, XGBoost, LightGBM, neural networks	CKD detection, staging, and incident CKD prediction	AUROC generally 0.90–0.99	Limited	Routinely available data; scalable; compatible with EHR systems	Predominantly retrospective studies; limited multicenter validation; frequent reliance on public datasets
Clinical data + proteomics/spectrometry	Etiology differentiation	Zhang et al. [26], Glazyrin et al. [27]	Chinese CKD cohorts; proteomic datasets	RF, ensemble learning, spectrometry-assisted ML	Diabetic vs. non-diabetic nephropathy	AUROC up to 0.92	Minimal	May reduce diagnostic uncertainty and biopsy burden	Small cohorts; limited ethnic diversity
Clinical chemistry	GFR estimation	Zhao et al. [28], Lanot et al. [29]	CKD cohorts	RF, ensemble models	eGFR estimation	P30 accuracy up to 74%	Limited	Alternative estimation strategies	Did not consistently outperform CKD-EPI equations
Ultrasound	Structural kidney assessment	Tian et al. [31], Qin et al. [33]	CKD imaging cohorts	CNN, multimodal DL	CKD detection, fibrosis prediction	AUROC 0.86–0.92	Limited	Non-invasive imaging; automated interpretation	Few prospective multicenter validation studies
MRI/CT	Renal structure and tissue characterization	Lee et al. [42], Li et al. [43]	Imaging cohorts	CNN, deep learning	CKD staging, fibrosis	AUROC generally 0.94–0.99	Minimal	Detailed tissue characterization	Small pilot cohorts; limited external validation
Urine analysis	Biomarker analysis	Cakici et al. [35], Gholizadeh et al. [38], Zhang et al. [40]	Urinary proteomics, Raman spectroscopy, routine urinalysis	RF, SVM, CNN	CKD diagnosis, subtype classification	AUROC generally 0.90–0.95	Limited	Non-invasive biomarker assessment	Heterogeneous analytical platforms
Histopathology	Digital pathology	Kolachalama et al. [47], Hermsen et al. [48]	Kidney biopsy cohorts	CNN, DL	Fibrosis quantification, eGFR prediction	Dice coefficient up to 0.91; AUROC up to 0.96	Limited	Automated tissue characterization	Limited biopsy availability; annotation-intensive
At-home monitoring	Wearables/mobile health	Kwon et al. [53], others	Home monitoring datasets	RF, ANN	Remote CKD screening	AUROC approximately 0.85–0.91	Minimal	Accessible; continuous monitoring	Limited clinical validation
Retinal imaging	Fundus photography	Rim et al. [55], Sabanayagam et al. [57]	Population-based retinal cohorts	CNN	CKD detection	AUROC generally 0.80–0.95	Present in several studies	Completely non-invasive; large screening potential	Limited prospective implementation studies
**(b)**									
**Data Modality**	**Domain**	**Representative Studies**	**Population/Dataset**	**ML Approaches**	**Primary Clinical Task**	Representative Performance *	**External Validation**	**Main Strengths**	**Major Limitations/Translational Barriers**
Prognosis	CKD progression	Bellocchio et al. [64], Segal et al. [65], Tangri et al. [66], Li et al. [67], Wang et al. [68]	CKD cohorts	RF, XGBoost, RNN, Naïve Bayes	Kidney failure, eGFR decline	AUROC generally 0.81–0.96	Moderate	Longitudinal prediction	Variable outcome definitions; calibration is infrequently reported
Prognosis	Complications and mortality	Chang et al. [69], Lu et al. [70], Hsu et al. [71], Oh et al. [72], Zhu et al. [73], Tran et al. [74]	CKD cohorts	XGBoost, GBM, ANN, Naïve Bayes	Hyperkalemia, sarcopenia, osteoporosis, postoperative CKD, cardiovascular disease, and mortality	AUROC generally 0.81–0.93	Limited	Broad range of clinically relevant outcomes	Few prospective implementation studies

Representative performance values summarize the range most consistently reported across studies rather than a single highest metric. AUROC is reported for classification tasks whenever available. Task-specific metrics (e.g., the Dice coefficient for image segmentation or P30 accuracy for estimated glomerular filtration rate prediction) are reported only when AUROC is not applicable. Abbreviations: ANN, artificial neural network; AUROC, area under the receiver operating characteristic curve; CKD, chronic kidney disease; CNN, convolutional neural network; CT, computed tomography; DL, deep learning; eGFR, estimated glomerular filtration rate; EHR, electronic health record; GBM, gradient boosting machine; LightGBM, Light Gradient Boosting Machine; ML, machine learning; MRI, magnetic resonance imaging; P30, percentage of estimated glomerular filtration rate values within 30% of the reference measured glomerular filtration rate; RF, random forest; RNN, recurrent neural network; SVM, support vector machine; UACR, urinary albumin-to-creatinine ratio; XGBoost, Extreme Gradient Boosting. * Representative performance values refer to the best reported results in the cited studies. Performance metrics (e.g., AUROC, Dice coefficient, P30 accuracy) are shown as reported by the original authors and should not be interpreted as directly comparable because of differences in study populations, datasets, outcome definitions, validation strategies, and machine learning methodologies.

**Table 2:** Summary of domain-level PROBAST(+AI) findings across the included studies.

PROBAST(+AI) Domain	Most Frequent Methodological Concerns	Overall Judgement
Participants	Predominantly retrospective single-center cohorts; frequent use of publicly available datasets; insufficient reporting of participant selection	Frequent concern
Predictors	Generally, clinically relevant predictors; occasional insufficient description of preprocessing and feature selection	Low to moderate concern
Outcome	Heterogeneous CKD definitions; inconsistent outcome ascertainment; variable endpoint definitions	Frequent concern
Analysis	Limited external validation; inadequate handling of missing data; insufficient assessment of overfitting; calibration rarely reported	Highest risk of bias
Applicability	Limited evidence of generalizability across healthcare systems and patient populations	Moderate to high concern

**Table 3:** Head-to-head comparisons of machine learning models with validated clinical tools or clinician judgment were identified in this review.

Study	Clinical Task	ML Model (Performance)	Comparator (Performance)	Difference (ML vs. Comparator)	Direction of Benefit	Formal Statistical Comparison Reported
Zhao et al. [28]	eGFR estimation	Ensemble model, P30 accuracy 58.9%	Asian-modified CKD-EPI equation, P30 accuracy 74.1%	P30 accuracy 15.2 percentage points lower (statistical significance not reported)	ML inferior	No
Lanot et al. [29]	eGFR estimation	Random forest, P10 accuracy below 60%	Creatinine-based eGFR equation	Performance below the validated creatinine-based eGFR equation (statistical significance not reported)	ML inferior	No
Tian et al. [31]	CKD detection on ultrasound	CNN, AUROC 0.918	Senior physicians, AUROC 0.869 (*p* < 0.001)	AUROC +0.049 (*p* < 0.001)	ML superior (largest gains in early stages)	Yes (*p* < 0.001)
Qin et al. [33]	Renal fibrosis prediction	Multimodal ultrasound DL, AUROC 0.86	Clinical model (eGFR +24 h proteinuria), AUROC 0.80	AUROC +0.06 (difference not statistically significant)	Comparable	Yes (not significant)
Kolachalama et al. [47]	eGFR-stage classification from biopsy	CNN, accuracy 0.649 (κ 0.519)	PEFS-based classifier, accuracy 0.345 (κ 0.051)	Accuracy +0.304; κ +0.468 (statistical significance not reported)	ML superior	No
Bellocchio et al. [64]	6-month kidney failure prediction	Naïve Bayes classifier	Kidney Failure Risk Equation (KFRE)	AUROC +0.149 (*p* = 0.0013)	ML superior	Yes (*p* = 0.0013)
Bellocchio et al. [64]	24-month kidney failure prediction	Naïve Bayes classifier, AUROC 0.96	Expert clinicians, mean AUROC 0.79	AUROC +0.17 (statistical significance not reported)	ML superior	No
Chang et al. [69]	Hyperkalemia prediction	XGBoost, AUROC 0.876	Two nephrologists, AUROC 0.745 and 0.741	AUROC +0.13 versus nephrologists (statistical significance not reported)	ML superior	No

Abbreviations: AUROC, area under the receiver operating characteristic curve; P10, percentage of estimates within 10% of the reference value; P30, percentage of estimates within 30% of the reference value; κ, Cohen’s kappa. Performance metrics differ across studies and are therefore not directly comparable. Reported differences are descriptive unless statistical significance was explicitly reported in the original study. Because the included studies reported heterogeneous performance metrics (including AUROC, accuracy, P10, P30, Cohen’s κ, Dice coefficient, c-index, and F1-score), the reported performance values are presented for descriptive purposes only. No statistical harmonization or quantitative comparison across studies was performed. The “Formal statistical comparison reported” column indicates whether the original study reported a formal statistical comparison between models (with the corresponding *p*-value where applicable).

## Data Availability

No new data were created or analyzed in this study. Data sharing is not applicable to this article.

## Supplementary Materials

The following supporting information can be downloaded at: https://www.mdpi.com/article/10.3390/diagnostics16152354/s1. Figure S1. PRISMA 2020 flow diagram. Table S1: Complete database-specific search strategies, search date, and number of retrieved records. Table S2. Methodological criteria used to interpret studies reporting exceptionally high predictive performance.

## Abbreviations

The following abbreviations are used in this manuscript:

ACEI	Angiotensin-converting enzyme inhibitor
ACR	Albumin-to-creatinine ratio
AI	Artificial intelligence
ANN	Artificial neural network
ARB	Angiotensin receptor blocker
AUC	Area under the curve
AUROC	Area under the receiver operating characteristic curve
BP	Blood pressure
CKD	Chronic kidney disease
CKD-EPI	Chronic Kidney Disease Epidemiology Collaboration
CNN	Convolutional neural network
COPD	Chronic obstructive pulmonary disease
CT	Computed tomography
DL	Deep learning
DN	Diabetic nephropathy
DT	Decision tree
EHR	Electronic health record
eGFR	Estimated glomerular filtration rate
ESKD	End-stage kidney disease
GBM	Gradient boosting machine
GEO	Gene Expression Omnibus
GFR	Glomerular filtration rate
HR-pQCT	High-resolution peripheral quantitative computed tomography
KDIGO	Kidney Disease: Improving Global Outcomes
KFRE	Kidney Failure Risk Equation
KNN	k-nearest neighbor
KRT	Kidney replacement therapy
LASSO	Least absolute shrinkage and selection operator
LightGBM	Light Gradient Boosting Machine
LR	Logistic regression
LSTM	Long short-term memory
ML	Machine learning
MLP	Multilayer perceptron
MRI	Magnetic resonance imaging
NGAL	Neutrophil gelatinase-associated lipocalin
NN	Neural network
PEFS	Pathologist-estimated fibrosis score
RAAS	Renin–angiotensin–aldosterone system
RCT	Randomized controlled trial
RF	Random forest
RNN	Recurrent neural network
RRT	Renal replacement therapy
SDI	Socio-demographic index
SERS	Surface-Enhanced Raman Spectroscopy
SGLT-2	Sodium/glucose cotransporter 2
SHAP	Shapley Additive Explanations
SIMCA	Soft independent modeling by class analogy
SVM	Support vector machine
UACR	Urine albumin-to-creatinine ratio
US	Ultrasound
US-CDI	Ultrasound and color Doppler imaging
XGBoost	Extreme Gradient Boosting
