# Supplementary material for: Applications of Machine Learning for Early Diagnosis and Prognosis of Chronic Kidney Disease: Current Evidence
Source: Diagnostics (Basel). 2026 Jul 27;16(15):2354. doi: 10.3390/diagnostics16152354 (PMC13464806; doi:10.3390/diagnostics16152354)
Supplement: Supplementary file 1 [file diagnostics-16-02354-s001.zip › diagnostics-4367106-supplementary.pdf]

Figure S1. PRISMA 2020 flow diagram

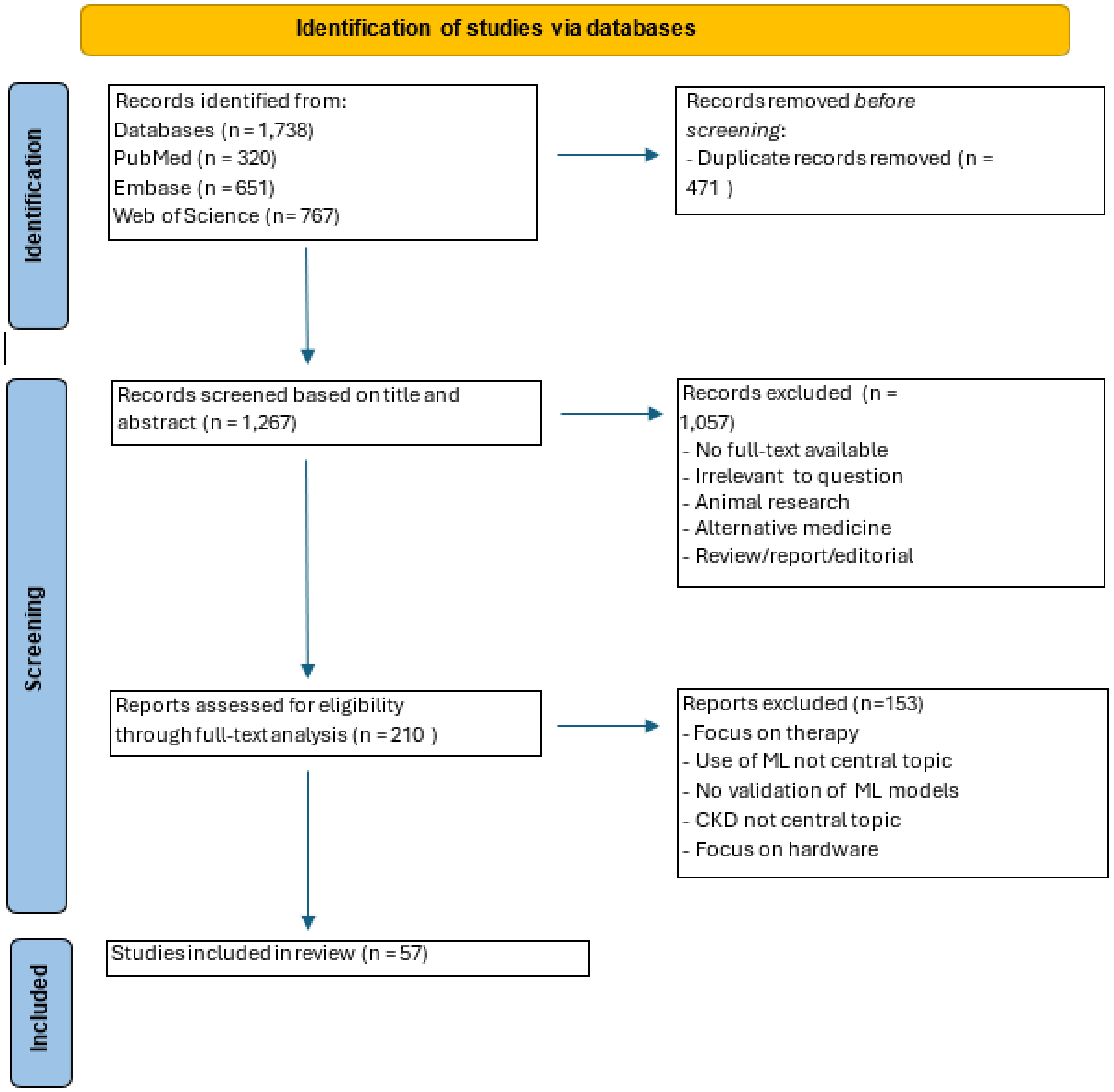

Table S1: Complete database-specific search strategies, search date, and number of retrieved records.

Database	Complete search strategy (verbatim)	Search date	Retrieved records
PubMed (Free Text)	("chronic kidney disease" OR CKD OR "chronic renal disease") AND ("machine learning" OR "artificial intelligence" OR "deep learning") Filters: Publication date from 1 January 2016 to 8 April 2025; Humans.	8 April 2025	215
PubMed (MeSH)	("Renal Insufficiency, Chronic"[Majr]) AND ("Machine Learning"[Majr]) Filters: Publication date from 1 January 2016 to 8 April 2025; Humans.	8 April 2025	105
Embase (Quick Search)	'chronic kidney disease' AND 'machine learning' Limited to articles published between 1 January 2016 and 8 April 2025.	8 April 2025	484
Embase (Emtree)	'chronic kidney failure'/mj AND 'machine learning'/mj Limited to articles published between 1 January 2016 and 8 April 2025.	8 April 2025	167
Web of Science Core Collection	TS=("chronic kidney disease") AND TS=("machine learning") AND DT=(Article) Timespan: 2016-2025.	8 April 2025	767

Table S2. Methodological criteria used to interpret studies reporting exceptionally high predictive performance

Criterion	Interpretation
Small sample size	May increase the risk of overfitting and unstable performance estimates.
Reuse of publicly available benchmark datasets (e.g., UCI CKD dataset)	Repeated use may result in optimistic performance estimates and reduced generalizability.
Lack of independent external validation	Generalizability cannot be established.
Internal validation only	Apparent performance may overestimate real-world performance.
Potential data leakage	May artificially inflate predictive performance.
Near-perfect discrimination (e.g., AUROC ≥0.99 or accuracy ≥99%)	Requires careful methodological scrutiny rather than being interpreted as evidence of superior clinical performance.
Calibration not reported	Reliable individual risk estimation cannot be assessed.
Clinical utility not evaluated	Improved discrimination alone does not demonstrate clinical benefit.

PROBAST-AI TOOL ()

Chen et al. (19)

Step 1 - PICOTS

**Population**	386 patients from the UCI CKD dataset
**Index model(s)**	KNN, SVM, SIMCA

**Comparator model(s)**	Three models compared against each other
**Outcome(s)**	CKD or non-CKD
**Timing**	Cross-sectional
**Setting and intended use of the prediction model**	Community-based screening

Step 2 – Classify the type of prediction model assessment

**Classify the assessment based on its aim**			
Type of prediction study	***Explanation***	***PROBAST+AI boxes to complete***	***Tick as appropriate***
**Development only**	**Prediction model development only, i.e., without evaluation of its performance.**	**Model development**	
**Evaluation only**	**External validation of one or more existing models in new data**	**Model evaluation**	
**Combination**	**Prediction model development combined in the same study (publication) with the evaluation of its apparent performance, internal validation performance, or external validation performance.**	**Model development and evaluation**	**x**

Step 3 - Assess quality and applicability or risk of bias and applicability

**Publication reference**	Chen ZW, Zhang X, Zhang ZY. Clinical risk assessment of patients with chronic kidney disease by using clinical data and multivariate models. Int Urol Nephrol. 2016;48(12):2069–75.
**Models of interest**	KNN, SVM, SIMCA
**Outcome of interest**	CKD or non-CKD

Model development

**DOMAIN 1: Participants and data sources**	
**A. Quality**	
UCI CKD dataset; 400 samples; 21 variables; 14 CKD cases removed due to missing variables	
	**Y/ PY/ PN/ N/ NI**

**1.1 Were appropriate data sources used?**	PY
**1.2 Was an appropriate study design used?**	PN
**1.3 Did the in- and exclusions of study participants result in a representative dataset?**	NI
**Concern regarding quality of selection of participants and data sources**	**QUALITYCONCERN:** *low/**high**/unclear*
Rationale of quality rating: unclear CKD definition, biased deletion	
**B. Applicability**	
Describe included data sources, participants, setting, and dates:<br>UCI (University of California Irvine) Dataset, no further information	
**Concern that the (data of the) included participants do not match the review question or the assessor's intended use of the prediction model**	**APPLICABILITY CONCERN:** *low/**high**/unclear*
Rationale of applicability rating: non-representative dataset, unclear patient selection	

**DOMAIN 2: Predictors**	
**A. Quality**	
21 clinical variables, nominal variables recoded into binary variables<br>*Age, red blood cell count and white blood cell count excluded*	
	**Y/ PY/ PN/ N/ NI**
**2.1 Were predictors defined and assessed in a similar way for all participants?**	NI
**2.2 Was any pre-processing of predictors similar for all participants?**	NI
**2.3 Were predictor assessments made without knowledge of outcome data?**	NI
**2.4 Were the predictors included in the model available at the time the model was intended to be used?**	NI
**Concern regarding quality of selection of participants and data sources**	**QUALITYCONCERN:** *low/**high**/unclear*
Rationale of quality rating: *removal of clinically relevant predictors*	
**B. Applicability**	
**Concern that the definition, pre-processing, assessment, or timing of assessment of the predictors in the model do not match the review question or the assessor's intended use**	**APPLICABILITY CONCERN:** *low/**high**/unclear*
Rationale of applicability rating: *Predictors are clinically relevant but altered*	

**DOMAIN 3: Outcome**
**A. Quality**
Binary CKD vs. non-CKD label from UCI dataset

	Y/ PY/ PN/ N/ NI
**3.1 Were outcomes defined and assessed appropriately?**	N
**3.2 Were outcomes defined and assessed in a similar way for all participants?**	Y
**3.3 Were outcome assessments made without use or knowledge of predictor data?**	NI
**3.4 Was the time interval between predictor assessment and outcome assessment appropriate?**	NI
**Concern regarding quality of the outcome or its determination**	**QUALITYCONCERN:** *low/**high**/unclear*
Rationale of quality rating: *no clear CKD definition, no reference to guidelines, public database without clear data-management*	
**B. Applicability**	
Unclear how and when outcome was determined	
**Concern that the outcome, its definition, assessment, or timing of assessment do not match the review question or the assessor's intended use**	**APPLICABILITY CONCERN:** *low/**high**/unclear*
Rationale of applicability rating: *Outcome definition not comparable with clinical definition*	

**DOMAIN 4: Analysis**	
**A. Quality**	
Describe the numbers of participants, number of candidate predictors, number of outcome events:<br>*386 samples, 21 predictors, 251 CKD, 149 non-CKD.*	
Describe how the prediction model was developed):<br>*KNN (k=3), SVM (RBF kernel, C=12, γ=1), SIMCA (6 PCs).*	
Describe the performance measures of the prediction model:<br>*Accuracy, sensitivity, specificity*	
Describe missing data on predictors and outcomes as well as methods used for handling these missing data:<br>*Mean imputation within class, deletion of 14 CKD cases.*	
	**Y/ PY/ PN/ N/ NI**
**4.1 Was there evidence that the sample size was reasonable?**	PN
**4.2 Were continuous and categorical predictors handled appropriately?**	PY
**4.3 Were participants with missing or censored data handled appropriately in the analysis?**	N
**4.4 If methods to address class imbalance were used, was the model or the model predictions recalibrated?***	NA
**4.5 Were methods used to address potential model overfitting?**	N
**Concern regarding quality of the analysis**	**QUALITYCONCERN:** *low/**high**/unclear*
Rationale of quality rating: *Mean imputation, case deletion, limited validation.*	

Model evaluation

Domain 1,2,3 same analysis as model development

**DOMAIN 4: Analysis**			
**Risk of bias**			
Describe numbers of participants, number of predictors, outcome events and events per predictor:<br>*386 patients, 21 variables, CKD vs non-CKD*			
Describe the performance measures of the evaluated model:<br>*Accuracy, sensitivity, specificity*			
Describe any participants who were excluded from the analysis:<br>*No information, multiple missing variables*			
Describe missing data on predictors and outcomes as well as methods used for handling these missing data:<br>*Mean imputation within class, deletion of 14 CKD cases.*			
	**Y/ PY/ PN/ N/ NI/ NA**		
**4.1 Was model evaluation based on only apparent performance avoided?**	NI		
	**A**	**I**	**E**
**4.2 Was there evidence that the sample size was reasonable?**	PN	PN	NA
**4.3 Were participants with missing or censored data handled appropriately in the analysis?**	N	N	NA
**4.4 If methods to address class imbalance were used, was the evaluation done in a dataset without imbalance correction?***	NA	NA	NA
**4.5 If data splitting was done to create training and test datasets, was there evidence that data leakage was avoided?***	**NA**	PN	**NA**
**4.6 If resampling methods were used to evaluate model performance, were all model development steps replicated in the resampling process?***	**NA**	NA	**NA**
**4.7 Was the predictive performance of the model evaluated appropriately, e.g., calibration, discrimination, and net benefit?**	PN	PN	NA
**Risk of bias introduced by the analysis**	**RISK OF BIAS:**<br>*low/**high**/unclear*		
Rationale of risk of bias rating:<br>No external validation.<br>Internal validation based on random noise addition<br>Missing data were handled using simple mean imputation and case deletion.			

Step 4 - Assess the overall concerns regarding quality, risk of bias and applicability of the prediction model

**OVERALL JUDGEMENT OF PREDICTION MODEL**	
**Overall judgement of quality (development)**	**QUALITY CONCERN:**<br>*low/**high**/unclear*
Summary of quality concern: Non-representative dataset, poor missing data handling, outcome not clinically defined.	
**Overall judgement of risk of bias (evaluation)**	**RISK OF BIAS:**<br>*low/**high**/unclear*
Summary of sources of potential bias: No external validation, inappropriate outcome definition.	
**Overall judgement of applicability (development)**	**APPLICABILITY CONCERN:**<br>*low/**high**/unclear*
Summary of applicability concern: Dataset and outcome do not match intended clinical use.	

**Overall judgement of applicability (evaluation)**	**APPLICABILITY CONCERN:** *low/**high**/unclear*
Summary of applicability concern: Same issues as development, not clinically deployable.	

Polat et al. (20)

Step 1 - PICOTS

**Population**	Adults with suspected or confirmed CKD (UCI dataset)
**Index model(s)**	SVM (with or without feature selection methods)
**Comparator model(s)**	No index, comparison against each other
**Outcome(s)**	CKD or non-CKD
**Timing**	Cross-sectional
**Setting and intended use of the prediction model**	Not specified

Step 2 – Classify the type of prediction model assessment

**Classify the assessment based on its aim**			
Type of prediction study	***Explanation***	***PROBAST+AI boxes to complete***	***Tick as appropriate***
**Development only**	**Prediction model development only, i.e., without evaluation of its performance.**	**Model development**	
**Evaluation only**	**External validation of one or more existing models in new data**	**Model evaluation**	
**Combination**	**Prediction model development combined in the same study (publication) with the evaluation of its apparent performance, internal validation performance, or external validation performance.**	**Model development and evaluation**	**x**

Step 3 - Assess quality and applicability or risk of bias and applicability

**Publication reference**	Polat H, Danaei Mehr H, Cetin A. Diagnosis of Chronic Kidney Disease Based on Support Vector Machine by Feature Selection Methods. J Med Syst. 2017;41(4):55.
**Models of interest**	SVM with different feature selection methods

Outcome of interest	CKD or non-CKD

Model development

DOMAIN 1: Participants and data sources	
A. Quality	
UCI CKD dataset; 400 samples; 21 variables; no design, no recruitment criteria, no timeline	
	**Y/ PY/ PN/ N/ NI**
**1.1 Were appropriate data sources used?**	PY
**1.2 Was an appropriate study design used?**	PN
**1.3 Did the in- and exclusions of study participants result in a representative dataset?**	NI
**Concern regarding quality of selection of participants and data sources**	**QUALITYCONCERN:** *low/**high**/unclear*
Rationale of quality rating: no information about collection, no recruitment critieria, no missing data reported	
**B. Applicability**	
Describe included data sources, participants, setting, and dates:<br>UCI (University of California Irvine) Dataset, no further information	
**Concern that the (data of the) included participants do not match the review question or the assessor's intended use of the prediction model**	**APPLICABILITY CONCERN:** *low/**high**/unclear*
Rationale of applicability rating: non-representative dataset, unclear patient selection (same critique as chen et al. (19)	

DOMAIN 2: Predictors	
A. Quality	
24 UCI attributes, final modal uses 13 features based on different feature selection methods	
	**Y/ PY/ PN/ N/ NI**
**2.1 Were predictors defined and assessed in a similar way for all participants?**	PY
**2.2 Was any pre-processing of predictors similar for all participants?**	NI
**2.3 Were predictor assessments made without knowledge of outcome data?**	NI
**2.4 Were the predictors included in the model available at the time the model was intended to be used?**	PY
**Concern regarding quality of selection of participants and data sources**	**QUALITYCONCERN:** ***low**/high/unclear*
Rationale of quality rating: *all attributes applied uniformly, data is pre-labelled, standard clinical or laboratory variables*	
**B. Applicability**	

**Concern that the definition, pre-processing, assessment, or timing of assessment of the predictors in the model do not match the review question or the assessor's intended use**	**APPLICABILITY CONCERN:** ***low**/high/unclear*
Rationale of applicability rating: *final selected predictors are standard/routine variables*	

**DOMAIN 3: Outcome**	
**A. Quality**	
Binary CKD vs. non-CKD label from UCI dataset	
	**Y/ PY/ PN/ N/ NI**
**3.1 Were outcomes defined and assessed appropriately?**	NI
**3.2 Were outcomes defined and assessed in a similar way for all participants?**	NI
**3.3 Were outcome assessments made without use or knowledge of predictor data?**	NI
**3.4 Was the time interval between predictor assessment and outcome assessment appropriate?**	NI
**Concern regarding quality of the outcome or its determination**	**QUALITYCONCERN:** *low/**high**/unclear*
Rationale of quality rating: *no clear CKD definition, no reference to guidelines, public database without clear data-management, no blinding, pre-labelled data*	
**B. Applicability**	
Unclear how and when outcome was determined	
**Concern that the outcome, its definition, assessment, or timing of assessment do not match the review question or the assessor's intended use**	**APPLICABILITY CONCERN:** *low/**high**/unclear*
Rationale of applicability rating: *Outcome definition not comparable with clinical definition*	

**DOMAIN 4: Analysis**	
**A. Quality**	
Describe the numbers of participants, number of candidate predictors, number of outcome events:<br>*400 samples, 24 predictors,*	
SVM. Feature selection: wrapper and filter. 10-fold cross-validation.	
Describe the performance measures of the prediction model:<br>*Accuracy, TP rate, FP rate, precision, recall, F-easure, AUROC*	
Describe missing data on predictors and outcomes as well as methods used for handling these missing data:<br>*No report*	
	**Y/ PY/ PN/ N/ NI**

**4.1 Was there evidence that the sample size was reasonable?**	PY
**4.2 Were continuous and categorical predictors handled appropriately?**	NI
**4.3 Were participants with missing or censored data handled appropriately in the analysis?**	N
**4.4 If methods to address class imbalance were used, was the model or the model predictions recalibrated?***	N
**4.5 Were methods used to address potential model overfitting?**	PN
**Concern regarding quality of the analysis**	**QUALITYCONCERN:** *low/**high**/unclear*
Rationale of quality rating: *missing data not addressed, class imbalance not addressed, feature selection before cross-validation no external validation*	

Model evaluation

Domain 1,2,3 same analysis as model development

**DOMAIN 4: Analysis**			
**Risk of bias**			
Describe numbers of participants, number of predictors, outcome events and events per predictor: *400 patients, 24 variables, CKD vs non-CKD, 250 CKD*			
Describe the performance measures of the evaluated model: *Accuracy, sensitivity, specificity*			
Describe any participants who were excluded from the analysis: *Accuracy, TP rate, FP rate, precision, recall, F-measure, ROC/AUC*			
Describe missing data on predictors and outcomes as well as methods used for handling these missing data: *Not reported*			
	**Y/ PY/ PN/ N/ NI/ NA**		
**4.1 Was model evaluation based on only apparent performance avoided?**	PN		
	**A**	**I**	**E**
**4.2 Was there evidence that the sample size was reasonable?**	NI	NI	NA
**4.3 Were participants with missing or censored data handled appropriately in the analysis?**	NI	NI	NA
**4.4 If methods to address class imbalance were used, was the evaluation done in a dataset without imbalance correction?***	N	N	NA
**4.5 If data splitting was done to create training and test datasets, was there evidence that data leakage was avoided?***	**NA**	PN	**NA**
**4.6 If resampling methods were used to evaluate model performance, were all model development steps replicated in the resampling process?***	**NA**	NA	**NA**
**4.7 Was the predictive performance of the model evaluated appropriately, e.g., calibration, discrimination, and net benefit?**	N	N	
**Risk of bias introduced by the analysis**	**RISK OF BIAS:** *low/**high**/unclear*		
Rationale of risk of bias rating: No external validation. Missing data unadressed No class imbalance correction			

Feature selection before CV

Step 4 - Assess the overall concerns regarding quality, risk of bias and applicability of the prediction model

OVERALL JUDGEMENT OF PREDICTION MODEL	
**Overall judgement of quality (development)**	**QUALITY CONCERN:**<br>*low/**high**/unclear*
Domain 1,3 and 4 high quality concerns	
**Overall judgement of risk of bias (evaluation)**	**RISK OF BIAS:**<br>*low/**high**/unclear*
Domain 1,3 and 4 high risk of bias	
**Overall judgement of applicability (development)**	**APPLICABILITY CONCERN:**<br>*low/**high**/unclear*
Population not well-described, no clinical setting, outcome not matched to standard diagnostic criteria	
**Overall judgement of applicability (evaluation)**	**APPLICABILITY CONCERN:**<br>*low/**high**/unclear*
Same issues as development, not clinically deployable.	

Singh et al. (21)

Step 1 - PICOTS

**Population**	Adults with suspected or confirmed CKD
**Index model(s)**	Neural network
**Comparator model(s)**	SVM, KNN, LR, RF, NB
**Outcome(s)**	CKD or non-CKD
**Timing**	Cross-sectional
**Setting and intended use of the prediction model**	Not specified

Step 2 – Classify the type of prediction model assessment

Classify the assessment based on its aim			
Type of prediction study	***Explanation***	***PROBAST+AI boxes to complete***	***Tick as appropriate***
**Development only**	**Prediction model development only, i.e., without evaluation of its performance.**	**Model development**	
**Evaluation only**	**External validation of one or more existing models in new data**	**Model evaluation**	

**Combination**	**Prediction model development combined in the same study (publication) with the evaluation of its apparent performance, internal validation performance, or external validation performance.**	**Model development and evaluation**	x

Step 3 - Assess quality and applicability or risk of bias and applicability

**Publication reference**	Singh V, Asari VK, Rajasekaran R. A Deep Neural Network for Early Detection and Prediction of Chronic Kidney Disease. Diagnostics. 2022;12(1):116.
**Models of interest**	DNN
**Outcome of interest**	CKD or non-CKD

Model development

**DOMAIN 1: Participants and data sources**	
**A. Quality**	
UCI CKD dataset; 400 samples; 21 variables; no design, no recruitment criteria, no timeline	
	**Y/ PY/ PN/ N/ NI**
**1.1 Were appropriate data sources used?**	PY
**1.2 Was an appropriate study design used?**	PN
**1.3 Did the in- and exclusions of study participants result in a representative dataset?**	NI
**Concern regarding quality of selection of participants and data sources**	**QUALITYCONCERN:** *low/**high**/unclear*
Rationale of quality rating: no information about collection, no recruitment criteria, no missing data reported	
**B. Applicability**	
Describe included data sources, participants, setting, and dates:<br>UCI (University of California Irvine) Dataset, no further information	
**Concern that the (data of the) included participants do not match the review question or the assessor's intended use of the prediction model**	**APPLICABILITY CONCERN:** *low/**high**/unclear*
Rationale of applicability rating: non-representative dataset, unclear patient selection (same critique as chen et al. (19) and Polat et al. (20)	

**DOMAIN 2: Predictors**
**A. Quality**

24 UCI attributes, final modal uses 7 features after RFE feature selection, data preprocessing described: : mean imputation (numerical), mode imputation (nominal), IQR-based outlier removal, z-score normalisation,	
	**Y/ PY/ PN/ N/ NI**
**2.1 Were predictors defined and assessed in a similar way for all participants?**	PY
**2.2 Was any pre-processing of predictors similar for all participants?**	Y
**2.3 Were predictor assessments made without knowledge of outcome data?**	NI
**2.4 Were the predictors included in the model available at the time the model was intended to be used?**	PY
**Concern regarding quality of selection of participants and data sources**	**QUALITYCONCERN:** ***low****/high/unclear*
Rationale of quality rating: *all attributes applied uniformly, data is pre-labelled, standard clinical or laboratory variables*	
**B. Applicability**	
**Concern that the definition, pre-processing, assessment, or timing of assessment of the predictors in the model do not match the review question or the assessor's intended use**	**APPLICABILITY CONCERN:** ***low****/high/unclear*
Rationale of applicability rating: *final selected predictors are standard/routine variables*	

**DOMAIN 3: Outcome**	
**A. Quality**	
Binary CKD vs. non-CKD label from UCI dataset	
	**Y/ PY/ PN/ N/ NI**
**3.1 Were outcomes defined and assessed appropriately?**	NI
**3.2 Were outcomes defined and assessed in a similar way for all participants?**	NI
**3.3 Were outcome assessments made without use or knowledge of predictor data?**	NI
**3.4 Was the time interval between predictor assessment and outcome assessment appropriate?**	NI
**Concern regarding quality of the outcome or its determination**	**QUALITYCONCERN:** *low/****high****/unclear*
Rationale of quality rating: *no clear CKD definition, no reference to guidelines, public database without clear data-management, no blinding, pre-labelled data*	
**B. Applicability**	
Unclear how and when outcome was determined	
**Concern that the outcome, its definition, assessment, or timing of assessment do not match the review question or the assessor's intended use**	**APPLICABILITY CONCERN:** *low/****high****/unclear*
Rationale of applicability rating: *Outcome definition not comparable with clinical definition*	

DOMAIN 4: Analysis	
A. Quality	
Describe the numbers of participants, number of candidate predictors, number of outcome events:<br>*400 samples, 250 CKD events, 75% training/25% testing*	
DNN: 12 layer. Feature selection: RFE applied before train/test split, potential leakage not discussed.	
Describe the performance measures of the prediction model:<br>*Accuracy, recall, precision, F-measure, AUC/ROC*	
Describe missing data on predictors and outcomes as well as methods used for handling these missing data:<br>*Missing values imputed (mean for numerical, mode for nominal). IQR-based outlier removal applied.*	
	**Y/ PY/ PN/ N/ NI**
**4.1 Was there evidence that the sample size was reasonable?**	PY
**4.2 Were continuous and categorical predictors handled appropriately?**	Y
**4.3 Were participants with missing or censored data handled appropriately in the analysis?**	PY
**4.4 If methods to address class imbalance were used, was the model or the model predictions recalibrated?***	N
**4.5 Were methods used to address potential model overfitting?**	PN
**Concern regarding quality of the analysis**	**QUALITYCONCERN:**<br>*low/**high**/unclear*
Rationale of quality rating: *impact data imputation and outlier removal not addressed, class imbalance not addressed, feature selection before data split, no external validation*	

Model evaluation

Domain 1,2,3 same analysis as model development

DOMAIN 4: Analysis			
Risk of bias			
Describe numbers of participants, number of predictors, outcome events and events per predictor:<br>*400 patients(75/25 split), 7 final predictions, 33 CKD events in test set*			
Describe the performance measures of the evaluated model:<br>*Accuracy, sensitivity, specificity*			
Describe any participants who were excluded from the analysis:<br>*Accuracy, TP rate, FP rate, precision, recall, F-measure, ROC/AUC*			
Describe missing data on predictors and outcomes as well as methods used for handling these missing data:<br>*Missing data imputed before analysis, effect not assessed*			
	**Y/ PY/ PN/ N/ NI/ NA**		
**4.1 Was model evaluation based on only apparent performance avoided?**	PN		
	**A**	**I**	**E**
**4.2 Was there evidence that the sample size was reasonable?**	NI	NI	NA
**4.3 Were participants with missing or censored data handled appropriately in the analysis?**	PY	PY	NA

**4.4 If methods to address class imbalance were used, was the evaluation done in a dataset without imbalance correction?***	N	N	NA
**4.5 If data splitting was done to create training and test datasets, was there evidence that data leakage was avoided?***	**NA**	PN	**NA**
**4.6 If resampling methods were used to evaluate model performance, were all model development steps replicated in the resampling process?***	**NA**	N	**NA**
**4.7 Was the predictive performance of the model evaluated appropriately, e.g., calibration, discrimination, and net benefit?**	PN	PN	
**Risk of bias introduced by the analysis**	**RISK OF BIAS:** *low/**high**/unclear*		
Rationale of risk of bias rating:<br>No external validation.<br>Missing data imputed before evaluation<br>No class imbalance correction<br>Feature selection before dataset splitting<br>100% accuracy implausible			

Step 4 - Assess the overall concerns regarding quality, risk of bias and applicability of the prediction model

OVERALL JUDGEMENT OF PREDICTION MODEL	
**Overall judgement of quality (development)**	**QUALITY CONCERN:** *low/**high**/unclear*
Domain 1,3 and 4 high quality concerns	
**Overall judgement of risk of bias (evaluation)**	**RISK OF BIAS:** *low/**high**/unclear*
Domain 1,3 and 4 high risk of bias	
**Overall judgement of applicability (development)**	**APPLICABILITY CONCERN:** *low/**high**/unclear*
Domain 1 and domain 3 high applicability concerns	
**Overall judgement of applicability (evaluation)**	**APPLICABILITY CONCERN:** *low/**high**/unclear*
Same issues as development, not clinically deployable.	

1. Vasquez-Morales et al. (22)

Step 1 - PICOTS

**Population**	Colombian Adults with suspected or confirmed CKD (ICD-10 subgroup N18)
**Index model(s)**	Neural network
**Comparator model(s)**	SVM, RF
**Outcome(s)**	CKD or no CKD
**Timing**	Retrospective
**Setting and intended use of the prediction model**	Colombian population, national health system, CKD

Step 2 – Classify the type of prediction model assessment

Classify the assessment based on its aim			
Type of prediction study	***Explanation***	***PROBAST+AI boxes to complete***	***Tick as appropriate***
**Development only**	**Prediction model development only, i.e., without evaluation of its performance.**	**Model development**	
**Evaluation only**	**External validation of one or more existing models in new data**	**Model evaluation**	
**Combination**	**Prediction model development combined in the same study (publication) with the evaluation of its apparent performance, internal validation performance, or external validation performance.**	**Model development and evaluation**	**x**

Step 3 - Assess quality and applicability or risk of bias and applicability

**Publication reference**	Vásquez-Morales GR, Martínez-Monterrubio SM, Moreno-Ger P, Recio-García JA. Explainable Prediction of Chronic Renal Disease in the Colombian Population Using Neural Networks and Case-Based Reasoning. Ieee Access. 2019;7:152900–10
**Models of interest**	NN, SVM, RF
**Outcome of interest**	CKD or non-CKD

Model development

**DOMAIN 1: Participants and data sources**	
**A. Quality**	
UCI CKD dataset; 400 samples; 21 variables; no design, no recruitment criteria, no timeline	
	**Y/ PY/ PN/ N/ NI**
**1.1 Were appropriate data sources used?**	Y
**1.2 Was an appropriate study design used?**	PY
**1.3 Did the in- and exclusions of study participants result in a representative dataset?**	PY
**Concern regarding quality of selection of participants and data sources**	**QUALITYCONCERN:** ***low***/*high*/*unclear*
Rationale of quality rating: validated national health information system, CKD cases defined by ICD-10 N18, contrals filtered to exclude prior diagnoses, sampling method described	
**B. Applicability**	

Describe included data sources, participants, setting, and dates:<br>RIPS database Colombia 2009-2019, n= 40,000 (20,000 CKD), CKD defined ICD-10 N18	
**Concern that the (data of the) included participants do not match the review question or the assessor's intended use of the prediction model**	**APPLICABILITY CONCERN:**<br>***low****/high/unclear*
Rationale of applicability rating: high number of patients, no class imbalance, random sampling	

**DOMAIN 2: Predictors**	
**A. Quality**	
7,492 input variables: demographic data and aggregated history of ICD-10 disease, medical visits<br>*15 most discriminant variables selected by random forest*	
	**Y/ PY/ PN/ N/ NI**
**2.1 Were predictors defined and assessed in a similar way for all participants?**	PY
**2.2 Was any pre-processing of predictors similar for all participants?**	PY
**2.3 Were predictor assessments made without knowledge of outcome data?**	NI
**2.4 Were the predictors included in the model available at the time the model was intended to be used?**	PY
**Concern regarding quality of selection of participants and data sources**	**QUALITYCONCERN:**<br>***low****/high/unclear*
Rationale of quality rating: *all attributes applied uniformly, pre-labelled, routine health records, clinically available*	
**B. Applicability**	
**Concern that the definition, pre-processing, assessment, or timing of assessment of the predictors in the model do not match the review question or the assessor's intended use**	**APPLICABILITY CONCERN:**<br>***low****/high/unclear*
Rationale of applicability rating: *real administrative data, may be limited other populations, approach transferable*	

**DOMAIN 3: Outcome**	
**A. Quality**	
Binary CKD vs. non-CKD, CKD diagnosis 2018 used as case identification, outcome is administrative diagnosis	
	**Y/ PY/ PN/ N/ NI**
**3.1 Were outcomes defined and assessed appropriately?**	PY
**3.2 Were outcomes defined and assessed in a similar way for all participants?**	PY
**3.3 Were outcome assessments made without use or knowledge of predictor data?**	NI
**3.4 Was the time interval between predictor assessment and outcome assessment appropriate?**	PN
**Concern regarding quality of the outcome or its determination**	**QUALITYCONCERN:**<br>*low/****high****/unclear*

Rationale of quality rating: *ICD-10 N18 widely accepted definition, retrospective data, blinding not discussed, diagnoses from 2009-2018 included, same year as outcome definition: risk outcome contamination*
**B. Applicability**
CKD outcome defined as ICD-10 N18 diagnosis in 2018, risking temporal overlap with outcome definition.

**Concern that the outcome, its definition, assessment, or timing of assessment do not match the review question or the assessor's intended use**	**APPLICABILITY CONCERN:** *low/**high**/unclear*
Rationale of applicability rating: *ICD-10 N18 is valid definition, but administrative instead of clinical, risk of missing early-stage CKD, risk predictors and outcome from same year (2018).*	

**DOMAIN 4: Analysis**	
**A. Quality**	
Describe the numbers of participants, number of candidate predictors, number of outcome events: n=40,000 (20,000 CKD, 20,000 controls); 7,492 candidate predictors; Training: 70% (n=28,000); test: 30% (n=12,000).	
NN: 5 layers	
Describe the performance measures of the prediction model:<br>*Accuracy, sensitivity, specificity, precision, recall, F-measure, AUC/ROC reported for NN, SVM and Random Forest*	
Describe missing data on predictors and outcomes as well as methods used for handling these missing data:<br>*Null values in RIPS described; treatment mentioned but specific strategy not detailed.*	
	**Y/ PY/ PN/ N/ NI**
**4.1 Was there evidence that the sample size was reasonable?**	PY
**4.2 Were continuous and categorical predictors handled appropriately?**	PY
**4.3 Were participants with missing or censored data handled appropriately in the analysis?**	NI
**4.4 If methods to address class imbalance were used, was the model or the model predictions recalibrated?***	Y
**4.5 Were methods used to address potential model overfitting?**	PN
**Concern regarding quality of the analysis**	**QUALITYCONCERN:** *low/high/**unclear***
Rationale of quality rating: *n=40,000 is large, null value treatment described, applied uniformly, null value handling mentioned but specific strategy not detailed, effect on results not assessed, dataset deliberately balanced, no external validation, predictor and outcome from same year (leakage risk).*	

Model evaluation

Domain 1,2,3 same analysis as model development

**DOMAIN 4: Analysis**
**Risk of bias**
Describe numbers of participants, number of predictors, outcome events and events per predictor: Test set: n=12,000); 7,492 predictors, CKD events in test set ~ 6,000

Describe the performance measures of the evaluated model:<br>*Accuracy, sensitivity, specificity*			
Describe any participants who were excluded from the analysis:<br>*No explicit exclusions from the test set reported.*			
Describe missing data on predictors and outcomes as well as methods used for handling these missing data:<br>*Null values treated in preprocessing, no formal missing data analysis*			
	**Y/ PY/ PN/ N/ NI/ NA**		
**4.1 Was model evaluation based on only apparent performance avoided?**	PN		
	**A**	**I**	**E**
**4.2 Was there evidence that the sample size was reasonable?**	Y	Y	NA
**4.3 Were participants with missing or censored data handled appropriately in the analysis?**	NI	NI	NA
**4.4 If methods to address class imbalance were used, was the evaluation done in<br>a dataset without imbalance correction?***	NA	NA	NA
**4.5 If data splitting was done to create training and test datasets, was there evidence that data leakage was avoided?***	**NA**	PN	**NA**
**4.6 If resampling methods were used to evaluate model performance, were all<br>model development steps replicated in the resampling process?***	**NA**	NA	**NA**
**4.7 Was the predictive performance of the model evaluated appropriately, e.g., calibration, discrimination, and net benefit?**	PY	PY	
**Risk of bias introduced by the analysis**	**RISK OF BIAS:**<br>*low/**high**/unclear*		
Rationale of risk of bias rating:<br>No external validation.<br>No null value handling analysis<br>Balanced dataset, prediction and outcome timeline overlap			

Step 4 - Assess the overall concerns regarding quality, risk of bias and applicability of the prediction model

**OVERALL JUDGEMENT OF PREDICTION MODEL**	
**Overall judgement of quality (development)**	**QUALITY CONCERN:**<br>*low/**high**/unclear*
Domain 3 and 4 high quality concerns	
**Overall judgement of risk of bias (evaluation)**	**RISK OF BIAS:**<br>*low/**high**/unclear*
Domain 3 and 4 high risk of bias	
**Overall judgement of applicability (development)**	**APPLICABILITY CONCERN:**<br>*low/**high**/unclear*
Domain 3 high applicability concerns	
**Overall judgement of applicability (evaluation)**	**APPLICABILITY CONCERN:**<br>*low/**high**/unclear*
Predictor-outcome overlap limits applicability, generalizability other health systems not assessed.	

Ilyas et al. (23)

Step 1 - PICOTS

**Population**	Adults with confirmed or suspected chronic kidney disease (CKD), UCI dataset
**Index model(s)**	J48 DT
**Comparator model(s)**	RF
**Outcome(s)**	CKD stages
**Timing**	Cross-sectional
**Setting and intended use of the prediction model**	UCI dataset, automated CKD staging decision support system

Step 2 – Classify the type of prediction model assessment

**Classify the assessment based on its aim**			
Type of prediction study	***Explanation***	***PROBAST+AI boxes to complete***	***Tick as appropriate***
**Development only**	**Prediction model development only, i.e., without evaluation of its performance.**	**Model development**	
**Evaluation only**	**External validation of one or more existing models in new data**	**Model evaluation**	
**Combination**	**Prediction model development combined in the same study (publication) with the evaluation of its apparent performance, internal validation performance, or external validation performance.**	**Model development and evaluation**	**X**

Step 3 - Assess quality and applicability or risk of bias and applicability

**Publication reference**	Iyas H, Ali S, Ponum M, Hasan O, Mahmood MT, Iftikhar M, et al. Chronic kidney disease diagnosis using decision tree algorithms. BMC Nephrol. 2021;22(1):273.
**Models of interest**	J48 DT and RF
**Outcome of interest**	Multiclass CKD severity stage prediciton

Model development

**DOMAIN 1: Participants and data sources**
**A. Quality**

UCI CKD dataset (n=400, 250 CKD, 150 not-CKD), 24 prediction features. No clinical setting, country, hospital, or time period described. No description of how the records were collected	
	**Y/ PY/ PN/ N/ NI**
**1.1 Were appropriate data sources used?**	Y
**1.2 Was an appropriate study design used?**	PY
**1.3 Did the in- and exclusions of study participants result in a representative dataset?**	PY
**Concern regarding quality of selection of participants and data sources**	**QUALITYCONCERN:** *low/**high**/unclear*
Rationale of quality rating: widely used dataset, no study design, no data collection description, no patient criteria, missing data handling not described	
**B. Applicability**	
No dates, clinical centre, country, or setting described.	
**Concern that the (data of the) included participants do not match the review question or the assessor's intended use of the prediction model**	**APPLICABILITY CONCERN:** *low/**high**/unclear*
Rationale of applicability rating: : unspecified clinical population, unclear specific clinical setting, low applicability to general populatioin	

**DOMAIN 2: Predictors**	
**A. Quality**	
24 UCI CKD attributes used as predictors . GFR additionally derived from 4 attributes (age, sex, race, serum creatinine) via CKD-EPI equation to generate stage labels. No pre-processing of predictors described	
	**Y/ PY/ PN/ N/ NI**
**2.1 Were predictors defined and assessed in a similar way for all participants?**	PY
**2.2 Was any pre-processing of predictors similar for all participants?**	NI
**2.3 Were predictor assessments made without knowledge of outcome data?**	NI
**2.4 Were the predictors included in the model available at the time the model was intended to be used?**	PY
**Concern regarding quality of selection of participants and data sources**	**QUALITYCONCERN:** *low/**high**/unclear*
Rationale of quality rating: *attributes used uniformly across all 400 instances, no description of pre-processing, dataset is pre-labelled, attributes are standard clinical/laboratory variables routinely available.*	
**B. Applicability**	
**Concern that the definition, pre-processing, assessment, or timing of assessment of the predictors in the model do not match the review question or the assessor's intended use**	**APPLICABILITY CONCERN:** *low/**high**/unclear*
Rationale of applicability rating: *predictors are standard CKD variable, most are routinely available, no pre-processing*	

**DOMAIN 3: Outcome**

A. Quality	
Multiclass outcome: six CKD stages, The outcome is not an independent clinical measurement but a deterministic calculation from predictor variables already in the dataset.	
	**Y/ PY/ PN/ N/ NI**
**3.1 Were outcomes defined and assessed appropriately?**	PY
**3.2 Were outcomes defined and assessed in a similar way for all participants?**	PY
**3.3 Were outcome assessments made without use or knowledge of predictor data?**	N
**3.4 Was the time interval between predictor assessment and outcome assessment appropriate?**	NI
**Concern regarding quality of the outcome or its determination**	**QUALITYCONCERN:** *low/**high**/unclear*
Rationale of quality rating *Same CKD-EPI formula applied to all 400 instances uniformly. 3.3 N: Critical concern — the outcome is calculated directly from predictor variables already in the dataset). variables are also among the 24 ML predictors, the ML model is trained to predict a stage that is itself a deterministic function of some of its input features. Overall: High quality concern.*	
**B. Applicability**	
GFR-derived stages are calculated at time of dataset assessment, single eGFR-based multiclass stage label.	
**Concern that the outcome, its definition, assessment, or timing of assessment do not match the review question or the assessor's intended use**	**APPLICABILITY CONCERN:** *low/**high**/unclear*
Rationale of applicability rating: *GFR outcome is clinically meaningful, but the model's applicability is severely limited because the outcome is a function of serum creatinine, age, sex, and race, GFR would simply be calculated directly, an ML model adds no value*	

DOMAIN 4: Analysis	
**A. Quality**	
n=400 (250 CKD, 150 non-CKD from UCI), 24 candidate predictors; multiclass outcome), class imbalance is severe, 15-fold cross-validation used	
J48 (C4.5 decision tree) and Random Forest, GFR calculated via CKD-EPI equation from 4 attributes to derive stage labels prior to ML training, 15-fold cross-validation for model evaluation, no feature selection applied prior to modelling	
Describe the performance measures of the prediction model:<br>*Overall accuracy, sensitivity, specificity, precision, recall, F-measure, and ROC area reported per stage*	
Describe missing data on predictors and outcomes as well as methods used for handling these missing data:<br>*Not reported*	
	**Y/ PY/ PN/ N/ NI**
**4.1 Was there evidence that the sample size was reasonable?**	PN
**4.2 Were continuous and categorical predictors handled appropriately?**	NI
**4.3 Were participants with missing or censored data handled appropriately in the analysis?**	N
**4.4 If methods to address class imbalance were used, was the model or the model predictions recalibrated?***	NI

**4.5 Were methods used to address potential model overfitting?**	PN
**Concern regarding quality of the analysis**	**QUALITYCONCERN:** *low/**high**/unclear*
Rationale of quality rating: *n=400 very small for a 6-class problem, class imbalance is severe and unaddressed, dataset has known missing values, no imputation or exclusion strategy reported, fundamental methodological flaw: the outcome is derived from predictor variables already in the dataset, no external validation.*	

Model evaluation

Domain 1,2 and 3 same analysis as model development

**DOMAIN 4: Analysis**			
**Risk of bias**			
Describe numbers of participants, number of predictors, outcome events and events per predictor:<br>*n=400 total, 15-fold CV, 6-class outcome, events per class very low for minority stages.*			
Describe the performance measures of the evaluated model:<br>*accuracy, sensitivity, specificity, precision, recall, F-measure, AUROC*			
Describe any participants who were excluded from the analysis:<br>*No exclusions reported*			
Describe missing data on predictors and outcomes as well as methods used for handling these missing data:<br>*Missing values not reported*			
	**Y/ PY/ PN/ N/ NI/ NA**		
**4.1 Was model evaluation based on only apparent performance avoided?**	PN		
	**A**	**I**	**E**
**4.2 Was there evidence that the sample size was reasonable?**	PN	PN	NA
**4.3 Were participants with missing or censored data handled appropriately in the analysis?**	N	N	NA
**4.4 If methods to address class imbalance were used, was the evaluation done in a dataset without imbalance correction?***	N	N	NA
**4.5 If data splitting was done to create training and test datasets, was there evidence that data leakage was avoided?***	**NA**	N	**NA**
**4.6 If resampling methods were used to evaluate model performance, were all model development steps replicated in the resampling process?***	**NA**	NA	**NA**
**4.7 Was the predictive performance of the model evaluated appropriately, e.g., calibration, discrimination, and net benefit?**	PN	PN	
**Risk of bias introduced by the analysis**	**RISK OF BIAS:** *low/**high**/unclear*		
Rationale of risk of bias rating:<br>15-fold CV used but fundamental circularity undermines performance estimates; n=400 is small for 6-class problem, missing values in UCI CKD dataset not acknowledged, severe class imbalance, outcome is calculated deterministically from serum creatinine, age, sex, and race, which are all included as ML predictors, model predicts transformation of its own features			

Step 4 - Assess the overall concerns regarding quality, risk of bias and applicability of the prediction model

**OVERALL JUDGEMENT OF PREDICTION MODEL**

Overall judgement of quality (development)	QUALITY CONCERN: *low/**high**/unclear*
All four domains high quality concerns	
**Overall judgement of risk of bias (evaluation)**	**RISK OF BIAS:** *low/**high**/unclear*
All four domains high risk of bias	
**Overall judgement of applicability (development)**	**APPLICABILITY CONCERN:** *low/**high**/unclear*
All four domains applicability concerns	
**Overall judgement of applicability (evaluation)**	**APPLICABILITY CONCERN:** *low/**high**/unclear*
Evaluation performed via 15-fold CV only, no external validation, circular outcome definition	

Koch Nogueira et al. (24)

Step 1 - PICOTS

**Population**	Children with confirmed CKD and controles
**Index model(s)**	XGBoost
**Comparator model(s)**	DT
**Outcome(s)**	CKD vs no-CKD
**Timing**	Cross-sectional
**Setting and intended use of the prediction model**	UCI dataset, automated CKD staging decision support system

Step 2 – Classify the type of prediction model assessment

Classify the assessment based on its aim			
Type of prediction study	***Explanation***	***PROBAST+AI boxes to complete***	***Tick as appropriate***
**Development only**	**Prediction model development only, i.e., without evaluation of its performance.**	**Model development**	
**Evaluation only**	**External validation of one or more existing models in new data**	**Model evaluation**	
**Combination**	**Prediction model development combined in the same study (publication) with the evaluation of its apparent performance, internal validation performance, or**	**Model development and evaluation**	**X**

	**external validation performance.**		

Step 3 - Assess quality and applicability or risk of bias and applicability

**Publication reference**	Koch Nogueira PC, Venson AH, de Carvalho MFC, Konstantyner T, Sesso R. Symptoms for early diagnosis of chronic kidney disease in children — a machine learning–based score. Eur J Pediatr. 2023;182(8):3631–7.
**Models of interest**	XGBoost and DT
**Outcome of interest**	CKD or no CKD

Model development

**DOMAIN 1: Participants and data sources**	
**A. Quality**	
children with CKD (n=376), controls: age-matched (within 2 years) and sex-matched healthy children cared for at same institutions for acute illnesses not involving kidney (n=376 after excluding 38). 8 centres across Brazil, study period 2015-2019, exclusions clearly reported	
	**Y/ PY/ PN/ N/ NI**
**1.1 Were appropriate data sources used?**	Y
**1.2 Was an appropriate study design used?**	PY
**1.3 Did the in- and exclusions of study participants result in a representative dataset?**	PY
**Concern regarding quality of selection of participants and data sources**	**QUALITYCONCERN:** ***low****/high/unclear*
Rationale of quality rating: case-control study with real clinical data, clear case definition, controls age- and sex-matched; recruited from same institutions, inclusion and exclusion criteria clearly described for both cases and controls	
**B. Applicability**	
8 paediatric nephrology centres in Brazil, 376 CKD cases, 376 controls, data collection 2015-2019.	
**Concern that the (data of the) included participants do not match the review question or the assessor's intended use of the prediction model**	**APPLICABILITY CONCERN:** ***low****/high/unclear*
Rationale of applicability rating: well-described, multi-regional Brazilian paediatric CKD cohort, primary use is primary care in Brazil, applicability to other healthcare systems requires further validation	

**DOMAIN 2: Predictors**
**A. Quality**

30 pre-selected symptom/sign variables from a structured questionnaire of over 100 items, pre-selection was based on statistical association with outcome (Fisher exact test or chi-squared test) and clinical plausibility judged by author consensus	
	**Y/ PY/ PN/ N/ NI**
**2.1 Were predictors defined and assessed in a similar way for all participants?**	PY
**2.2 Was any pre-processing of predictors similar for all participants?**	PY
**2.3 Were predictor assessments made without knowledge of outcome data?**	PY
**2.4 Were the predictors included in the model available at the time the model was intended to be used?**	PY
**Concern regarding quality of selection of participants and data sources**	**QUALITYCONCERN:** ***low***/*high*/*unclear*
30 questionnaire variables applied uniformly to all 752 participants, same trained researchers administered questionnaire, questionnaire was completed at time of interview, caregivers answered based on history, with some recall bias acknowledged, 30 predictors are clinical symptoms/signs obtainable without laboratory tests at primary care level, the intended setting.	
**B. Applicability**	
**Concern that the definition, pre-processing, assessment, or timing of assessment of the predictors in the model do not match the review question or the assessor's intended use**	**APPLICABILITY CONCERN:** ***low***/*high*/*unclear*
Rationale of applicability rating: *predictors are standard CKD variable, most are routinely available, no pre-processing*	

**DOMAIN 3: Outcome**	
**A. Quality**	
CKD diagnosis no CKD, CKD defined as GFR below 90 ml/min/1.73m2 estimated by the Schwartz equation, confirmed at a paediatric nephrology centre with at least 3 months follow-up, outcome determined by the nephrology centre at time of enrolment, caregivers answered the questionnaire retrospectively about symptoms experienced prior to or around time of diagnosis	
	**Y/ PY/ PN/ N/ NI**
**3.1 Were outcomes defined and assessed appropriately?**	PY
**3.2 Were outcomes defined and assessed in a similar way for all participants?**	PY
**3.3 Were outcome assessments made without use or knowledge of predictor data?**	PY
**3.4 Was the time interval between predictor assessment and outcome assessment appropriate?**	PN
**Concern regarding quality of the outcome or its determination**	**QUALITYCONCERN:** *low/*___high___*/unclear*
same definition applied to all cases, CKD diagnosis determined by nephrologists independently, the outcome pre-dates the predictor data, introduces substantial recall bias: caregivers of CKD children may over-report symptoms compared to controls. Authors acknowledge this limitation. Controls were also retrospectively interviewed for historical symptoms.	
**B. Applicability**	
CKD diagnosis confirmed at nephrology centre prior to questionnaire.	
**Concern that the outcome, its definition, assessment, or timing of assessment do not match the review question or the assessor's intended use**	**APPLICABILITY CONCERN:** *low/*___high___*/unclear*

Rationale of applicability rating: *The intended use is prospective however, predictors were collected retrospectively in this study, limits the applicability*

**DOMAIN 4: Analysis**	
**A. Quality**	
n=752 total (376 CKD cases, 376 controls), 30 candidate predictors, 75/25 training/testing	
Decision tree and XGBoost, pre-selection of 30 from over 100 variables via Fisher exact/chi-squared test + clinical plausibility consensus (performed before train/evaluation split — potential optimism).	
Describe the performance measures of the prediction model:<br>*Sensitivity, specificity, PPV, NPV, positive and negative likelihood ratios, balanced accuracy, and AUC/ROC reported with 95%CI*	
Describe missing data on predictors and outcomes as well as methods used for handling these missing data:<br>*No formal missing data analysis reported. Effect on model performance not specifically assessed.*	
	**Y/ PY/ PN/ N/ NI**
**4.1 Was there evidence that the sample size was reasonable?**	PY
**4.2 Were continuous and categorical predictors handled appropriately?**	PY
**4.3 Were participants with missing or censored data handled appropriately in the analysis?**	PY
**4.4 If methods to address class imbalance were used, was the model or the model predictions recalibrated?***	Y
**4.5 Were methods used to address potential model overfitting?**	PY
**Concern regarding quality of the analysis**	**QUALITYCONCERN:**<br>*low/**high**/unclear*
Rationale of quality rating: *'Do not know' responses retained as a separate category, 1:1 case-control matching avoids class imbalance, pre-selection of 30 variables from over 100 was performed before the train/evaluation split, no external validation, recall bias in predictors*	

Model evaluation

Domain 1,2 and 3 same analysis as model development

**DOMAIN 4: Analysis**	
**Risk of bias**	
Evaluation subsample: n=188 (94 CKD, 94 controls); 30 predictors; 94 outcome events	
Describe the performance measures of the evaluated model:<br>*Sensitivity, specificity, PPV, NPV, balanced accuracy, positive and negative likelihood ratios reported, 95%CIs provided for AUC.*	
Describe any participants who were excluded from the analysis:<br>*21 cases and 38 controls excluded before analysis, exclusion criteria described*	
Describe missing data on predictors and outcomes as well as methods used for handling these missing data:<br>*'Do not know' responses coded as dummy variable*	
	**Y/ PY/ PN/ N/ NI/ NA**
**4.1 Was model evaluation based on only apparent performance avoided?**	PY

	A	I	E
**4.2 Was there evidence that the sample size was reasonable?**	PY	PY	NA
**4.3 Were participants with missing or censored data handled appropriately in the analysis?**	PY	PY	NA
**4.4 If methods to address class imbalance were used, was the evaluation done in<br>a dataset without imbalance correction?***	Y	Y	NA
**4.5 If data splitting was done to create training and test datasets, was there evidence that data leakage was avoided?***	**NA**	PY	**NA**
**4.6 If resampling methods were used to evaluate model performance, were all<br>model development steps replicated in the resampling process?***	**NA**	NA	**NA**
**4.7 Was the predictive performance of the model evaluated appropriately, e.g., calibration, discrimination, and net benefit?**	PY	PY	
**Risk of bias introduced by the analysis**	**RISK OF BIAS:**<br>*low/**high**/unclear*		
Rationale of risk of bias rating:<br>balanced 1:1 design, Train/evaluation split performed, pre-selection of 30 from over 100 variables was done before split, comprehensive discrimination metrics, primary concern is retrospective predictor collection.			

Step 4 - Assess the overall concerns regarding quality, risk of bias and applicability of the prediction model

OVERALL JUDGEMENT OF PREDICTION MODEL	
**Overall judgement of quality (development)**	**QUALITY CONCERN:**<br>*low/**high**/unclear*
Domain 3 and 4 high quality concerns	
**Overall judgement of risk of bias (evaluation)**	**RISK OF BIAS:**<br>*low/**high**/unclear*
Domain 3 and 4 high risk of bias	
**Overall judgement of applicability (development)**	**APPLICABILITY CONCERN:**<br>*low/**high**/unclear*
Domain 3 and 4 high applicability concern	
**Overall judgement of applicability (evaluation)**	**APPLICABILITY CONCERN:**<br>*low/**high**/unclear*
No external validation, retrospective predictor recall issue in Domain 3 applies also to evaluation	

Lapi et al. (25)

Step 1 - PICOTS

**Population**	Italian population (14< years old), no prior
**Index model(s)**	GA2M
**Comparator model(s)**	Light GBM, XGBoost GBM, CatBoost GBM, GAM, Logistic regression, Random Forest

**Outcome(s)**	Incident CKD: defined as a ICD-9-CM diagnosis of CKD, and/or GFR below 60 ml/min per 1.73m2 during follow-up.
**Timing**	Prospective cohort. Follow-up until: first CKD diagnosis, death, end of registration with GP, or December 31, 2020.(2018-2020).
**Setting and intended use of the prediction model**	Italian general practice (primary care). HSD database, Italy, 2018-2020.

Step 2 – Classify the type of prediction model assessment

**Classify the assessment based on its aim**			
Type of prediction study	***Explanation***	***PROBAST+AI boxes to complete***	***Tick as appropriate***
**Development only**	**Prediction model development only, i.e., without evaluation of its performance.**	**Model development**	
**Evaluation only**	**External validation of one or more existing models in new data**	**Model evaluation**	
**Combination**	**Prediction model development combined in the same study (publication) with the evaluation of its apparent performance, internal validation performance, or external validation performance.**	**Model development and evaluation**	**X**

Step 3 - Assess quality and applicability or risk of bias and applicability

**Publication reference**	Lapi F, Nuti L, Marconi E, Medea G, Cricelli I, Papi M, et al. To predict the risk of chronic kidney disease (CKD) using Generalized Additive2 Models (GA2M). J Am Med Inform Assoc. 2023;30(9):1494–502.
**Models of interest**	GA2M (Explainable Boosting Machine), comparators: Light GBM, XGBoost, CatBoost, GAM, Logistic regression, Random Forest
**Outcome of interest**	Incident CKD (ICD-9-CM diagnosis or GFR below 60 ml/min/1.73m2) during 2018-2020 follow-up

Model development

**DOMAIN 1: Participants and data sources**
**A. Quality**

Health Search Database (HSD): longitudinal electronic health records. All patients aged 15-95 years, active in HSD between January 1, 2018 and December 31, 2020, no prior CKD diagnosis, at least 1 year look-back, at least 6 months follow-up, n=997,864 eligible, 30,705 incident CKD cases during follow-up. Cohort randomly split 4:1 into training and testing	
	**Y/ PY/ PN/ N/ NI**
**1.1 Were appropriate data sources used?**	Y
**1.2 Was an appropriate study design used?**	Y
**1.3 Did the in- and exclusions of study participants result in a representative dataset?**	Y
**Concern regarding quality of selection of participants and data sources**	**QUALITYCONCERN:** ***low***/*high/unclear*
Rationale of quality rating: 1.1 Y: HSD is a validated, nationally representative Italian primary care database used in multiple previous prediction studies; diagnostic and prescription coding standardised (ICD-9-CM, ATC). cohort design is appropriate study design for incident CKD prediction, eligibility criteria clearly defined, large sample with clear eligibility flowchart, exclusion of patients with less than 6 months follow-up clearly described, cohort is representative of adults in Italian general practice. Overall: Low quality concern.	
**B. Applicability**	
HSD database, Italy, 2018-2020, n=997,864 adults in Italian general practice, 30,705 incident CKD cases; mean age 53 years,	
**Concern that the (data of the) included participants do not match the review question or the assessor's intended use of the prediction model**	**APPLICABILITY CONCERN:** ***low***/*high/unclear*
Rationale of applicability rating:; demographic and clinical characteristics well described, explicitly designed for Italian primary care, GP-registered adults only, relatively short time window.	

**DOMAIN 2: Predictors**	
**A. Quality**	
20 candidate determinants selected from previous HSD-based CKD work, current medical literature, and clinical rationale. Missing values for creatinine, BMI, and blood pressure imputed by MICE (Multiple Imputation by Chained Equations). All predictors operationally defined at or before study entry date.	
	**Y/ PY/ PN/ N/ NI**
**2.1 Were predictors defined and assessed in a similar way for all participants?**	Y
**2.2 Was any pre-processing of predictors similar for all participants?**	Y
**2.3 Were predictor assessments made without knowledge of outcome data?**	Y
**2.4 Were the predictors included in the model available at the time the model was intended to be used?**	Y
**Concern regarding quality of selection of participants and data sources**	**QUALITYCONCERN:** ***low***/*high/unclear*
2.1 Y: All 20 predictors applied uniformly to all eligible participants, MICE imputation for missing continuous variables %) described and justified, weighting applied uniformly in, outcome occurs after entry, all 20 predictors are routinely available in GP records	
**B. Applicability**	
**Concern that the definition, pre-processing, assessment, or timing of assessment of the predictors in the model do not match the review question or the assessor's intended use**	**APPLICABILITY CONCERN:** ***low***/*high/unclear*

Rationale of applicability rating: *All 20 predictors are standard variables fully available in GP software.*

**DOMAIN 3: Outcome**	
**A. Quality**	
Incident CKD: operationally defined as ICD-9-CM/free-text CKD diagnosis, validated by expert clinician, and/or GFR below 60 ml/min per 1.73m2 recorded during follow-up. Time interval: from study entry date to first CKD event or censoring.	
	**Y/ PY/ PN/ N/ NI**
**3.1 Were outcomes defined and assessed appropriately?**	Y
**3.2 Were outcomes defined and assessed in a similar way for all participants?**	Y
**3.3 Were outcome assessments made without use or knowledge of predictor data?**	Y
**3.4 Was the time interval between predictor assessment and outcome assessment appropriate?**	Y
**Concern regarding quality of the outcome or its determination**	**QUALITYCONCERN:** ***low****/high/unclear*
Composite outcome combines ICD-9-CM/free-text coding with GFR criterion (below 60 ml/min/1.73m2); free-text, prospective cohort, CKD outcome occurs after study entry date, follow-up from study entry to first event, death, deregistration, or end of study, minimum 6-month follow-up required	
**B. Applicability**	
Incident CKD during 2018-2020 follow-up; 30,705 events, composite outcome, relative frequency: GFR-based vs diagnosis-based split not reported separately.	
**Concern that the outcome, its definition, assessment, or timing of assessment do not match the review question or the assessor's intended use**	**APPLICABILITY CONCERN:** ***low****/high/unclear*
CKD defined by GFR below 60 ml/min/1.73m2 corresponds to KDIGO G3a+ (moderate-severe CKD), early stages (G1-G2) are not captured, predicts moderate-to-severe incident CKD rather than all-stage early CKD. appropriate for the Italian primary care context but limits applicability to early-stage detection	

**DOMAIN 4: Analysis**	
**A. Quality**	
n=997,864 eligible, 30,705 incident CKD cases, 20 candidate predictors, training/testing 4/1, class imbalance, addressed by case weighting	
GA2M (EBM), Light GBM, XGBoost GBM, CatBoost GBM, GAM, Logistic regression, Random Forest.. Case weighting for class imbalance. MICE imputation for missing continuous values.	
AUC (95%CI) and Average Precision (AP, 95%CI) reported for all 7 models, at 80% sensitivity threshold: accuracy, specificity, precision, F1 score, Youden J index with 95%CI reported	
Describe missing data on predictors and outcomes as well as methods used for handling these missing data: *Missing values imputed by MICE assuming MAR.*	
	**Y/ PY/ PN/ N/ NI**
**4.1 Was there evidence that the sample size was reasonable?**	Y
**4.2 Were continuous and categorical predictors handled appropriately?**	Y

**4.3 Were participants with missing or censored data handled appropriately in the analysis?**	Y
**4.4 If methods to address class imbalance were used, was the model or the model predictions recalibrated?***	Y
**4.5 Were methods used to address potential model overfitting?**	Y
**Concern regarding quality of the analysis**	**QUALITYCONCERN:** ***low***/*high*/*unclear*
n=997,864 is very large, MICE imputation with MAR assumption, described and justified, class imbalance explicitly addressed by case weighting in training, no external validation on independent dataset; no calibration plots; funding from AstraZeneca Italy	

Model evaluation

Domain 1,2 and 3 same analysis as model development

**DOMAIN 4: Analysis**			
**Risk of bias**			
Test set: approx. n=200,000; 20 predictors; approximately 7,700 incident CKD events. Training/testing 4/1			
AUC (95%CI) and AP (95%CI) reported for all 7 models, at 80% sensitivity: accuracy, specificity, precision, F1, Youden J with 95%.			
Describe any participants who were excluded from the analysis: *Patients with less than 6 months follow-up excluded at study entry.*			
Describe missing data on predictors and outcomes as well as methods used for handling these missing data: *Missing values imputed by MICE in training set*			
	**Y/ PY/ PN/ N/ NI/ NA**		
**4.1 Was model evaluation based on only apparent performance avoided?**	Y		
	**A**	**I**	**E**
**4.2 Was there evidence that the sample size was reasonable?**	Y	Y	NA
**4.3 Were participants with missing or censored data handled appropriately in the analysis?**	Y	Y	NA
**4.4 If methods to address class imbalance were used, was the evaluation done in a dataset without imbalance correction?***	Y	Y	NA
**4.5 If data splitting was done to create training and test datasets, was there evidence that data leakage was avoided?***	**NA**	Y	**NA**
**4.6 If resampling methods were used to evaluate model performance, were all model development steps replicated in the resampling process?***	**NA**	NA	**NA**
**4.7 Was the predictive performance of the model evaluated appropriately, e.g., calibration, discrimination, and net benefit?**	PY	PY	
**Risk of bias introduced by the analysis**	**RISK OF BIAS:** ***low***/*high*/*unclear*		
no evaluation on training data only, large test set, MICE imputation applied consistently to test set, natural CKD prevalence in test set, case weighting used in training mitigates the impact of imbalance, no evidence of data leakage, potential conflict of interest: study funded by AstraZeneca Italy			

Step 4 - Assess the overall concerns regarding quality, risk of bias and applicability of the prediction model

OVERALL JUDGEMENT OF PREDICTION MODEL	
**Overall judgement of quality (development)**	**QUALITY CONCERN:** ***low****/high/unclear*
All domains low quality concerns	
**Overall judgement of risk of bias (evaluation)**	**RISK OF BIAS:** ***low****/high/unclear*
All domains low risk of bias	
**Overall judgement of applicability (development)**	**APPLICABILITY CONCERN:** ***low****/high/unclear*
Low applicability concerns	
**Overall judgement of applicability (evaluation)**	**APPLICABILITY CONCERN:** ***low****/high/unclear*
Evaluation performed on a large test set, no external validation, prospective validation in independent Italian GP cohort or other country would be needed before wide deployment, low concern for the Italian primary care setting as intended.	

Yoshizaki et al. (26)

Step 1 - PICOTS

**Population**	Adults who underwent annual health examinations in Japan every year, between 2017 and 2022, with eGFR at least 60 ml/min/1.73m2 and negative proteinuria from 2017-2021, n=30,273 in final analysis (1,372 with incident CKD).
**Index model(s)**	Recurrent Neural Network (RNN), Neural Network (NN), Logistic Regression (LR), Conditional Logistic Regression (CLR for matched analysis).
**Comparator model(s)**	Compared against each other
**Outcome(s)**	Incident eGFR below 60ml/min/1.73m2, proteinuria, or both outcomes together
**Timing**	Prospective cohort. Inception cohort. Predictors: health examination data 2017-2021 (5-year longitudinal or single year).
**Setting and intended use of the prediction model**	Occupational health examination centres, Japan, 2017-2022, risk prediction tool using routine annual health examination data to identify individuals at risk of incident CKD

Step 2 – Classify the type of prediction model assessment

**Classify the assessment based on its aim**			
Type of prediction study	***Explanation***	***PROBAST+AI boxes to complete***	***Tick as appropriate***

**Development only**	**Prediction model development only, i.e., without evaluation of its performance.**	**Model development**	
**Evaluation only**	**External validation of one or more existing models in new data**	**Model evaluation**	
**Combination**	**Prediction model development combined in the same study (publication) with the evaluation of its apparent performance, internal validation performance, or external validation performance.**	**Model development and evaluation**	**X**

Step 3 - Assess quality and applicability or risk of bias and applicability

**Publication reference**	Yoshizaki Y, Kato K, Fujihara K, Sone H, Akazawa K. Development of a machine learning tool to predict the risk of incident chronic kidney disease using health examination data. Front Public Health. 2024;12:1495054.
**Models of interest**	RNN, Neural Network, Logistic Regression, and Conditional Logistic Regression for incident CKD prediction at 1 and 5 years
**Outcome of interest**	Three binary outcomes: incident eGFR below 60 ml/min/1.73m2; proteinuria; or eGFR below 60 ml/min/1.73m2 or proteinuria (incident CKD in 2022)

Model development

**DOMAIN 1: Participants and data sources**	
**A. Quality**	
Occupational health examination database, 2017-2022, excluded: n=9,748 with history of antihypertensive, antidiabetic, or anticholesterol drug use, final: n=30,273, outcome subgroups: eGFR below 60 (672 CKD / 29,601 non-CKD); proteinuria (743 / 29,530); combined (1,372 / 28,901), matched analysis: 1:1 age- and sex-matched non-CKD controls.	
	**Y/ PY/ PN/ N/ NI**
**1.1 Were appropriate data sources used?**	Y
**1.2 Was an appropriate study design used?**	Y
**1.3 Did the in- and exclusions of study participants result in a representative dataset?**	PY
**Concern regarding quality of selection of participants and data sources**	**QUALITYCONCERN:** ***low***/*high*/*unclear*
Rationale of quality rating: routine annual occupational health examination data, accurately recorded every year for 6 years; appropriate for incident CKD prediction, inception cohort with CKD-free baseline appropriate design, clear eligibility flowchart, exclusion of patients on antihypertensive/antidiabetic/anticholesterol drugs is justified but limits representativeness of the general at-risk population	
**B. Applicability**	

Niigata occupational health examination cohort, Japan, 2017-2022; n=30,273 working adults; CKD-free at baseline.

**Concern that the (data of the) included participants do not match the review question or the assessor's intended use of the prediction model**	**APPLICABILITY CONCERN:** ***low****/high/unclear*
Rationale of applicability rating: the cohort consists of working adults undergoing routine occupational health checks in one Japanese prefecture, well-suited to the intended use However, exclusion of patients already on certain medications removes a substantial high-risk group, and the occupational setting introduces a healthy-worker effect. Generalisability to the general population, non-working adults (elderly, unemployed), and non-Japanese populations is uncertain. Low applicability concern for the intended occupational health screening context.	

**DOMAIN 2: Predictors**	
**A. Quality**	
Predictors from annual health examinations: demographic, clinical, biochemical, questionnaire lifestyle, proteinuria NOT used as predictor, missing data imputed by random-forest multiple imputation. eGFR is a key predictor.	
	**Y/ PY/ PN/ N/ NI**
**2.1 Were predictors defined and assessed in a similar way for all participants?**	Y
**2.2 Was any pre-processing of predictors similar for all participants?**	Y
**2.3 Were predictor assessments made without knowledge of outcome data?**	Y
**2.4 Were the predictors included in the model available at the time the model was intended to be used?**	Y
**Concern regarding quality of selection of participants and data sources**	**QUALITYCONCERN:** ***low****/high/unclear*
All predictors applied uniformly to all 30,273 participants, no temporal overlap, all predictors obtainable from routine annual health examinations	
**B. Applicability**	
**Concern that the definition, pre-processing, assessment, or timing of assessment of the predictors in the model do not match the review question or the assessor's intended use**	**APPLICABILITY CONCERN:** ***low****/high/unclear*
Rationale of applicability rating: *All predictors obtainable from routine annual health examinations, matching intended use, eGFR variable (Japan-specific formula) is the strongest predictor, model performance depends heavily on it, low applicability concern within the Japanese occupational health context.*	

**DOMAIN 3: Outcome**	
**A. Quality**	
Three binary outcomes in 2022: incident eGFR below 60 ml/min/1.73m2, proteinuria; eGFR below 60 OR proteinuria, outcome ascertained at single 2022 examination. Persistence for at least 3 months not confirmed. Time interval: 1 year or 5 years to 2022 outcome.	
Or	**Y/ PY/ PN/ N/ NI**
**3.1 Were outcomes defined and assessed appropriately?**	PY
**3.2 Were outcomes defined and assessed in a similar way for all participants?**	Y
**3.3 Were outcome assessments made without use or knowledge of predictor data?**	PN

**3.4 Was the time interval between predictor assessment and outcome assessment appropriate?**	Y
**Concern regarding quality of the outcome or its determination**	**QUALITYCONCERN:** *low/**high**/unclear*
authors acknowledge that 3-month persistence could not be confirmed (single 2022 measurement), transient changes may be misclassified, same outcome definitions applied uniformly, the eGFR-based outcome derives from serum creatinine, and baseline eGFR/creatinine is also a key predictor, the models predict a future value of essentially the same variable; performance collapses when eGFR is excluded, model largely tracks creatinine correlation	
**B. Applicability**	
Incident CKD outcomes (eGFR below 60, proteinuria, or both) at single 2022 examination, 3-month persistence not confirmed, outcome variable (eGFR) is a future value of a baseline predictor variable.	
**Concern that the outcome, its definition, assessment, or timing of assessment do not match the review question or the assessor's intended use**	**APPLICABILITY CONCERN:** *low/**high**/unclear*
eGFR outcome uses Japan-specific formula, without confirming 3-month persistence, risking misclassification of transient changes, strong dependence on baseline	

**DOMAIN 4: Analysis**	
**A. Quality**	
n=30,273, about 25-30 candidate predictors, matched analysis, training/testing 4/1	
Four model types: RNN, NN, LR, CLR (matched). RNN/NN/LR for non-matched single-year and 5-year data; CLR substitutes LR in matched analysis. Random-forest multiple imputation.	
Accuracy, sensitivity, specificity, sensitivity analysis: with vs without eGFR	
Missing data imputed using random-forest multiple imputation. Impact not analyzed	
	**Y/ PY/ PN/ N/ NI**
**4.1 Was there evidence that the sample size was reasonable?**	PY
**4.2 Were continuous and categorical predictors handled appropriately?**	Y
**4.3 Were participants with missing or censored data handled appropriately in the analysis?**	PY
**4.4 If methods to address class imbalance were used, was the model or the model predictions recalibrated?***	PY
**4.5 Were methods used to address potential model overfitting?**	PY
**Concern regarding quality of the analysis**	**QUALITYCONCERN:** ***low**/high/unclear*
matched samples small and proteinuria/combined matched analyses have limited events, predictors handled appropriately, random forest imputation is reasonable but missingness extent and sensitivity not detailed, matched analysis addresses class imbalance, non-matched analysis is highly imbalanced and performance differs between matched and non-matched, 80/20 train/test split, confidence intervals largely absent, small matched samples increase overfitting risk, strong reliance on baseline inflates apparent performance and is not corrected analytically, no external validation	

Model evaluation

Domain 1,2 and 3 same analysis as model development

DOMAIN 4: Analysis			
**Risk of bias**			
Test sample: 20% holdout, matched analyses use small balanced samples			
AUC (95%CI) and AP (95%CI) reported for all 7 models, at 80% sensitivity: accuracy, specificity, precision, F1, Youden J with 95%.			
Describe any participants who were excluded from the analysis: Patients with less than 6 months follow-up excluded at study entry.			
Describe missing data on predictors and outcomes as well as methods used for handling these missing data: Missing values imputed by MICE in training set			
	**Y/ PY/ PN/ N/ NI/ NA**		
**4.1 Was model evaluation based on only apparent performance avoided?**	PY		
	**A**	**I**	**E**
**4.2 Was there evidence that the sample size was reasonable?**	PY	PY	NA
**4.3 Were participants with missing or censored data handled appropriately in the analysis?**	PY	PY	NA
**4.4 If methods to address class imbalance were used, was the evaluation done in a dataset without imbalance correction?***	PY	PY	NA
**4.5 If data splitting was done to create training and test datasets, was there evidence that data leakage was avoided?***	**NA**	PN	**NA**
**4.6 If resampling methods were used to evaluate model performance, were all model development steps replicated in the resampling process?***	**NA**	NA	**NA**
**4.7 Was the predictive performance of the model evaluated appropriately, e.g., calibration, discrimination, and net benefit?**	PN	PN	
**Risk of bias introduced by the analysis**	**RISK OF BIAS:** *low/**high**/unclear*		
20% test sample, RF imputation applied consistently; detail limited, 1:1 matched analysis addresses imbalance; non-matched analysis highly imbalanced and authors note performance varies, eGFR-based outcome is a future value of the baseline eGFR predictor, dramatic AUROC drop (to 0.498-0.732) when eGFR is excluded demonstrates that high performance (AUROC above 0.9 for 1-year eGFR outcome) largely reflects creatinine correlation rather than multifactorial risk prediction, structural leakage-like issue			

Step 4 - Assess the overall concerns regarding quality, risk of bias and applicability of the prediction model

OVERALL JUDGEMENT OF PREDICTION MODEL	
**Overall judgement of quality (development)**	**QUALITY CONCERN:** *low/**high**/unclear*
Domains 1 and 2 low quality concern, inception cohort design, annual health examination data. High Domain 3 (outcome): the eGFR outcome is a future value of the baseline eGFR predictor, the outcome relied on a single measurement without confirming 3-month persistence, and proteinuria prediction was near chance level..	
**Overall judgement of risk of bias (evaluation)**	**RISK OF BIAS:** *low/**high**/unclear*
High risk of bias Domains 3 and 4, central issue is that the eGFR-based CKD outcome is essentially a future measurement of the same variable used as the dominant predictor, sensitivity analysis shows removing eGFR collapses AUROC, demonstrating the headline performance largely reflects creatinine	

autocorrelation rather than genuine multifactorial CKD prediction, measurement outcome without 3-month persistence confirmation, no external validation; poor proteinuria prediction, transparently reported

**Overall judgement of applicability (development)**	**APPLICABILITY CONCERN:** *low/**high**/unclear*
Domains 1 and 2 low applicability concern domain 3 raises high applicability concern: the eGFR outcome uses a Japan-specific formula, relies on a single unconfirmed measurement cohort exclusions (patients on cardiometabolic medications) and the occupational healthy-worker effect further limit applicability to the general at-risk population.	
**Overall judgement of applicability (evaluation)**	**APPLICABILITY CONCERN:** *low/**high**/unclear*
Evaluation on a 20% test sample from the same Japanese occupational cohort, no external validation, reported performance does not reflect prospective multifactorial CKD risk prediction, cannot be assumed to transfer to other populations or to a clinically meaningful CKD definition.	

Zhang et al. (27)

Step 1 - PICOTS

**Population**	Adults with type 2 diabetes mellitus and CKD/renal lesions in whom differentiation of DN from NDRD is clinically required and who are candidates for kidney biopsy, single tertiary nephrology referral centre in China.
**Index model(s)**	SVM and RF
**Comparator model(s)**	Two earlier LR models (model-2008 and model 2014)
**Outcome(s)**	Histopathological diagnosis of DN vs NDRD on renal biopsy
**Timing**	Cross-sectional
**Setting and intended use of the prediction model**	Tertiary nephrology referral setting. Intended as a noninvasive differention of DN from NDRD

Step 2 – Classify the type of prediction model assessment

**Classify the assessment based on its aim**			
Type of prediction study	***Explanation***	***PROBAST+AI boxes to complete***	***Tick as appropriate***
**Development only**	**Prediction model development only, i.e., without evaluation of its performance.**	**Model development**	
**Evaluation only**	**External validation of one or more existing models in new data**	**Model evaluation**	

**Combination**	**Prediction model development combined in the same study (publication) with the evaluation of its apparent performance, internal validation performance, or external validation performance.**	**Model development and evaluation**	**X**

Step 3 - Assess quality and applicability or risk of bias and applicability

**Publication reference**	Zhang WG, Liu XM, Dong ZY, Wang Q, Pei ZY, Chen YZ, et al. New Diagnostic Model for the Differentiation of Diabetic Nephropathy From Non-Diabetic Nephropathy in Chinese Patients. Front Endocrinol (Lausanne). 2022;13.
**Models of interest**	RF and SVM 10-variable diagnostic
**Outcome of interest**	Biopsy-proven DN vs NDRD.

Model development

**DOMAIN 1: Participants and data sources**	
**A. Quality**	
retrospective, single-centre cohort of 1,030 consecutive T2DM patients who underwent kidney biopsy (Jan 2005–Mar 2017), after exclusions 929 patients analysed (DN 329, NDRD 600).	
	**Y/ PY/ PN/ N/ NI**
**1.1 Were appropriate data sources used?**	PY
**1.2 Was an appropriate study design used?**	PY
**1.3 Did the in- and exclusions of study participants result in a representative dataset?**	PN
**Concern regarding quality of selection of participants and data sources**	**QUALITYCONCERN:** *low/**high**/unclear*
Rationale of quality rating: cohort consists exclusively of biopsied patients from one tertiary referral centre, creating strong selection/spectrum bias: NDRD prevalence is high because biopsy is preferentially performed when NDRD is suspected, mixed DN+NDRD cases were excluded from development, not reflecting real practice and removing a clinically important group the tool would encounter.	
**B. Applicability**	
biopsied T2DM patients, single tertiary nephrology centre in China, 2005–2017.	
**Concern that the (data of the) included participants do not match the review question or the assessor's intended use of the prediction model**	**APPLICABILITY CONCERN:** *low/**high**/unclear*
Rationale of applicability rating: model intended for the broad population of T2DM-CKD patients being considered for biopsy, but the development is a highly selected, authors themselves caution that the findings may not transfer to other countries.	

**DOMAIN 2: Predictors**

A. Quality	
10 final predictors, all routine clinical/laboratory variables collected at baseline before biopsy.	
	**Y/ PY/ PN/ N/ NI**
**2.1 Were predictors defined and assessed in a similar way for all participants?**	PY
**2.2 Was any pre-processing of predictors similar for all participants?**	PY
**2.3 Were predictor assessments made without knowledge of outcome data?**	Y
**2.4 Were the predictors included in the model available at the time the model was intended to be used?**	Y
**Concern regarding quality of selection of participants and data sources**	**QUALITYCONCERN:** ***low***/*high/unclear*
Predictors are routine variables collected before the biopsy, uniformly pre-processed some items carry mild subjectivity	
**B. Applicability**	
**Concern that the definition, pre-processing, assessment, or timing of assessment of the predictors in the model do not match the review question or the assessor's intended use**	**APPLICABILITY CONCERN:** ***low***/*high/unclear*
Rationale of applicability rating: Predictors are inexpensive, routinely available clinical and laboratory measures, well matched to noninvasive use.	

DOMAIN 3: Outcome	
**A. Quality**	
DN vs NDRD on renal biopsy, DN diagnosed on established histological criteria, NDRD on classical criteria, biopsies read independently by two pathologists, with discordant results resolved by discussion.	
Or	**Y/ PY/ PN/ N/ NI**
**3.1 Were outcomes defined and assessed appropriately?**	Y
**3.2 Were outcomes defined and assessed in a similar way for all participants?**	Y
**3.3 Were outcome assessments made without use or knowledge of predictor data?**	NI
**3.4 Was the time interval between predictor assessment and outcome assessment appropriate?**	Y
**Concern regarding quality of the outcome or its determination**	**QUALITYCONCERN:** ***low***/*high/unclear*
Renal biopsy is accepted gold standard, with two independent pathologist reads and consensus adjudication. Whether pathologists were blinded to clinical/predictor data is not reported, diagnosis is cross-sectional, predictor–outcome interval is appropriate.	
**B. Applicability**	
Outcome determined at the time of biopsy. No composite outcome.	
**Concern that the outcome, its definition, assessment, or timing of assessment do not match the review question or the assessor's intended use**	**APPLICABILITY CONCERN:** ***low***/*high/unclear*

Biopsy-confirmed DN vs NDRD is precisely the target classification of interest.

**DOMAIN 4: Analysis**	
**A. Quality**	
Numbers: 929 participants, 10 predictors in the final models	
RF and SVM 10 variables retained. Five-fold cross-validation (80% train / 20% test) repeated 5 times,	
AUROC/accuracy/sensitivity/specificity/PPV/NPV	
Missing data: imputed by predictive mean matching, 13 patients excluded	
	**Y/ PY/ PN/ N/ NI**
**4.1 Was there evidence that the sample size was reasonable?**	PY
**4.2 Were continuous and categorical predictors handled appropriately?**	PY
**4.3 Were participants with missing or censored data handled appropriately in the analysis?**	PY
**4.4 If methods to address class imbalance were used, was the model or the model predictions recalibrated?***	NA
**4.5 Were methods used to address potential model overfitting?**	PN
**Concern regarding quality of the analysis**	**QUALITYCONCERN:** *low/**high**/unclear*
Sample adequate and ML methods reasonable, candidate-variable ranking/selection performed on the full development dataset rather than within each cross-validation fold, risk optimistic feature-selection leakage, classification thresholds for sensitivity/specificity are not justified and model optimism is not formally quantified, no class-imbalance correction was applied	

Model evaluation

Domain 1,2 and 3 same analysis as model development

**DOMAIN 4: Analysis**	
**Risk of bias**	
External temporal validation (n=329) (april 2017 – April 2019)	
AUROC, sensitivity, specificity, PPV, NPV	
Describe any participants who were excluded from the analysis:<br>9 excluded for external validation, based on incomplete data or acute illness	
Describe missing data on predictors and outcomes as well as methods used for handling these missing data:<br>Missing values imputed by predictive mean matching	
	**Y/ PY/ PN/ N/ NI/ NA**
**4.1 Was model evaluation based on only apparent performance avoided?**	PY

	A	I	E
**4.2 Was there evidence that the sample size was reasonable?**	PY	PY	PY
**4.3 Were participants with missing or censored data handled appropriately in the analysis?**	PY	PY	PY
**4.4 If methods to address class imbalance were used, was the evaluation done in a dataset without imbalance correction?***	NA	NA	NA
**4.5 If data splitting was done to create training and test datasets, was there evidence that data leakage was avoided?***	**NA**	PA	**NA**
**4.6 If resampling methods were used to evaluate model performance, were all model development steps replicated in the resampling process?***	**NA**	NA	**NA**
**4.7 Was the predictive performance of the model evaluated appropriately, e.g., calibration, discrimination, and net benefit?**	PN	PN	PN
**Risk of bias introduced by the analysis**	**RISK OF BIAS:** *low/**high**/unclear*		
External temporal validation against gold standard and against prior models, with good discrimination, no calibration or clinical-utility/net-benefit analysis is reported, internal validation of the feature-selection step outside the cross-validation loop, so the reported internal performance is likely optimistic analytic, high risk-of-bias judgement			

Step 4 - Assess the overall concerns regarding quality, risk of bias and applicability of the prediction model

**OVERALL JUDGEMENT OF PREDICTION MODEL**	
**Overall judgement of quality (development)**	**QUALITY CONCERN:** *low/**high**/unclear*
development quality is undermined by highly selected, single-centre, biopsy-only cohort, analysis weaknesses, probable feature-selection leakage outside the cross-validation loop	
**Overall judgement of risk of bias (evaluation)**	**RISK OF BIAS:** *low/**high**/unclear*
Same selection bias as development and absence of calibration or net-benefit assessment	
**Overall judgement of applicability (development)**	**APPLICABILITY CONCERN:** *low/**high**/unclear*
Predictors and outcome match intended noninvasive DN-vs-NDRD use, single-centre Chinese biopsy population with enriched NDRD prevalence	
**Overall judgement of applicability (evaluation)**	**APPLICABILITY CONCERN:** *low/**high**/unclear*
As for development, validation population is same selected single-centre Chinese cohort	

Glazyrin et al. (28)

Step 1 - PICOTS

**Population**	Patients with CKD of three origins: diabetic nephropathy, glomerulonephritis, hypertensive nephropathy, healthy volunteers . Single centre, Russia
**Index model(s)**	KNN/SVM/LR/DT

**Comparator model(s)**	Compared against each other
**Outcome(s)**	Diagnostic class: CKD type and CKD vs healthy.
**Timing**	Cross-sectional
**Setting and intended use of the prediction model**	Clinical nephrology, porteomics-based noninvasive test to differentiate CKD origin

Step 2 – Classify the type of prediction model assessment

**Classify the assessment based on its aim**			
Type of prediction study	***Explanation***	***PROBAST+AI boxes to complete***	***Tick as appropriate***
**Development only**	**Prediction model development only, i.e., without evaluation of its performance.**	**Model development**	
**Evaluation only**	**External validation of one or more existing models in new data**	**Model evaluation**	
**Combination**	**Prediction model development combined in the same study (publication) with the evaluation of its apparent performance, internal validation performance, or external validation performance.**	**Model development and evaluation**	**X**

Step 3 - Assess quality and applicability or risk of bias and applicability

**Publication reference**	Glazyrin YE, Veprintsev DV, Ler IA, Rossovskaya ML, Varygina SA, Glizer SL, et al. Proteomics-Based Machine Learning Approach as an Alternative to Conventional Biomarkers for Differential Diagnosis of Chronic Kidney Diseases. Int J Mol Sci. 2020;21(13).
**Models of interest**	KNN, SVM, LR, DT
**Outcome of interest**	CKD-type, CKD vs. healthy

Model development

**DOMAIN 1: Participants and data sources**	
**A. Quality**	
34 CKD patients and 14 healthy volunteers from one centre; plasma and urine collected	
	**Y/ PY/ PN/ N/ NI**
**1.1 Were appropriate data sources used?**	PN

**1.2 Was an appropriate study design used?**	PN
**1.3 Did the in- and exclusions of study participants result in a representative dataset?**	PN
**Concern regarding quality of selection of participants and data sources**	**QUALITYCONCERN:** *low/**high**/unclear*
Rationale of quality rating: Very small, single-centre, by-diagnosis, one group has only 5 patients, no healthy urine collected	
**B. Applicability**	
Single Russian centre, tiny per-group numbers.	
**Concern that the (data of the) included participants do not match the review question or the assessor's intended use of the prediction model**	**APPLICABILITY CONCERN:** *low/**high**/unclear*
Rationale of applicability rating: extremely small, non-representative groups limit generalizability to CKD population	

**DOMAIN 2: Predictors**	
**A. Quality**	
Predictors: plasma (184) / urine (241) protein values, reduced by PCA, uniform MS protocol.	
	**Y/ PY/ PN/ N/ NI**
**2.1 Were predictors defined and assessed in a similar way for all participants?**	PY
**2.2 Was any pre-processing of predictors similar for all participants?**	PY
**2.3 Were predictor assessments made without knowledge of outcome data?**	Y
**2.4 Were the predictors included in the model available at the time the model was intended to be used?**	PY
**Concern regarding quality of selection of participants and data sources**	**QUALITYCONCERN:** ***low**/high/unclear*
Proteomic measurements obtained objectively under a uniform protocol, independent of diagnostic label.	
**B. Applicability**	
**Concern that the definition, pre-processing, assessment, or timing of assessment of the predictors in the model do not match the review question or the assessor's intended use**	**APPLICABILITY CONCERN:** ***low**/high/unclear*
Rationale of applicability rating: Proteomic plasma/urine profiles are intended noninvasive inputs	

**DOMAIN 3: Outcome**	
**A. Quality**	
D = presence of diabetes; H = hypertension without diabetes, only 2/14 glomerulonephritis biopsy-confirmed	
Or	**Y/ PY/ PN/ N/ NI**

**3.1 Were outcomes defined and assessed appropriately?**	N
**3.2 Were outcomes defined and assessed in a similar way for all participants?**	PN
**3.3 Were outcome assessments made without use or knowledge of predictor data?**	Y
**3.4 Was the time interval between predictor assessment and outcome assessment appropriate?**	Y
**Concern regarding quality of the outcome or its determination**	**QUALITYCONCERN:** *low/**high**/unclear*
reference standard is largely clinical, not biopsy, and partly circular (e.g. diabetes defines the DN label), target labels are weak and inconsistent.	
**B. Applicability**	
Outcome determined at the time of biopsy. No composite outcome.	
**Concern that the outcome, its definition, assessment, or timing of assessment do not match the review question or the assessor's intended use**	**APPLICABILITY CONCERN:** *low/**high**/unclear*
trained against non-biopsy clinical labels, so outcome does not match a robust diagnostic reference.	

**DOMAIN 4: Analysis**	
**A. Quality**	
after replicate-averaging/QC, plasma 90 samples, urine 47, many proteins reduced by PCA, per-group numbers tiny.	
PCA dimension reduction, multiple algorithms compared, best model selected; cross-validation on the whole dataset; mean-accuracy reported (no AUC/calibration/CIs), no imbalance correction	
low-quality samples (<50 proteins) and proteins found in <5 samples removed, replicate pairs failing the consistency check discarded, healthy urine not collected	
Missing data: no imputation, handled by filtering out sparse proteins and low-quality samples.	
	**Y/ PY/ PN/ N/ NI**
**4.1 Was there evidence that the sample size was reasonable?**	N
**4.2 Were continuous and categorical predictors handled appropriately?**	PY
**4.3 Were participants with missing or censored data handled appropriately in the analysis?**	PN
**4.4 If methods to address class imbalance were used, was the model or the model predictions recalibrated?***	NA
**4.5 Were methods used to address potential model overfitting?**	PN
**Concern regarding quality of the analysis**	**QUALITYCONCERN:** *low/**high**/unclear*
Sample size too small for the number of features, PCA and sample-consistency filtering were performed on the whole dataset before multiple models were compared and the best selected without correction; only mean accuracy is reported (no calibration or discrimination). Headline results such as 100% / >96% rest on 5–9 patients per group.	

Model evaluation

Domain 1,2 and 3 same analysis as model development

DOMAIN 4: Analysis			
**Risk of bias**			
plasma 90 samples (D 28, G 27, H 9, N 26); urine 47 (D 20, G 19, H 8). Features = 184 (plasma) / 241 (urine) proteins reduced by PCA			
mean accuracy only, no AUROC, no calibration, no confidence intervals or net benefit, not adjusted for optimism.			
Describe any participants who were excluded from the analysis: low-quality samples (<50 proteins) and proteins found in <5 samples removed, replicate pairs failing the consistency check discarded, healthy urine not collected			
Describe missing data on predictors and outcomes as well as methods used for handling these missing data: no imputation — handled by filtering out sparse proteins and low-quality samples.			
	**Y/ PY/ PN/ N/ NI/ NA**		
**4.1 Was model evaluation based on only apparent performance avoided?**	Y		
	**A**	**I**	**E**
**4.2 Was there evidence that the sample size was reasonable?**	N	N	NA
**4.3 Were participants with missing or censored data handled appropriately in the analysis?**	PN	PN	NA
**4.4 If methods to address class imbalance were used, was the evaluation done in a dataset without imbalance correction?***	NA	NA	NA
**4.5 If data splitting was done to create training and test datasets, was there evidence that data leakage was avoided?***	**NA**	PN	**NA**
**4.6 If resampling methods were used to evaluate model performance, were all model development steps replicated in the resampling process?***	**NA**	PN	**NA**
**4.7 Was the predictive performance of the model evaluated appropriately, e.g., calibration, discrimination, and net benefit?**	PN	PN	NA
**Risk of bias introduced by the analysis**	**RISK OF BIAS:** *low/**high**/unclear*		
Only cross-validation was performed, PCA done on the full dataset and not re-run inside each fold (the sample is too small, only mean accuracy is reported with no calibration, discrimination or confidence intervals, reported accuracies optimistic and unstable.			

Step 4 - Assess the overall concerns regarding quality, risk of bias and applicability of the prediction model

OVERALL JUDGEMENT OF PREDICTION MODEL	
**Overall judgement of quality (development)**	**QUALITY CONCERN:** *low/**high**/unclear*
Tiny non-representative sample, clinical reference labels, analysis with severe overfitting risk and full-dataset data leakage.	
**Overall judgement of risk of bias (evaluation)**	**RISK OF BIAS:** *low/**high**/unclear*
Cross-validation with leakage on a very small sample, weak reference labels, accuracy-only metrics, no external validation.	
**Overall judgement of applicability (development)**	**APPLICABILITY CONCERN:** *low/**high**/unclear*
Proteomic inputs fit the intended use, tiny single-centre groups, non-gold-standard outcome	

**Overall judgement of applicability (evaluation)**	**APPLICABILITY CONCERN:**<br>*low/**high**/unclear*
Same limitations as development	

Zhao et al. (29)

Step 1 - PICOTS

**Population**	Adults with CKD, attending a single Chinese centre.
**Index model(s)**	Ensemble learning model developed by Liu et al. 2017.
**Comparator model(s)**	Asian-modified CKD-EPI equation
**Outcome(s)**	Continuous estimation of glomerular filtration rate (mL/min/1.73 m²). Reference: mGFR by 99mTc-DTPA dual plasma sample clearance.
**Timing**	Cross-sectional
**Setting and intended use of the prediction model**	Clinical nephrology, routine GFR estimation in Chinese CKD patients to guide diagnosis, staging and management.

Step 2 – Classify the type of prediction model assessment

**Classify the assessment based on its aim**			
Type of prediction study	***Explanation***	***PROBAST+AI boxes to complete***	***Tick as appropriate***
**Development only**	**Prediction model development only, i.e., without evaluation of its performance.**	**Model development**	
**Evaluation only**	**External validation of one or more existing models in new data**	**Model evaluation**	**x**
**Combination**	**Prediction model development combined in the same study (publication) with the evaluation of its apparent performance, internal validation performance, or external validation performance.**	**Model development and evaluation**	

Step 3 - Assess quality and applicability or risk of bias and applicability

**Publication reference**	Zhao L, Zhang JJ, Tian X, Huang JM, Xie P, Li XZ. The ensemble learning model is not better than the Asian modified CKD-EPI equation for glomerular filtration rate estimation in Chinese CKD patients in the external validation study. BMC Nephrol. 2021;22(1):372.
**Models of interest**	Ensemble learning model (NN, SVM, regression)
**Outcome of interest**	Estimated GFR vs measured GFR by 99mTc-DTPA dual plasma clearance.

Model Evaluation

**DOMAIN 1: Participants and data sources**	
**A. Quality**	
192 Chinese CKD patients undergoing 99mTc-DTPA at a single hospital. After exclusions: 158 analysed	
	**Y/ PY/ PN/ N/ NI**
**1.1 Were appropriate data sources used?**	PY
**1.2 Was an appropriate study design used?**	PY
**1.3 Did the in- and exclusions of study participants result in a representative dataset?**	PN
**Concern regarding quality of selection of participants and data sources**	**QUALITYCONCERN:** *low/**high**/unclear*
Rationale of quality rating: Independent cohort and reasonable consecutive sample, 12 “outliers” removed without pre-specified criteria, can distort performance estimates and raises selection-bias concern.	
**B. Applicability**	
Single Chinese centre, mixed CKD aetiologies,	
**Concern that the (data of the) included participants do not match the review question or the assessor’s intended use of the prediction model**	**APPLICABILITY CONCERN:** ***low**/high/unclear*
Rationale of applicability rating: Population matches the intended use, aetiologic mix	

**DOMAIN 2: Predictors**	
**A. Quality**	
Predictors: serum creatinine, age, sex — i.e. the inputs required by both equations. Assessed at the same visit as mGFR.	
	**Y/ PY/ PN/ N/ NI**
**2.1 Were predictors defined and assessed in a similar way for all participants?**	Y
**2.2 Was any pre-processing of predictors similar for all participants?**	Y
**2.3 Were predictor assessments made without knowledge of outcome data?**	Y

**2.4 Were the predictors included in the model available at the time the model was intended to be used?**	Y
**Concern regarding quality of selection of participants and data sources**	**QUALITYCONCERN:** ***low****/high/unclear*
Scr was measured by an enzymatic assay uniformly across patients, independent of mGFR.	
**B. Applicability**	
**Concern that the definition, pre-processing, assessment, or timing of assessment of the predictors in the model do not match the review question or the assessor's intended use**	**APPLICABILITY CONCERN:** ***low****/high/unclear*
Rationale of applicability rating: Scr, age, sex are routine, low-cost inputs aligned with intended use.	

**DOMAIN 3: Outcome**	
**A. Quality**	
mGFR by 99mTc-DTPA dual plasma sample clearance, recognised reference method	
Or	**Y/ PY/ PN/ N/ NI**
**3.1 Were outcomes defined and assessed appropriately?**	Y
**3.2 Were outcomes defined and assessed in a similar way for all participants?**	Y
**3.3 Were outcome assessments made without use or knowledge of predictor data?**	PY
**3.4 Was the time interval between predictor assessment and outcome assessment appropriate?**	Y
**Concern regarding quality of the outcome or its determination**	**QUALITYCONCERN:** ***low****/high/unclear*
Dual-plasma 99mTc-DTPA with standard physiological corrections is an accepted reference, applied uniformly, mGFR is an independent quantitative measurement	
**B. Applicability**	
Outcome determined at the same visit; continuous GFR (no composite).	
**Concern that the outcome, its definition, assessment, or timing of assessment do not match the review question or the assessor's intended use**	**APPLICABILITY CONCERN:** ***low****/high/unclear*
Inulin clearance is strict gold standard, but 99mTc-DTPA dual-plasma clearance is accepted clinical reference	

**DOMAIN 4: Analysis**
**Risk of bias**
158 analysed (12 outliers removed, predictors: 3 (Scr, age, sex), GFR is continuous
Spearman correlation, linear regression slope/intercept, Bland-Altman 95% limits of agreement, bias (median difference, 95% CI), precision (IQR of differences), and accuracy P30. Wilcoxon signed-rank, McNemar and bootstrap precision tests compared the two models.

Describe any participants who were excluded from the analysis:<br>34 of 192 excluded as above (incl. 12 outliers), no other exclusions			
Describe missing data on predictors and outcomes as well as methods used for handling these missing data:<br>patients with missing Scr/age/weight excluded (n=7), no imputation			
	**Y/ PY/ PN/ N/ NI/ NA**		
**4.1 Was model evaluation based on only apparent performance avoided?**	Y		
	**A**	**I**	**E**
**4.2 Was there evidence that the sample size was reasonable?**	NA	NA	PY
**4.3 Were participants with missing or censored data handled appropriately in the analysis?**	NA	NA	PN
**4.4 If methods to address class imbalance were used, was the evaluation done in a dataset without imbalance correction?***	NA	NA	NA
**4.5 If data splitting was done to create training and test datasets, was there evidence that data leakage was avoided?***	**NA**	NA	**NA**
**4.6 If resampling methods were used to evaluate model performance, were all model development steps replicated in the resampling process?***	**NA**	NA	**NA**
**4.7 Was the predictive performance of the model evaluated appropriately, e.g., calibration, discrimination, and net benefit?**	NA	NA	PY
**Risk of bias introduced by the analysis**	**RISK OF BIAS:**<br>*low/**high**/unclear*		
performance was assessed appropriately and comprehensively, sample size of 158 is adequate for. 12 patients removed as outliers without pre-specified criteria and impact not tested, no data splitting or resampling for model development performed			

Step 4 - Assess the overall concerns regarding quality, risk of bias and applicability of the prediction model

**OVERALL JUDGEMENT OF PREDICTION MODEL**	
**Overall judgement of quality (development)**	**QUALITY CONCERN:**<br>low/high/unclear
NA – evaluation only	
**Overall judgement of risk of bias (evaluation)**	**RISK OF BIAS:**<br>*low/**high**/unclear*
Strong design overall, high rating is driven by the undocumented removal of 12 outliers and lack of sensitivity analysis, missing-data handling beyond exclusion is not described.	
**Overall judgement of applicability (development)**	**APPLICABILITY CONCERN:**<br>*low/high/unclear*
NA – evaluation only	
**Overall judgement of applicability (evaluation)**	**APPLICABILITY CONCERN:**<br>***low**/high/unclear*
Population, predictors and reference standard all match intended use of routine GFR estimation in Chinese CKD patients, findings should generalise reasonably to similar settings	

Lanot et al. (30)

Step 1 - PICOTS

**Population**	Participants of all ages (2–97 y) from pooled European and US adult cohorts, all with

	measured GFR by an exogenous marker (iohexol, inulin, 51Cr-EDTA, iothalamate).
**Index model(s)**	RF/XGBoost/KNN/SVM
**Comparator model(s)**	Two LR models
**Outcome(s)**	eGFR within p30 (and separately p20, p10) of mGFR.
**Timing**	Cross-sectional
**Setting and intended use of the prediction model**	Clinical nephrology to aid the decision to confirm eGFR

Step 2 – Classify the type of prediction model assessment

**Classify the assessment based on its aim**			
Type of prediction study	***Explanation***	***PROBAST+AI boxes to complete***	***Tick as appropriate***
**Development only**	**Prediction model development only, i.e., without evaluation of its performance.**	**Model development**	
**Evaluation only**	**External validation of one or more existing models in new data**	**Model evaluation**	
**Combination**	**Prediction model development combined in the same study (publication) with the evaluation of its apparent performance, internal validation performance, or external validation performance.**	**Model development and evaluation**	**X**

Step 3 - Assess quality and applicability or risk of bias and applicability

**Publication reference**	Lanot A, Akesson A, Nakano FK, Vens C, Bjoerk J, Nyman U, et al. Enhancing individual glomerular filtration rate assessment: can we trust the equation? Development and vali-dation of machine learning models to assess the trustworthiness of estimated GFR com-pared to measured GFR. BMC Nephrol. 2025;26(1).
**Models of interest**	RF/XGBoost/KNN/SVM/LR
**Outcome of interest**	eGFR within p30/p20/p10 of mGFR

Model development

**DOMAIN 1: Participants and data sources**

A. Quality	
Training cohort n=9,202, 248 patients with missing weight/height excluded	
	**Y/ PY/ PN/ N/ NI**
**1.1 Were appropriate data sources used?**	Y
**1.2 Was an appropriate study design used?**	PY
**1.3 Did the in- and exclusions of study participants result in a representative dataset?**	PY
**Concern regarding quality of selection of participants and data sources**	**QUALITYCONCERN:** ***low****/high/unclear*
Rationale of quality rating: Large pooled multi-country cohort with mGFR by exogenous markers, broad age range, exclusion of Black participants, reliance on existing research cohorts	
**B. Applicability**	
European + US adult research cohorts; ages 2–97 y.	
**Concern that the (data of the) included participants do not match the review question or the assessor's intended use of the prediction model**	**APPLICABILITY CONCERN:** *low/high/****unclear***
Rationale of applicability rating: intended use for European/US (non-Black) patients; transportability to Black or other ancestries unproven	

DOMAIN 2: Predictors	
**A. Quality**	
Age, sex, height, weight, creatinine, EKFC creatinine-based eGFR. Scaled and centred.	
	**Y/ PY/ PN/ N/ NI**
**2.1 Were predictors defined and assessed in a similar way for all participants?**	Y
**2.2 Was any pre-processing of predictors similar for all participants?**	Y
**2.3 Were predictor assessments made without knowledge of outcome data?**	Y
**2.4 Were the predictors included in the model available at the time the model was intended to be used?**	Y
**Concern regarding quality of selection of participants and data sources**	**QUALITYCONCERN:** ***low****/high/unclear*
Routine, objective measurements obtained uniformly and independently of the outcome.	
**B. Applicability**	
**Concern that the definition, pre-processing, assessment, or timing of assessment of the predictors in the model do not match the review question or the assessor's intended use**	**APPLICABILITY CONCERN:** ***low****/high/unclear*
Rationale of applicability rating: All predictors are routinely available at the intended point of clinical use.	

DOMAIN 3: Outcome	
**A. Quality**	
EKFC eGFR within ±30/20/10% of mGFR, reference mGFR by iohexol, inulin, 51Cr-EDTA or iothalamate	
Or	**Y/ PY/ PN/ N/ NI**
**3.1 Were outcomes defined and assessed appropriately?**	Y
**3.2 Were outcomes defined and assessed in a similar way for all participants?**	PY
**3.3 Were outcome assessments made without use or knowledge of predictor data?**	Y
**3.4 Was the time interval between predictor assessment and outcome assessment appropriate?**	Y
**Concern regarding quality of the outcome or its determination**	**QUALITYCONCERN:** ***low**/high/unclear*
mGFR by exogenous tracer is appropriate referenc, minor heterogeneity in tracer across cohorts	
**B. Applicability**	
Outcome determined at same visit as predictors, dichotomised reference directly aligned with intended clinical decision threshold.	
**Concern that the outcome, its definition, assessment, or timing of assessment do not match the review question or the assessor's intended use**	**APPLICABILITY CONCERN:** ***low**/high/unclear*
Matches the intended clinical decision	

DOMAIN 4: Analysis	
**A. Quality**	
training n=9,202; ~86% positives for P30 (high class imbalance for P20/P10), 6 predictors	
10-fold inner CV, no formal calibration analysis	
AUC, accuracy, sensitivity, specificity, PPV, NPV, lass-imbalance not corrected	
Missing data: 248 subjects with missing weight/height excluded	
	**Y/ PY/ PN/ N/ NI**
**4.1 Was there evidence that the sample size was reasonable?**	Y
**4.2 Were continuous and categorical predictors handled appropriately?**	PY
**4.3 Were participants with missing or censored data handled appropriately in the analysis?**	PY
**4.4 If methods to address class imbalance were used, was the model or the model predictions recalibrated?***	NA
**4.5 Were methods used to address potential model overfitting?**	PY

Concern regarding quality of the analysis	QUALITYCONCERN: *low/high/**unclear***
Sample size is large and CV is appropriate, discrimination alone is reported, class imbalance is not formally corrected	

Model evaluation

Domain 1,2 and 3 same analysis as model development

**DOMAIN 4: Analysis**			
**Risk of bias**			
training 9,202; internal validation 3,034; external validation 10,107; 6 predictors.			
AUROC, accuracy, sensitivity, PPV, NPV			
Describe any participants who were excluded from the analysis: external cohort had no additional exclusions beyond missing weight/height (already excluded in 248 development subjects)			
Describe missing data on predictors and outcomes as well as methods used for handling these missing data: Missing data: <1%, complete-case analysis			
	**Y/ PY/ PN/ N/ NI/ NA**		
**4.1 Was model evaluation based on only apparent performance avoided?**	Y		
	**A**	**I**	**E**
**4.2 Was there evidence that the sample size was reasonable?**	NA	Y	Y
**4.3 Were participants with missing or censored data handled appropriately in the analysis?**	NA	PY	PY
**4.4 If methods to address class imbalance were used, was the evaluation done in a dataset without imbalance correction?***	NA	NA	NA
**4.5 If data splitting was done to create training and test datasets, was there evidence that data leakage was avoided?***	**NA**	PY	**NA**
**4.6 If resampling methods were used to evaluate model performance, were all model development steps replicated in the resampling process?***	**NA**	PY	**NA**
**4.7 Was the predictive performance of the model evaluated appropriately, e.g., calibration, discrimination, and net benefit?**	NA	PN	NN
**Risk of bias introduced by the analysis**	**RISK OF BIAS:** *low/**high**/unclear*		
Strong external validation in independent multi-country cohort with large numbers and honest report that the modest improvement over EKFC alone is small, no calibration analysis is reported, despite fixed 80% threshold			

Step 4 - Assess the overall concerns regarding quality, risk of bias and applicability of the prediction model

**OVERALL JUDGEMENT OF PREDICTION MODEL**	
**Overall judgement of quality (development)**	**QUALITY CONCERN:** *low/high/**unclear***
Large, well-designed development with appropriate predictors, gold-standard mGFR outcome, cross-validated tuning, calibration is assessed even though probability outputs drive a fixed decision threshold.	

**Overall judgement of risk of bias (evaluation)**	**RISK OF BIAS:**<br>*low/**high**/unclear*
independent external validation in 10,107 participants, no calibration plot, no decision-curve analysis, marginal gains over EKFC alone,	
**Overall judgement of applicability (development)**	**APPLICABILITY CONCERN:**<br>*low/high/**unclear***
Findings should generalise to European/US non-Black populations, transportability to other ancestries and to routine-care is unproven and explicitly flagged by authors.	
**Overall judgement of applicability (evaluation)**	**APPLICABILITY CONCERN:**<br>*low/high/**unclear***
Same limitations as development	

Chen et al. (31)

Step 1 - PICOTS

**Population**	Outpatients aged 18–75 years in Taiwan.
**Index model(s)**	SVM with different SVM kernels
**Comparator model(s)**	SVM kernels compared internally; no external comparator.
**Outcome(s)**	CKD stage 1–5, defined by eGFR thresholds
**Timing**	Cross-sectional
**Setting and intended use of the prediction model**	Hospital ultrasound clinic, intended as a complementary tool for CKD-stage diagnosis.

Step 2 – Classify the type of prediction model assessment

**Classify the assessment based on its aim**			
Type of prediction study	***Explanation***	***PROBAST+AI boxes to complete***	***Tick as appropriate***
**Development only**	**Prediction model development only, i.e., without evaluation of its performance.**	**Model development**	
**Evaluation only**	**External validation of one or more existing models in new data**	**Model evaluation**	
**Combination**	**Prediction model development combined in the same study (publication) with the evaluation of its apparent performance, internal validation performance, or external validation performance.**	**Model development and evaluation**	**X**

Step 3 - Assess quality and applicability or risk of bias and applicability

**Publication reference**	Chen CJ, Pai TW, Hsu HH, Lee CH, Chen KS, Chen YC. Prediction of chronic kidney dis-ease stages by renal ultrasound imaging. Enterprise Information Systems. 2020;14(2):178–95.
**Models of interest**	SVM (RBF kernel) using hand-crafted texture/area/brightness features from US-image ROIs.
**Outcome of interest**	5-class CKD stage

Model development

**DOMAIN 1: Participants and data sources**	
**A. Quality**	
205 patients, 798 kidney images (per-stage left-kidney counts 127/69/46/118/43, right-kidney counts 119/69/47/119/41)	
	**Y/ PY/ PN/ N/ NI**
**1.1 Were appropriate data sources used?**	PN
**1.2 Was an appropriate study design used?**	PN
**1.3 Did the in- and exclusions of study participants result in a representative dataset?**	N
**Concern regarding quality of selection of participants and data sources**	**QUALITYCONCERN:** *low/**high**/unclear*
Rationale of quality rating: Small single-centre sample, exclusion of diabetics, inclusion of multiple images per patient (and left/right of the same kidney) without addressing within-patient correlation	
**B. Applicability**	
Single-centre, strict exclusions: diabetics, acute renal failure, PKD, hydronephrosis, inpatients.	
**Concern that the (data of the) included participants do not match the review question or the assessor's intended use of the prediction model**	**APPLICABILITY CONCERN:** *low/**high**/unclear*
Rationale of applicability rating: no match with realistic CKD population	

**DOMAIN 2: Predictors**	
**A. Quality**	
hand-crafted features from manually segmented ROIs (parenchyma, central sinus, whole kidney), entropy, contrast, homogeneity, energy (GLCM), standard deviation, coefficient of variation, parenchyma area ratio, brightness ratio.	
	**Y/ PY/ PN/ N/ NI**
**2.1 Were predictors defined and assessed in a similar way for all participants?**	PY
**2.2 Was any pre-processing of predictors similar for all participants?**	PY
**2.3 Were predictor assessments made without knowledge of outcome data?**	NI

**2.4 Were the predictors included in the model available at the time the model was intended to be used?**	PY
**Concern regarding quality of selection of participants and data sources**	**QUALITYCONCERN:** *low/**high**/unclear*
Manual ROI segmentation by physicians who also diagnosed CKD-stage, risk of outcome-aware feature extraction, single machine acquisition	
**B. Applicability**	
**Concern that the definition, pre-processing, assessment, or timing of assessment of the predictors in the model do not match the review question or the assessor's intended use**	**APPLICABILITY CONCERN:** *low/**high**/unclear*
Rationale of applicability rating: Reliance on manual ROI segmentation and a specific scanner model limits applicability	

**DOMAIN 3: Outcome**	
**A. Quality**	
CKD stage by NKF/K-DOQI eGFR thresholds, determined by two physicians using both eGFR and US images, US-based reference labelling	
Or	**Y/ PY/ PN/ N/ NI**
**3.1 Were outcomes defined and assessed appropriately?**	PN
**3.2 Were outcomes defined and assessed in a similar way for all participants?**	PY
**3.3 Were outcome assessments made without use or knowledge of predictor data?**	N
**3.4 Was the time interval between predictor assessment and outcome assessment appropriate?**	PY
**Concern regarding quality of the outcome or its determination**	**QUALITYCONCERN:** *low/**high**/unclear*
Reference label assigned by physicians who used same US images as the model input, incorporation bias, the model trained to predict label depends on inputs	
**B. Applicability**	
Outcome determined at the time of US examination, CKD-stage label assigned by physicians who considered eGFR AND the US image	
**Concern that the outcome, its definition, assessment, or timing of assessment do not match the review question or the assessor's intended use**	**APPLICABILITY CONCERN:** *low/high/**unclear***
eGFR-defined CKD-stage is intended target, US-aware labelling biases apparent target.	

**DOMAIN 4: Analysis**	
**A. Quality**	
798 images from 205 patients across 5 classes, severe per-class scarcity and heavy class imbalance	
10-fold inner CV, no formal calibration analysis	

Only accuracy reported
poor-quality images excluded, ROIs reconstructed by inpainting.

	Y/ PY/ PN/ N/ NI
**4.1 Was there evidence that the sample size was reasonable?**	N
**4.2 Were continuous and categorical predictors handled appropriately?**	PY
**4.3 Were participants with missing or censored data handled appropriately in the analysis?**	PN
**4.4 If methods to address class imbalance were used, was the model or the model predictions recalibrated?***	NA
**4.5 Were methods used to address potential model overfitting?**	PN
**Concern regarding quality of the analysis**	**QUALITYCONCERN:** *low/**high**/unclear*
Sample size too small for a 5-class classifier from 205 patients, 10-fold CV likely splits at the image level, mixing left/right images of the same patient between train and test folds, multiple kernels were tried without correction for model selection, no calibration, no AUC, no class-imbalance correction, only mean accuracy reported.	

Model evaluation

Domain 1,2 and 3 same analysis as model development

**DOMAIN 4: Analysis**			
**Risk of bias**			
798 images from 205 patients across 5 classes, severe per-class scarcity and heavy class imbalance			
10-fold CV, only accuracy reported, no AUC, no calibration, no sensitivity, no specificity, no CI, no external validation			
Describe any participants who were excluded from the analysis: Exclusion same as development			
Describe missing data on predictors and outcomes as well as methods used for handling these missing data: Missing data same as development			
	Y/ PY/ PN/ N/ NI/ NA		
**4.1 Was model evaluation based on only apparent performance avoided?**	Y		
	**A**	**I**	**E**
**4.2 Was there evidence that the sample size was reasonable?**	N	N	NA
**4.3 Were participants with missing or censored data handled appropriately in the analysis?**	PN	PN	NA
**4.4 If methods to address class imbalance were used, was the evaluation done in a dataset without imbalance correction?***	NA	NA	NA
**4.5 If data splitting was done to create training and test datasets, was there evidence that data leakage was avoided?***	**NA**	PN	**NA**
**4.6 If resampling methods were used to evaluate model performance, were all model development steps replicated in the resampling process?***	**NA**	PN	**NA**

4.7 Was the predictive performance of the model evaluated appropriately, e.g., calibration, discrimination, and net benefit?	PN	PN	NA
**Risk of bias introduced by the analysis**	**RISK OF BIAS:** *low/**high**/unclear*		
Severe per-class sample-size limits, plausible within-patient leakage at the image level, kernel selection by trial-and-error without correction, accuracy-only reporting, no external validation			

Step 4 - Assess the overall concerns regarding quality, risk of bias and applicability of the prediction model

OVERALL JUDGEMENT OF PREDICTION MODEL	
**Overall judgement of quality (development)**	**QUALITY CONCERN:** *low/**high**/unclear*
All four domains high concern	
**Overall judgement of risk of bias (evaluation)**	**RISK OF BIAS:** *low/**high**/unclear*
Only internal 10-fold CV, no external validation, multiple sources of likely optimism.	
**Overall judgement of applicability (development)**	**APPLICABILITY CONCERN:** *low/**high**/unclear*
Domain 1 and 2 high concern, domain 3 unclear	
**Overall judgement of applicability (evaluation)**	**APPLICABILITY CONCERN:** *low/**high**/unclear*
Same limitations as development	

Tian et al. (32)

Step 1 - PICOTS

**Population**	1,049 patients from a single hospital in China
**Index model(s)**	Deep learning radiomics model
**Comparator model(s)**	Junior and senior radiologists
**Outcome(s)**	non-CKD and CKD stages
**Timing**	Cross-sectional
**Setting and intended use of the prediction model**	Hospital ultrasound clinic in China, diagnostic aid for CKD detection and staging

Step 2 – Classify the type of prediction model assessment

Classify the assessment based on its aim			
Type of prediction study	***Explanation***	***PROBAST+AI boxes to complete***	***Tick as appropriate***

**Development only**	**Prediction model development only, i.e., without evaluation of its performance.**	**Model development**	
**Evaluation only**	**External validation of one or more existing models in new data**	**Model evaluation**	
**Combination**	**Prediction model development combined in the same study (publication) with the evaluation of its apparent performance, internal validation performance, or external validation performance.**	**Model development and evaluation**	**X**

Step 3 - Assess quality and applicability or risk of bias and applicability

**Publication reference**	Tian S, Yu Y, Shi K, Jiang Y, Song H, Wang Y, et al. Deep learning radiomics based on ul-trasound images for the assisted diagnosis of chronic kidney disease. Nephrology (Carl-ton). 2024;29(11):748–57.
**Models of interest**	Deep learning radiomics fusing ResNet-34 + GLCM + HOG texture features.
**Outcome of interest**	CKD vs non-CKD + 5-stage classification.

Model development

**DOMAIN 1: Participants and data sources**	
**A. Quality**	
870 CKD + 179 non-CKD, total images n= 4365, non-CKD images n=756, same-patient images kept in the same split, severe class imbalance	
	**Y/ PY/ PN/ N/ NI**
**1.1 Were appropriate data sources used?**	PY
**1.2 Was an appropriate study design used?**	PY
**1.3 Did the in- and exclusions of study participants result in a representative dataset?**	PN
**Concern regarding quality of selection of participants and data sources**	**QUALITYCONCERN:** *low/high/**unclear***
Rationale of quality rating: Reasonable sample size and patient-level splitting, single-centre Chinese cohort, disproportionate non-CKD vs CKD distribution, structural exclusion of nodular kidneys	
**B. Applicability**	
Single-centre, exclusion of solitary kidney, kidney nodules, unqualified US images, images of poor quality	
**Concern that the (data of the) included participants do not match the review question or the assessor's intended use of the prediction model**	**APPLICABILITY CONCERN:** *low/**high**/unclear*

Rationale of applicability rating: Single-centre Chinese sample with no external validation, the authors themselves flag this limitation

DOMAIN 2: Predictors	
**A. Quality**	
ResNet-34 deep features + GLCM texture + HOG features, fused into a unified representation, U-Net + RNN renal-cortex segmentation.	
	**Y/ PY/ PN/ N/ NI**
**2.1 Were predictors defined and assessed in a similar way for all participants?**	Y
**2.2 Was any pre-processing of predictors similar for all participants?**	Y
**2.3 Were predictor assessments made without knowledge of outcome data?**	PY
**2.4 Were the predictors included in the model available at the time the model was intended to be used?**	Y
**Concern regarding quality of selection of participants and data sources**	**QUALITYCONCERN:** ***low****/high/unclear*
Predictors extracted from US images by a uniform automated pipeline, features are computed automatically and independently of the eGFR label.	
**B. Applicability**	
**Concern that the definition, pre-processing, assessment, or timing of assessment of the predictors in the model do not match the review question or the assessor's intended use**	**APPLICABILITY CONCERN:** *low/high/****unclear***
Rationale of applicability rating: Single-machine (LOGIQ E9) acquisition without external scanner validation	

DOMAIN 3: Outcome	
**A. Quality**	
KDIGO 2012 stage based on eGFR within 24 h of kidney US examination	
Or	**Y/ PY/ PN/ N/ NI**
**3.1 Were outcomes defined and assessed appropriately?**	Y
**3.2 Were outcomes defined and assessed in a similar way for all participants?**	Y
**3.3 Were outcome assessments made without use or knowledge of predictor data?**	Y
**3.4 Was the time interval between predictor assessment and outcome assessment appropriate?**	Y
**Concern regarding quality of the outcome or its determination**	**QUALITYCONCERN:** ***low****/high/unclear*
eGFR is the standard KDIGO criterium, assayed objectively and independently of US image	
**B. Applicability**	
Outcome determined within 24 h of US examination; KDIGO eGFR-based stage is standard clinical target.	

**Concern that the outcome, its definition, assessment, or timing of assessment do not match the review question or the assessor's intended use**	**APPLICABILITY CONCERN:** ***low**/high/unclear*
eGFR-defined CKD/stage matches the intended diagnostic target.	

**DOMAIN 4: Analysis**	
**A. Quality**	
4,365 images, 8/1/1 train/val/test split at the patient level, six classes	
ImageNet-pretrained ResNet-34 fine-tuned on US images, no formal class-imbalance correction, only accuracy/sensitivity/specificity/AUC reported, comparison with radiologists by Mann–Whitney U and chi-square.	
Exclusion: solitary kidney / unilateral nephrectomy, nodules, poor image quality.	
not explicitly described, US-image and same-day eGFR were inclusion requirements.	
	**Y/ PY/ PN/ N/ NI**
**4.1 Was there evidence that the sample size was reasonable?**	PY
**4.2 Were continuous and categorical predictors handled appropriately?**	PY
**4.3 Were participants with missing or censored data handled appropriately in the analysis?**	PY
**4.4 If methods to address class imbalance were used, was the model or the model predictions recalibrated?***	NA
**4.5 Were methods used to address potential model overfitting?**	PY
**Concern regarding quality of the analysis**	**QUALITYCONCERN:** *low/high/**unclear***
Patient-level splitting, no calibration assessment, no decision-curve analysis, no class-imbalance correction, no confidence intervals on AUC, and a single fixed 8/1/1 split rather than cross-validation or repeated splits.	

Model evaluation

Domain 1,2 and 3 same analysis as model development

**DOMAIN 4: Analysis**
**Risk of bias**
Around 10% of patients in the test set, performance reported per class
10-fold CV, only accuracy reported, no AUC, no calibration, no sensitivity, no specificity, no CI, no external validation
Describe any participants who were excluded from the analysis: Exclusion same as development
Describe missing data on predictors and outcomes as well as methods used for handling these missing data: Missing data same as development

	Y/ PY/ PN/ N/ NI/ NA		
**4.1 Was model evaluation based on only apparent performance avoided?**	Y		
	**A**	**I**	**E**
**4.2 Was there evidence that the sample size was reasonable?**	PY	PY	NA
**4.3 Were participants with missing or censored data handled appropriately in the analysis?**	PY	PY	NA
**4.4 If methods to address class imbalance were used, was the evaluation done in a dataset without imbalance correction?***	NA	NA	NA
**4.5 If data splitting was done to create training and test datasets, was there evidence that data leakage was avoided?***	**NA**	PY	**NA**
**4.6 If resampling methods were used to evaluate model performance, were all model development steps replicated in the resampling process?***	**NA**	PN	**NA**
**4.7 Was the predictive performance of the model evaluated appropriately, e.g., calibration, discrimination, and net benefit?**	PN	PN	NA
**Risk of bias introduced by the analysis**	**RISK OF BIAS:** *low/**high**/unclear*		
Patient-level splitting and head-to-head radiologist comparison, absence of external validation, no calibration or net-benefit analysis, reliance on a single hold-out split with no confidence intervals			

Step 4 - Assess the overall concerns regarding quality, risk of bias and applicability of the prediction model

**OVERALL JUDGEMENT OF PREDICTION MODEL**	
**Overall judgement of quality (development)**	**QUALITY CONCERN:** *low/**high**/unclear*
High due to selection issues (D1) and analytic gaps (D4)	
**Overall judgement of risk of bias (evaluation)**	**RISK OF BIAS:** *low/**high**/unclear*
Only an internal hold-out test, no calibration, no external validation.	
**Overall judgement of applicability (development)**	**APPLICABILITY CONCERN:** *low/**high**/unclear*
Single-centre Chinese cohort, single scanner, no external validation	
**Overall judgement of applicability (evaluation)**	**APPLICABILITY CONCERN:** *low/**high**/unclear*
Same single-centre limitations.	

Weaver et al. (33)

Step 1 - PICOTS

**Population**	Boys with biopsy/cystoscopy-confirmed PUV in first year of life at Children's Hospital of Philadelphia (CHOP) or Hospital for Sick Children, Toronto (HSC)

**Index model(s)**	Three RF models: imaging, clinical and ensemble, time-dependent versions at 6 mo, 1 y, 3 y, 5 y.
**Comparator model(s)**	Compared against each other
**Outcome(s)**	CKD progression: kidney failure (dialysis/transplant) or >50% decline in eGFR
**Timing**	Retrospective, median follow-up 6.1 years
**Setting and intended use of the prediction model**	Tertiary paediatric urology/nephrology, risk-stratify newborns with PUV

Step 2 – Classify the type of prediction model assessment

**Classify the assessment based on its aim**			
Type of prediction study	***Explanation***	***PROBAST+AI boxes to complete***	***Tick as appropriate***
**Development only**	**Prediction model development only, i.e., without evaluation of its performance.**	**Model development**	
**Evaluation only**	**External validation of one or more existing models in new data**	**Model evaluation**	
**Combination**	**Prediction model development combined in the same study (publication) with the evaluation of its apparent performance, internal validation performance, or external validation performance.**	**Model development and evaluation**	**X**

Step 3 - Assess quality and applicability or risk of bias and applicability

**Publication reference**	Weaver JK, Milford K, Rickard M, Logan J, Erdman L, Viteri B, et al. Deep learning imaging features derived from kidney ultrasounds predict chronic kidney disease progression in children with posterior urethral valves. Pediatr Nephrol. 2023;38(3):839–46.
**Models of interest**	imaging, clinical and ensemble RF models at 6 mo, 1 y, 3 y, 5 y.
**Outcome of interest**	Time to CKD progression (dialysis/transplant or eGFR halved)

Model development

**DOMAIN 1: Participants and data sources**
**A. Quality**

Retrospective cohort of 537 PUV patients identified, 225 included after requiring a US in first 90 days and a recorded nadir creatinine in year 1.	
	**Y/ PY/ PN/ N/ NI**
**1.1 Were appropriate data sources used?**	P
**1.2 Was an appropriate study design used?**	PY
**1.3 Did the in- and exclusions of study participants result in a representative dataset?**	PN
**Concern regarding quality of selection of participants and data sources**	**QUALITYCONCERN:** *low/**high**/unclear*
Rationale of quality rating: Multi-centre with biopsy/cystoscopy-confirmed PUV, restricting inclusion to early US and complete year-1 nadir creatinine excludes many eligible patients, source of selection bias that the authors flag, boys presenting later in life are excluded	
**B. Applicability**	
Only two centres in North-America, high standard for inclusion, diagnoses after 1y not included	
**Concern that the (data of the) included participants do not match the review question or the assessor's intended use of the prediction model**	**APPLICABILITY CONCERN:** *low/high/**unclear***
Rationale of applicability rating: Population matches intended early-postnatal use, generalizability beyond two North American tertiary centres unproven.	

**DOMAIN 2: Predictors**	
**A. Quality**	
Imaging: ResNet-50 deep features, clinical features: VUR, nadir creatinine in year 1, CIC, vesicostomy, catheterisable channel, anticholinergic use, prior ablation, concomitant genetic abnormality.	
	**Y/ PY/ PN/ N/ NI**
**2.1 Were predictors defined and assessed in a similar way for all participants?**	PY
**2.2 Was any pre-processing of predictors similar for all participants?**	PY
**2.3 Were predictor assessments made without knowledge of outcome data?**	Y
**2.4 Were the predictors included in the model available at the time the model was intended to be used?**	Y
**Concern regarding quality of selection of participants and data sources**	**QUALITYCONCERN:** ***low**/high/unclear*
Imaging features extracted by a uniform automated pipeline, clinical predictors are routine	
**B. Applicability**	
**Concern that the definition, pre-processing, assessment, or timing of assessment of the predictors in the model do not match the review question or the assessor's intended use**	**APPLICABILITY CONCERN:** ***low**/high/unclear*
Rationale of applicability rating: All predictors available at the intended decision points in routine paediatric urology care	

**DOMAIN 3: Outcome**	
**A. Quality**	
Composite CKD progression: dialysis, transplant, or >50% eGFR decline, eGFR only calculated when creatinine and height were within 6 months of each other.	
Or	**Y/ PY/ PN/ N/ NI**
**3.1 Were outcomes defined and assessed appropriately?**	PY
**3.2 Were outcomes defined and assessed in a similar way for all participants?**	PY
**3.3 Were outcome assessments made without use or knowledge of predictor data?**	PY
**3.4 Was the time interval between predictor assessment and outcome assessment appropriate?**	Y
**Concern regarding quality of the outcome or its determination**	**QUALITYCONCERN:** ***low***/*high/unclear*
Composite of objective kidney-failure events plus eGFR halving is standard CKD-progression endpoint, sensitivity analysis with the U25 CKiD equation, outcome independent of imaging predictors.	
**B. Applicability**	
Composite endpoint of dialysis/transplant or >50% eGFR decline, outcome definition standard in CKD progression studies.	
**Concern that the outcome, its definition, assessment, or timing of assessment do not match the review question or the assessor's intended use**	**APPLICABILITY CONCERN:** ***low***/*high/unclear*
Matches clinical question of identifying boys at risk of CKD progression	

**DOMAIN 4: Analysis**	
**A. Quality**	
225 patients, 51 events (22.6%), maging branch high-dimensional ResNet-50 features on 225 patients	
RF, stratified 5-fold CV (80/20), identical splits across the three models, time-dependent covariates introduced at 6 mo/1 y/3 y/5 y, performance: concordance index (C-index) and AUROC at 2/5/10 y, sensitivity analyses: ≥35% eGFR decline and U25 CKiD equation.	
Exclusion: 312/537 (58%) of eligible PUV patients excluded for lack of US within 90 d or incomplete clinical data.	
Missing data: complete-case (inclusion required nadir creatinine in year 1).	
	**Y/ PY/ PN/ N/ NI**
**4.1 Was there evidence that the sample size was reasonable?**	PY
**4.2 Were continuous and categorical predictors handled appropriately?**	PY
**4.3 Were participants with missing or censored data handled appropriately in the analysis?**	PY
**4.4 If methods to address class imbalance were used, was the model or the model predictions recalibrated?***	NA
**4.5 Were methods used to address potential model overfitting?**	PY

Concern regarding quality of the analysis	QUALITYCONCERN: *low/high/**unclear***
Random survival forests with stratified 5-fold CV, identical splits across models, time-dependent covariates, sensitivity analyses on outcome definition and eGFR formula, only C-index and ROC reported, no calibration plot	

Model evaluation

Domain 1,2 and 3 same analysis as model development

**DOMAIN 4: Analysis**			
**Risk of bias**			
225 patients, 51 events; CV with 80/20 splits.			
AUROC and c-statistic reported, risk-stratification: 27/51 events in high-risk group vs 19/174 in low-risk (p < 0.01).			
Describe any participants who were excluded from the analysis: Exclusion same as development			
Describe missing data on predictors and outcomes as well as methods used for handling these missing data: Missing data same as development			
	**Y/ PY/ PN/ N/ NI/ NA**		
**4.1 Was model evaluation based on only apparent performance avoided?**	Y		
	**A**	**I**	**E**
**4.2 Was there evidence that the sample size was reasonable?**	PY	PY	NA
**4.3 Were participants with missing or censored data handled appropriately in the analysis?**	PY	PY	NA
**4.4 If methods to address class imbalance were used, was the evaluation done in a dataset without imbalance correction?***	NA	NA	NA
**4.5 If data splitting was done to create training and test datasets, was there evidence that data leakage was avoided?***	**NA**	PY	**NA**
**4.6 If resampling methods were used to evaluate model performance, were all model development steps replicated in the resampling process?***	**NA**	PY	**NA**
**4.7 Was the predictive performance of the model evaluated appropriately, e.g., calibration, discrimination, and net benefit?**	PN	PN	NA
**Risk of bias introduced by the analysis**	**RISK OF BIAS:** *low/**high**/unclear*		
Stratified CV with identical splits across models, Kaplan-Meier risk stratification, pre-specified sensitivity analyses, no calibration of survival predictions, no decision-curve analysis, absence of external validation			

Step 4 - Assess the overall concerns regarding quality, risk of bias and applicability of the prediction model

**OVERALL JUDGEMENT OF PREDICTION MODEL**	
**Overall judgement of quality (development)**	**QUALITY CONCERN:** *low/**high**/unclear*
High due to selection issues (D1) and unclear D4	
**Overall judgement of risk of bias (evaluation)**	**RISK OF BIAS:** *low/**high**/unclear*

No calibration analysis, no external validation, selection bias from inclusion requirements.	
**Overall judgement of applicability (development)**	**APPLICABILITY CONCERN:** *low/high/**unclear***
Two North-American tertiary centres; transportability to other settings unproven.	
**Overall judgement of applicability (evaluation)**	**APPLICABILITY CONCERN:** *low/high/**unclear***
Same as development	

Qin et al. (34)

Step 1 - PICOTS

**Population**	Adults with CKD undergoing right-kidney biopsy at a Chinese hospital
**Index model(s)**	ResNet-18-based multimodal deep-learning model fusing grey-scale US, superb microvascular imaging (SMI), and strain elastography (SE)
**Comparator model(s)**	Single-mode DL models (US, SMI, SE) and a clinical logistic regression model using eGFR and 24-h proteinuria.
**Outcome(s)**	Minimal (≤10%) vs mild (11–25%) IF/TA on biopsy, scored by two pathologists.
**Timing**	Cross-sectional
**Setting and intended use of the prediction model**	Hospital ultrasound clinic, non-invasive early-fibrosis screening tool to reduce repeat biopsies

Step 2 – Classify the type of prediction model assessment

**Classify the assessment based on its aim**			
Type of prediction study	***Explanation***	***PROBAST+AI boxes to complete***	***Tick as appropriate***
**Development only**	**Prediction model development only, i.e., without evaluation of its performance.**	**Model development**	
**Evaluation only**	**External validation of one or more existing models in new data**	**Model evaluation**	
**Combination**	**Prediction model development combined in the same study (publication) with the evaluation of its apparent performance, internal validation performance, or**	**Model development and evaluation**	**X**

	**external validation performance.**		

Step 3 - Assess quality and applicability or risk of bias and applicability

**Publication reference**	Qin X, Liu X, Xia L, Luo Q, Zhang C. Multimodal ultrasound deep learning to detect fibro-sis in early chronic kidney disease. Renal failure. 2024;46(2):2417740.
**Models of interest**	Multimodal US (US + SMI + SE) deep learning vs single-mode DL vs clinical model.
**Outcome of interest**	Minimal vs mild IF/TA on biopsy.

Model development

**DOMAIN 1: Participants and data sources**	
**A. Quality**	
Prospective single-centre cohort, 114 analysed, exclusions: kidney-skin >5 cm, breath-hold inability, cyst/tumour, no biopsy, poor images, missing clinical data, moderate-to-severe IF/TA (>25%) excluded by design.	
	**Y/ PY/ PN/ N/ NI**
**1.1 Were appropriate data sources used?**	PY
**1.2 Was an appropriate study design used?**	PY
**1.3 Did the in- and exclusions of study participants result in a representative dataset?**	PN
**Concern regarding quality of selection of participants and data sources**	**QUALITYCONCERN:** *low/**high**/unclear*
Rationale of quality rating: Prospective design with biopsy reference, excluding moderate-to-severe, only discriminating minimal vs mild, easier task than “detecting fibrosis”, n=114 is small for a deep-learning model.	
**B. Applicability**	
Single-center, small sample for DL, strict inclusion criteria	
**Concern that the (data of the) included participants do not match the review question or the assessor’s intended use of the prediction model**	**APPLICABILITY CONCERN:** *low/**high**/unclear*
Rationale of applicability rating: Exclusion >25% IF/TA limits applicability to early-CKD only, no application to broader CKD population.	

**DOMAIN 2: Predictors**
**A. Quality**

Three US modalities per patient acquired on the same Aplio i700 system: grey-scale US, SMI (Doppler-based microvasculature), and continuous strain elastography, manual segmentation by two experienced doctors	
	**Y/ PY/ PN/ N/ NI**
**2.1 Were predictors defined and assessed in a similar way for all participants?**	PY
**2.2 Was any pre-processing of predictors similar for all participants?**	PY
**2.3 Were predictor assessments made without knowledge of outcome data?**	NI
**2.4 Were the predictors included in the model available at the time the model was intended to be used?**	PY
**Concern regarding quality of selection of participants and data sources**	**QUALITYCONCERN:** *low/high/**unclear***
Multimodal uniform acquisition, manual segmentation by sonographers raises potential operator dependence, not stated whether segmentation was blinded	
**B. Applicability**	
**Concern that the definition, pre-processing, assessment, or timing of assessment of the predictors in the model do not match the review question or the assessor's intended use**	**APPLICABILITY CONCERN:** *low/high/**unclear***
Rationale of applicability rating: Single-scanner manual-ROI predictors and operator-dependent segmentation	

**DOMAIN 3: Outcome**	
**A. Quality**	
Composite CKD progression: IF/TA score (0–100%) by two renal pathologists dichotomised at ≤10% vs 11–25%.	
Or	**Y/ PY/ PN/ N/ NI**
**3.1 Were outcomes defined and assessed appropriately?**	Y
**3.2 Were outcomes defined and assessed in a similar way for all participants?**	Y
**3.3 Were outcome assessments made without use or knowledge of predictor data?**	PY
**3.4 Was the time interval between predictor assessment and outcome assessment appropriate?**	Y
**Concern regarding quality of the outcome or its determination**	**QUALITYCONCERN:** ***low**/high/unclear*
Biopsy is gold standard, semi-quantitative IF/TA grading by two pathologists, dichotomisation at 10% loses information but matches defensible boundary.	
**B. Applicability**	
Outcome determined on biopsy one day after US imaging, binary IF/TA ≤10% vs 11–25% directly matches target classification.	
**Concern that the outcome, its definition, assessment, or timing of assessment do not match the review question or the assessor's intended use**	**APPLICABILITY CONCERN:** ***low**/high/unclear*
Biopsy IF/TA matches the histological target of intended noninvasive test.	

DOMAIN 4: Analysis	
A. Quality	
114 patients; training 80 / test 34; 2,922 training images and 1,140 test images counted at the image level	
DeLong test to compare AUCs, DCA for clinical utility, SHAP for global modality importance, no cross-validation, no calibration plot, no confidence intervals on AUC, class imbalance not corrected.	
Exclusion: 29/143 excluded, mentioned above	
Missing data: complete-case	
	**Y/ PY/ PN/ N/ NI**
**4.1 Was there evidence that the sample size was reasonable?**	PN
**4.2 Were continuous and categorical predictors handled appropriately?**	PY
**4.3 Were participants with missing or censored data handled appropriately in the analysis?**	PY
**4.4 If methods to address class imbalance were used, was the model or the model predictions recalibrated?***	NA
**4.5 Were methods used to address potential model overfitting?**	PY
**Concern regarding quality of the analysis**	**QUALITYCONCERN:** *low/**high**/unclear*
Multimodal fusion, transfer learning, DeLong comparison, DCA and SHAP interpretation, 114 patients for a deep-learning model, no cross-validation, no calibration analysis, AUC differences vs single-mode and clinical models lean on 34-patient test set	

Model evaluation

Domain 1,2 and 3 same analysis as model development

DOMAIN 4: Analysis			
Risk of bias			
34-patient test set, 380 images per modality			
AUROC, accuracy, sensitivity, specificity, NPV, PPV, F1-score, Delong p-value, SHAP analysis, no calibration			
Describe any participants who were excluded from the analysis: Exclusion same as development			
Describe missing data on predictors and outcomes as well as methods used for handling these missing data: Missing data: complete case			
	**Y/ PY/ PN/ N/ NI/ NA**		
**4.1 Was model evaluation based on only apparent performance avoided?**	Y		
	**A**	**I**	**E**
**4.2 Was there evidence that the sample size was reasonable?**	PN	PN	NA
**4.3 Were participants with missing or censored data handled appropriately in the analysis?**	PY	PY	NA

**4.4 If methods to address class imbalance were used, was the evaluation done in a dataset without imbalance correction?***	NA	NA	NA
**4.5 If data splitting was done to create training and test datasets, was there evidence that data leakage was avoided?***	**NA**	PY	**NA**
**4.6 If resampling methods were used to evaluate model performance, were all model development steps replicated in the resampling process?***	**NA**	NA	**NA**
**4.7 Was the predictive performance of the model evaluated appropriately, e.g., calibration, discrimination, and net benefit?**	PN	PN	NA
**Risk of bias introduced by the analysis**	**RISK OF BIAS:** *low/**high**/unclear*		
DeLong comparison, DCA, SHAP interpretation, comparison with single-mode and clinical baselines. test set is only 34 patients, no cross-validation or confidence intervals, no calibration, no external validation			

Step 4 - Assess the overall concerns regarding quality, risk of bias and applicability of the prediction model

**OVERALL JUDGEMENT OF PREDICTION MODEL**	
**Overall judgement of quality (development)**	**QUALITY CONCERN:** *low/**high**/unclear*
High concern in D1 and D4	
**Overall judgement of risk of bias (evaluation)**	**RISK OF BIAS:** *low/**high**/unclear*
34-patient hold-out test, no CV, no external validation, no calibration	
**Overall judgement of applicability (development)**	**APPLICABILITY CONCERN:** *low/**high**/unclear*
Single-centre, single scanner, manual ROI, early-fibrosis-only.	
**Overall judgement of applicability (evaluation)**	**APPLICABILITY CONCERN:** *low/**high**/unclear*
Same as development	

Chang et al. (35)

Step 1 - PICOTS

**Population**	Adults with CKD undergoing pre-biopsy renal US at multiple hospitals in Taipei
**Index model(s)**	Multimodal: DPCNN (2 048-d feature vector) on Mask R-CNN-segmented kidney ROIs concatenated with 5 biomarkers, three classifiers; XGBoost, RF, LR
**Comparator model(s)**	eGFR-only threshold,biomarkers only, image features only
**Outcome(s)**	IFTA score 0–3 dichotomised: mild (0–1, ≤25 %) vs severe (2–3, >25 %).
**Timing**	Retrospective. US within ±3 months of biopsy.

**Setting and intended use of the prediction model**	Three Taipei teaching hospitals; intended non-invasive triage of CKD patients before / instead of biopsy.

Step 2 – Classify the type of prediction model assessment

**Classify the assessment based on its aim**			
Type of prediction study	***Explanation***	***PROBAST+AI boxes to complete***	***Tick as appropriate***
**Development only**	**Prediction model development only, i.e., without evaluation of its performance.**	**Model development**	
**Evaluation only**	**External validation of one or more existing models in new data**	**Model evaluation**	
**Combination**	**Prediction model development combined in the same study (publication) with the evaluation of its apparent performance, internal validation performance, or external validation performance.**	**Model development and evaluation**	**X**

Step 3 - Assess quality and applicability or risk of bias and applicability

**Publication reference**	Chang TW, Tsai CY, Tang ZY, Zheng CM, Liao CT, Cheng CY, et al. Artificial intelligence for predicting interstitial fibrosis and tubular atrophy using diagnostic ultrasound imag-ing and biomarkers. BMJ Health Care Inform. 2025;32(1).
**Models of interest**	LR, XGBoost, RF
**Outcome of interest**	Mild (0–1, ≤25 %) vs severe (2–3, >25 %).

Model development

**DOMAIN 1: Participants and data sources**	
**A. Quality**	
Retrospective multicentre cohort (three Taipei hospitals, 2014–2024), 223 patients / 1 576 images with complete biomarkers entered ML modelling (training 171 / test 52), 129 patients IFTA 0–25 % and 94 patients IFTA > 25 %.	
	**Y/ PY/ PN/ N/ NI**
**1.1 Were appropriate data sources used?**	PY
**1.2 Was an appropriate study design used?**	PY
**1.3 Did the in- and exclusions of study participants result in a representative dataset?**	PN

**Concern regarding quality of selection of participants and data sources**	**QUALITYCONCERN:** *low/**high**/unclear*
Rationale of quality rating: Large, multicentre cohort, heavy, manual image-quality filtering, exclusion of incomplete-biomarker patients, retrospective design with consent waiver, no external validation	
**B. Applicability**	
Three Taipei teaching hospitals, Taiwan, retrospective cohort, final ML cohort 223 patients, diabetic-nephropathy patients excluded by design	
**Concern that the (data of the) included participants do not match the review question or the assessor's intended use of the prediction model**	**APPLICABILITY CONCERN:** *low/**high**/unclear*
Rationale of applicability rating: Excluding diabetic nephropathy narrows the applicable population, single-region Taiwanese ethnicity	

**DOMAIN 2: Predictors**	
**A. Quality**	
Predictors: 2 048-d image feature vector from a dual-path CNN trained on Mask R-CNN-segmented kidney ROIs, 5 biomarkers: age, sex, eGFR, serum albumin, US-measured kidney size.	
	**Y/ PY/ PN/ N/ NI**
**2.1 Were predictors defined and assessed in a similar way for all participants?**	Y
**2.2 Was any pre-processing of predictors similar for all participants?**	Y
**2.3 Were predictor assessments made without knowledge of outcome data?**	PY
**2.4 Were the predictors included in the model available at the time the model was intended to be used?**	Y
**Concern regarding quality of selection of participants and data sources**	**QUALITYCONCERN:** ***low**/high/unclear*
Uniform automated pipeline for image segmentation and feature extraction, standard biomarkers, minor residual concern about possible overlap between Mask R-CNN training images and the downstream ML training set	
**B. Applicability**	
**Concern that the definition, pre-processing, assessment, or timing of assessment of the predictors in the model do not match the review question or the assessor's intended use**	**APPLICABILITY CONCERN:** ***low**/high/unclear*
Rationale of applicability rating: Biomarkers are routinely available, the DPCNN pipeline requires identical implementation and is a deployment dependency	

**DOMAIN 3: Outcome**	
**A. Quality**	
IFTA score (0/1/2/3) from the local pathology report closest to US date, dichotomised mild (0–1, 0–25 %) vs severe (2–3, >25 %), no central re-read of biopsies, each centre's local pathology guidelines used	
Or	**Y/ PY/ PN/ N/ NI**
**3.1 Were outcomes defined and assessed appropriately?**	PY
**3.2 Were outcomes defined and assessed in a similar way for all participants?**	PN

**3.3 Were outcome assessments made without use or knowledge of predictor data?**	PY
**3.4 Was the time interval between predictor assessment and outcome assessment appropriate?**	PY
**Concern regarding quality of the outcome or its determination**	**QUALITYCONCERN:** *low/**high**/unclear*
No standardised pathology re-read across the three centres, inter-observer variability in IFTA scoring is well-known and not mitigated, US-biopsy interval up to 3 months	
**B. Applicability**	
Binary IFTA outcome (≤25 % vs >25 %), biopsy and US up to 3 months apart.	
**Concern that the outcome, its definition, assessment, or timing of assessment do not match the review question or the assessor's intended use**	**APPLICABILITY CONCERN:** ***low**/high/unclear*
Outcome and dichotomisation match clinically meaningful threshold for intended decision.	

**DOMAIN 4: Analysis**	
**A. Quality**	
223 patients / 1 576 images in the ML cohort (training 171 / test 52). 5 biomarker inputs + 2 048-d image feature vector.	
three classifiers (XGBoost, RF, logistic), three inputs (BM5, F2048, F2048+BM5), 5-fold cross-validation, DeLong tests for AUC comparisons.	
AUROC, accuracy, precision, recall, F1. No calibration plot, no DCA, no 95 % CIs.	
Missing data: complete-case	
	**Y/ PY/ PN/ N/ NI**
**4.1 Was there evidence that the sample size was reasonable?**	PY
**4.2 Were continuous and categorical predictors handled appropriately?**	PY
**4.3 Were participants with missing or censored data handled appropriately in the analysis?**	N
**4.4 If methods to address class imbalance were used, was the model or the model predictions recalibrated?***	PY
**4.5 Were methods used to address potential model overfitting?**	PY
**Concern regarding quality of the analysis**	**QUALITYCONCERN:** *low/**high**/unclear*
5-fold CV, well-defined image- vs patient-level evaluation, DeLong comparison across feature sets, 52-patient test set, complete-case exclusion of incomplete-biomarker patients, no calibration/DCA/external validation, and potential overlap between Mask R-CNN and ML training images	

Model evaluation

Domain 1,2 and 3 same analysis as model development

**DOMAIN 4: Analysis**
**Risk of bias**

patient-level n = 52, image-level n = 384.			
AUROC, accuracy, precision, recall, F1-score, DeLong value			
Describe any participants who were excluded from the analysis: Exclusion same as development			
Describe missing data on predictors and outcomes as well as methods used for handling these missing data: Missing data: complete case			
	**Y/ PY/ PN/ N/ NI/ NA**		
**4.1 Was model evaluation based on only apparent performance avoided?**	Y		
	**A**	**I**	**E**
**4.2 Was there evidence that the sample size was reasonable?**	PN	PN	NA
**4.3 Were participants with missing or censored data handled appropriately in the analysis?**	N	N	NA
**4.4 If methods to address class imbalance were used, was the evaluation done in a dataset without imbalance correction?***	PY	PY	NA
**4.5 If data splitting was done to create training and test datasets, was there evidence that data leakage was avoided?***	**NA**	PY	**NA**
**4.6 If resampling methods were used to evaluate model performance, were all model development steps replicated in the resampling process?***	**NA**	PY	**NA**
**4.7 Was the predictive performance of the model evaluated appropriately, e.g., calibration, discrimination, and net benefit?**	PN	PN	NA
**Risk of bias introduced by the analysis**	**RISK OF BIAS:** *low/**high**/unclear*		
DeLong comparison, DCA, SHAP interpretation, comparison with single-mode and clinical baselines. test set is only 34 patients, no cross-validation or confidence intervals, no calibration, no external validation			

Step 4 - Assess the overall concerns regarding quality, risk of bias and applicability of the prediction model

**OVERALL JUDGEMENT OF PREDICTION MODEL**	
**Overall judgement of quality (development)**	**QUALITY CONCERN:** *low/**high**/unclear*
High concern in D1, D2 and D4	
**Overall judgement of risk of bias (evaluation)**	**RISK OF BIAS:** *low/**high**/unclear*
5-fold CV + 52-patient test, no external validation, non-standardised pathology reference.	
**Overall judgement of applicability (development)**	**APPLICABILITY CONCERN:** *low/**high**/unclear*
Diabetic-nephropathy patients excluded, single-region Taiwanese cohort.	
**Overall judgement of applicability (evaluation)**	**APPLICABILITY CONCERN:** *low/**high**/unclear*
Same as development	

Zhu et al. (36)

Step 1 - PICOTS

**Population**	Adults with CKD at a Chinese hospital, with a clinical indication for native kidney biopsy
**Index model(s)**	SVM combining SWE + B-mode CDFI. Random-forest used for feature importance.
**Comparator model(s)**	B-mode + CDFI alone, SWE alone, eGFR, ACR,
**Outcome(s)**	Semi-quantitative IFTA area graded 0/1/2/3 (≤10 % / 11–25 % / 26–50 % / >50 %); multiple binary tasks
**Timing**	Cross-sectional. Ultrasound, eGFR and biopsy on the same day.
**Setting and intended use of the prediction model**	Tertiary nephrology centre, Shanghai, non-invasive triage of CKD patients to monitor and stratify fibrosis without repeated biopsy

Step 2 – Classify the type of prediction model assessment

**Classify the assessment based on its aim**			
Type of prediction study	***Explanation***	***PROBAST+AI boxes to complete***	***Tick as appropriate***
**Development only**	**Prediction model development only, i.e., without evaluation of its performance.**	**Model development**	
**Evaluation only**	**External validation of one or more existing models in new data**	**Model evaluation**	
**Combination**	**Prediction model development combined in the same study (publication) with the evaluation of its apparent performance, internal validation performance, or external validation performance.**	**Model development and evaluation**	**X**

Step 3 - Assess quality and applicability or risk of bias and applicability

**Publication reference**	Zhu M, Ma L, Yang W, Tang L, Li H, Zheng M, et al. Elastography ultrasound with ma-chine learning improves the diagnostic performance of traditional ultrasound in predict-ing kidney fibrosis. J Formos Med Assoc. 2022;121(6):1062–72.

**Models of interest**	SVM with SWE + B-mode + CDFI features vs comparator models above.
**Outcome of interest**	Four-grade tubulointerstitial fibrosis dichotomised in different ways

Model development

**DOMAIN 1: Participants and data sources**	
**A. Quality**	
Prospective single-centre cohort, 117 analysed, exclusions: inability to breath-hold for SWE, no corticomedullary differentiation on B-mode, poorly filled SWE box, contraindications to puncture, severe hypoxia, pregnancy, asymmetry, structural abnormality of left kidney.	
	**Y/ PY/ PN/ N/ NI**
**1.1 Were appropriate data sources used?**	PY
**1.2 Was an appropriate study design used?**	Y
**1.3 Did the in- and exclusions of study participants result in a representative dataset?**	PN
**Concern regarding quality of selection of participants and data sources**	**QUALITYCONCERN:** *low/**high**/unclear*
Rationale of quality rating: Source population and prospective cross-sectional design are appropriate, 91 patients excluded mostly for technical SWE reasons, biasing the dataset, cohort imbalanced	
**B. Applicability**	
Single tertiary nephrology centre, adult Chinese CKD patients, IFTA grades: none, mild, moderate, severe, imaging platform: Supersonic Aixplorer with SC6-1 convex transducer.	
**Concern that the (data of the) included participants do not match the review question or the assessor's intended use of the prediction model**	**APPLICABILITY CONCERN:** *low/**high**/unclear*
Rationale of applicability rating: Population is biopsy-eligible Chinese tertiary-care patients, generalisability to community CKD limited	

**DOMAIN 2: Predictors**	
**A. Quality**	
age, sex, BMI, history of T2DM and hypertension, left-kidney SWE, CDFI Vmax, end-diastolic velocity and RI in interlobar and segmental arteries of both kidneys, left-kidney B-mode length, width, thickness, all acquired by one experienced operator on the day of biops, random-forest used for unsupervised variable-importance screening	
	**Y/ PY/ PN/ N/ NI**
**2.1 Were predictors defined and assessed in a similar way for all participants?**	Y
**2.2 Was any pre-processing of predictors similar for all participants?**	Y
**2.3 Were predictor assessments made without knowledge of outcome data?**	Y
**2.4 Were the predictors included in the model available at the time the model was intended to be used?**	Y
**Concern regarding quality of selection of participants and data sources**	**QUALITYCONCERN:** ***low**/high/unclear*

Standardised acquisition by single operator blinded to the histological diagnosis, single-operator design limits inter-observer assessment but eliminates inter-operator variability

**B. Applicability**	
**Concern that the definition, pre-processing, assessment, or timing of assessment of the predictors in the model do not match the review question or the assessor's intended use**	**APPLICABILITY CONCERN:** ***low****/high/unclear*
Rationale of applicability rating: SWE + B-mode + CDFI routinely obtainable in renal ultrasound workflows.	

**DOMAIN 3: Outcome**	
**A. Quality**	
IFTA area on left kidney, grades 0 (≤10 %), 1 (11–25 %), 2 (26–50 %), 3 (>50 %), predictor (SWE) and outcome (biopsy) obtained on the same day.	
Or	**Y/ PY/ PN/ N/ NI**
**3.1 Were outcomes defined and assessed appropriately?**	Y
**3.2 Were outcomes defined and assessed in a similar way for all participants?**	Y
**3.3 Were outcome assessments made without use or knowledge of predictor data?**	Y
**3.4 Was the time interval between predictor assessment and outcome assessment appropriate?**	Y
**Concern regarding quality of the outcome or its determination**	**QUALITYCONCERN:** ***low****/high/unclear*
Outcome determined on same-day biopsy, composite four-grade IFTA outcome dichotomised in multiple ways.	
**B. Applicability**	
Binary IFTA outcome (≤25 % vs >25 %), biopsy and US up to 3 months apart.	
**Concern that the outcome, its definition, assessment, or timing of assessment do not match the review question or the assessor's intended use**	**APPLICABILITY CONCERN:** ***low****/high/unclear*
Histological IFTA grade directly matches clinical question and intended use.	

**DOMAIN 4: Analysis**
**A. Quality**
n = 117 patients, after RF screening candidate predictors reduced to a smaller subset (left-kidney cortical SWE, sinus SWE, segmental-artery flow indices, T2DM, left-kidney length).
SVM and 8/2 train/test split, 5-fold cross-validation within training, DeLong test for AUC comparisons.
AUC, sensitivity, specificity, accuracy, F1, no calibration, no devision-curve analysis

Missing data: complete-case analysis, many screened patients excluded prior to modelling (mostly for SWE technical failure).

	Y/ PY/ PN/ N/ NI
**4.1 Was there evidence that the sample size was reasonable?**	PN
**4.2 Were continuous and categorical predictors handled appropriately?**	PY
**4.3 Were participants with missing or censored data handled appropriately in the analysis?**	NI
**4.4 If methods to address class imbalance were used, was the model or the model predictions recalibrated?***	N
**4.5 Were methods used to address potential model overfitting?**	PY
**Concern regarding quality of the analysis**	**QUALITYCONCERN:** *low/**high**/unclear*
Small sample size relative to candidate predictors, class imbalance was not addressed, discrimination-only reporting, 5-fold CV mitigates overfitting risk	

Model evaluation

Domain 1,2 and 3 same analysis as model development

**DOMAIN 4: Analysis**			
**Risk of bias**			
20 % test set, with few moderate and severe IFTA cases in the test split.			
AUROC, accuracy, sensitivity, specificity, precision, F1-score			
Describe any participants who were excluded from the analysis: Exclusion same as development			
Describe missing data on predictors and outcomes as well as methods used for handling these missing data: Missing data: complete case			
	**Y/ PY/ PN/ N/ NI/ NA**		
**4.1 Was model evaluation based on only apparent performance avoided?**	Y		
	**A**	**I**	**E**
**4.2 Was there evidence that the sample size was reasonable?**	PN	PN	NA
**4.3 Were participants with missing or censored data handled appropriately in the analysis?**	PY	PY	NA
**4.4 If methods to address class imbalance were used, was the evaluation done in a dataset without imbalance correction?***	NA	NA	NA
**4.5 If data splitting was done to create training and test datasets, was there evidence that data leakage was avoided?***	**NA**	PY	**NA**
**4.6 If resampling methods were used to evaluate model performance, were all model development steps replicated in the resampling process?***	**NA**	PY	**NA**
**4.7 Was the predictive performance of the model evaluated appropriately, e.g., calibration, discrimination, and net benefit?**	PN	PN	NA
**Risk of bias introduced by the analysis**	**RISK OF BIAS:** *low/**high**/unclear*		

5-fold CV and a held-out test set are good practice, test set is small, few high-grade events, no calibration/decision-curve analysis, no external validation, no confidence intervals around the AUROC

Step 4 - Assess the overall concerns regarding quality, risk of bias and applicability of the prediction model

OVERALL JUDGEMENT OF PREDICTION MODEL	
**Overall judgement of quality (development)**	**QUALITY CONCERN:** *low/**high**/unclear*
High concern in D1 and D4, selection bias, no calibration	
**Overall judgement of risk of bias (evaluation)**	**RISK OF BIAS:** *low/**high**/unclear*
Internal hold-out + 5-fold CV only, small test set, no calibration, no external validation.	
**Overall judgement of applicability (development)**	**APPLICABILITY CONCERN:** *low/**high**/unclear*
Single Chinese tertiary centre, biopsy-eligible CKD with adequate ultrasound windows, restricted to glomerular-disease-dominated cohort.	
**Overall judgement of applicability (evaluation)**	**APPLICABILITY CONCERN:** *low/**high**/unclear*
Same as development	

Chen et al. (37)

Step 1 - PICOTS

**Population**	Adults with CKD undergoing 2D-SWE and renal biopsy at a Chinese hospital
**Index model(s)**	MLP
**Comparator model(s)**	Logistic regression with the same inputs
**Outcome(s)**	Composite semi-quantitative pathology score dichotomised: mild (9 pts) vs moderate–severe (≥10 pts).
**Timing**	Cross-sectional.
**Setting and intended use of the prediction model**	Tertiary nephrology centre, China, intended non-invasive risk stratification of CKD severity to support clinical decisions.

Step 2 – Classify the type of prediction model assessment

**Classify the assessment based on its aim**			
Type of prediction study	***Explanation***	***PROBAST+AI boxes to complete***	***Tick as appropriate***
**Development only**	**Prediction model development only, i.e., without evaluation of its performance.**	**Model development**	
**Evaluation only**	**External validation of one or more existing models in new data**	**Model evaluation**	
**Combination**	**Prediction model development combined in the same study (publication) with the evaluation of its apparent performance, internal validation performance, or external validation performance.**	**Model development and evaluation**	**X**

Step 3 - Assess quality and applicability or risk of bias and applicability

**Publication reference**	Chen ZM, Ying TC, Chen JX, Wu CQ, Li LJ, Chen H, et al. Using elastography-based mul-tilayer perceptron model to evaluate renal fibrosis in chronic kidney disease. Renal Fail-ure. 2023;45(1).
**Models of interest**	MLP
**Outcome of interest**	Mild vs moderate–severe composite renal pathology score.

Model development

DOMAIN 1: Participants and data sources	
**A. Quality**	
Prospective single-centre cross-sectional cohort, 7/3 training/testing, exclusions: multiple renal cysts / nephrolithiasis / hydronephrosis / masses interfering with 2D-SWE, failure to control breathing, technical 2D-SWE failure (obesity, mental tension), insufficient biopsy tissue.	
	**Y/ PY/ PN/ N/ NI**
**1.1 Were appropriate data sources used?**	PY
**1.2 Was an appropriate study design used?**	Y
**1.3 Did the in- and exclusions of study participants result in a representative dataset?**	PN
**Concern regarding quality of selection of participants and data sources**	**QUALITYCONCERN:** *low/**high**/unclear*
Rationale of quality rating: Source population and prospective design appropriate, multiple exclusions for technical 2D-SWE failure, exact number of screened patients not reported, IgA-nephropathy-dominated cohort	
**B. Applicability**	
Single tertiary radiology / nephrology centre in China, 162 adult CKD patients recruited April 2019 – December 2021, imaging platform: Supersonic Aixplorer with SC6-1 convex transducer.	
**Concern that the (data of the) included participants do not match the review question or the assessor's intended use of the prediction model**	**APPLICABILITY CONCERN:** *low/**high**/unclear*
Rationale of applicability rating: Young and high eGFR cohort, single Chinese tertiary centre, few severe cases, generalisability uncertain	

DOMAIN 2: Predictors	
**A. Quality**	
2D-SWE, five independent valid measurements averaged,;<br>conventional US: renal length (upper-to-lower pole), parenchymal thickness (renal margin to sinus), inter-lobar artery RI; clinical: age, sex, BMI; biopsy biomarkers BUN, creatinine, uric acid, albumin, ACR; eGFR by CKD-EPI; comorbidities (diabetes / hypertension / CVD).	
	**Y/ PY/ PN/ N/ NI**
**2.1 Were predictors defined and assessed in a similar way for all participants?**	Y
**2.2 Was any pre-processing of predictors similar for all participants?**	Y
**2.3 Were predictor assessments made without knowledge of outcome data?**	Y
**2.4 Were the predictors included in the model available at the time the model was intended to be used?**	Y
**Concern regarding quality of selection of participants and data sources**	**QUALITYCONCERN:** ***low**/high/unclear*
Standardised, blinded predictor pipeline with quality-control on weak/no signal frames, single experienced radiologist	
**B. Applicability**	
**Concern that the definition, pre-processing, assessment, or timing of assessment of the predictors in the model do not match the review question or the assessor's intended use**	**APPLICABILITY CONCERN:** ***low**/high/unclear*

Rationale of applicability rating: Routine clinical predictors with broad applicability across nephrology and radiology workflows.

DOMAIN 3: Outcome	
**A. Quality**	
Outcome scored by two pathologists using a published semi-quantitative system, blinded to clinical and US data, disagreements resolved by negotiation, severe combined with moderate for binary outcome, 2D-SWE within 2 days before biopsy.	
Or	**Y/ PY/ PN/ N/ NI**
**3.1 Were outcomes defined and assessed appropriately?**	Y
**3.2 Were outcomes defined and assessed in a similar way for all participants?**	Y
**3.3 Were outcome assessments made without use or knowledge of predictor data?**	Y
**3.4 Was the time interval between predictor assessment and outcome assessment appropriate?**	Y
**Concern regarding quality of the outcome or its determination**	**QUALITYCONCERN:** ***low****/high/unclear*
Standard histological reference; blinded pathology; clinically appropriate predictor–outcome interval.	
**B. Applicability**	
Binary mild vs moderate–severe, clinically meaningful decision boundary for treatment intensification and prognostic counselling.	
**Concern that the outcome, its definition, assessment, or timing of assessment do not match the review question or the assessor's intended use**	**APPLICABILITY CONCERN:** ***low****/high/unclear*
Histological reference standard matches clinical question.	

DOMAIN 4: Analysis	
**A. Quality**	
n = 162 (training 114/test 48), 22 input variables,2 output probabilities.	
MLP and 7/3 train/test split, compared to LR	
AUROC, sensitivity, specificity, accuracy, calibration, clinical utility	
Missing data: not explicitly described	
	**Y/ PY/ PN/ N/ NI**
**4.1 Was there evidence that the sample size was reasonable?**	PN
**4.2 Were continuous and categorical predictors handled appropriately?**	PY
**4.3 Were participants with missing or censored data handled appropriately in the analysis?**	NI

**4.4 If methods to address class imbalance were used, was the model or the model predictions recalibrated?***	N
**4.5 Were methods used to address potential model overfitting?**	PY
**Concern regarding quality of the analysis**	**QUALITYCONCERN:** *low/**high**/unclear*
Reporting of calibration, DCA and clinical-impact curve, explicit comparison with logistic regression, reliance on a single random 7:3 split, no cross-validation, no external validation, no missing-data description.	

Model evaluation

Domain 1,2 and 3 same analysis as model development

**DOMAIN 4: Analysis**			
**Risk of bias**			
30 % test set, n = 48 (24 mild / 24 moderate-severe).			
AUROC, sensitivity, specificity, accuracy, calibration, clinical utility			
Describe any participants who were excluded from the analysis: Exclusion same as development			
Describe missing data on predictors and outcomes as well as methods used for handling these missing data: Missing data: not mentioned			
	**Y/ PY/ PN/ N/ NI/ NA**		
**4.1 Was model evaluation based on only apparent performance avoided?**	Y		
	**A**	**I**	**E**
**4.2 Was there evidence that the sample size was reasonable?**	PN	PN	NA
**4.3 Were participants with missing or censored data handled appropriately in the analysis?**	PY	PY	NA
**4.4 If methods to address class imbalance were used, was the evaluation done in a dataset without imbalance correction?***	NA	NA	NA
**4.5 If data splitting was done to create training and test datasets, was there evidence that data leakage was avoided?***	**NA**	PY	**NA**
**4.6 If resampling methods were used to evaluate model performance, were all model development steps replicated in the resampling process?***	**NA**	NA	**NA**
**4.7 Was the predictive performance of the model evaluated appropriately, e.g., calibration, discrimination, and net benefit?**	Y	Y	NA
**Risk of bias introduced by the analysis**	**RISK OF BIAS:** *low/**high**/unclear*		
Reporting of calibration and clinical utility on the test set, small test set supplementary results show, no bootstrap CIs around AUROC, no cross-validation, no external validation.			

Step 4 - Assess the overall concerns regarding quality, risk of bias and applicability of the prediction model

OVERALL JUDGEMENT OF PREDICTION MODEL	
**Overall judgement of quality (development)**	**QUALITY CONCERN:**<br>*low/**high**/unclear*
High concern in D1 and D4	
**Overall judgement of risk of bias (evaluation)**	**RISK OF BIAS:**<br>*low/**high**/unclear*
Single random 7/3 split, test = 48, no cross-validation, no external validation.	
**Overall judgement of applicability (development)**	**APPLICABILITY CONCERN:**<br>*low/**high**/unclear*
Young, glomerular-disease-dominated, single-centre Chinese cohort.	
**Overall judgement of applicability (evaluation)**	**APPLICABILITY CONCERN:**<br>*low/**high**/unclear*
Same as development	

Chen et al. (38)

Step 1 - PICOTS

**Population**	Single-centre Chinese CKD cohort, n = 162
**Index model(s)**	XGBoost integrating five final predictors: SWE Emax, renal length, renal RI, hypertension, eGFR
**Comparator model(s)**	SVM, LightGBM, KNN
**Outcome(s)**	Mild vs moderate–severe based on semi-quantitative renal pathology score
**Timing**	Cross-sectional
**Setting and intended use of the prediction model**	Tertiary nephrology centre, China, intended interpretable non-invasive triage of CKD fibrosis severity.

Step 2 – Classify the type of prediction model assessment

Classify the assessment based on its aim			
Type of prediction study	***Explanation***	***PROBAST+AI boxes to complete***	***Tick as appropriate***
**Development only**	**Prediction model development only, i.e., without evaluation of its performance.**	**Model development**	
**Evaluation only**	**External validation of one or more existing models in new data**	**Model evaluation**	

**Combination**	**Prediction model development combined in the same study (publication) with the evaluation of its apparent performance, internal validation performance, or external validation performance.**	**Model development and evaluation**	**X**

Step 3 - Assess quality and applicability or risk of bias and applicability

**Publication reference**	Chen Z, Wang Y, Ying MTC, Su Z. Interpretable machine learning model integrating clin-ical and elastosonographic features to detect renal fibrosis in Asian patients with chronic kidney disease. Journal of nephrology. 2024;37(4):1027–39.
**Models of interest**	XGBoost/SVM/KNN/LightGBM
**Outcome of interest**	Mild vs moderate–severe composite renal pathology score.

Model development

**DOMAIN 1: Participants and data sources**	
**A. Quality**	
Prospective single-centre Asian CKD cohort, 162 patients (74 mild, 88 moderate–severe) recruited April 2019 – December 2021, exclusion criteria: cysts/masses, breath-control failure, technical SWE failure, insufficient biopsy, 5-fold CV across the entire cohort; no separate test cohort	
	**Y/ PY/ PN/ N/ NI**
**1.1 Were appropriate data sources used?**	PY
**1.2 Was an appropriate study design used?**	Y
**1.3 Did the in- and exclusions of study participants result in a representative dataset?**	PN
**Concern regarding quality of selection of participants and data sources**	**QUALITYCONCERN:** *low/**high**/unclear*
Rationale of quality rating: Selection bias based on exclusion/disease-spectrum, no external evaluation cohort, 5-fold CV, single-centre	
**B. Applicability**	
Single Chinese tertiary-care 162 adult CKD patients (April 2019 – December 2021); 74 mild) and 88 moderate–severe fibrosis, imaging platform: Supersonic Aixplorer with SC6-1 convex transducer.	
**Concern that the (data of the) included participants do not match the review question or the assessor's intended use of the prediction model**	**APPLICABILITY CONCERN:** *low/**high**/unclear*
Rationale of applicability rating: Asian, glomerular-disease-dominated, biopsy-eligible CKD cohort, IgA-nephropathy-heavy, limits extrapolation to more general CKD population	

**DOMAIN 2: Predictors**
**A. Quality**

Final five-feature model selected by univariable + multivariable logistic regression: Emax, renal length, RI, hypertension, eGFR.	
	**Y/ PY/ PN/ N/ NI**
**2.1 Were predictors defined and assessed in a similar way for all participants?**	Y
**2.2 Was any pre-processing of predictors similar for all participants?**	Y
**2.3 Were predictor assessments made without knowledge of outcome data?**	Y
**2.4 Were the predictors included in the model available at the time the model was intended to be used?**	Y
**Concern regarding quality of selection of participants and data sources**	**QUALITYCONCERN:** ***low***/*high/unclear*
Standardised blinded predictor acquisition, final model uses widely-available variables	
**B. Applicability**	
**Concern that the definition, pre-processing, assessment, or timing of assessment of the predictors in the model do not match the review question or the assessor's intended use**	**APPLICABILITY CONCERN:** ***low***/*high/unclear*
Rationale of applicability rating: Five-feature final model uses routinely-available US and clinical variables.	

**DOMAIN 3: Outcome**	
**A. Quality**	
Composite semi-quantitative score, blinded two-pathologist consensus, dichotomised mild vs moderate–severe.	
Or	**Y/ PY/ PN/ N/ NI**
**3.1 Were outcomes defined and assessed appropriately?**	Y
**3.2 Were outcomes defined and assessed in a similar way for all participants?**	Y
**3.3 Were outcome assessments made without use or knowledge of predictor data?**	Y
**3.4 Was the time interval between predictor assessment and outcome assessment appropriate?**	Y
**Concern regarding quality of the outcome or its determination**	**QUALITYCONCERN:** ***low***/*high/unclear*
Standard histological reference, blinded pathology, clinically appropriate predictor–outcome interval.	
**B. Applicability**	
Binary mild vs moderate–severe, matches intended clinical decision boundary.	
**Concern that the outcome, its definition, assessment, or timing of assessment do not match the review question or the assessor's intended use**	**APPLICABILITY CONCERN:** ***low***/*high/unclear*
Outcome and timing match intended use.	

DOMAIN 4: Analysis	
A. Quality	
n = 162, 5 final predictors after univariable + multivariable selection from 18 candidate variables.	
Four ML algorithms (XGBoost, SVM, LightGBM, KNN), 5-fold cross-validation as the primary validation strategy, SHAP for global feature importance evaluation.	
AUROC/average precision/sensitivity /specificity/accuracy/ F1/CI, calibration not reported, DCA not reported.	
Missing data: not explicitly described	
	**Y/ PY/ PN/ N/ NI**
**4.1 Was there evidence that the sample size was reasonable?**	PN
**4.2 Were continuous and categorical predictors handled appropriately?**	PY
**4.3 Were participants with missing or censored data handled appropriately in the analysis?**	NI
**4.4 If methods to address class imbalance were used, was the model or the model predictions recalibrated?***	N
**4.5 Were methods used to address potential model overfitting?**	PY
**Concern regarding quality of the analysis**	**QUALITYCONCERN:** *low/**high**/unclear*
Comparison across four ML algorithms, SHAP-based interpretability, 5-fold CV, CIs on AUROCs, no truly external validation, no calibration	

Model evaluation

Domain 1,2 and 3 same analysis as model development

DOMAIN 4: Analysis			
Risk of bias			
5-fold CV, 162 patients, 88 moderate-severe fibrosis			
AUROC, sensitivity, specificity, accuracy, average precision, SHAP-analysis			
Describe any participants who were excluded from the analysis:<br>Exclusion same as development			
Describe missing data on predictors and outcomes as well as methods used for handling these missing data:<br>Missing data: not explicitly described			
	**Y/ PY/ PN/ N/ NI/ NA**		
**4.1 Was model evaluation based on only apparent performance avoided?**	Y		
	**A**	**I**	**E**
**4.2 Was there evidence that the sample size was reasonable?**	PY	PY	NA
**4.3 Were participants with missing or censored data handled appropriately in the analysis?**	PY	PY	NA

**4.4 If methods to address class imbalance were used, was the evaluation done in a dataset without imbalance correction?***	NA	NA	NA
**4.5 If data splitting was done to create training and test datasets, was there evidence that data leakage was avoided?***	**NA**	PY	**NA**
**4.6 If resampling methods were used to evaluate model performance, were all model development steps replicated in the resampling process?***	**NA**	PY	**NA**
**4.7 Was the predictive performance of the model evaluated appropriately, e.g., calibration, discrimination, and net benefit?**	PN	PN	NA
**Risk of bias introduced by the analysis**	**RISK OF BIAS:** *low/**high**/unclear*		
5-fold CV, CIs around AUROCs, no calibration / DCA, no external validation, SHAP-analysis			

Step 4 - Assess the overall concerns regarding quality, risk of bias and applicability of the prediction model

**OVERALL JUDGEMENT OF PREDICTION MODEL**	
**Overall judgement of quality (development)**	**QUALITY CONCERN:** *low/**high**/unclear*
High concern in D1 and D4	
**Overall judgement of risk of bias (evaluation)**	**RISK OF BIAS:** *low/**high**/unclear*
5-fold CV only, no external validation, calibration not reported.	
**Overall judgement of applicability (development)**	**APPLICABILITY CONCERN:** *low/**high**/unclear*
Young, glomerular-disease-dominated, single-centre Chinese cohort.	
**Overall judgement of applicability (evaluation)**	**APPLICABILITY CONCERN:** *low/**high**/unclear*
Same as development	

Yoruk et al. (39)

Step 1 - PICOTS

**Population**	26 paediatric subjects, referred for contrast-enhanced MR urography at Stanford
**Index model(s)**	Pipeline: medulla-score map, whole-parenchyma segmentation, random-forest voxel classifier
**Comparator model(s)**	SVM-rbf/Adaboost
**Outcome(s)**	Single-kidney GFR (mL/min) and right-kidney split function

**Timing**	Cross-sectional
**Setting and intended use of the prediction model**	Paediatric MRI service at single US academic hospital, intended replacement of manual segmentation for routine GFR estimation in paediatric MR urography.

Step 2 – Classify the type of prediction model assessment

**Classify the assessment based on its aim**			
Type of prediction study	***Explanation***	***PROBAST+AI boxes to complete***	***Tick as appropriate***
**Development only**	**Prediction model development only, i.e., without evaluation of its performance.**	**Model development**	
**Evaluation only**	**External validation of one or more existing models in new data**	**Model evaluation**	
**Combination**	**Prediction model development combined in the same study (publication) with the evaluation of its apparent performance, internal validation performance, or external validation performance.**	**Model development and evaluation**	**X**

Step 3 - Assess quality and applicability or risk of bias and applicability

**Publication reference**	Yoruk U, Hargreaves BA, Vasanawala SS. Automatic renal segmentation for MR urogra-phy using 3D-GrabCut and random forests. Magn Reson Med. 2018;79(3):1696–707.
**Models of interest**	RF classifier
**Outcome of interest**	Single-kidney GFR and per-voxel tissue labelling.

Model development

**DOMAIN 1: Participants and data sources**	
**A. Quality**	
26 consecutive paediatric clinical DCE-MR urography subjects, retrospectively identified, three acquisition protocols, anatomical anomalies present, 11 selected for training, remaining 15 for testing.	
	**Y/ PY/ PN/ N/ NI**
**1.1 Were appropriate data sources used?**	PY
**1.2 Was an appropriate study design used?**	PY

**1.3 Did the in- and exclusions of study participants result in a representative dataset?**	PN
**Concern regarding quality of selection of participants and data sources**	**QUALITYCONCERN:** *low/**high**/unclear*
Rationale of quality rating: Single-centre, small subject sample, heterogeneous acquisition protocols and anatomy, voxel-level n is large but voxels not independent.	
**B. Applicability**	
Single US academic centre, 26 paediatric subjects retrospectively identified, three acquisition-protocol groups	
**Concern that the (data of the) included participants do not match the review question or the assessor's intended use of the prediction model**	**APPLICABILITY CONCERN:** *low/**high**/unclear*
Rationale of applicability rating: Paediatric DCE-MRI on a single GE 3T scanner, low generalizability	

**DOMAIN 2: Predictors**	
**A. Quality**	
Per-voxel features: signal intensity at 0, 30, 60, 90, 120, 150 s post-contrast, plus depth within the kidney mask	
	**Y/ PY/ PN/ N/ NI**
**2.1 Were predictors defined and assessed in a similar way for all participants?**	Y
**2.2 Was any pre-processing of predictors similar for all participants?**	Y
**2.3 Were predictor assessments made without knowledge of outcome data?**	Y
**2.4 Were the predictors included in the model available at the time the model was intended to be used?**	Y
**Concern regarding quality of selection of participants and data sources**	**QUALITYCONCERN:** *__low__/high/unclear*
Predictor pipeline fully standardised, blinded to outcome	
**B. Applicability**	
**Concern that the definition, pre-processing, assessment, or timing of assessment of the predictors in the model do not match the review question or the assessor's intended use**	**APPLICABILITY CONCERN:** *low/**high**/unclear*
Rationale of applicability rating: Predictors are DCE-MRI-specific.	

**DOMAIN 3: Outcome**	
**A. Quality**	
Reference 'outcome' for voxel labelling = manually drawn cortex / medulla / collecting-system labels by a trained technologist, reference 'outcome' for GFR = GFR computed from a two-compartment pharmacokinetic model fed with the manual whole-kidney ROI signal-time curve, F1 score used for segmentation agreement, Spearman ρ between automatic and manual GFR, no temporal mismatch	
	**Y/ PY/ PN/ N/ NI**

**3.1 Were outcomes defined and assessed appropriately?**	PY
**3.2 Were outcomes defined and assessed in a similar way for all participants?**	Y
**3.3 Were outcome assessments made without use or knowledge of predictor data?**	PY
**3.4 Was the time interval between predictor assessment and outcome assessment appropriate?**	Y
**Concern regarding quality of the outcome or its determination**	**QUALITYCONCERN:** ***low**/high/unclear*
Manual ROI is the standard practical reference, but is operator-dependent surrogate, not a true clinical gold standard.	
**B. Applicability**	
Outcome = GFR from compartmental modelling, not a measured GFR.	
**Concern that the outcome, its definition, assessment, or timing of assessment do not match the review question or the assessor's intended use**	**APPLICABILITY CONCERN:** *low/**high**/unclear*
Outcome is a different construct, compared to manual segmentation which is operator dependent.	

**DOMAIN 4: Analysis**	
**A. Quality**	
Numbers: 11 training subjects, 15 test subjects, 7 voxel features.	
5-fold CV, comparison with SVM-rbf and AdaBoost on a small balanced 10 000-voxel sample (50/50 train/test split).	
Per-voxel: F1, kidney / cortex F1, Spearman ρ between automatic and manual GFR, mean absolute % GFR error, reporting of two complete algorithm failures	
Missing data: voxel-level data, no missingness.	
	**Y/ PY/ PN/ N/ NI**
**4.1 Was there evidence that the sample size was reasonable?**	PN
**4.2 Were continuous and categorical predictors handled appropriately?**	PY
**4.3 Were participants with missing or censored data handled appropriately in the analysis?**	NA
**4.4 If methods to address class imbalance were used, was the model or the model predictions recalibrated?***	PY
**4.5 Were methods used to address potential model overfitting?**	PY
**Concern regarding quality of the analysis**	**QUALITYCONCERN:** *low/**high**/unclear*
Subject-level whitening, 5-fold CV, multi-classifier comparison, explicit failure-case reporting, small subject sample, limits generalisability	

Model evaluation

Domain 1,2 and 3 same analysis as model development

DOMAIN 4: Analysis			
**Risk of bias**			
15 test subjects, 915 000 test voxels.			
Per-voxel: F1, kidney / cortex F1, Spearman ρ between automatic and manual GFR, mean absolute % GFR error, reporting of two complete algorithm failures			
Describe any participants who were excluded from the analysis: Exclusion same as development			
Describe missing data on predictors and outcomes as well as methods used for handling these missing data: Not applicable at the voxel level.			
	**Y/ PY/ PN/ N/ NI/ NA**		
**4.1 Was model evaluation based on only apparent performance avoided?**	Y		
	**A**	**I**	**E**
**4.2 Was there evidence that the sample size was reasonable?**	PN	PN	NA
**4.3 Were participants with missing or censored data handled appropriately in the analysis?**	NA	NA	NA
**4.4 If methods to address class imbalance were used, was the evaluation done in a dataset without imbalance correction?***	PN	PN	NA
**4.5 If data splitting was done to create training and test datasets, was there evidence that data leakage was avoided?***	**NA**	PY	**NA**
**4.6 If resampling methods were used to evaluate model performance, were all model development steps replicated in the resampling process?***	**NA**	PY	**NA**
**4.7 Was the predictive performance of the model evaluated appropriately, e.g., calibration, discrimination, and net benefit?**	PN	PN	NA
**Risk of bias introduced by the analysis**	**RISK OF BIAS:** *low/**high**/unclear*		
Hold-out test on 15 unseen subjects, voxel-level F1 + GFR error reported, transparent failure-case reporting, open-source code, sample too small to provide meaningful confidence intervals on GFR error or to demonstrate robustness.			

Step 4 - Assess the overall concerns regarding quality, risk of bias and applicability of the prediction model

OVERALL JUDGEMENT OF PREDICTION MODEL	
**Overall judgement of quality (development)**	**QUALITY CONCERN:** *low/**high**/unclear*
High concern in D1 and D4	
**Overall judgement of risk of bias (evaluation)**	**RISK OF BIAS:** *low/**high**/unclear*
Hold-out test on 15 subjects, no external validation.	
**Overall judgement of applicability (development)**	**APPLICABILITY CONCERN:** *low/**high**/unclear*

Paediatric DCE-MRI for GFR estimation	
**Overall judgement of applicability (evaluation)**	**APPLICABILITY CONCERN:** *low/**high**/unclear*
Same as development	

Mo et al. (40)

Step 1 - PICOTS

**Population**	Adults with hypertension and diabetes recruited at a Chinese hospital for renal MRI.
**Index model(s)**	SVM classifier on SURF + Bag-of-Words texture features from coronal T2WI, on four ROI strategies: All-K (all coronal slices), LC-K (largest coronal slice), TLCO-K (12 concentric layers of largest slice), PIZZA-K (6 wedges of largest slice).
**Comparator model(s)**	Comparison four segmentation strategies
**Outcome(s)**	Three-class CKD-EPI eGFR category: normal vs mild–moderate vs severe impairment.
**Timing**	Cross-sectional
**Setting and intended use of the prediction model**	Tertiary nephrology centre, China, intended non-invasive functional triage for diabetic + hypertensive CKD patients.

Step 2 – Classify the type of prediction model assessment

**Classify the assessment based on its aim**			
Type of prediction study	***Explanation***	***PROBAST+AI boxes to complete***	***Tick as appropriate***
**Development only**	**Prediction model development only, i.e., without evaluation of its performance.**	**Model development**	
**Evaluation only**	**External validation of one or more existing models in new data**	**Model evaluation**	
**Combination**	**Prediction model development combined in the same study (publication) with the evaluation of its apparent performance, internal validation performance, or external validation performance.**	**Model development and evaluation**	**X**

Step 3 - Assess quality and applicability or risk of bias and applicability

**Publication reference**	Mo XK, Chen WB, Chen SM, Chen ZZ, Guo YS, Chen YL, et al. MRI texture-based machine learning models for the evaluation of renal function on different segmentations: a proof-of-concept study. Insights into Imaging. 2023;14(1).
**Models of interest**	SVM with SURF + BoW texture features across four segmentation strategies (All-K / LC-K / TLCO-K / PIZZA-K).
**Outcome of interest**	Three-class eGFR-based category.

Model development

**DOMAIN 1: Participants and data sources**	
**A. Quality**	
Hypertensive and diabetic patients undergoing renal MRI at one Chinese tertiary hospital between October 2013 and November 2019, 174 analysed, 7/3 training/ testing, exclusion criteria: severe T2WI artefacts, polycystic kidney disease, acute kidney injury, medications affecting creatinine, poorly-controlled hypertension.	
	**Y/ PY/ PN/ N/ NI**
**1.1 Were appropriate data sources used?**	PY
**1.2 Was an appropriate study design used?**	PY
**1.3 Did the in- and exclusions of study participants result in a representative dataset?**	PN
**Concern regarding quality of selection of participants and data sources**	**QUALITYCONCERN:** *low/**high**/unclear*
Rationale of quality rating: Single-centre Chinese cohort restricted to hypertensive + diabetic patients a small severe-impairment subgroup.	
**B. Applicability**	
Single Chinese tertiary centre ,174 hypertensive + diabetic patients prospectively recruited October 2013 – November 2019 for renal MRI.	
**Concern that the (data of the) included participants do not match the review question or the assessor's intended use of the prediction model**	**APPLICABILITY CONCERN:** *low/**high**/unclear*
Rationale of applicability rating: Restriction to diabetic + hypertensive CKD, excludes glomerular and other CKD causes, single Chinese centre, single 3T GE scanner.	

**DOMAIN 2: Predictors**	
**A. Quality**	
Coronal T2WI on 3T GE Discovery MR750, kidneys manually segmented on coronal T2WI in ITK-SNAP by 5-y radiologist (reader 1), 30 randomly chosen cases re-segmented by 8-y radiologist (reader 2), disagreements resolved by consensus with an abdominal-radiology expert, four ROI strategies (All-K / LC-K / TLCO-K / PIZZA-K), Bag-of-Words clustering to harmonise feature length.	
	**Y/ PY/ PN/ N/ NI**
**2.1 Were predictors defined and assessed in a similar way for all participants?**	Y
**2.2 Was any pre-processing of predictors similar for all participants?**	Y
**2.3 Were predictor assessments made without knowledge of outcome data?**	Y

**2.4 Were the predictors included in the model available at the time the model was intended to be used?**	Y
**Concern regarding quality of selection of participants and data sources**	**QUALITYCONCERN:** ***low***/*high/unclear*
Predictor pipeline is well standardized, intensity normalisation, BoW harmonisation, ROI drawing remains source of variability	
**B. Applicability**	
**Concern that the definition, pre-processing, assessment, or timing of assessment of the predictors in the model do not match the review question or the assessor's intended use**	**APPLICABILITY CONCERN:** ***low***/*high/unclear*
Rationale of applicability rating: Coronal T2WI is a routine clinical sequence, the SURF + BoW + SVM pipeline is general and could be deployed in any radiology workflow with comparable scanners	

**DOMAIN 3: Outcome**	
**A. Quality**	
Outcome = three-category CKD-EPI eGFR (normal / mild–moderate / severe), computed from serum creatinine averaged from one week pre- and one week post-MRI, outcome independent of texture features, eGFR-based	
	**Y/ PY/ PN/ N/ NI**
**3.1 Were outcomes defined and assessed appropriately?**	PY
**3.2 Were outcomes defined and assessed in a similar way for all participants?**	Y
**3.3 Were outcome assessments made without use or knowledge of predictor data?**	Y
**3.4 Was the time interval between predictor assessment and outcome assessment appropriate?**	Y
**Concern regarding quality of the outcome or its determination**	**QUALITYCONCERN:** ***low***/*high/unclear*
eGFR is reference standard, outcome independent of features	
**B. Applicability**	
Outcome = GFR from compartmental modelling, not a measured GFR.	
**Concern that the outcome, its definition, assessment, or timing of assessment do not match the review question or the assessor's intended use**	**APPLICABILITY CONCERN:** ***low***/*high/unclear*
Outcome is a different construct than features, easily measured, widely-used	

**DOMAIN 4: Analysis**
**A. Quality**
Numbers: n = 174 (training 123, testing 51), SVM for three classes.
DeLong test for AUC comparisons between segmentation strategies, AUROC, sensitivity, specificity, accuracy reported with CIs in three binary tasks .

14 patients excluded for motion / cysts
Missing data: no imputation

	Y/ PY/ PN/ N/ NI
**4.1 Was there evidence that the sample size was reasonable?**	PN
**4.2 Were continuous and categorical predictors handled appropriately?**	PY
**4.3 Were participants with missing or censored data handled appropriately in the analysis?**	NI
**4.4 If methods to address class imbalance were used, was the model or the model predictions recalibrated?***	PY
**4.5 Were methods used to address potential model overfitting?**	PY
**Concern regarding quality of the analysis**	**QUALITYCONCERN:** *low/**high**/unclear*
Dataset is too small to stably train high-dimensional texture models, no cross-validation, no calibration, no external validation.	

Model evaluation

Domain 1,2 and 3 same analysis as model development

**DOMAIN 4: Analysis**
**Risk of bias**
51 validation patients across three eGFR classes.
DeLong test for AUC comparisons between segmentation strategies, AUROC, sensitivity, specificity, accuracy reported with CIs in three binary tasks.
Describe any participants who were excluded from the analysis: Exclusion same as development
Describe missing data on predictors and outcomes as well as methods used for handling these missing data: Same as development

	Y/ PY/ PN/ N/ NI/ NA		
**4.1 Was model evaluation based on only apparent performance avoided?**	Y		
	**A**	**I**	**E**
**4.2 Was there evidence that the sample size was reasonable?**	PN	PN	NA
**4.3 Were participants with missing or censored data handled appropriately in the analysis?**	NI	NI	NA
**4.4 If methods to address class imbalance were used, was the evaluation done in a dataset without imbalance correction?***	NA	NA	NA
**4.5 If data splitting was done to create training and test datasets, was there evidence that data leakage was avoided?***	**NA**	PY	**NA**
**4.6 If resampling methods were used to evaluate model performance, were all model development steps replicated in the resampling process?***	**NA**	NA	**NA**
**4.7 Was the predictive performance of the model evaluated appropriately, e.g., calibration, discrimination, and net benefit?**	PN	PN	NA

Risk of bias introduced by the analysis	RISK OF BIAS: *low/**high**/unclear*
Random 7/3 split with only 51 validation patients limits stability, no cross-validation, no calibration, no external validation.	

Step 4 - Assess the overall concerns regarding quality, risk of bias and applicability of the prediction model

OVERALL JUDGEMENT OF PREDICTION MODEL	
**Overall judgement of quality (development)**	**QUALITY CONCERN:** *low/**high**/unclear*
High concern in D1 and D4, small single-centre cohort, no calibration, no external validation.	
**Overall judgement of risk of bias (evaluation)**	**RISK OF BIAS:** *low/**high**/unclear*
Single 7/3 split, no cross-validation, no external validation.	
**Overall judgement of applicability (development)**	**APPLICABILITY CONCERN:** *low/**high**/unclear*
Single Chinese centre, restricted to hypertensive + diabetic CKD	
**Overall judgement of applicability (evaluation)**	**APPLICABILITY CONCERN:** *low/**high**/unclear*
Same as development	

Bin Islam et al. (41)

Step 1 - PICOTS

**Population**	Public Zenodo dataset (60 individuals: 30 healthy, 30 CKD) + small test set data with subjects scanned 5x each
**Index model(s)**	Resnet18, Densenet201, VGG19, MobileNetv2, Inceptionv3, AlexNet, EfficientNet_b1, tf_mobilenetv3_large_075, xception71. Best: EfficientNet_b1. Segmentation, proposed Resnet18-Self-ONN-UNet++
**Comparator model(s)**	Resnet18-UNet++, MobileNetv2-UNet++, Densenet121-UNet++, Inceptionv4-UNet++; existing literature methods.
**Outcome(s)**	Binary classification healthy vs CKD; kidney-region segmentation evaluated by IoU, Dice, accuracy, precision, sensitivity, specificity, FNR, FPR.
**Timing**	Cross-sectional

**Setting and intended use of the prediction model**	Research, envisaged as aid for early kidney-disease screening and treatment planning by MRI image analysis.

Step 2 – Classify the type of prediction model assessment

**Classify the assessment based on its aim**			
Type of prediction study	***Explanation***	***PROBAST+AI boxes to complete***	***Tick as appropriate***
**Development only**	**Prediction model development only, i.e., without evaluation of its performance.**	**Model development**	
**Evaluation only**	**External validation of one or more existing models in new data**	**Model evaluation**	
**Combination**	**Prediction model development combined in the same study (publication) with the evaluation of its apparent performance, internal validation performance, or external validation performance.**	**Model development and evaluation**	**X**

Step 3 - Assess quality and applicability or risk of bias and applicability

**Publication reference**	Bin Islam MS, Sumon MSI, Sarmun R, Bhuiyan EH, Chowdhury MEH. Classification and segmentation of kidney MRI images for chronic kidney disease detection. Computers & Electrical Engineering. 2024;119.
**Models of interest**	EfficientNet_b1
**Outcome of interest**	Healthy vs CKD classification and kidney segmentation on T2-weighted MRI.

Model development

**DOMAIN 1: Participants and data sources**	
**A. Quality**	
60 individuals (30 healthy / 30 CKD) Image-level 5-fold CV and 70:10:20 split (909:130:260 per fold).	
	**Y/ PY/ PN/ N/ NI**
**1.1 Were appropriate data sources used?**	PY
**1.2 Was an appropriate study design used?**	PY
**1.3 Did the in- and exclusions of study participants result in a representative**	PN

**dataset?**	
**Concern regarding quality of selection of participants and data sources**	**QUALITYCONCERN:** *low/**high**/unclear*
Rationale of quality rating: Only 60 patients with a >30-year mean age gap between healthy and CKD groups, binary classifier can exploit age-correlated MRI features rather than CKD-specific signal. No external cohort.	
**B. Applicability**	
Public-dataset convenience sample with a marked age imbalance, scanned on a single 3T machine intended use is broad early-CKD MRI screening.	
**Concern that the (data of the) included participants do not match the review question or the assessor's intended use of the prediction model**	**APPLICABILITY CONCERN:** *low/**high**/unclear*
Rationale of applicability rating: Small, age-confounded, single-scanner cohort; MRI is not standard for CKD diagnosis in most clinical settings, further limiting applicability of the model to its stated intended use.	

**DOMAIN 2: Predictors**	
**A. Quality**	
Predictors are T2-HASTE coronal MRI slices converted to PNG, aggressive augmentation inflates 1,299 images to 8,402 for classification and 12,000 for segmentation.	
	**Y/ PY/ PN/ N/ NI**
**2.1 Were predictors defined and assessed in a similar way for all participants?**	Y
**2.2 Was any pre-processing of predictors similar for all participants?**	Y
**2.3 Were predictor assessments made without knowledge of outcome data?**	Y
**2.4 Were the predictors included in the model available at the time the model was intended to be used?**	Y
**Concern regarding quality of selection of participants and data sources**	**QUALITYCONCERN:** ***low**/high/unclear*
Uniform MRI acquisition and processing pipeline; pre-processing applied identically to healthy and CKD groups	
**B. Applicability**	
**Concern that the definition, pre-processing, assessment, or timing of assessment of the predictors in the model do not match the review question or the assessor's intended use**	**APPLICABILITY CONCERN:** *low/**high**/unclear*
Rationale of applicability rating: MRI is not routine CKD imaging modality and model is tied to single scanner and protocol, transportability to other devices is unverified.	

**DOMAIN 3: Outcome**	
**A. Quality**	
Outcome labels pre-defined with the Zenodo dataset, segmentation ground truth is a binary mask provided with the data.	
	**Y/ PY/ PN/ N/ NI**
**3.1 Were outcomes defined and assessed appropriately?**	PY

**3.2 Were outcomes defined and assessed in a similar way for all participants?**	PY
**3.3 Were outcome assessments made without use or knowledge of predictor data?**	NI
**3.4 Was the time interval between predictor assessment and outcome assessment appropriate?**	PY
**Concern regarding quality of the outcome or its determination**	**QUALITYCONCERN:** *low/high/**unclear***
CKD definition and procedure for generating binary segmentation masks are not described in detail, CKD stages 1–5 are pooled into one positive class.	
**B. Applicability**	
Outcome is single pooled CKD class without staging.	
**Concern that the outcome, its definition, assessment, or timing of assessment do not match the review question or the assessor's intended use**	**APPLICABILITY CONCERN:** *low/**high**/unclear*
Pooling CKD stages 1–5 means the model cannot inform any clinically meaningful decision about disease severity or prognosis.	

**DOMAIN 4: Analysis**	
**A. Quality**	
60 patients, 1,299 native slices augmented to 8,402 (classification) / 12,000 (segmentation). Train/val/test split is at the IMAGE level (909/130/260 per fold), not patient level, same patient's slices may appear in train and test, creating substantial leakage risk.	
BCE/Dice loss, 5-fold CV	
Accuracy, precision, F1, sensitivity, specificity, IoU, Dice, no CIs, no calibration, no patient-level analysis.	
Missing data: no imputation	
	**Y/ PY/ PN/ N/ NI**
**4.1 Was there evidence that the sample size was reasonable?**	PN
**4.2 Were continuous and categorical predictors handled appropriately?**	PY
**4.3 Were participants with missing or censored data handled appropriately in the analysis?**	NA
**4.4 If methods to address class imbalance were used, was the model or the model predictions recalibrated?***	NA
**4.5 Were methods used to address potential model overfitting?**	PN
**Concern regarding quality of the analysis**	**QUALITYCONCERN:** *low/**high**/unclear*
Only 60 patients with image-level splitting that almost certainly mixes same patient's slices across train, validation and test sets, reported accuracies very likely inflated by leakage and by age confounding, no patient-level metrics or confidence intervals.	

Model evaluation

Domain 1,2 and 3 same analysis as model development

DOMAIN 4: Analysis			
**Risk of bias**			
260 test images per fold from the same 60-patient pool.			
Accuracy, precision, F1, sensitivity, specificity, IoU, Dice. No CIs, no calibration, no patient-level analysis.			
Describe any participants who were excluded from the analysis:<br>No information about exclusion			
Describe missing data on predictors and outcomes as well as methods used for handling these missing data:<br>No information about handling missing data			
	**Y/ PY/ PN/ N/ NI/ NA**		
**4.1 Was model evaluation based on only apparent performance avoided?**	Y		
	**A**	**I**	**E**
**4.2 Was there evidence that the sample size was reasonable?**	PN	PN	NA
**4.3 Were participants with missing or censored data handled appropriately in the analysis?**	NA	NA	NA
**4.4 If methods to address class imbalance were used, was the evaluation done in a dataset without imbalance correction?***	NA	NA	NA
**4.5 If data splitting was done to create training and test datasets, was there evidence that data leakage was avoided?***	**NA**	PN	**NA**
**4.6 If resampling methods were used to evaluate model performance, were all model development steps replicated in the resampling process?***	**NA**	NY	**NA**
**4.7 Was the predictive performance of the model evaluated appropriately, e.g., calibration, discrimination, and net benefit?**	PN	PN	NA
**Risk of bias introduced by the analysis**	**RISK OF BIAS:**<br>*low/**high**/unclear*		
Image-level splitting from a 60-patient pool ,patient-level data leakage is almost certain and inflates all metrics, no calibration or AUC-ROC reported for classification, no external validation.			

Step 4 - Assess the overall concerns regarding quality, risk of bias and applicability of the prediction model

OVERALL JUDGEMENT OF PREDICTION MODEL	
**Overall judgement of quality (development)**	**QUALITY CONCERN:**<br>*low/**high**/unclear*
High concern in D1 and D4, D3 unclear, tiny age-confounded sample with image-level splits.	
**Overall judgement of risk of bias (evaluation)**	**RISK OF BIAS:**<br>*low/**high**/unclear*
Internal-only evaluation with image-level splits and likely leakage, no calibration, no patient-level metrics.	
**Overall judgement of applicability (development)**	**APPLICABILITY CONCERN:**<br>*low/**high**/unclear*

Public single-scanner dataset, MRI not a routine CKD screening modality, binary label pools all CKD stages.

**Overall judgement of applicability (evaluation)**	**APPLICABILITY CONCERN:** *low/**high**/unclear*
Same single-scanner, age-confounded limitations apply.	

Fu et al. (42)

Step 1 - PICOTS

**Population**	79 patients with renal cysts at a single hospital, China, 52 training, 27 testing, 6000 CT slice images. No external cohort.
**Index model(s)**	RDA-UNET: U-Net backbone augmented with a residual dual-attention
**Comparator model(s)**	FCN-8S, FCN-16S, U-Net, U-Net (+BN), UNet++ and FCN-VGG10
**Outcome(s)**	Pixel-wise cyst/kidney segmentation evaluated by Dice similarity coefficient, precision and recall for left and right kidney.
**Timing**	Cross-sectional
**Setting and intended use of the prediction model**	Hospital CT clinic in China, intended as quantitative CT segmentation aid for assessing renal cysts in CKD and supporting 3D kidney-volume reconstruction.

Step 2 – Classify the type of prediction model assessment

**Classify the assessment based on its aim**			
Type of prediction study	***Explanation***	***PROBAST+AI boxes to complete***	***Tick as appropriate***
**Development only**	**Prediction model development only, i.e., without evaluation of its performance.**	**Model development**	
**Evaluation only**	**External validation of one or more existing models in new data**	**Model evaluation**	
**Combination**	**Prediction model development combined in the same study (publication) with the evaluation of its apparent performance, internal validation performance, or external validation performance.**	**Model development and evaluation**	**X**

Step 3 - Assess quality and applicability or risk of bias and applicability

**Publication reference**	Fu X, Liu H, Bi X, Gong X. Deep-Learning-Based CT Imaging in the Quantitative Evalua-tion of Chronic Kidney Diseases. J Healthc Eng. 2021;2021:3774423.
**Models of interest**	RDA-UNET
**Outcome of interest**	Voxel-level renal-cyst/kidney segmentation as a substrate for CKD quantification.

Model development

**DOMAIN 1: Participants and data sources**	
**A. Quality**	
Single-centre cohort, 79 patients selected, 27 patients in testing group, 6,000 CT slices in total, no demographic table or co-morbidity profile reported, no external dataset.	
	**Y/ PY/ PN/ N/ NI**
**1.1 Were appropriate data sources used?**	PY
**1.2 Was an appropriate study design used?**	PY
**1.3 Did the in- and exclusions of study participants result in a representative dataset?**	PN
**Concern regarding quality of selection of participants and data sources**	**QUALITYCONCERN:** *low/**high**/unclear*
Rationale of quality rating: Very small (n=79) single-centre sample selected on the presence of cysts, with no demographic or clinical description and no external cohort, case selection is not representative of a general CKD population.	
**B. Applicability**	
Population is patients with renal cysts on CT from one Chinese hospital, with no validation in CKD patients without cysts or in other populations.	
**Concern that the (data of the) included participants do not match the review question or the assessor's intended use of the prediction model**	**APPLICABILITY CONCERN:** *low/**high**/unclear*
Rationale of applicability rating: Renal cysts one of many structural CKD features, selecting patients on the presence of cysts and limiting to single hospital substantially narrows the population.	

**DOMAIN 2: Predictors**	
**A. Quality**	
Raw CT voxels processed through the RDA-UNET pipeline: window 420/60 HU, resized to 256×256,.	
	**Y/ PY/ PN/ N/ NI**
**2.1 Were predictors defined and assessed in a similar way for all participants?**	Y
**2.2 Was any pre-processing of predictors similar for all participants?**	Y
**2.3 Were predictor assessments made without knowledge of outcome data?**	Y

**2.4 Were the predictors included in the model available at the time the model was intended to be used?**	Y
**Concern regarding quality of selection of participants and data sources**	**QUALITYCONCERN:** ***low****/high/unclear*
CT images acquired and pre-processed uniformly, feature extraction is automated and independent of the segmentation label.	
**B. Applicability**	
**Concern that the definition, pre-processing, assessment, or timing of assessment of the predictors in the model do not match the review question or the assessor's intended use**	**APPLICABILITY CONCERN:** *low/****high****/unclear*
Rationale of applicability rating: Single-vendor, single-protocol CT acquisition limits transportability to scanners with different reconstruction kernels or slice thicknesses.	

**DOMAIN 3: Outcome**	
**A. Quality**	
Outcome is the cyst contour drawn by experienced experts. Dice score is computed against this manual reference on the test set.	
	**Y/ PY/ PN/ N/ NI**
**3.1 Were outcomes defined and assessed appropriately?**	PY
**3.2 Were outcomes defined and assessed in a similar way for all participants?**	Y
**3.3 Were outcome assessments made without use or knowledge of predictor data?**	PN
**3.4 Was the time interval between predictor assessment and outcome assessment appropriate?**	NA
**Concern regarding quality of the outcome or its determination**	**QUALITYCONCERN:** *low/high/****unclear***
Ground truth itself derived from the CT images and is tnot blinded to predictor, number of annotators and training not reported.	
**B. Applicability**	
Outcome is cyst/kidney contour, not a clinical CKD endpoint	
**Concern that the outcome, its definition, assessment, or timing of assessment do not match the review question or the assessor's intended use**	**APPLICABILITY CONCERN:** *low/****high****/unclear*
Pixel-level segmentation accuracy is only a surrogate for clinical intended use, no link to eGFR, kidney volume change or patient-level outcomes is established.	

**DOMAIN 4: Analysis**
**A. Quality**
6,000 slices from 79 patients; 52/27 patient split (no explicit patient-level guarantee for slices).
BCE/Dice loss

DSC, precision, recall, no confidence intervals, no calibration, no decision-curve analysis, no cross-validation.
Missing data not described, class imbalance not addressed.

	Y/ PY/ PN/ N/ NI
**4.1 Was there evidence that the sample size was reasonable?**	PN
**4.2 Were continuous and categorical predictors handled appropriately?**	PY
**4.3 Were participants with missing or censored data handled appropriately in the analysis?**	NI
**4.4 If methods to address class imbalance were used, was the model or the model predictions recalibrated?***	NA
**4.5 Were methods used to address potential model overfitting?**	PY
**Concern regarding quality of the analysis**	**QUALITYCONCERN:** *low/**high**/unclear*
Only 79 patients with no cross-validation or confidence intervals, single hold-out split, no calibration or decision-curve analysis, patient-level integrity of the slice split is not explicitly confirmed.	

Model evaluation

Domain 1,2 and 3 same analysis as model development

**DOMAIN 4: Analysis**
**Risk of bias**
27 test patients, segmentation reported per kidney (left, right).
DSC, precision, recall, no confidence intervals, no calibration, no decision-curve analysis, no cross-validation.
Describe any participants who were excluded from the analysis: Patients without obvious cysts or with poor image quality.
Describe missing data on predictors and outcomes as well as methods used for handling these missing data: Missing data: not described

	Y/ PY/ PN/ N/ NI/ NA		
**4.1 Was model evaluation based on only apparent performance avoided?**	Y		
	**A**	**I**	**E**
**4.2 Was there evidence that the sample size was reasonable?**	PN	PN	NA
**4.3 Were participants with missing or censored data handled appropriately in the analysis?**	NA	NI	NA
**4.4 If methods to address class imbalance were used, was the evaluation done in a dataset without imbalance correction?***	NA	NA	NA
**4.5 If data splitting was done to create training and test datasets, was there evidence that data leakage was avoided?***	**NA**	NI	**NA**
**4.6 If resampling methods were used to evaluate model performance, were all model development steps replicated in the resampling process?***	**NA**	NA	**NA**
**4.7 Was the predictive performance of the model evaluated appropriately, e.g., calibration, discrimination, and net benefit?**	PN	PN	NA

Risk of bias introduced by the analysis	RISK OF BIAS:<br>*low/**high**/unclear*
Tiny internal-only test (27 patients), no confidence intervals, no calibration or decision-curve analysis, ambiguous slice-vs-patient split (potential leakage) and no external validation.	

Step 4 - Assess the overall concerns regarding quality, risk of bias and applicability of the prediction model

OVERALL JUDGEMENT OF PREDICTION MODEL	
**Overall judgement of quality (development)**	**QUALITY CONCERN:**<br>*low/**high**/unclear*
High concern in D1 and D4, D3 unclear, very small selected sample and weak analytic methodology.	
**Overall judgement of risk of bias (evaluation)**	**RISK OF BIAS:**<br>*low/**high**/unclear*
Internal hold-out only with 27 patients, no calibration, no external validation, possible slice-level leakage.	
**Overall judgement of applicability (development)**	**APPLICABILITY CONCERN:**<br>*low/**high**/unclear*
Single-centre, single-scanner Chinese cohort of patients selected on cyst presence.	
**Overall judgement of applicability (evaluation)**	**APPLICABILITY CONCERN:**<br>*low/**high**/unclear*
Same single-centre, cyst-selected limitations apply.	

Lee et al. (43)

Step 1 - PICOTS

**Population**	30 individuals from US hospital, independent validation: 42 additional individuals (18 non-CKD, 24 CKD stage 5D). HRpQCT scans of distal and diaphyseal radius and tibia.
**Index model(s)**	Four site-specific models, U-Net segmentation, GLCM texture features, XGBoost classifier
**Comparator model(s)**	Manufacturer-supplied segmentation and standard HRpQCT outcomes, DXA
**Outcome(s)**	Image-level binary classification CKD vs non-CKD, per-image segmentation metrics
**Timing**	Cross-sectional
**Setting and intended use of the prediction model**	Research, improve sensitivity of HRpQCT-based detection of CKD-related cortical bone abnormalities, complementing DXA and conventional CT outcomes.

Step 2 – Classify the type of prediction model assessment

**Classify the assessment based on its aim**			
Type of prediction study	***Explanation***	***PROBAST+AI boxes to complete***	***Tick as appropriate***
**Development only**	**Prediction model development only, i.e., without evaluation of its performance.**	**Model development**	
**Evaluation only**	**External validation of one or more existing models in new data**	**Model evaluation**	
**Combination**	**Prediction model development combined in the same study (publication) with the evaluation of its apparent performance, internal validation performance, or external validation performance.**	**Model development and evaluation**	**X**

Step 3 - Assess quality and applicability or risk of bias and applicability

**Publication reference**	Lee Y, Bandara WR, Park S, Lee M, Seo C, Yang S, et al. Integrating deep learning and ma-chine learning for improved CKD-related cortical bone assessment in HRpQCT images: A pilot study. Bone Rep. 2025;24:101821.
**Models of interest**	Site-specific U-Net (segmentation) + XGBoost (classification)
**Outcome of interest**	CKD vs non-CKD image-level classification and cortical-bone segmentation in HRpQCT.

Model development

**DOMAIN 1: Participants and data sources**	
**A. Quality**	
Single-centre cohort, 30 participants split 70/10/20 by patient, CKD group skewed to advanced disease (8/10 stage 5D), independent validation comprises more advanced CKD subset, conventional DXA/HRpQCT outcomes did not statistically differ between CKD and non-CKD apart from female whole-body Z-score.	
	**Y/ PY/ PN/ N/ NI**
**1.1 Were appropriate data sources used?**	Y
**1.2 Was an appropriate study design used?**	PY
**1.3 Did the in- and exclusions of study participants result in a representative dataset?**	PN
**Concern regarding quality of selection of participants and data sources**	**QUALITYCONCERN:** *low/**high**/unclear*
Rationale of quality rating: Very small patient counts from a single institution, CKD group is heavily weighted to dialysis-dependent stage 5D, limiting representativeness across the CKD spectrum.	

B. Applicability	
Single US-institution pilot cohort weighted to advanced CKD (mostly stage 5D dialysis patients); intended use is broad CKD-related cortical-bone assessment but population effectively covers severe CKD only.	
**Concern that the (data of the) included participants do not match the review question or the assessor's intended use of the prediction model**	**APPLICABILITY CONCERN:** *low/**high**/unclear*
Rationale of applicability rating: Pilot single-institution cohort skewed to dialysis-dependent CKD, HRpQCT itself is not widely available.	

DOMAIN 2: Predictors	
**A. Quality**	
Predictors are GLCM textural features computed from U-Net-segmented cortical bone on HRpQCT images, all scans on a single XtremeCT II scanner with standardised acquisition.	
	**Y/ PY/ PN/ N/ NI**
**2.1 Were predictors defined and assessed in a similar way for all participants?**	Y
**2.2 Was any pre-processing of predictors similar for all participants?**	Y
**2.3 Were predictor assessments made without knowledge of outcome data?**	Y
**2.4 Were the predictors included in the model available at the time the model was intended to be used?**	Y
**Concern regarding quality of selection of participants and data sources**	**QUALITYCONCERN:** ***low**/high/unclear*
Single scanner type with proprietary protocol, HRpQCT availability is limited globally.	
**B. Applicability**	
**Concern that the definition, pre-processing, assessment, or timing of assessment of the predictors in the model do not match the review question or the assessor's intended use**	**APPLICABILITY CONCERN:** *low/**high**/unclear*
Rationale of applicability rating: HRpQCT is a research-grade modality with limited clinical availability and no multi-vendor validation.	

DOMAIN 3: Outcome	
**A. Quality**	
Outcome is clinical CKD stage 3–5D vs age/sex-matched non-CKD controls, defined independently of the imaging features, independent validation restricted to CKD stage 5D.	
	**Y/ PY/ PN/ N/ NI**
**3.1 Were outcomes defined and assessed appropriately?**	Y
**3.2 Were outcomes defined and assessed in a similar way for all participants?**	Y
**3.3 Were outcome assessments made without use or knowledge of predictor data?**	Y
**3.4 Was the time interval between predictor assessment and outcome assessment appropriate?**	Y

**Concern regarding quality of the outcome or its determination**	**QUALITYCONCERN:** ***low***/*high/unclear*
CKD status clinically defined and independent of the HRpQCT-derived predictors.	
**B. Applicability**	
Binary CKD vs non-CKD, no severity gradation despite mixed CKD stages in development.	
**Concern that the outcome, its definition, assessment, or timing of assessment do not match the review question or the assessor's intended use**	**APPLICABILITY CONCERN:** *low/high/****unclear***
Outcome is binary and biased toward severe CKD, cannot answer the broader clinical question of fracture-risk stratification across CKD stages.	

**DOMAIN 4: Analysis**	
**A. Quality**	
30 patients producing 20,160 images split 70/10/20 by patient, independent validation 42 patients	
U-Net, BCE loss, XGBoost	
Segmentation: Dice, IoU, accuracy; classification: accuracy, precision, recall, F1, AUC-ROC, no calibration plot, no decision-curve analysis, per-image rather than per-patient performance reported	
Exclusion of 9 non-CKD participants and 5 CKD participants who had a visual grading score (VGS) for motion artifacts of three or above in at least one location.	
	**Y/ PY/ PN/ N/ NI**
**4.1 Was there evidence that the sample size was reasonable?**	PN
**4.2 Were continuous and categorical predictors handled appropriately?**	PY
**4.3 Were participants with missing or censored data handled appropriately in the analysis?**	PY
**4.4 If methods to address class imbalance were used, was the model or the model predictions recalibrated?***	NA
**4.5 Were methods used to address potential model overfitting?**	PY
**Concern regarding quality of the analysis**	**QUALITYCONCERN:** *low/****high****/unclear*
Patient-level splitting correct but only 30 patients and a strong image-level pseudo-replication, per-image metrics are reported, no calibration or decision-curve analysis.	

Model evaluation

Domain 1,2 and 3 same analysis as model development

**DOMAIN 4: Analysis**
**Risk of bias**
internal test 6 patients, independent validation 42 patients

DSC, IoU, accuracy, average Hausdorff Distance, precision, recall, F1-score, accuracy, AUROC
Describe any participants who were excluded from the analysis:<br>Exclusion of 9 non-CKD participants and 5 CKD participants who had a visual grading score (VGS) for motion artifacts of three or above in at least one location.
Describe missing data on predictors and outcomes as well as methods used for handling these missing data:<br>Missing data: complete-case patients, manual annotations by two independent experts.

	Y/ PY/ PN/ N/ NI/ NA		
**4.1 Was model evaluation based on only apparent performance avoided?**	Y		
	**A**	**I**	**E**
**4.2 Was there evidence that the sample size was reasonable?**	PN	PN	PN
**4.3 Were participants with missing or censored data handled appropriately in the analysis?**	NA	PY	PY
**4.4 If methods to address class imbalance were used, was the evaluation done in<br>a dataset without imbalance correction?***	NA	NA	NA
**4.5 If data splitting was done to create training and test datasets, was there evidence that data leakage was avoided?***	**NA**	Y	**NA**
**4.6 If resampling methods were used to evaluate model performance, were all<br>model development steps replicated in the resampling process?***	**NA**	PY	**NA**
**4.7 Was the predictive performance of the model evaluated appropriately, e.g., calibration, discrimination, and net benefit?**	PN	PN	PN
**Risk of bias introduced by the analysis**	**RISK OF BIAS:**<br>*low/**high**/unclear*		
Patient-level splitting and an independent same-institution validation cohort, tiny patient counts), absence of any inter-institution external validation, no calibration plot or decision-curve analysis, per-image rather than per-patient performance reporting.			

Step 4 - Assess the overall concerns regarding quality, risk of bias and applicability of the prediction model

OVERALL JUDGEMENT OF PREDICTION MODEL	
**Overall judgement of quality (development)**	**QUALITY CONCERN:**<br>*low/**high**/unclear*
High concern in D1 and D4, tiny pilot sample skewed to advanced CKD	
**Overall judgement of risk of bias (evaluation)**	**RISK OF BIAS:**<br>*low/**high**/unclear*
Independent same-institution validation only, no calibration or decision-curve analysis, per-image rather than per-patient metrics	
**Overall judgement of applicability (development)**	**APPLICABILITY CONCERN:**<br>*low/**high**/unclear*
Single US institution, single scanner, predominantly stage 5D CKD, HRpQCT not broadly available.	

**Overall judgement of applicability (evaluation)**	**APPLICABILITY CONCERN:** *low/**high**/unclear*
Same single-institution, advanced-CKD-skewed limitations.	

Jang et al. (44)

Step 1 - PICOTS

**Population**	220,018 adults, Korea; Internal validation: 74,380 (Severance Checkup Health Promotion Center); External validation: 62,945 KNHANES participants (nationwide Korean population, multistage clustered probability sample).
**Index model(s)**	Two XGBoost classifiers: eGFR60 model (eGFR<60) and eGFR45 model (eGFR<45).
**Comparator model(s)**	Prior literature using dipstick alone, no formal head-to-head model.
**Outcome(s)**	eGFR <60 or <45 mL/min/1.73 m² by CKD-EPI 2009 equation from serum creatinine measured on the same day.
**Timing**	Cross-sectional
**Setting and intended use of the prediction model**	Point-of-care screening in general practice or community settings, potential digital-urinalysis app to flag CKD without blood sampling

Step 2 – Classify the type of prediction model assessment

**Classify the assessment based on its aim**			
Type of prediction study	***Explanation***	***PROBAST+AI boxes to complete***	***Tick as appropriate***
**Development only**	**Prediction model development only, i.e., without evaluation of its performance.**	**Model development**	
**Evaluation only**	**External validation of one or more existing models in new data**	**Model evaluation**	
**Combination**	**Prediction model development combined in the same study (publication) with the evaluation of its apparent performance, internal validation performance, or external validation performance.**	**Model development and evaluation**	**X**

Step 3 - Assess quality and applicability or risk of bias and applicability

Publication reference	Jang EC, Park YM, Han HW, Lee CS, Kang ES, Lee YH, et al. Machine-learning enhance-ment of urine dipstick tests for chronic kidney disease detection. Journal of the American Medical Informatics Association. 2023;30(6):1114–24.
**Models of interest**	XGBoost eGFR60 and eGFR45 classifiers
**Outcome of interest**	Detection of eGFR <60 and <45 mL/min/1.73 m² (CKD stages G3a–G5) from urine dipstick + demographics.

Model development

DOMAIN 1: Participants and data sources	
A. Quality	
21 years of EHR from tertiary university hospital and diabetes centre, both inpatients and outpatients included, external validation in a checkup population and in the nationally representative KNHANES survey.	
	**Y/ PY/ PN/ N/ NI**
**1.1 Were appropriate data sources used?**	Y
**1.2 Was an appropriate study design used?**	Y
**1.3 Did the in- and exclusions of study participants result in a representative dataset?**	PY
**Concern regarding quality of selection of participants and data sources**	**QUALITYCONCERN:** ***low**/high/unclear*
Rationale of quality rating: Very large sample, multi-source, with two external validation cohorts including a nationally representative survey, inclusion of inpatients may introduce AKI-driven creatinine elevations.	
B. Applicability	
All data are from Korean adults, model is built and validated for routine community / point-of-care CKD screening using a standard urine dipstick.	
**Concern that the (data of the) included participants do not match the review question or the assessor's intended use of the prediction model**	**APPLICABILITY CONCERN:** ***low**/high/unclear*
Rationale of applicability rating: Population, sampling and intended use are aligned, restriction to Korean adults is the only material limitation for generalisability to other ethnicities.	

DOMAIN 2: Predictors	
A. Quality	
Predictors are age, sex and standard urine dipstick variables (protein, blood, glucose, pH, specific gravity, plus dropped leukocyte, bilirubin, urobilinogen, ketone, nitrite). Multiple analyser brands across centres, continuous variables standardised; ordinal levels coded.	
	**Y/ PY/ PN/ N/ NI**
**2.1 Were predictors defined and assessed in a similar way for all participants?**	PY
**2.2 Was any pre-processing of predictors similar for all participants?**	Y
**2.3 Were predictor assessments made without knowledge of outcome data?**	Y
**2.4 Were the predictors included in the model available at the time the model was intended to be used?**	Y

**Concern regarding quality of selection of participants and data sources**	**QUALITYCONCERN:** ***low****/high/unclear*
Dipstick predictors are simple, semi-quantitative and available at point-of-care, some between-centre device variation exists.	
**B. Applicability**	
**Concern that the definition, pre-processing, assessment, or timing of assessment of the predictors in the model do not match the review question or the assessor's intended use**	**APPLICABILITY CONCERN:** ***low****/high/unclear*
Rationale of applicability rating: Predictors match the routine clinical workflow exactly.	

**DOMAIN 3: Outcome**	
**A. Quality**	
Outcome is eGFR calculated by the CKD-EPI 2009 equation from same-day serum creatinine, subjects with multiple records keep the lowest eGFR.	
	**Y/ PY/ PN/ N/ NI**
**3.1 Were outcomes defined and assessed appropriately?**	Y
**3.2 Were outcomes defined and assessed in a similar way for all participants?**	Y
**3.3 Were outcome assessments made without use or knowledge of predictor data?**	Y
**3.4 Was the time interval between predictor assessment and outcome assessment appropriate?**	Y
**Concern regarding quality of the outcome or its determination**	**QUALITYCONCERN:** ***low****/high/unclear*
eGFR is standard CKD definition, computed identically across cohorts and independent of dipstick.	
**B. Applicability**	
Single-measurement eGFR is used rather than the KDIGO ≥3-month chronicity criterion.	
**Concern that the outcome, its definition, assessment, or timing of assessment do not match the review question or the assessor's intended use**	**APPLICABILITY CONCERN:** *low/high/****unclear***
A single-time eGFR cannot distinguish CKD from acute kidney injury, inclusion of inpatients in development means some labels could reflect AKI.	

**DOMAIN 4: Analysis**
**A. Quality**
198,015 train / 22,003 internal validation / 74,380 + 62,945 external validation.
GPU-accelerated TPOT Bayesian optimisation of 9 XGBoost hyperparameters, feature selection by importance from 12 to 7.
AUROC, sensitivity, specificity at Youden index no calibration SHAP-analysis, subgroup analyses by age, diabetes, hypertension, proteinuria.

Subjects with any missing values were deleted

	Y/ PY/ PN/ N/ NI
**4.1 Was there evidence that the sample size was reasonable?**	Y
**4.2 Were continuous and categorical predictors handled appropriately?**	Y
**4.3 Were participants with missing or censored data handled appropriately in the analysis?**	PN
**4.4 If methods to address class imbalance were used, was the model or the model predictions recalibrated?***	PN
**4.5 Were methods used to address potential model overfitting?**	Y
**Concern regarding quality of the analysis**	**QUALITYCONCERN:** *low/high/**unclear***
Very large sample, sensible feature selection, complete-case deletion of >40,000 records, absence of a calibration plot or decision-curve analysis.	

Model evaluation

Domain 1,2 and 3 same analysis as model development

**DOMAIN 4: Analysis**			
**Risk of bias**			
External validation 1 SCHPC n=74,380, external validation 2 KNHANES n=62,945.			
AUROC, sensitivity/specificity + threshold.			
Describe any participants who were excluded from the analysis: As development			
Describe missing data on predictors and outcomes as well as methods used for handling these missing data: Missing data: complete-case deletion across all sets.			
	**Y/ PY/ PN/ N/ NI/ NA**		
**4.1 Was model evaluation based on only apparent performance avoided?**	Y		
	**A**	**I**	**E**
**4.2 Was there evidence that the sample size was reasonable?**	NA	Y	Y
**4.3 Were participants with missing or censored data handled appropriately in the analysis?**	NA	PN	PN
**4.4 If methods to address class imbalance were used, was the evaluation done in a dataset without imbalance correction?***	NA	Y	Y
**4.5 If data splitting was done to create training and test datasets, was there evidence that data leakage was avoided?***	**NA**	Y	**NA**
**4.6 If resampling methods were used to evaluate model performance, were all model development steps replicated in the resampling process?***	**NA**	NA	**NA**
**4.7 Was the predictive performance of the model evaluated appropriately, e.g., calibration, discrimination, and net benefit?**	NA	PN	PN
**Risk of bias introduced by the analysis**	**RISK OF BIAS:** *low/high/**unclear***		

Discrimination is well characterised across, no calibration plot or decision-curve analysis is reported, missing data are handled by complete-case deletion, subgroup performance drops substantially in older and diabetic patients.

Step 4 - Assess the overall concerns regarding quality, risk of bias and applicability of the prediction model

OVERALL JUDGEMENT OF PREDICTION MODEL	
**Overall judgement of quality (development)**	**QUALITY CONCERN:**<br>***low***/*high/unclear*
D4 unclear, very large multi-source sample, feature selection, calibration absence keeps D4 unclear.	
**Overall judgement of risk of bias (evaluation)**	**RISK OF BIAS:**<br>*low/high/**unclear***
Strong discrimination in two large external sets, calibration and decision-curve analyses are missing, complete-case deletion was applied.	
**Overall judgement of applicability (development)**	**APPLICABILITY CONCERN:**<br>***low***/*high/unclear*
Population, predictors and outcome closely match the intended community/point-of-care CKD screening use case, restriction to Korean population.	
**Overall judgement of applicability (evaluation)**	**APPLICABILITY CONCERN:**<br>***low***/*high/unclear*
External validation in a general-population survey directly supports the intended use, reduced performance in diabetes and the elderly is acknowledged.	

Mavrogeorgis et al. (45)

Step 1 - PICOTS

**Population**	1,850 individuals from the Human Urinary Proteome Database
**Index model(s)**	Binary and multiclass SVM models.
**Comparator model(s)**	Single-aetiology SVM peptide models
**Outcome(s)**	Binary and multiclass classification of CKD aetiology.
**Timing**	Cross-sectional
**Setting and intended use of the prediction model**	Multi-centre European clinical research, intended as non-invasive liquid-biopsy adjunct/alternative to kidney biopsy for CKD aetiology.

Step 2 – Classify the type of prediction model assessment

**Classify the assessment based on its aim**			
Type of prediction study	***Explanation***	***PROBAST+AI boxes to complete***	***Tick as appropriate***

**Development only**	**Prediction model development only, i.e., without evaluation of its performance.**	**Model development**	
**Evaluation only**	**External validation of one or more existing models in new data**	**Model evaluation**	
**Combination**	**Prediction model development combined in the same study (publication) with the evaluation of its apparent performance, internal validation performance, or external validation performance.**	**Model development and evaluation**	**X**

Step 3 - Assess quality and applicability or risk of bias and applicability

**Publication reference**	Mavrogeorgis E, He TL, Mischak H, Latosinska A, Vlahou A, Schanstra JP, et al. Urinary peptidomic liquid biopsy for non-invasive differential diagnosis of chronic kidney dis-ease. Nephrology Dialysis Transplantation. 2024;39(3):453–62.
**Models of interest**	UMAP + SVM (binary and multiclass).
**Outcome of interest**	Non-invasive CKD aetiology classification.

Model development

**DOMAIN 1: Participants and data sources**	
**A. Quality**	
Convenience sample drawn from a single peptidome database aggregating multiple published European cohorts severe class imbalance, DKD largely clinical (only 6 biopsies); HC = no signs of kidney disease.	
	**Y/ PY/ PN/ N/ NI**
**1.1 Were appropriate data sources used?**	Y
**1.2 Was an appropriate study design used?**	PY
**1.3 Did the in- and exclusions of study participants result in a representative dataset?**	PN
**Concern regarding quality of selection of participants and data sources**	**QUALITYCONCERN:** *low/high/**unclear***
Rationale of quality rating: Reasonable overall size but heavy class, retrospective database-derived sample, and only 3 of possible CKD aetiologies included.	
**B. Applicability**	
Only four classes of the broad CKD aetiology spectrum are modelled, vasculitis cohort is very small and biopsy-confirmation status differs by class, all cohorts are European.	
**Concern that the (data of the) included participants do not match the review question or the assessor's intended use of the prediction model**	**APPLICABILITY CONCERN:** *low/**high**/unclear*

Rationale of applicability rating: Model cannot triage full clinically relevant range of CKD aetiologies and vasculitis class is underpowered.

**DOMAIN 2: Predictors**	
**A. Quality**	
Predictors are CE-MS urinary peptide features, with minimum-value imputation and z-score normalization, all samples processed at one specialist laboratory.	
	**Y/ PY/ PN/ N/ NI**
**2.1 Were predictors defined and assessed in a similar way for all participants?**	Y
**2.2 Was any pre-processing of predictors similar for all participants?**	Y
**2.3 Were predictor assessments made without knowledge of outcome data?**	Y
**2.4 Were the predictors included in the model available at the time the model was intended to be used?**	Y
**Concern regarding quality of selection of participants and data sources**	**QUALITYCONCERN:** ***low***/*high/unclear*
CE-MS urinary peptidomics is a highly specialised technology limited to a small number of laboratories.	
**B. Applicability**	
**Concern that the definition, pre-processing, assessment, or timing of assessment of the predictors in the model do not match the review question or the assessor's intended use**	**APPLICABILITY CONCERN:** *low/**high**/unclear*
Rationale of applicability rating: CE-MS is not routinely available, transportability to other labs/platforms is unverified, restricting model.	

**DOMAIN 3: Outcome**	
**A. Quality**	
Outcome classes use heterogeneous reference standards: IgAN and vasculitis biopsy-confirmed; DKD typically by clinical parameters, HC = no clinical/laboratory signs of kidney disease.	
	**Y/ PY/ PN/ N/ NI**
**3.1 Were outcomes defined and assessed appropriately?**	PY
**3.2 Were outcomes defined and assessed in a similar way for all participants?**	PN
**3.3 Were outcome assessments made without use or knowledge of predictor data?**	Y
**3.4 Was the time interval between predictor assessment and outcome assessment appropriate?**	Y
**Concern regarding quality of the outcome or its determination**	**QUALITYCONCERN:** *low/high/**unclear***
DKD label rests on clinical inference rather than histology, IgAN and vasculitis use biopsy, differential standards may bias multiclass classification.	
**B. Applicability**	
DKD label without biopsy may misclassify mixed aetiologies, and HC may not reflect a clinic-presenting comparator.	

Concern that the outcome, its definition, assessment, or timing of assessment do not match the review question or the assessor's intended use	APPLICABILITY CONCERN: *low/high/**unclear***
Outcome definitions are usable for proof of concept but label standard would improve applicability.	

DOMAIN 4: Analysis	
A. Quality	
1,038 (binary) or 1,850 (multiclass) participants. Features: 1,183 or 1,206 peptides, 75/25 train/test by participant, stratified; imbalance: SMOTE oversampling in the training data only.	
4-fold CV with Bayesian hyperparameter optimisation of UMAP + SVM	
Accuracy + class-wise confusion matrices in CV and test; no AUROC, no calibration plot, no decision-curve analysis.	
Excluded: NA. Missing data: handled by 30% frequency threshold and minimum-value imputation.	
	**Y/ PY/ PN/ N/ NI**
**4.1 Was there evidence that the sample size was reasonable?**	PY
**4.2 Were continuous and categorical predictors handled appropriately?**	PY
**4.3 Were participants with missing or censored data handled appropriately in the analysis?**	PY
**4.4 If methods to address class imbalance were used, was the model or the model predictions recalibrated?***	PN
**4.5 Were methods used to address potential model overfitting?**	Y
**Concern regarding quality of the analysis**	**QUALITYCONCERN:** *low/high/**unclear***
Nested CV with Bayesian hyperparameter optimisation, frequency-threshold imputation and SMOTE applied only to training data, vasculitis EPV is very low, only radial-basis SVM was assessed, no calibration or net-benefit analysis.	

Model evaluation

Domain 1,2 and 3 same analysis as model development

DOMAIN 4: Analysis
Risk of bias
UMAP+SVM multiclass test accuracy 70.13% overall, per-class 56.39% / 88.89% / 66.30% / 78.95%.
Accuracy + class-wise confusion matrices in CV and test, no AUROC, no calibration plot, no decision-curve analysis.
Describe any participants who were excluded from the analysis:<br>Not specifically stated
Describe missing data on predictors and outcomes as well as methods used for handling these missing data:<br>Missing data: handled by 30% frequency threshold and minimum-value imputation.

	Y/ PY/ PN/ N/ NI/ NA		
**4.1 Was model evaluation based on only apparent performance avoided?**	Y		
	**A**	**I**	**E**
**4.2 Was there evidence that the sample size was reasonable?**	NA	PN	NA
**4.3 Were participants with missing or censored data handled appropriately in the analysis?**	NA	PY	NA
**4.4 If methods to address class imbalance were used, was the evaluation done in a dataset without imbalance correction?***	NA	Y	NA
**4.5 If data splitting was done to create training and test datasets, was there evidence that data leakage was avoided?***	**NA**	Y	**NA**
**4.6 If resampling methods were used to evaluate model performance, were all model development steps replicated in the resampling process?***	**NA**	NA	**NA**
**4.7 Was the predictive performance of the model evaluated appropriately, e.g., calibration, discrimination, and net benefit?**	NA	PN	NA
**Risk of bias introduced by the analysis**	**RISK OF BIAS:** *low/**high**/unclear*		
Internal hold-out only, vasculitis class evaluated on only 19 test patients, no calibration or decision-curve analysis, substantial drop in performance versus the no-UMAP SVM (70% vs 85%) calls into question added value of UMAP for prediction.			

Step 4 - Assess the overall concerns regarding quality, risk of bias and applicability of the prediction model

OVERALL JUDGEMENT OF PREDICTION MODEL	
**Overall judgement of quality (development)**	**QUALITY CONCERN:** *low/**high**/unclear*
D1, D2, D4 unclear, severe vasculitis imbalance, differential outcome standards and no calibration/external validation.	
**Overall judgement of risk of bias (evaluation)**	**RISK OF BIAS:** *low/**high**/unclear*
Single internal test split, very small vasculitis cell, no calibration or decision-curve analysis.	
**Overall judgement of applicability (development)**	**APPLICABILITY CONCERN:** *low/**high**/unclear*
CE-MS not routinely available, only 4 of many CKD aetiologies, differential label standards limit clinical mapping.	
**Overall judgement of applicability (evaluation)**	**APPLICABILITY CONCERN:** *low/**high**/unclear*
Same as development.	

Wang et al. (46)

Step 1 - PICOTS

**Population**	Urine samples from healthy controls (G0) and CKD stages G1–G5, Chinese university hospital
**Index model(s)**	SERS spectra PCA / SVM / DT / KNN / RF and 5-fold CV

**Comparator model(s)**	Comparison against each other
**Outcome(s)**	Multi-class classification G0/G1/G2/G3/G4/G5 by KDIGO GFR thresholds.
**Timing**	Cross-sectional
**Setting and intended use of the prediction model**	Research, intended as a low-cost, rapid point-of-care SERS test for CKD screening and staging using the proposed aerogel platform.

Step 2 – Classify the type of prediction model assessment

**Classify the assessment based on its aim**			
Type of prediction study	***Explanation***	***PROBAST+AI boxes to complete***	***Tick as appropriate***
**Development only**	**Prediction model development only, i.e., without evaluation of its performance.**	**Model development**	
**Evaluation only**	**External validation of one or more existing models in new data**	**Model evaluation**	
**Combination**	**Prediction model development combined in the same study (publication) with the evaluation of its apparent performance, internal validation performance, or external validation performance.**	**Model development and evaluation**	**X**

Step 3 - Assess quality and applicability or risk of bias and applicability

**Publication reference**	Wang HN, Xu PP, Wei JR, Qiu LT, Zou J, Lin CL, et al. Urine collection by sodium algi-nate/CMC composite self-calibrating aerogel SERS platform for accurate screening and staging of chronic kidney disease. Int J Biol Macromol. 2025;302.
**Models of interest**	Five ML classifiers (PCA, SVM, DT, KNN, RF) on CS-Ag@4-mbn aerogel SERS spectra of urine.
**Outcome of interest**	Six-class KDIGO CKD staging (G0–G5) from urine SERS spectra.

Model development

**DOMAIN 1: Participants and data sources**
**A. Quality**

Small cohort with heavy class imbalance, no demographic or comorbidity description, recruitment site, ethnicity and inclusion criteria not detailed in the available text, no external validation	
	**Y/ PY/ PN/ N/ NI**
**1.1 Were appropriate data sources used?**	PY
**1.2 Was an appropriate study design used?**	PY
**1.3 Did the in- and exclusions of study participants result in a representative dataset?**	PN
**Concern regarding quality of selection of participants and data sources**	**QUALITYCONCERN:** *low/**high**/unclear*
Rationale of quality rating: Severe class imbalance and tiny per-stage patient numbers for G1–G4, no clinical-demographic characterization, no information on selection or inclusion criteria.	
**B. Applicability**	
Single-centre Chinese cohort with extreme imbalance, no demographic information, cohort does not support inferences across all stages and ethnicities.	
**Concern that the (data of the) included participants do not match the review question or the assessor's intended use of the prediction model**	**APPLICABILITY CONCERN:** *low/**high**/unclear*
Rationale of applicability rating: Single-centre, undocumented population with extreme imbalance and no external cohort, model cannot generalise to other settings or detect early-stage CKD.	

**DOMAIN 2: Predictors**	
**A. Quality**	
Predictors are SERS spectra acquired on CS-Ag@4-mbn aerogel platform, PCA used for dimensionality reduction.	
	**Y/ PY/ PN/ N/ NI**
**2.1 Were predictors defined and assessed in a similar way for all participants?**	Y
**2.2 Was any pre-processing of predictors similar for all participants?**	Y
**2.3 Were predictor assessments made without knowledge of outcome data?**	Y
**2.4 Were the predictors included in the model available at the time the model was intended to be used?**	Y
**Concern regarding quality of selection of participants and data sources**	**QUALITYCONCERN:** ***low**/high/unclear*
Internal-standard 4-mbn signal-corrected SERS with documented homogeneity, reproducibility and 50-day stability supports consistent predictor measurement.	
**B. Applicability**	
**Concern that the definition, pre-processing, assessment, or timing of assessment of the predictors in the model do not match the review question or the assessor's intended use**	**APPLICABILITY CONCERN:** *low/**high**/unclear*
Rationale of applicability rating: Laboratory-grade SERS substrate that requires a Raman spectrometer not realistic point-of-care infrastructure outside specialist labs.	

**DOMAIN 3: Outcome**

A. Quality	
Outcome is KDIGO 2012 GFR-based staging G1 (≥90), G2 (60–89), G3 (30–59), G4 (15–29), G5 (<15) plus a healthy G0 group, reference measurement not explicitly stated.	
	**Y/ PY/ PN/ N/ NI**
**3.1 Were outcomes defined and assessed appropriately?**	PY
**3.2 Were outcomes defined and assessed in a similar way for all participants?**	PY
**3.3 Were outcome assessments made without use or knowledge of predictor data?**	Y
**3.4 Was the time interval between predictor assessment and outcome assessment appropriate?**	PY
**Concern regarding quality of the outcome or its determination**	**QUALITYCONCERN:** *low/high/**unclear***
GFR thresholds are KDIGO-standard, but article does not state whether GFR is measured or estimated, nor whether the ≥3-month chronicity criterion was applied.	
**B. Applicability**	
KDIGO GFR-based staging is intended target.	
**Concern that the outcome, its definition, assessment, or timing of assessment do not match the review question or the assessor's intended use**	**APPLICABILITY CONCERN:** ***low**/high/unclear*
Outcome definition aligns with clinical CKD staging target.	

DOMAIN 4: Analysis	
**A. Quality**	
training G0/G1/G2/G3/G4/G5 = 200/28/20/24/24/200, test = 50/7/5/5/6/50	
Spectral normalisation with internal 4-mbn standard, PCA, 5-fold CV and grid search for SVM/DT/KNN/RF	
Accuracy, precision, recall, F1, AUROC, confusion matrices, no calibration plot, no decision-curve analysis.	
Excluded: NA. Missing data: NA	
	**Y/ PY/ PN/ N/ NI**
**4.1 Was there evidence that the sample size was reasonable?**	PN
**4.2 Were continuous and categorical predictors handled appropriately?**	PY
**4.3 Were participants with missing or censored data handled appropriately in the analysis?**	NI
**4.4 If methods to address class imbalance were used, was the model or the model predictions recalibrated?***	PN
**4.5 Were methods used to address potential model overfitting?**	PY
**Concern regarding quality of the analysis**	**QUALITYCONCERN:** *low/**high**/unclear*

Heavy class imbalance not corrected no confidence intervals, no calibration plot, no decision-curve analysis, no external validation.

Model evaluation

Domain 1,2 and 3 same analysis as model development

**DOMAIN 4: Analysis**			
**Risk of bias**			
Test 49 G0, 7 G1, 5 G2, 5 G3, 6 G4, 48 G5, extreme imbalance, G2 and G3 evaluated on only 5 events each.			
Accuracy, precision, recall, F1, AUROC, confusion matrices, no calibration plot, no decision-curve analysis.			
Describe any participants who were excluded from the analysis: Not stated			
Describe missing data on predictors and outcomes as well as methods used for handling these missing data: Not stated			
	**Y/ PY/ PN/ N/ NI/ NA**		
**4.1 Was model evaluation based on only apparent performance avoided?**	Y		
	**A**	**I**	**E**
**4.2 Was there evidence that the sample size was reasonable?**	NA	PN	NA
**4.3 Were participants with missing or censored data handled appropriately in the analysis?**	NA	NI	NA
**4.4 If methods to address class imbalance were used, was the evaluation done in a dataset without imbalance correction?***	NA	NA	NA
**4.5 If data splitting was done to create training and test datasets, was there evidence that data leakage was avoided?***	**NA**	NI	**NA**
**4.6 If resampling methods were used to evaluate model performance, were all model development steps replicated in the resampling process?***	**NA**	NA	**NA**
**4.7 Was the predictive performance of the model evaluated appropriately, e.g., calibration, discrimination, and net benefit?**	NA	PN	NA
**Risk of bias introduced by the analysis**	**RISK OF BIAS:** *low/**high**/unclear*		
Tiny per-stage test cells, extreme imbalance, evident overfitting in training, no calibration, no decision-curve analysis or no confidence intervals.			

Step 4 - Assess the overall concerns regarding quality, risk of bias and applicability of the prediction model

**OVERALL JUDGEMENT OF PREDICTION MODEL**	
**Overall judgement of quality (development)**	**QUALITY CONCERN:** *low/**high**/unclear*
D1 and D4 high, small imbalanced sample with weak analytic safeguards	
**Overall judgement of risk of bias (evaluation)**	**RISK OF BIAS:** *low/**high**/unclear*
Internal-only evaluation with extreme imbalance and tiny per-stage cells, potential leakage from repeated spectra, no calibration.	

Overall judgement of applicability (development)	APPLICABILITY CONCERN:<br>*low/**high**/unclear*
Single-centre Chinese pilot with SERS aerogel not available outside the lab.	
**Overall judgement of applicability (evaluation)**	**APPLICABILITY CONCERN:**<br>*low/**high**/unclear*
Same as development.	

Iftikhar et al. (47)

Step 1 - PICOTS

**Population**	382 patients, Pakistan, November 2020 – March 2021
**Index model(s)**	SVM with four kernels (linear, Laplacian, Bessel, radial basis) on 21 categorical / numerical features
**Comparator model(s)**	Logistic regression, probit, decision tree, k-nearest neighbour, random forest.
**Outcome(s)**	Binary disease status (CKD vs non-CKD), Medical Complex Buner diagnostic report.
**Timing**	Cross-sectional
**Setting and intended use of the prediction model**	Single Pakistani regional medical complex, intended low-cost, lab-based screening tool for CKD in developing-country settings.

Step 2 – Classify the type of prediction model assessment

Classify the assessment based on its aim			
Type of prediction study	***Explanation***	***PROBAST+AI boxes to complete***	***Tick as appropriate***
**Development only**	**Prediction model development only, i.e., without evaluation of its performance.**	**Model development**	
**Evaluation only**	**External validation of one or more existing models in new data**	**Model evaluation**	
**Combination**	**Prediction model development combined in the same study (publication) with the evaluation of its apparent performance, internal validation performance, or external validation performance.**	**Model development and evaluation**	**X**

Step 3 - Assess quality and applicability or risk of bias and applicability

**Publication reference**	Iftikhar H, Khan M, Khan Z, Khan F, Alshanbari HM, Ahmad Z. A Comparative Analysis of Machine Learning Models: A Case Study in Predicting Chronic Kidney Disease. Sus-tainability. 2023;15(3).
**Models of interest**	Logistic, probit, DT, KNN, SVM (4 kernels), RF.
**Outcome of interest**	Binary CKD vs non-CKD.

Model development

**DOMAIN 1: Participants and data sources**	
**A. Quality**	
Hospital case-control, Pakistan, 240 CKD / 142 non-CKD, diagnostic report from clinic nephrologist.	
	**Y/ PY/ PN/ N/ NI**
**1.1 Were appropriate data sources used?**	PY
**1.2 Was an appropriate study design used?**	PN
**1.3 Did the in- and exclusions of study participants result in a representative dataset?**	NI
**Concern regarding quality of selection of participants and data sources**	**QUALITYCONCERN:** *low/**high**/unclear*
Rationale of quality rating: Case-control design with a 63% CKD prevalence in the sample, no description of consecutive enrolment, exclusion criteria, or missing-data handling, single regional centre.	
**B. Applicability**	
Single Pakistani regional centre with case-control selection, cohort is heavily enriched for CKD, intended use as a screening tool for general/healthy populations	
**Concern that the (data of the) included participants do not match the review question or the assessor's intended use of the prediction model**	**APPLICABILITY CONCERN:** *low/**high**/unclear*
Rationale of applicability rating: Case-control case-mix poorly reflects screening / general-population CKD prevalence, geographically and demographically narrow	

**DOMAIN 2: Predictors**	
**A. Quality**	
21 routine diagnostic variables (mostly categorical urinalysis features + age, pH, specific gravity, gender, urine colour). Predictors collected at clinic check-up. No explicit pre-processing pipeline beyond model-by-model normalisation.	
	**Y/ PY/ PN/ N/ NI**
**2.1 Were predictors defined and assessed in a similar way for all participants?**	PY
**2.2 Was any pre-processing of predictors similar for all participants?**	PY
**2.3 Were predictor assessments made without knowledge of outcome data?**	NI
**2.4 Were the predictors included in the model available at the time the model**	Y

**was intended to be used?**	
**Concern regarding quality of selection of participants and data sources**	**QUALITYCONCERN:** *low/**high**/unclear*
Single-centre routine urinalysis is consistent within site, but laboratory reading of pus cells, RBC, casts and bacteria is operator-dependent and not blinded to clinical context, predictor reliability and measurement protocol not described.	
**B. Applicability**	
**Concern that the definition, pre-processing, assessment, or timing of assessment of the predictors in the model do not match the review question or the assessor's intended use**	**APPLICABILITY CONCERN:** ***low**/high/unclear*
Rationale of applicability rating: Routine urinalysis + demographics are universally accessible in primary-care settings.	

**DOMAIN 3: Outcome**	
**A. Quality**	
Binary CKD vs non-CKD from the clinical record, no description of diagnostic criteria (e.g. KDIGO eGFR or albuminuria thresholds), same-visit assessment.	
	**Y/ PY/ PN/ N/ NI**
**3.1 Were outcomes defined and assessed appropriately?**	PN
**3.2 Were outcomes defined and assessed in a similar way for all participants?**	PY
**3.3 Were outcome assessments made without use or knowledge of predictor data?**	PN
**3.4 Was the time interval between predictor assessment and outcome assessment appropriate?**	Y
**Concern regarding quality of the outcome or its determination**	**QUALITYCONCERN:** *low/**high**/unclear*
Outcome defined only as clinician-recorded CKD label without explicit KDIGO/eGFR criteria, predictors (urinalysis) directly inform clinical CKD diagnosis, creating circular outcome-from-predictor risk.	
**B. Applicability**	
Same-day binary CKD label without staging or progression	
**Concern that the outcome, its definition, assessment, or timing of assessment do not match the review question or the assessor's intended use**	**APPLICABILITY CONCERN:** *low/**high**/unclear*
Outcome is single binary label without severity stratification or prognostic horizon, does not align with the broader review question on CKD prognosis or stratification.	

**DOMAIN 4: Analysis**
**A. Quality**
240 CKD, 142 non-CKD, 21 predictors, pipeline includes nine models with multiple kernels and hyperparameters.
Three random train/test split scenarios (90/10, 75/25, 50/50), Diebold-Mariano test for accuracy comparison between models.

Accuracy, Brier score, sensitivity, specificity, Youden, F1. No discrimination AUC, no calibration plot, no decision-curve analysis.
Missing data not described

	Y/ PY/ PN/ N/ NI
**4.1 Was there evidence that the sample size was reasonable?**	PY
**4.2 Were continuous and categorical predictors handled appropriately?**	PY
**4.3 Were participants with missing or censored data handled appropriately in the analysis?**	NI
**4.4 If methods to address class imbalance were used, was the model or the model predictions recalibrated?***	N
**4.5 Were methods used to address potential model overfitting?**	PY
**Concern regarding quality of the analysis**	**QUALITYCONCERN:** *low/**high**/unclear*
Sample size reasonable for classical ML, repeated random splits do not constitute external validation, no calibration/AUROC/DCA, class imbalance moderate not addressed, no description of missing data.	

Model evaluation

Domain 1,2 and 3 same analysis as model development

**DOMAIN 4: Analysis**			
**Risk of bias**			
per-fold test sets of ~38 (10%), ~96 (25%), ~191 (50%) patients across 1000 random splits.			
Accuracy, Brier score, sensitivity, specificity, Youden, F1. No discrimination AUC, no calibration plot, no decision-curve analysis.			
Describe any participants who were excluded from the analysis: As in development			
Describe missing data on predictors and outcomes as well as methods used for handling these missing data: As in development			
	**Y/ PY/ PN/ N/ NI/ NA**		
**4.1 Was model evaluation based on only apparent performance avoided?**	PY		
	**A**	**I**	**E**
**4.2 Was there evidence that the sample size was reasonable?**	NA	PY	NA
**4.3 Were participants with missing or censored data handled appropriately in the analysis?**	NA	NI	NA
**4.4 If methods to address class imbalance were used, was the evaluation done in a dataset without imbalance correction?***	NA	NA	NA
**4.5 If data splitting was done to create training and test datasets, was there evidence that data leakage was avoided?***	**NA**	PY	**NA**
**4.6 If resampling methods were used to evaluate model performance, were all model development steps replicated in the resampling process?***	**NA**	PN	**NA**
**4.7 Was the predictive performance of the model evaluated appropriately, e.g., calibration, discrimination, and net benefit?**	NA	PN	NA

Risk of bias introduced by the analysis	RISK OF BIAS: *low/**high**/unclear*
Repeated hold-out splits document model stability, no calibration, discrimination AUC, or external generalisability.	

Step 4 - Assess the overall concerns regarding quality, risk of bias and applicability of the prediction model

OVERALL JUDGEMENT OF PREDICTION MODEL	
**Overall judgement of quality (development)**	**QUALITY CONCERN:** *low/**high**/unclear*
D1-D4 high, case-control design, ill-defined outcome, no calibration or AUROC.	
**Overall judgement of risk of bias (evaluation)**	**RISK OF BIAS:** *low/**high**/unclear*
Repeated internal random splits only, no AUROC/calibration/external validation.	
**Overall judgement of applicability (development)**	**APPLICABILITY CONCERN:** *low/**high**/unclear*
Single Pakistani regional centre with case-control case-mix, outcome binary clinician label.	
**Overall judgement of applicability (evaluation)**	**APPLICABILITY CONCERN:** *low/**high**/unclear*
Same as development.	

Kolachalama et al. (48)

Step 1 - PICOTS

**Population**	171 patients with native or allograft renal biopsy at Boston Medical Center
**Index model(s)**	Inception-v3 CNN; six models for CKD stage, high/low creatinine, nephrotic-range proteinuria, and 1/3/5-yr renal survival.
**Comparator model(s)**	Linear discriminant / Naïve Bayes / SVM classifiers using single pathologist-estimated fibrosis score (PEFS, 0–100) as input.
**Outcome(s)**	eGFR-based CKD stage (1–5, KDOQI); binarised creatinine (>1.3 male / >1.1 female mg/dL); nephrotic-range proteinuria (>3.5 g/d); renal survival at 1/3/5 yr (dialysis, transplant or all-cause mortality).
**Timing**	Biopsy at time-zero; clinical labs same day; survival across 1–7 years follow-up via chart review.
**Setting and intended use of the prediction model**	Single US urban academic centre (BMC) intended proof-of-principle for an app to support nephropathologic assessment of biopsies, particularly where expertise is scarce.

Step 2 – Classify the type of prediction model assessment

Classify the assessment based on its aim			
Type of prediction study	***Explanation***	***PROBAST+AI boxes to complete***	***Tick as appropriate***
**Development only**	**Prediction model development only, i.e., without evaluation of its performance.**	**Model development**	
**Evaluation only**	**External validation of one or more existing models in new data**	**Model evaluation**	
**Combination**	**Prediction model development combined in the same study (publication) with the evaluation of its apparent performance, internal validation performance, or external validation performance.**	**Model development and evaluation**	**X**

Step 3 - Assess quality and applicability or risk of bias and applicability

**Publication reference**	Kolachalama VB, Singh P, Lin CQ, Mun D, Belghasem ME, Henderson JM, et al. Associa-tion of Pathological Fibrosis With Renal Survival Using Deep Neural Networks. Kidney International Reports. 2018;3(2):464–75
**Models of interest**	Inception-v3 CNN (six task-specific models) vs PEFS-based linear classifier.
**Outcome of interest**	CKD stage, creatinine, proteinuria, 1/3/5-yr renal survival.

Model development

**DOMAIN 1: Participants and data sources**	
**A. Quality**	
Retrospective single-centre cohort, inclusion criterion: availability of pathological slide + accompanying clinical data, independent of biopsy indication.	
	**Y/ PY/ PN/ N/ NI**
**1.1 Were appropriate data sources used?**	Y
**1.2 Was an appropriate study design used?**	PY
**1.3 Did the in- and exclusions of study participants result in a representative dataset?**	PY
**Concern regarding quality of selection of participants and data sources**	**QUALITYCONCERN:** ***low**/high/unclear*
Rationale of quality rating: Single-centre retrospective cohort with detailed chart review and consecutive inclusion criterion, broad case-mix of native and transplant biopsies.	

B. Applicability	
Single urban US academic centre with high African American representation, CKD stages 3–5 over-represented, broad biopsy indications, intended use is decision support across nephropathology settings.	
**Concern that the (data of the) included participants do not match the review question or the assessor's intended use of the prediction model**	**APPLICABILITY CONCERN:** *low/**high**/unclear*
Rationale of applicability rating: Single-centre US cohort with high proportion of African American patients and CKD 3–5, transplant biopsies included, generalisability to other ethnicities, community CKD, predominantly diabetic cohorts and non-trichrome staining protocols is unproven.	

DOMAIN 2: Predictors	
**A. Quality**	
Digitised trichrome-stained biopsy slides, comparator predictor: single PEFS (0–100) by an in-house nephropathologist.	
	**Y/ PY/ PN/ N/ NI**
**2.1 Were predictors defined and assessed in a similar way for all participants?**	Y
**2.2 Was any pre-processing of predictors similar for all participants?**	Y
**2.3 Were predictor assessments made without knowledge of outcome data?**	PY
**2.4 Were the predictors included in the model available at the time the model was intended to be used?**	Y
**Concern regarding quality of selection of participants and data sources**	**QUALITYCONCERN:** ***low**/high/unclear*
Standardised imaging protocol and pre-processing pipeline, single pathologist for PEFS limits inter-observer assessment but ensures uniformity, predictors routinely obtained at time of biopsy.	
**B. Applicability**	
**Concern that the definition, pre-processing, assessment, or timing of assessment of the predictors in the model do not match the review question or the assessor's intended use**	**APPLICABILITY CONCERN:** ***low**/high/unclear*
Rationale of applicability rating: Trichrome staining and routine light microscopy are universally available in nephropathology, predictor is the same artefact already used for clinical reporting.	

DOMAIN 3: Outcome	
**A. Quality**	
Multiple outcomes from EMR: eGFR-based CKD stage (CKD-EPI), binarised serum creatinine, nephrotic-range proteinuria (>3.5 g/d), and renal survival to 1/3/5 yr (composite of dialysis initiation, transplant or all-cause mortality), chart review by team of nephrologists.	
	**Y/ PY/ PN/ N/ NI**
**3.1 Were outcomes defined and assessed appropriately?**	PY
**3.2 Were outcomes defined and assessed in a similar way for all participants?**	Y
**3.3 Were outcome assessments made without use or knowledge of predictor data?**	PY
**3.4 Was the time interval between predictor assessment and outcome assessment appropriate?**	PY

Concern regarding quality of the outcome or its determination	QUALITYCONCERN: ***low***/*high*/*unclear*
Standard laboratory and clinical-event outcomes from EMR chart review, composite survival outcome (death + dialysis + transplant) is heterogeneous but standard for renal-outcome research.	
**B. Applicability**	
Outcomes are routine clinical endpoints and CKD-staging indices, composite renal-survival is widely used.	
**Concern that the outcome, its definition, assessment, or timing of assessment do not match the review question or the assessor's intended use**	**APPLICABILITY CONCERN:** ***low***/*high*/*unclear*
Outcomes align directly with the review question.	

DOMAIN 4: Analysis	
**A. Quality**	
n = 171 patients, 2,255 images; survival sub-cohorts 158/146/117 patients for 1/3/5-yr. Class imbalance variable across tasks.	
Inception-v3 transfer learning with fine-tuning, 70:30 train/test split with cross-validation.	
Accuracy + Cohen κ (CKD stage), AUC + F1 + MCC (binary tasks). No calibration plot or decision-curve analysis.	
Complete-case approach (only patients with slides + clinical data included), survival sub-cohorts decreased over time horizons.	
	**Y/ PY/ PN/ N/ NI**
**4.1 Was there evidence that the sample size was reasonable?**	PN
**4.2 Were continuous and categorical predictors handled appropriately?**	Y
**4.3 Were participants with missing or censored data handled appropriately in the analysis?**	PN
**4.4 If methods to address class imbalance were used, was the model or the model predictions recalibrated?***	N
**4.5 Were methods used to address potential model overfitting?**	Y
**Concern regarding quality of the analysis**	**QUALITYCONCERN:** *low*/***high***/*unclear*
Sample size small relative to multi-task deep learning, class imbalance not addressed, strong overfitting controls (transfer learning, augmentation, CV, early stopping), no calibration or net-benefit analysis.	

Model evaluation

Domain 1,2 and 3 same analysis as model development

DOMAIN 4: Analysis
**Risk of bias**
~30% hold-out test image-level sets ranging 615–680 images (158/146/117 patients for survival tasks).

Accuracy + Cohen κ (CKD stage), AUC + F1 + MCC (binary tasks). No calibration plot or decision-curve analysis, no calibration, no DCA, no CI.
Describe any participants who were excluded from the analysis:<br>As in development
Describe missing data on predictors and outcomes as well as methods used for handling these missing data:<br>As in development

	**Y/ PY/ PN/ N/ NI/ NA**		
**4.1 Was model evaluation based on only apparent performance avoided?**	Y		
	**A**	**I**	**E**
**4.2 Was there evidence that the sample size was reasonable?**	NA	PN	NA
**4.3 Were participants with missing or censored data handled appropriately in the analysis?**	NA	PN	NA
**4.4 If methods to address class imbalance were used, was the evaluation done in a dataset without imbalance correction?***	NA	NA	NA
**4.5 If data splitting was done to create training and test datasets, was there evidence that data leakage was avoided?***	**NA**	PY	**NA**
**4.6 If resampling methods were used to evaluate model performance, were all model development steps replicated in the resampling process?***	**NA**	PY	**NA**
**4.7 Was the predictive performance of the model evaluated appropriately, e.g., calibration, discrimination, and net benefit?**	NA	PN	NA
**Risk of bias introduced by the analysis**	**RISK OF BIAS:**<br>*low/**high**/unclear*		
Evaluation is restricted to a 30% internal hold-out; no calibration, DCA, 95% CIs, or external validation.			

Step 4 - Assess the overall concerns regarding quality, risk of bias and applicability of the prediction model

**OVERALL JUDGEMENT OF PREDICTION MODEL**	
**Overall judgement of quality (development)**	**QUALITY CONCERN:**<br>*low/**high**/unclear*
D4 high, small sample, no calibration, no class imbalance correction	
**Overall judgement of risk of bias (evaluation)**	**RISK OF BIAS:**<br>*low/**high**/unclear*
Internal hold-out + CV only, no calibration, no external validation.	
**Overall judgement of applicability (development)**	**APPLICABILITY CONCERN:**<br>*low/**high**/unclear*
Single US centre with high African American representation and CKD 3–5 over-representation, transplant biopsies included.	
**Overall judgement of applicability (evaluation)**	**APPLICABILITY CONCERN:**<br>*low/**high**/unclear*
Same as development.	

Mendapara et al. (49)

Step 1 - PICOTS

**Population**	Four GEO microarray datasets pooled into a single discovery cohort of 248 kidney-tissue samples.
**Index model(s)**	Random forest on six signature genes: DUSP1, GADD45B, IFI30, IFI44L, ATF3, LYZ.
**Comparator model(s)**	Lasso-only model, intermediate 16-gene RF-RFE model, RF model excluding DUSP1.
**Outcome(s)**	Binary CKD vs non-CKD status
**Timing**	Cross-sectional gene expression at time of biopsy.
**Setting and intended use of the prediction model**	Public GEO microarray data, mixed centres. intended transcriptomic disease-risk classifier to aid CKD identification.

Step 2 – Classify the type of prediction model assessment

**Classify the assessment based on its aim**			
Type of prediction study	***Explanation***	***PROBAST+AI boxes to complete***	***Tick as appropriate***
**Development only**	**Prediction model development only, i.e., without evaluation of its performance.**	**Model development**	
**Evaluation only**	**External validation of one or more existing models in new data**	**Model evaluation**	
**Combination**	**Prediction model development combined in the same study (publication) with the evaluation of its apparent performance, internal validation performance, or external validation performance.**	**Model development and evaluation**	**X**

Step 3 - Assess quality and applicability or risk of bias and applicability

**Publication reference**	Mendapara K. Development and evaluation of a chronic kidney disease risk prediction model using random forest. Frontiers in Genetics. 2024;15.
**Models of interest**	RF on six DEGs; Lasso comparator.

Outcome of interest	Binary CKD vs non-CKD.

Model development

DOMAIN 1: Participants and data sources	
A. Quality	
Pooled GEO datasets 7/3 training/testin, external validation: GSE180394 dataset (mixed CKD vs healthy controls).	
	**Y/ PY/ PN/ N/ NI**
**1.1 Were appropriate data sources used?**	PY
**1.2 Was an appropriate study design used?**	PY
**1.3 Did the in- and exclusions of study participants result in a representative dataset?**	PN
**Concern regarding quality of selection of participants and data sources**	**QUALITYCONCERN:** *low/**high**/unclear*
Rationale of quality rating: Re-use of public microarrays from heterogeneous platforms with risk of residual batch confounding. detailed clinical phenotype, comorbidities and CKD stage not available at the patient level, control samples are few.	
B. Applicability	
Mixed GEO renal-tissue cohorts with diverse CKD aetiologies, cases pooled across platforms, intended use is transcriptomic CKD risk identification, applicable only when tissue gene-expression profiles are available.	
**Concern that the (data of the) included participants do not match the review question or the assessor's intended use of the prediction model**	**APPLICABILITY CONCERN:** *low/**high**/unclear*
Rationale of applicability rating: Predictor is invasive and not routinely available in clinical care, intended-use population is restricted to settings with research-grade tissue transcriptomics.	

DOMAIN 2: Predictors	
A. Quality	
35 DEGs , Lasso 10-fold CV reduced to 16 candidates, RF-RFE confirmed 1, final RF importance > 25 selected six genes (DUSP1, GADD45B, IFI30, IFI44L, ATF3, LYZ.	
	**Y/ PY/ PN/ N/ NI**
**2.1 Were predictors defined and assessed in a similar way for all participants?**	PY
**2.2 Was any pre-processing of predictors similar for all participants?**	Y
**2.3 Were predictor assessments made without knowledge of outcome data?**	N
**2.4 Were the predictors included in the model available at the time the model was intended to be used?**	PN
**Concern regarding quality of selection of participants and data sources**	**QUALITYCONCERN:** *low/**high**/unclear*
DEG selection performed using outcome labels on the same pooled cohort that is later split for training, feature-selection optimism	
B. Applicability	

**Concern that the definition, pre-processing, assessment, or timing of assessment of the predictors in the model do not match the review question or the assessor's intended use**	**APPLICABILITY CONCERN:** *low/**high**/unclear*
Rationale of applicability rating: Microarray gene expression on renal tissue is not feasible at the point of care, predictors require invasive biopsy + research transcriptomics.	

**DOMAIN 3: Outcome**	
**A. Quality**	
Binary CKD vs non-CKD status from the GEO submitter's annotation, no harmonised KDIGO/eGFR criteria across datasets, control definition varies (tumour-adjacent or healthy donors).	
	**Y/ PY/ PN/ N/ NI**
**3.1 Were outcomes defined and assessed appropriately?**	PN
**3.2 Were outcomes defined and assessed in a similar way for all participants?**	PN
**3.3 Were outcome assessments made without use or knowledge of predictor data?**	Y
**3.4 Was the time interval between predictor assessment and outcome assessment appropriate?**	Y
**Concern regarding quality of the outcome or its determination**	**QUALITYCONCERN:** *low/**high**/unclear*
Outcome label varies across original studies, harmonisation rests on submitter annotation and no unified diagnostic criteria, no CKD staging, eGFR threshold or progression endpoint.	
**B. Applicability**	
Binary CKD label assembled from multiple GEO studies with heterogeneous case definitions.	
**Concern that the outcome, its definition, assessment, or timing of assessment do not match the review question or the assessor's intended use**	**APPLICABILITY CONCERN:** *low/**high**/unclear*
Binary CKD vs non-CKD label lacks severity stratification, progression horizon, or harmonised diagnostic criteria.	

**DOMAIN 4: Analysis**	
**A. Quality**	
Training 174 (141 CKD / 33 controls), validation 74 (60/14); external 59 (50/9), 3:1 - 4:1 imbalance	
DEG selection with limma, Lasso (glmnet, 10-fold CV), RF-RFE.	
AUC + accuracy + sensitivity, precision, recall, F1 on validation, no calibration plot or DCA.	
Missing data: not described.	
	**Y/ PY/ PN/ N/ NI**
**4.1 Was there evidence that the sample size was reasonable?**	PY

**4.2 Were continuous and categorical predictors handled appropriately?**	Y
**4.3 Were participants with missing or censored data handled appropriately in the analysis?**	NI
**4.4 If methods to address class imbalance were used, was the model or the model predictions recalibrated?***	N
**4.5 Were methods used to address potential model overfitting?**	PY
**Concern regarding quality of the analysis**	**QUALITYCONCERN:** *low/**high**/unclear*
Repeated 10-fold CV plus an external dataset, applying ComBat to external dataset prior to testing introduces leakage, feature selection done on the full pooled cohort, class imbalance unaddressed, no calibration or DCA.	

Model evaluation

Domain 1,2 and 3 same analysis as model development

**DOMAIN 4: Analysis**			
**Risk of bias**			
Internal validation 74 patients (60/14); external GSE180394 59 patients (50/9).			
AUC + accuracy + sensitivity, precision, recall, F1 on validation, no calibration plot or DCA.			
Describe any participants who were excluded from the analysis: As in development			
Describe missing data on predictors and outcomes as well as methods used for handling these missing data: As in development			
	**Y/ PY/ PN/ N/ NI/ NA**		
**4.1 Was model evaluation based on only apparent performance avoided?**	Y		
	**A**	**I**	**E**
**4.2 Was there evidence that the sample size was reasonable?**	NA	PY	PY
**4.3 Were participants with missing or censored data handled appropriately in the analysis?**	NA	NI	NI
**4.4 If methods to address class imbalance were used, was the evaluation done in a dataset without imbalance correction?***	NA	NA	NA
**4.5 If data splitting was done to create training and test datasets, was there evidence that data leakage was avoided?***	**NA**	PN	**NA**
**4.6 If resampling methods were used to evaluate model performance, were all model development steps replicated in the resampling process?***	**NA**	PN	**NA**
**4.7 Was the predictive performance of the model evaluated appropriately, e.g., calibration, discrimination, and net benefit?**	NA	PN	PN
**Risk of bias introduced by the analysis**	**RISK OF BIAS:** *low/**high**/unclear*		
External validation, feature selection done on pooled training+validation, no calibration, no DCA, no CIs on external AUC.			

Step 4 - Assess the overall concerns regarding quality, risk of bias and applicability of the prediction model

OVERALL JUDGEMENT OF PREDICTION MODEL	
**Overall judgement of quality (development)**	**QUALITY CONCERN:**<br>*low/**high**/unclear*
D1-D4 high, feature-selection leakage, heterogeneous outcome labels.	
**Overall judgement of risk of bias (evaluation)**	**RISK OF BIAS:**<br>*low/**high**/unclear*
External validation done with batch correction across discovery + external sets, no calibration/DCA	
**Overall judgement of applicability (development)**	**APPLICABILITY CONCERN:**<br>*low/**high**/unclear*
Tissue-microarray predictor is not deployable at point of care; outcome label heterogeneous across studies.	
**Overall judgement of applicability (evaluation)**	**APPLICABILITY CONCERN:**<br>*low/**high**/unclear*
Same as development.	

Lee et al. (50)

Step 1 - PICOTS

**Population**	57 patients from the C-PROBE multicentre CKD cohort with diverse etiologies.
**Index model(s)**	Unsupervised K-means clustering of 256×256 patch feature vectors from ResNet-18/DeepLab V3+ encoder, summarised as a per-patient frequency histogram + 4th-order polynomial coefficients, random forest classifier on the resulting features + clinical (age, race, sex, diagnosis).
**Comparator model(s)**	Clinical features alone, sub-models using only histogram frequencies (F1+F2) or only polynomial coefficients (F3), top-7 feature subset selected by Gini importance.
**Outcome(s)**	Dichotomised eGFR at biopsy (≥60 vs <60 mL/min/1.73 m²), eGFR slope at 1 year (positive vs negative slope).
**Timing**	Cross-sectional histology at biopsy, eGFR slope computed from eGFR at 1 year vs at biopsy
**Setting and intended use of the prediction model**	Multicentre US CKD biobank (C-PROBE). Intended computer-aided, label-free identification of morphological features predictive of kidney function.

Step 2 – Classify the type of prediction model assessment

Classify the assessment based on its aim			
Type of prediction study	***Explanation***	***PROBAST+AI boxes to complete***	***Tick as appropriate***

**Development only**	**Prediction model development only, i.e., without evaluation of its performance.**	**Model development**	
**Evaluation only**	**External validation of one or more existing models in new data**	**Model evaluation**	
**Combination**	**Prediction model development combined in the same study (publication) with the evaluation of its apparent performance, internal validation performance, or external validation performance.**	**Model development and evaluation**	**X**

Step 3 - Assess quality and applicability or risk of bias and applicability

**Publication reference**	Lee J, Warner E, Shaikhouni S, Bitzer M, Kretzler M, Gipson D, et al. Unsupervised ma-chine learning for identifying important visual features through bag-of-words using his-topathology data from chronic kidney disease. Sci Rep. 2022;12(1).
**Models of interest**	Bag-of-words + K-means + random forest pipeline vs clinical-only models.
**Outcome of interest**	Dichotomised eGFR at biopsy and eGFR slope at 1 year.

Model development

**DOMAIN 1: Participants and data sources**	
**A. Quality**	
57 patients with CKD enrolled in the C-PROBE biobank with biopsy + 1-yr follow-up eGFR, ethnically diverse, diagnoses include lupus, diabetic/hypertensive nephropathy, minimal-change/FSGS, membranous, IgA/HSP, other GN, cortex manually annotated by a pathologist.	
	**Y/ PY/ PN/ N/ NI**
**1.1 Were appropriate data sources used?**	Y
**1.2 Was an appropriate study design used?**	PY
**1.3 Did the in- and exclusions of study participants result in a representative dataset?**	PN
**Concern regarding quality of selection of participants and data sources**	**QUALITYCONCERN:** *low/**high**/unclear*
Rationale of quality rating: Multicentre biobank with detailed phenotyping, analysis is restricted to only 57 patients, with no clear inclusion/exclusion description and class imbalance across diagnoses.	
**B. Applicability**	
US multicentre CKD biobank with broad etiologic mix but small sample, cortex annotation by a single pathologist, intended use is morphological discovery and prediction in routine CKD biopsy practice.	
**Concern that the (data of the) included participants do not match the review question or the assessor's intended use of the prediction model**	**APPLICABILITY CONCERN:** *low/**high**/unclear*

Rationale of applicability rating: Etiologically diverse cohort is a strength, lupus dominance limits generalisability to community CKD and to diabetic-nephropathy-dominated populations.

**DOMAIN 2: Predictors**	
**A. Quality**	
Whole-slide trichrome images tiled into 256×256 patches, ResNet-18 + DeepLab V3+ encoder used for feature extraction, K-means yields visual word frequencies, 4th-order polynomial fit gives 5 coefficients per case. Clinical features: age, race, sex, diagnosis.	
	**Y/ PY/ PN/ N/ NI**
**2.1 Were predictors defined and assessed in a similar way for all participants?**	Y
**2.2 Was any pre-processing of predictors similar for all participants?**	Y
**2.3 Were predictor assessments made without knowledge of outcome data?**	PY
**2.4 Were the predictors included in the model available at the time the model was intended to be used?**	Y
**Concern regarding quality of selection of participants and data sources**	**QUALITYCONCERN:** ***low****/high/unclear*
Standardised pre-processing, unsupervised clustering minimises label-bias, encoder fine-tuned with pathologist supervision but separately from main analysis cohort.	
**B. Applicability**	
**Concern that the definition, pre-processing, assessment, or timing of assessment of the predictors in the model do not match the review question or the assessor's intended use**	**APPLICABILITY CONCERN:** ***low****/high/unclear*
Rationale of applicability rating: Trichrome staining and whole-slide imaging are routinely available in academic nephropathology workflow.	

**DOMAIN 3: Outcome**	
**A. Quality**	
eGFR computed from serum creatinine using CKD-EPI, dichotomised at 60 mL/min/1.73 m² at biopsy eGFR slope: dichotomised at slope ≥0 vs <0.	
	**Y/ PY/ PN/ N/ NI**
**3.1 Were outcomes defined and assessed appropriately?**	Y
**3.2 Were outcomes defined and assessed in a similar way for all participants?**	Y
**3.3 Were outcome assessments made without use or knowledge of predictor data?**	Y
**3.4 Was the time interval between predictor assessment and outcome assessment appropriate?**	Y
**Concern regarding quality of the outcome or its determination**	**QUALITYCONCERN:** ***low****/high/unclear*
Standard CKD-EPI eGFR with established dichotomization, 1-year horizon is short relative to CKD natural history but appropriate for slope estimation.	
**B. Applicability**	
Dichotomised eGFR at biopsy and eGFR slope at 1 year are clinically meaningful outcomes.	

Concern that the outcome, its definition, assessment, or timing of assessment do not match the review question or the assessor's intended use	APPLICABILITY CONCERN: ***low***/*high*/*unclear*
Both outcomes align with the review question (CKD severity and short-term progression).	

DOMAIN 4: Analysis	
A. Quality	
N = 57 patients; 107,471 patches collapsed to 9 cluster-frequency features + 5 polynomial coefficients + 4 clinical features.	
Random forest classifier, top-7 features chosen by Gini importance	
AUROC + 95% CI, sensitivity, specificity, accuracy, no calibration, no decision-curve analysis.	
Missing data: not described, presumed complete-case	
	**Y/ PY/ PN/ N/ NI**
**4.1 Was there evidence that the sample size was reasonable?**	N
**4.2 Were continuous and categorical predictors handled appropriately?**	Y
**4.3 Were participants with missing or censored data handled appropriately in the analysis?**	NI
**4.4 If methods to address class imbalance were used, was the model or the model predictions recalibrated?***	NA
**4.5 Were methods used to address potential model overfitting?**	PY
**Concern regarding quality of the analysis**	**QUALITYCONCERN:** *low*/***high***/*unclear*
Small sample for a 9-cluster BoW + 4th-order polynomial pipeline, risk of overfitting despite cross-validation and top-7 feature subsetting, no calibration/DCA/no external validation, missing-data handling not described.	

Model evaluation

Domain 1,2 and 3 same analysis as model development

DOMAIN 4: Analysis
**Risk of bias**
57 patients, OOB sampling within RF, classification repeated 10 times.
AUROC + 95% CI, sensitivity, specificity, accuracy, no calibration, no decision-curve analysis.
Describe any participants who were excluded from the analysis: As in development
Describe missing data on predictors and outcomes as well as methods used for handling these missing data: As in development

	Y/ PY/ PN/ N/ NI/ NA		
**4.1 Was model evaluation based on only apparent performance avoided?**	Y		
	**A**	**I**	**E**
**4.2 Was there evidence that the sample size was reasonable?**	NA	N	NA
**4.3 Were participants with missing or censored data handled appropriately in the analysis?**	NA	NI	NA
**4.4 If methods to address class imbalance were used, was the evaluation done in a dataset without imbalance correction?***	NA	NA	NA
**4.5 If data splitting was done to create training and test datasets, was there evidence that data leakage was avoided?***	**NA**	PY	**NA**
**4.6 If resampling methods were used to evaluate model performance, were all model development steps replicated in the resampling process?***	**NA**	PN	**NA**
**4.7 Was the predictive performance of the model evaluated appropriately, e.g., calibration, discrimination, and net benefit?**	NA	PN	NA
**Risk of bias introduced by the analysis**	**RISK OF BIAS:** *low/**high**/unclear*		
RF is reasonable but full pipeline was not refit within each fold, raising data-leakage risk, no calibration/ DCA/external validation, class imbalance unaddressed.			

Step 4 - Assess the overall concerns regarding quality, risk of bias and applicability of the prediction model

OVERALL JUDGEMENT OF PREDICTION MODEL	
**Overall judgement of quality (development)**	**QUALITY CONCERN:** *low/**high**/unclear*
D1 and D4 high, small sample, missing-data ambiguity, no calibration.	
**Overall judgement of risk of bias (evaluation)**	**RISK OF BIAS:** *low/**high**/unclear*
Potential leakage from upstream clustering steps, no external validation.	
**Overall judgement of applicability (development)**	**APPLICABILITY CONCERN:** *low/**high**/unclear*
Etiologically diverse US biobank but n=57 with lupus dominance limits generalisability.	
**Overall judgement of applicability (evaluation)**	**APPLICABILITY CONCERN:** *low/**high**/unclear*
Same as development.	

Lee et al. (51)

Step 1 - PICOTS

**Population**	57 patients from the C-PROBE multicentre CKD cohort with diverse etiologies.
**Index model(s)**	DeepLab V3+/ResNet-18 feature extraction + K-means clustering + Deep Graph Convolutional Neural Network (DGCNN) on spatial graphs of cluster-coded patches, random forest classifier

	with frequency, polynomial-fit, clinical and area-weighted-spatial features.
**Comparator model(s)**	Sub-models without spatial information (visual frequency only, polynomial coefficients only, clinical only, individual feature sets, top-7).
**Outcome(s)**	Dichotomised eGFR at biopsy (≥60 vs <60 mL/min/1.73 m²), eGFR slope at 1 year (positive vs negative slope).
**Timing**	Cross-sectional histology at biopsy, eGFR slope computed from eGFR at 1 year vs at biopsy
**Setting and intended use of the prediction model**	Multicentre US CKD biobank (C-PROBE). Intended computer-aided, label-free identification of morphological features predictive of kidney function.

Step 2 – Classify the type of prediction model assessment

**Classify the assessment based on its aim**			
Type of prediction study	***Explanation***	***PROBAST+AI boxes to complete***	***Tick as appropriate***
**Development only**	**Prediction model development only, i.e., without evaluation of its performance.**	**Model development**	
**Evaluation only**	**External validation of one or more existing models in new data**	**Model evaluation**	
**Combination**	**Prediction model development combined in the same study (publication) with the evaluation of its apparent performance, internal validation performance, or external validation performance.**	**Model development and evaluation**	**X**

Step 3 - Assess quality and applicability or risk of bias and applicability

**Publication reference**	Lee J, Warner E, Shaikhouni S, Bitzer M, Kretzler M, Gipson D, et al. Clustering-based spa-tial analysis (CluSA) framework through graph neural network for chronic kidney disease prediction using histopathology images. Sci Rep. 2023;13(1).
**Models of interest**	CluSA = K-means + DGCNN + RF, vs sub-models without spatial features.
**Outcome of interest**	Dichotomised eGFR at biopsy and eGFR slope at 1 year.

Model development

DOMAIN 1: Participants and data sources	
**A. Quality**	
Same 57-patient C-PROBE cohort as Lee 2022 with additional cores (172 vs 161), ethnically diverse, broad diagnostic mix dominated by lupus nephritis, cortex annotated manually from WSI by a pathologist.	
	**Y/ PY/ PN/ N/ NI**
**1.1 Were appropriate data sources used?**	Y
**1.2 Was an appropriate study design used?**	PY
**1.3 Did the in- and exclusions of study participants result in a representative dataset?**	PN
**Concern regarding quality of selection of participants and data sources**	**QUALITYCONCERN:** *low/**high**/unclear*
Rationale of quality rating: Multicentre biobank with detailed phenotyping, analysis is restricted to only 57 patients, with no clear inclusion/exclusion description and class imbalance across diagnoses.	
**B. Applicability**	
US multicentre CKD biobank with broad etiologic mix but small sample, cortex annotation by a single pathologist, intended use is interpretable spatial-aware prediction of CKD function from routine biopsies.	
**Concern that the (data of the) included participants do not match the review question or the assessor's intended use of the prediction model**	**APPLICABILITY CONCERN:** *low/**high**/unclear*
Rationale of applicability rating: Etiologically diverse but lupus over-representation, generalisability to diabetic-nephropathy-dominated or non-US CKD populations unproven.	

DOMAIN 2: Predictors	
**A. Quality**	
Whole-slide trichrome images, tiled into 256×256 patches, DeepLab V3+ with ResNet-18 backbone pretrained on ImageNet, K-means clusters patches into nine 'visual words', each biopsy is encoded as a spatial graph of patches, fed to DGCNN. Random forest classifier on frequency, 4th-order polynomial coefficients, clinical and aw-scores from DGCNN.	
	**Y/ PY/ PN/ N/ NI**
**2.1 Were predictors defined and assessed in a similar way for all participants?**	Y
**2.2 Was any pre-processing of predictors similar for all participants?**	Y
**2.3 Were predictor assessments made without knowledge of outcome data?**	PY
**2.4 Were the predictors included in the model available at the time the model was intended to be used?**	Y
**Concern regarding quality of selection of participants and data sources**	**QUALITYCONCERN:** ***low**/high/unclear*
Standardised pipeline with stain normalisation, fixed tile size and pretrained encoder. K and feature engineering pre-specified before classification.	
**B. Applicability**	
**Concern that the definition, pre-processing, assessment, or timing of assessment of the predictors in the model do not match the review question or the assessor's intended use**	**APPLICABILITY CONCERN:** ***low**/high/unclear*

Rationale of applicability rating: Trichrome staining and whole-slide imaging are routinely available in academic nephropathology workflow.

**DOMAIN 3: Outcome**	
**A. Quality**	
eGFR computed from serum creatinine using CKD-EPI, dichotomised at 60 mL/min/1.73 m² at biopsy<br>eGFR slope: dichotomised at slope ≥0 vs <0.	
	**Y/ PY/ PN/ N/ NI**
**3.1 Were outcomes defined and assessed appropriately?**	Y
**3.2 Were outcomes defined and assessed in a similar way for all participants?**	Y
**3.3 Were outcome assessments made without use or knowledge of predictor data?**	Y
**3.4 Was the time interval between predictor assessment and outcome assessment appropriate?**	Y
**Concern regarding quality of the outcome or its determination**	**QUALITYCONCERN:** ***low****/high/unclear*
Standard CKD-EPI eGFR with established dichotomization, 1-year horizon is short relative to CKD natural history but appropriate for slope estimation.	
**B. Applicability**	
Dichotomised eGFR at biopsy and eGFR slope at 1 year are clinically meaningful outcomes.	
**Concern that the outcome, its definition, assessment, or timing of assessment do not match the review question or the assessor's intended use**	**APPLICABILITY CONCERN:** ***low****/high/unclear*
Both outcomes align with the review question (CKD severity and short-term progression).	

**DOMAIN 4: Analysis**	
**A. Quality**	
N = 57 patients; 107,471 patches collapsed to 9 visual words features + 5 polynomial coefficients + aw-scores	
3-fold cross-validation stratified by patient, DGCNN with 4 graph-convolutional layers, RF classifier with various feature subsets and top-7 selection by Gini importance.	
AUROC + 95% CI, accuracy, confusion matrices, no calibration plot, no DCA	
Missing data: not described, presumed complete-case	
	**Y/ PY/ PN/ N/ NI**
**4.1 Was there evidence that the sample size was reasonable?**	N
**4.2 Were continuous and categorical predictors handled appropriately?**	Y
**4.3 Were participants with missing or censored data handled appropriately in the analysis?**	NI

**4.4 If methods to address class imbalance were used, was the model or the model predictions recalibrated?***	NA
**4.5 Were methods used to address potential model overfitting?**	PY
**Concern regarding quality of the analysis**	**QUALITYCONCERN:** *low/**high**/unclear*
Three-fold CV, small sample to a DGCNN + RF ensemble with multi-modal features, no calibration/DCA /external validation, missing data not described.	

Model evaluation

Domain 1,2 and 3 same analysis as model development

**DOMAIN 4: Analysis**			
**Risk of bias**			
Three folds of ~19 patients each, per-fold class counts not reported.			
AUROC + 95% CI, accuracy, confusion matrices, no calibration plot, no DCA			
Describe any participants who were excluded from the analysis: As in development			
Describe missing data on predictors and outcomes as well as methods used for handling these missing data: As in development			
	**Y/ PY/ PN/ N/ NI/ NA**		
**4.1 Was model evaluation based on only apparent performance avoided?**	Y		
	**A**	**I**	**E**
**4.2 Was there evidence that the sample size was reasonable?**	NA	N	NA
**4.3 Were participants with missing or censored data handled appropriately in the analysis?**	NA	NI	NA
**4.4 If methods to address class imbalance were used, was the evaluation done in a dataset without imbalance correction?***	NA	NA	NA
**4.5 If data splitting was done to create training and test datasets, was there evidence that data leakage was avoided?***	**NA**	PY	**NA**
**4.6 If resampling methods were used to evaluate model performance, were all model development steps replicated in the resampling process?***	**NA**	PN	**NA**
**4.7 Was the predictive performance of the model evaluated appropriately, e.g., calibration, discrimination, and net benefit?**	NA	PN	NA
**Risk of bias introduced by the analysis**	**RISK OF BIAS:** *low/**high**/unclear*		
3-fold CV, partial leakage is possible, no calibration, no DCA, no external validation.			

Step 4 - Assess the overall concerns regarding quality, risk of bias and applicability of the prediction model

**OVERALL JUDGEMENT OF PREDICTION MODEL**	
**Overall judgement of quality (development)**	**QUALITY CONCERN:** *low/**high**/unclear*

D1 and D4 high, small sample, missing-data ambiguity, no calibration.	
**Overall judgement of risk of bias (evaluation)**	**RISK OF BIAS:**<br>*low/**high**/unclear*
Patient-stratified 3-fold CV good, partial leakage from upstream clustering, no external validation.	
**Overall judgement of applicability (development)**	**APPLICABILITY CONCERN:**<br>*low/**high**/unclear*
Etiologically diverse US biobank but n=57 with lupus dominance limits generalisability.	
**Overall judgement of applicability (evaluation)**	**APPLICABILITY CONCERN:**<br>*low/**high**/unclear*
Same as development.	

Metherall et al. (52)

Step 1 - PICOTS

**Population**	400 patients from publicly available UCI CKD dataset, 383 patients with serum creatinine retained after pruning. 25 input features
**Index model(s)**	ANN and Random Forest
**Comparator model(s)**	ANN vs RF across the three feature subsets (at-home, monitoring, laboratory).
**Outcome(s)**	Binary CKD vs non-CKD (KDOQI-based label), log-serum creatinine.
**Timing**	Cross-sectional
**Setting and intended use of the prediction model**	Public UCI dataset, intended use is at-home / online CKD screening using reduced feature sets accessible to patients in their homes.

Step 2 – Classify the type of prediction model assessment

**Classify the assessment based on its aim**			
Type of prediction study	***Explanation***	***PROBAST+AI boxes to complete***	***Tick as appropriate***
**Development only**	**Prediction model development only, i.e., without evaluation of its performance.**	**Model development**	
**Evaluation only**	**External validation of one or more existing models in new data**	**Model evaluation**	

**Combination**	**Prediction model development combined in the same study (publication) with the evaluation of its apparent performance, internal validation performance, or external validation performance.**	**Model development and evaluation**	**X**

Step 3 - Assess quality and applicability or risk of bias and applicability

**Publication reference**	Metherall B, Berryman AK, Brennan GS. Machine learning for classifying chronic kidney disease and predicting creatinine levels using at-home measurements. Sci Rep. 2025;15(1):4364.
**Models of interest**	ANN and RF across three nested feature sets.
**Outcome of interest**	CKD classification and log-creatinine regression.

Model development

**DOMAIN 1: Participants and data sources**	
**A. Quality**	
Publicly available UCI CKD dataset, 17 patients excluded for missing serum creatinine, sex and race added from a separate UCI source, labels assigned by hospital nephrologists from patient history, symptoms, blood and urine tests.	
	**Y/ PY/ PN/ N/ NI**
**1.1 Were appropriate data sources used?**	PY
**1.2 Was an appropriate study design used?**	PY
**1.3 Did the in- and exclusions of study participants result in a representative dataset?**	PN
**Concern regarding quality of selection of participants and data sources**	**QUALITYCONCERN:** *low/**high**/unclear*
Rationale of quality rating: Use of single-hospital dataset, CKD stage not provided.	
**B. Applicability**	
Public dataset used for at-home screening application, CKD-enriched case-mix, intended use is screening of a general population using a smartphone/at-home tool.	
**Concern that the (data of the) included participants do not match the review question or the assessor's intended use of the prediction model**	**APPLICABILITY CONCERN:** *low/**high**/unclear*
Rationale of applicability rating: Source population and intended use are mismatched, demographics narrow, performance unlikely to generalise to a low-prevalence at-home setting.	

**DOMAIN 2: Predictors**
**A. Quality**

25 predictors classified by accessibility: at-home (age, race, sex, hypertension, diabetes, coronary artery disease, appetite, pedal edema, anaemia, blood pressure), monitoring (adds red/white blood cell counts, blood glucose, blood urea, sodium, potassium, haemoglobin), laboratory (adds specific gravity, albumin, sugar, bacteria, pus cell, pus cell clumps, packed cell volume, 5-NN imputation for missing numerical features.	
	**Y/ PY/ PN/ N/ NI**
**2.1 Were predictors defined and assessed in a similar way for all participants?**	PY
**2.2 Was any pre-processing of predictors similar for all participants?**	Y
**2.3 Were predictor assessments made without knowledge of outcome data?**	NI
**2.4 Were the predictors included in the model available at the time the model was intended to be used?**	PN
**Concern regarding quality of selection of participants and data sources**	**QUALITYCONCERN:** *low/**high**/unclear*
Predictor protocols not documented,'at-home' features include blood-pressure and 'anaemia' label, latter not strictly measurable at home, predictor blinding to outcome unclear.	
**B. Applicability**	
**Concern that the definition, pre-processing, assessment, or timing of assessment of the predictors in the model do not match the review question or the assessor's intended use**	**APPLICABILITY CONCERN:** *low/**high**/unclear*
Rationale of applicability rating: Several 'at-home' features (anaemia status, pedal edema, appetite) are clinician-defined rather than self-reported, undermining the at-home applicability narrative.	

**DOMAIN 3: Outcome**	
**A. Quality**	
Binary CKD label assigned by nephrologists based on history, symptoms, blood and urine tests. Serum creatinine: continuous secondary outcome used to estimate GFR via CKD-EPI.	
	**Y/ PY/ PN/ N/ NI**
**3.1 Were outcomes defined and assessed appropriately?**	PN
**3.2 Were outcomes defined and assessed in a similar way for all participants?**	PY
**3.3 Were outcome assessments made without use or knowledge of predictor data?**	N
**3.4 Was the time interval between predictor assessment and outcome assessment appropriate?**	Y
**Concern regarding quality of the outcome or its determination**	**QUALITYCONCERN:** *low/**high**/unclear*
Outcome is derived using the same blood/urine tests that are also predictors, predictor-outcome circularity, no harmonised KDIGO criteria reported, no CKD stage.	
**B. Applicability**	
Binary CKD label assigned at single hospital visit, no progression horizon or staging.	
**Concern that the outcome, its definition, assessment, or timing of assessment do not match the review question or the assessor's intended use**	**APPLICABILITY CONCERN:** *low/**high**/unclear*
Outcome is a binary clinician label, does not match the review question of CKD severity or progression.	

DOMAIN 4: Analysis	
A. Quality	
N=383 after pruning, 25 numerical/categorical features	
ANN and RF, 10-fold outer cross-validation.	
Classification: accuracy, TPR, TNR, FPR, FNR, AUROC; regression: MSE, R^2, MAE; no calibration plot or DCA.	
Missing data: 5-NN imputation for numerical features, missing class added in one-hot for nominal features.	
	**Y/ PY/ PN/ N/ NI**
**4.1 Was there evidence that the sample size was reasonable?**	PY
**4.2 Were continuous and categorical predictors handled appropriately?**	PY
**4.3 Were participants with missing or censored data handled appropriately in the analysis?**	PY
**4.4 If methods to address class imbalance were used, was the model or the model predictions recalibrated?***	NA
**4.5 Were methods used to address potential model overfitting?**	Y
**Concern regarding quality of the analysis**	**QUALITYCONCERN:** *low/**high**/unclear*
Solid methodology the predictor-outcome circularity and dataset prevalence mismatch with intended use, no calibration/DCA, imputation done globally before CV.	

Model evaluation

Domain 1,2 and 3 same analysis as model development

DOMAIN 4: Analysis			
Risk of bias			
10 folds × ~38 patients each, consistent class balance preserved.			
Classification: accuracy, TPR, TNR, FPR, FNR, AUROC; regression: MSE, R^2, MAE; no calibration plot or DCA.			
Describe any participants who were excluded from the analysis: As in development			
Describe missing data on predictors and outcomes as well as methods used for handling these missing data: 5-NN imputation done before CV split			
	**Y/ PY/ PN/ N/ NI/ NA**		
**4.1 Was model evaluation based on only apparent performance avoided?**	Y		
	**A**	**I**	**E**
**4.2 Was there evidence that the sample size was reasonable?**	NA	PY	NA

**4.3 Were participants with missing or censored data handled appropriately in the analysis?**	NA	PY	NA
**4.4 If methods to address class imbalance were used, was the evaluation done in a dataset without imbalance correction?***	NA	NA	NA
**4.5 If data splitting was done to create training and test datasets, was there evidence that data leakage was avoided?***	**NA**	PN	**NA**
**4.6 If resampling methods were used to evaluate model performance, were all model development steps replicated in the resampling process?***	**NA**	PN	**NA**
**4.7 Was the predictive performance of the model evaluated appropriately, e.g., calibration, discrimination, and net benefit?**	NA	PN	NA
**Risk of bias introduced by the analysis**	**RISK OF BIAS:** *low/**high**/unclear*		
10-fold CV with mean/SD reporting, imputation and standardisation done on the full dataset before splitting, no calibration/DCA/external validation, dataset's high CKD prevalence inflates absolute accuracy for at-home intended use.			

Step 4 - Assess the overall concerns regarding quality, risk of bias and applicability of the prediction model

**OVERALL JUDGEMENT OF PREDICTION MODEL**	
**Overall judgement of quality (development)**	**QUALITY CONCERN:** *low/**high**/unclear*
D1-D4 high, outcome circularity, mismatched prevalence, no calibration.	
**Overall judgement of risk of bias (evaluation)**	**RISK OF BIAS:** *low/**high**/unclear*
10-fold CV only, imputation pre-split, no external validation.	
**Overall judgement of applicability (development)**	**APPLICABILITY CONCERN:** *low/**high**/unclear*
Single hospital dataset for general at-home screening, 'at-home' features include clinician-defined items.	
**Overall judgement of applicability (evaluation)**	**APPLICABILITY CONCERN:** *low/**high**/unclear*
Same as development.	

Bhatt et al. (53)

Step 1 - PICOTS

**Population**	Patient urine samples. Five samples covering normal (<20), microalbuminuria (20–200) and macroalbuminuria (>200 mg/L) concentrations.
**Index model(s)**	KNN
**Comparator model(s)**	LR/SVM/RF
**Outcome(s)**	5-class urinary-albumin concentration
**Timing**	Cross-sectional.
**Setting and intended use of the prediction model**	Home-monitoring use, intended for community CKD screening with a standard smartphone and no auxiliary attachment

Step 2 – Classify the type of prediction model assessment

**Classify the assessment based on its aim**			
Type of prediction study	***Explanation***	***PROBAST+AI boxes to complete***	***Tick as appropriate***
**Development only**	**Prediction model development only, i.e., without evaluation of its performance.**	**Model development**	
**Evaluation only**	**External validation of one or more existing models in new data**	**Model evaluation**	
**Combination**	**Prediction model development combined in the same study (publication) with the evaluation of its apparent performance, internal validation performance, or external validation performance.**	**Model development and evaluation**	**X**

Step 3 - Assess quality and applicability or risk of bias and applicability

**Publication reference**	Bhatt S, Kumar S, Gupta MK, Datta SK, Dubey SK. Colorimetry-based and smartphone-assisted machine-learning model for quantification of urinary albumin. Measurement Science and Technology. 2024;35(1).
**Models of interest**	KNN
**Outcome of interest**	5-class urinary-albumin concentration

Model development

DOMAIN 1: Participants and data sources	
A. Quality	
Five patient urine samples spanning the clinically meaningful concentration range, each sample imaged on a chemically impregnated dipstick under 12 illumination conditions, using 4 smartphones with Flash ON / OFF.	
	**Y/ PY/ PN/ N/ NI**
**1.1 Were appropriate data sources used?**	PN
**1.2 Was an appropriate study design used?**	PY
**1.3 Did the in- and exclusions of study participants result in a representative dataset?**	N
**Concern regarding quality of selection of participants and data sources**	**QUALITYCONCERN:** *low/**high**/unclear*
Rationale of quality rating: Only 5 distinct urine samples, repeated imaging inflates sample size, dataset is matrix-controlled rather than population-based, representativeness of the CKD target population is not demonstrated.	
B. Applicability	
Sensors, Instrumentation and Cyber Physical System Engineering laboratory, IIT Delhi, urine samples, sample characteristics not reported, dipsticks: Siemens-style.	
**Concern that the (data of the) included participants do not match the review question or the assessor's intended use of the prediction model**	**APPLICABILITY CONCERN:** *low/**high**/unclear*
Rationale of applicability rating: Population, demographics and clinical context not described, only five biological samples, limited generalisability to real patient distributions of urinary protein, colour, and interfering substances	

DOMAIN 2: Predictors	
A. Quality	
mean R, G, B (RGB); H, S, V (HSV); L, a, b (Lab) colour features extracted from a manually-segmented albumin-reaction patch on the dipstick, random search used to select the best feature subset across illumination conditions and smartphone models, smartphone flash used to compensate shadow.	
	**Y/ PY/ PN/ N/ NI**
**2.1 Were predictors defined and assessed in a similar way for all participants?**	PY
**2.2 Was any pre-processing of predictors similar for all participants?**	PY
**2.3 Were predictor assessments made without knowledge of outcome data?**	Y
**2.4 Were the predictors included in the model available at the time the model was intended to be used?**	Y
**Concern regarding quality of selection of participants and data sources**	**QUALITYCONCERN:** ***low**/high/unclear*
Standardised feature extraction pipeline applied identically across images, predictors assessed without knowledge of label, flash-ON improves repeatability and is the chosen operating mode.	
B. Applicability	
**Concern that the definition, pre-processing, assessment, or timing of assessment of the predictors in the model do not match the review question or the assessor's intended use**	**APPLICABILITY CONCERN:** ***low**/high/unclear*

Rationale of applicability rating: Colour features from a smartphone-captured dipstick are obtainable in any point-of-care setting

**DOMAIN 3: Outcome**	
**A. Quality**	
Albumin concentration class (4, 20, 100, 300, 1270 mg/L), reference measurement performed before / on the same day as smartphone imaging.	
Or	**Y/ PY/ PN/ N/ NI**
**3.1 Were outcomes defined and assessed appropriately?**	Y
**3.2 Were outcomes defined and assessed in a similar way for all participants?**	Y
**3.3 Were outcome assessments made without use or knowledge of predictor data?**	Y
**3.4 Was the time interval between predictor assessment and outcome assessment appropriate?**	Y
**Concern regarding quality of the outcome or its determination**	**QUALITYCONCERN:** ***low****/high/unclear*
Validated laboratory reference standards, outcome assessed independently of the smartphone images.	
**B. Applicability**	
Outcome determined by laboratory assay on the same urine sample as imaging, class boundaries chosen to span clinical decision thresholds.	
**Concern that the outcome, its definition, assessment, or timing of assessment do not match the review question or the assessor's intended use**	**APPLICABILITY CONCERN:** ***low****/high/unclear*
Discrete concentration classes are clinically meaningful, continuous-concentration prediction would be more clinically informative.	

**DOMAIN 4: Analysis**	
**A. Quality**	
240 images / 960 images / 1920 images from only 5 distinct urine samples. 5-class classification, 70/30 training/testing	
ROI extraction → colour features → feature selection by random search → classifiers: LR, SVM, RF, KNN. Smartphone Flash ON used to mitigate ambient-light variability, experiment with separate train/test smartphones to test inter-phone transferability.	
Accuracy, precision, recall, F1, confusion matrices, t-SNE for class separability visualisation.	
Missing data: none reported	
	**Y/ PY/ PN/ N/ NI**
**4.1 Was there evidence that the sample size was reasonable?**	PN
**4.2 Were continuous and categorical predictors handled appropriately?**	PY
**4.3 Were participants with missing or censored data handled appropriately in the analysis?**	NI

**4.4 If methods to address class imbalance were used, was the model or the model predictions recalibrated?***	NI
**4.5 Were methods used to address potential model overfitting?**	PN
**Concern regarding quality of the analysis**	**QUALITYCONCERN:** *low/**high**/unclear*
Sample size inadequate for stable classifier training and almost certainly drives high reported accuracies, train/test split is image-level not sample-level, same urine sample's images can appear in both, high risk of data leakage and over-optimistic performance, no calibration/DCA/external validation.	

Model evaluation

Domain 1,2 and 3 same analysis as model development

**DOMAIN 4: Analysis**			
**Risk of bias**			
Experiment 1: 240 images, 168 train / 72 test (image-level split). Experiment 2: 960, 672 / 288. Experiment 3: 1920, 1344 / 576.			
Accuracy, precision, recall, F1, confusion matrices, t-SNE for class separability visualisation.			
Describe any participants who were excluded from the analysis: Exclusion same as development			
Describe missing data on predictors and outcomes as well as methods used for handling these missing data: Missing data: not mentioned			
	**Y/ PY/ PN/ N/ NI/ NA**		
**4.1 Was model evaluation based on only apparent performance avoided?**	Y		
	**A**	**I**	**E**
**4.2 Was there evidence that the sample size was reasonable?**	NA	PN	NA
**4.3 Were participants with missing or censored data handled appropriately in the analysis?**	NA	NA	NA
**4.4 If methods to address class imbalance were used, was the evaluation done in a dataset without imbalance correction?***	NA	NA	NA
**4.5 If data splitting was done to create training and test datasets, was there evidence that data leakage was avoided?***	**NA**	PN	**NA**
**4.6 If resampling methods were used to evaluate model performance, were all model development steps replicated in the resampling process?***	**NA**	NA	**NA**
**4.7 Was the predictive performance of the model evaluated appropriately, e.g., calibration, discrimination, and net benefit?**	NA	PN	NA
**Risk of bias introduced by the analysis**	**RISK OF BIAS:** *low/**high**/unclear*		
Image-level random split across only 5 biological samples, almost certainly causes data leakage and over-optimistic accuracy estimates, cross-colour-temperature performance drop to 65% shows fragility, no calibration, no external validation, sample of convenience.			

Step 4 - Assess the overall concerns regarding quality, risk of bias and applicability of the prediction model

OVERALL JUDGEMENT OF PREDICTION MODEL	
**Overall judgement of quality (development)**	**QUALITY CONCERN:**<br>*low/**high**/unclear*
High concern in D1 and D4	
**Overall judgement of risk of bias (evaluation)**	**RISK OF BIAS:**<br>*low/**high**/unclear*
Internal image-level split with leakage, large performance drop across colour temperatures, no external sample	
**Overall judgement of applicability (development)**	**APPLICABILITY CONCERN:**<br>*low/**high**/unclear*
Sample of convenience, no description of patient demographics, one dipstick brand, not validated on real-world patient urine variability.	
**Overall judgement of applicability (evaluation)**	**APPLICABILITY CONCERN:**<br>*low/**high**/unclear*
Same five samples used throughout, does not represent the heterogeneous PoC population.	

Xu et al. (54)

Step 1 - PICOTS

**Population**	195 urine samples collected from Chinese hospital, 187 samples
**Index model(s)**	Random Forest
**Comparator model(s)**	SVM/kNN/XGBoost/LightGBM
**Outcome(s)**	11 analytes: pH (binary), specific gravity (binary), urobilinogen (binary), bilirubin (multi-class), ketones, blood, protein, glucose, leukocytes, nitrite (binary), ascorbic acid (Vit-C).
**Timing**	Cross-sectional.
**Setting and intended use of the prediction model**	Home-monitoring multi-urinalysis with smartphone camera under indoor lighting, intended for non-professional CKD screening and chronic-disease home monitoring.

Step 2 – Classify the type of prediction model assessment

Classify the assessment based on its aim			
Type of prediction study	***Explanation***	***PROBAST+AI boxes to complete***	***Tick as appropriate***
**Development only**	**Prediction model development only, i.e., without evaluation of its performance.**	**Model development**	

**Evaluation only**	**External validation of one or more existing models in new data**	**Model evaluation**	
**Combination**	**Prediction model development combined in the same study (publication) with the evaluation of its apparent performance, internal validation performance, or external validation performance.**	**Model development and evaluation**	**X**

Step 3 - Assess quality and applicability or risk of bias and applicability

**Publication reference**	Xu Q, Yan R, Gui X, Song R, Wang X. Machine learning-assisted image label-free smartphone platform for rapid segmentation and robust multi-urinalysis. Anal Bioanal Chem. 2024;416(6):1443–55.
**Models of interest**	RF
**Outcome of interest**	11 analytes: pH (binary), specific gravity (binary), urobilinogen (binary), bilirubin (multi-class), ketones, blood, protein, glucose, leukocytes, nitrite (binary), ascorbic acid (Vit-C).

Model development

**DOMAIN 1: Participants and data sources**	
**A. Quality**	
195 clinical urine samples collected from Sanya Central Hospital, 187 retained, with 5 sets of 935 images per analyte, 80/20 training/testing	
	**Y/ PY/ PN/ N/ NI**
**1.1 Were appropriate data sources used?**	PY
**1.2 Was an appropriate study design used?**	PY
**1.3 Did the in- and exclusions of study participants result in a representative dataset?**	PY
**Concern regarding quality of selection of participants and data sources**	**QUALITYCONCERN:** ***low**/high/unclear*
Rationale of quality rating: Use of real clinical patient samples, sample is convenience-based and single-centre, image-augmentation by colour-correction algorithms	
**B. Applicability**	
Single-centre sample at Sanya Central Hospital, 195 patients, Dipstick: Dirui H11-800 multi-dipstick, reference instrument: Dirui FUS-2000 Urinalysis Hybrid.	
**Concern that the (data of the) included participants do not match the review question or the assessor's intended use of the prediction model**	**APPLICABILITY CONCERN:** ***low**/high/unclear*
Rationale of applicability rating: Wide age range and real patient samples, one Chinese centre, one dipstick brand, one smartphone model, generalisability to other devices/dipsticks and ethnic groups not demonstrated.	

DOMAIN 2: Predictors	
A. Quality	
Predictors: per-pad HSV features extracted after SAM segmentation of reaction pads, smartphone ISO parameter as additional input, five urine-colour-correction algorithms generate 5 corrected variants per analyte.	
	**Y/ PY/ PN/ N/ NI**
**2.1 Were predictors defined and assessed in a similar way for all participants?**	Y
**2.2 Was any pre-processing of predictors similar for all participants?**	Y
**2.3 Were predictor assessments made without knowledge of outcome data?**	Y
**2.4 Were the predictors included in the model available at the time the model was intended to be used?**	Y
**Concern regarding quality of selection of participants and data sources**	**QUALITYCONCERN:** ***low****/high/unclear*
Standardised feature extraction with on-strip calibration, pre-trained SAM enables label-free segmentation independent of dipstick type, ISO compensates for camera-setting variability.	
**B. Applicability**	
**Concern that the definition, pre-processing, assessment, or timing of assessment of the predictors in the model do not match the review question or the assessor's intended use**	**APPLICABILITY CONCERN:** ***low****/high/unclear*
Rationale of applicability rating: Approach is camera-/dipstick-agnostic in principle, only colour information from a standard smartphone is required.	

DOMAIN 3: Outcome	
A. Quality	
11 chemical components measured per strip. Outcomes treated as binary (NIT, URO, pH, SG) or multi-class (BIL, KET, BLD, PRO, GLU, LEU, Vit-C) by analyser.	
Or	**Y/ PY/ PN/ N/ NI**
**3.1 Were outcomes defined and assessed appropriately?**	Y
**3.2 Were outcomes defined and assessed in a similar way for all participants?**	Y
**3.3 Were outcome assessments made without use or knowledge of predictor data?**	Y
**3.4 Was the time interval between predictor assessment and outcome assessment appropriate?**	Y
**Concern regarding quality of the outcome or its determination**	**QUALITYCONCERN:** ***low****/high/unclear*
Industry-standard automated urinalysis reference, outcome and predictor obtained on the same strip, analyser blinded to images.	
**B. Applicability**	
Outcome determined by automated analyser on the same strip and within 60 s of imaging, composite multi-analyte panel: prevalence varies sharply.	
**Concern that the outcome, its definition, assessment, or timing of assessment do not match the review question or the assessor's intended use**	**APPLICABILITY CONCERN:** ***low****/high/unclear*

Matches routine clinical urinalysis output.

DOMAIN 4: Analysis	
A. Quality	
935 images per analyte from 187 patients × 5 variants, 80/20 training/testing,	
Bayesian optimisation per classifier, macro-average AUROC, One-vs-Rest for multi-class.	
AUROC, accuracy, precision, recall, F1; FPR/FNR with 95% CI; ablations of HSV/ISO/correction.	
Missing data: 8/195 excluded for image quality.	
	**Y/ PY/ PN/ N/ NI**
**4.1 Was there evidence that the sample size was reasonable?**	PY
**4.2 Were continuous and categorical predictors handled appropriately?**	Y
**4.3 Were participants with missing or censored data handled appropriately in the analysis?**	PY
**4.4 If methods to address class imbalance were used, was the model or the model predictions recalibrated?***	PY
**4.5 Were methods used to address potential model overfitting?**	Y
**Concern regarding quality of the analysis**	**QUALITYCONCERN:** ***low***/*high/unclear*
Bayesian tuning, stratified split, CV and bootstrap CI, augmentation-based n-inflation.	

Model evaluation

Domain 1,2 and 3 same analysis as model development

DOMAIN 4: Analysis			
Risk of bias			
20% hold-out per analyte × 5 variants, positive samples scarce for NIT, BIL, URO.			
AUROC, accuracy, precision, recall, F1; FPR/FNR with 95% CI; ablations of HSV/ISO/correction.			
Describe any participants who were excluded from the analysis: Exclusion same as development			
Describe missing data on predictors and outcomes as well as methods used for handling these missing data: Same as development			
	**Y/ PY/ PN/ N/ NI/ NA**		
**4.1 Was model evaluation based on only apparent performance avoided?**	Y		
	**A**	**I**	**E**

**4.2 Was there evidence that the sample size was reasonable?**	NA	PY	NA
**4.3 Were participants with missing or censored data handled appropriately in the analysis?**	NA	PY	NA
**4.4 If methods to address class imbalance were used, was the evaluation done in a dataset without imbalance correction?***	NA	PY	NA
**4.5 If data splitting was done to create training and test datasets, was there evidence that data leakage was avoided?***	**NA**	PY	**NA**
**4.6 If resampling methods were used to evaluate model performance, were all model development steps replicated in the resampling process?***	**NA**	Y	**NA**
**4.7 Was the predictive performance of the model evaluated appropriately, e.g., calibration, discrimination, and net benefit?**	NA	PY	NA
**Risk of bias introduced by the analysis**	**RISK OF BIAS:** *low/**high**/unclear*		
Internal only, augmentation-based splits, leakage risk, high FNR for rare-positive analytes, one dipstick brand and phone.			

Step 4 - Assess the overall concerns regarding quality, risk of bias and applicability of the prediction model

**OVERALL JUDGEMENT OF PREDICTION MODEL**	
**Overall judgement of quality (development)**	**QUALITY CONCERN:** *low/**high**/unclear*
Internal-only evaluation and augmentation-based splits	
**Overall judgement of risk of bias (evaluation)**	**RISK OF BIAS:** *low/**high**/unclear*
Internal-only, augmentation-based splits, no external cohort.	
**Overall judgement of applicability (development)**	**APPLICABILITY CONCERN:** ***low**/high/unclear*
Real clinical urine, single brand/phone.	
**Overall judgement of applicability (evaluation)**	**APPLICABILITY CONCERN:** ***low**/high/unclear*
Same as development.	

Sabanayagam et al. (55)

Step 1 - PICOTS

**Population**	Multi-ethnic adults from SEED (≥40 y, development), SP2 (≥25 y) and BES (≥40 y, external).
**Index model(s)**	Image DLA, RF model, hybrid (image+RF).
**Comparator model(s)**	Image-only vs RF-only vs hybrid; diabetes/HTN subgroups; alternative eGFR<45 threshold.
**Outcome(s)**	CKD = eGFR <60 mL/min/1.73 m^2 (CKD-EPI).
**Timing**	Cross-sectional.

**Setting and intended use of the prediction model**	Community/primary-care opportunistic CKD screening.

Step 2 – Classify the type of prediction model assessment

**Classify the assessment based on its aim**			
Type of prediction study	***Explanation***	***PROBAST+AI boxes to complete***	***Tick as appropriate***
**Development only**	**Prediction model development only, i.e., without evaluation of its performance.**	**Model development**	
**Evaluation only**	**External validation of one or more existing models in new data**	**Model evaluation**	
**Combination**	**Prediction model development combined in the same study (publication) with the evaluation of its apparent performance, internal validation performance, or external validation performance.**	**Model development and evaluation**	**X**

Step 3 - Assess quality and applicability or risk of bias and applicability

**Publication reference**	Sabanayagam C, Xu D, Ting DSW, Nusinovici S, Banu R, Hamzah H, et al. A deep learn-ing algorithm to detect chronic kidney disease from retinal photographs in communi-ty-based populations. Lancet Digit Health. 2020;2(6):e295–e302.
**Models of interest**	Image DLA, RF model, hybrid (image+RF).
**Outcome of interest**	CKD = eGFR <60 mL/min/1.73 m^2 (CKD-EPI).

Model development

**DOMAIN 1: Participants and data sources**	
**A. Quality**	
SEED (2004–17): 6485 patients, 12,970 images, 5188 train / 1297 validation. External: SP2 (n=3735) and BES (n=1538). Exclusions: poor-quality images, missing RFs, overlapping cases.	
	**Y/ PY/ PN/ N/ NI**
**1.1 Were appropriate data sources used?**	Y
**1.2 Was an appropriate study design used?**	Y
**1.3 Did the in- and exclusions of study participants result in a representative dataset?**	PY

**Concern regarding quality of selection of participants and data sources**	**QUALITYCONCERN:** ***low****/high/unclear*
Rationale of quality rating: Large population-based source, clear exclusions, case:control preserved across folds.	
**B. Applicability**	
Asian community cohorts: SEED, SP2, BES, CKD prevalence 3–19%.	
**Concern that the (data of the) included participants do not match the review question or the assessor's intended use of the prediction model**	**APPLICABILITY CONCERN:** ***low****/high/unclear*
Rationale of applicability rating: Population-based Asian cohorts match intended community screening; non-Asian generalisability untested.	

**DOMAIN 2: Predictors**	
**A. Quality**	
Macula-centred 45° non-mydriatic photographs, RFs collected by interview/exam.	
	**Y/ PY/ PN/ N/ NI**
**2.1 Were predictors defined and assessed in a similar way for all participants?**	Y
**2.2 Was any pre-processing of predictors similar for all participants?**	Y
**2.3 Were predictor assessments made without knowledge of outcome data?**	Y
**2.4 Were the predictors included in the model available at the time the model was intended to be used?**	Y
**Concern regarding quality of selection of participants and data sources**	**QUALITYCONCERN:** ***low****/high/unclear*
Standardised acquisition, predictors collected blind to outcome and available at point-of-use.	
**B. Applicability**	
**Concern that the definition, pre-processing, assessment, or timing of assessment of the predictors in the model do not match the review question or the assessor's intended use**	**APPLICABILITY CONCERN:** ***low****/high/unclear*
Rationale of applicability rating: Retinal photography and 5 routine RFs are obtainable in primary care.	

**DOMAIN 3: Outcome**	
**A. Quality**	
CKD = eGFR <60 (CKD-EPI from single-visit serum creatinine). Sensitivity analysis at eGFR <45.	
Or	**Y/ PY/ PN/ N/ NI**
**3.1 Were outcomes defined and assessed appropriately?**	Y
**3.2 Were outcomes defined and assessed in a similar way for all participants?**	Y

**3.3 Were outcome assessments made without use or knowledge of predictor data?**	Y
**3.4 Was the time interval between predictor assessment and outcome assessment appropriate?**	Y
**Concern regarding quality of the outcome or its determination**	**QUALITYCONCERN:** ***low****/high/unclear*
Standard CKD-EPI, same-visit measurement.	
**B. Applicability**	
Outcome determined at the same visit as imaging, composite eGFR-based label dominated by stage-3 CKD, stages 4–5 rare.	
**Concern that the outcome, its definition, assessment, or timing of assessment do not match the review question or the assessor's intended use**	**APPLICABILITY CONCERN:** ***low****/high/unclear*
eGFR <60 is the standard screening threshold.	

**DOMAIN 4: Analysis**	
**A. Quality**	
5188 train (974 CKD) / 1297 val (244). External 3735 / 1538.	
cCondenseNet, 5-fold CV, hybrid concatenates image+RF features.	
AUROC, AUPRC, Sn/Sp/PPV/NPV, DeLong test, heatmaps, no calibration plot or DCA.	
Missing data: Complete-case, poor-quality images excluded.	
	**Y/ PY/ PN/ N/ NI**
**4.1 Was there evidence that the sample size was reasonable?**	Y
**4.2 Were continuous and categorical predictors handled appropriately?**	Y
**4.3 Were participants with missing or censored data handled appropriately in the analysis?**	PY
**4.4 If methods to address class imbalance were used, was the model or the model predictions recalibrated?***	NI
**4.5 Were methods used to address potential model overfitting?**	Y
**Concern regarding quality of the analysis**	**QUALITYCONCERN:** ***low****/high/unclear*
Large sample, 5-fold CV, dropout regularisation; main gap is absence of calibration.	

Model evaluation

Domain 1,2 and 3 same analysis as model development

**DOMAIN 4: Analysis**
**Risk of bias**

SEED val 1297 (244); SP2 3735 (240); BES 1538 (53).			
AUROC, AUPRC, Sn/Sp/PPV/NPV, DeLong test, heatmaps, no calibration plot or DCA.			
Describe any participants who were excluded from the analysis: Exclusion same as development			
Describe missing data on predictors and outcomes as well as methods used for handling these missing data: Same as development			
	**Y/ PY/ PN/ N/ NI/ NA**		
**4.1 Was model evaluation based on only apparent performance avoided?**	Y		
	**A**	**I**	**E**
**4.2 Was there evidence that the sample size was reasonable?**	NA	Y	PY
**4.3 Were participants with missing or censored data handled appropriately in the analysis?**	NA	PY	PY
**4.4 If methods to address class imbalance were used, was the evaluation done in a dataset without imbalance correction?***	NA	NA	NA
**4.5 If data splitting was done to create training and test datasets, was there evidence that data leakage was avoided?***	**NA**	Y	**NA**
**4.6 If resampling methods were used to evaluate model performance, were all model development steps replicated in the resampling process?***	**NA**	Y	**NA**
**4.7 Was the predictive performance of the model evaluated appropriately, e.g., calibration, discrimination, and net benefit?**	NA	PN	PN
**Risk of bias introduced by the analysis**	**RISK OF BIAS:** *low/**high**/unclear*		
Marked AUC drop in SP2, no calibration, poor PPV in low-prevalence settings.			

Step 4 - Assess the overall concerns regarding quality, risk of bias and applicability of the prediction model

**OVERALL JUDGEMENT OF PREDICTION MODEL**	
**Overall judgement of quality (development)**	**QUALITY CONCERN:** ***low**/high/unclear*
All domains low.	
**Overall judgement of risk of bias (evaluation)**	**RISK OF BIAS:** *low/**high**/unclear*
External AUC drop, no calibration, low PPV.	
**Overall judgement of applicability (development)**	**APPLICABILITY CONCERN:** ***low**/high/unclear*
Asian community cohorts match intended use.	

Overall judgement of applicability (evaluation)	APPLICABILITY CONCERN: ***low***/*high*/*unclear*
External cohorts represent screening target.	

Zhang et al. (56)

Step 1 - PICOTS

**Population**	Chinese adults from CC-FII multi-hospital cohorts
**Index model(s)**	ResNet-50 ensemble, RF on metadata (age, sex, height, weight, BMI, BP); combined image+metadata via MLP
**Comparator model(s)**	Metadata-only vs fundus-only vs combined, standard cameras vs smartphone capture.
**Outcome(s)**	CKD (eGFR<60 or albuminuria, KDIGO, ≥2 visits ≥3 mo apart), T2DM (FBG≥7 or HbA1c≥6.5%), continuous eGFR / glucose; incident CKD/T2DM at 5 y.
**Timing**	Cross-sectional for detection; longitudinal for incidence.
**Setting and intended use of the prediction model**	Primary-care/community screening including smartphone-based PoC.

Step 2 – Classify the type of prediction model assessment

**Classify the assessment based on its aim**			
Type of prediction study	***Explanation***	***PROBAST+AI boxes to complete***	***Tick as appropriate***
**Development only**	**Prediction model development only, i.e., without evaluation of its performance.**	**Model development**	
**Evaluation only**	**External validation of one or more existing models in new data**	**Model evaluation**	
**Combination**	**Prediction model development combined in the same study (publication) with the evaluation of its apparent performance, internal validation performance, or external validation performance.**	**Model development and evaluation**	**X**

Step 3 - Assess quality and applicability or risk of bias and applicability

**Publication reference**	Zhang K, Liu X, Xu J, Yuan J, Cai W, Chen T, et al. Deep-learning models for the detection and incidence prediction of chronic kidney disease and type 2 diabetes from retinal fun-dus images. Nature biomedical engineering. 2021;5(6):533–45.
**Models of interest**	ResNet-50 ensemble, RF on metadata, image+metadata via MLP
**Outcome of interest**	CKD (eGFR<60 or albuminuria, KDIGO, ≥2 visits ≥3 mo apart); T2DM (FBG≥7 or HbA1c≥6.5%); continuous eGFR / glucose; incident CKD/T2DM at 5 y.

Model development

**DOMAIN 1: Participants and data sources**	
**A. Quality**	
CC-FII-C: 86,312 images / 43,156 patients split 7:1:2 (train 60,244 / tune 8614 / test 17,454). External 1: 16,118 / 8059. External 2: 6162 / 3081 (Tangshan smartphone). Longitudinal: 8157 dev / 2112 internal / 3376 Beijing external.	
	**Y/ PY/ PN/ N/ NI**
**1.1 Were appropriate data sources used?**	Y
**1.2 Was an appropriate study design used?**	Y
**1.3 Did the in- and exclusions of study participants result in a representative dataset?**	PY
**Concern regarding quality of selection of participants and data sources**	**QUALITYCONCERN:** ***low**/high/unclear*
Rationale of quality rating: Very large multicentre source, multiple independent externals incl. prospective smartphone.	
**B. Applicability**	
Multicentre Chinese patients, age 50–54 y, ~50% male, CKD ~15–18%, T2DM ~29–35%.	
**Concern that the (data of the) included participants do not match the review question or the assessor's intended use of the prediction model**	**APPLICABILITY CONCERN:** ***low**/high/unclear*
Rationale of applicability rating: Matches community-screening use, mainly Chinese ethnicity.	

**DOMAIN 2: Predictors**	
**A. Quality**	
Fundus image + metadata. CLAHE + colour normalization, 4 preprocessing variants in ensemble.	
	**Y/ PY/ PN/ N/ NI**
**2.1 Were predictors defined and assessed in a similar way for all participants?**	Y
**2.2 Was any pre-processing of predictors similar for all participants?**	Y
**2.3 Were predictor assessments made without knowledge of outcome data?**	PY

**2.4 Were the predictors included in the model available at the time the model was intended to be used?**	Y
**Concern regarding quality of selection of participants and data sources**	**QUALITYCONCERN:** ***low**/high/unclear*
Standardised QC pipeline, metadata routinely collected, predictors blind to outcome.	
**B. Applicability**	
**Concern that the definition, pre-processing, assessment, or timing of assessment of the predictors in the model do not match the review question or the assessor's intended use**	**APPLICABILITY CONCERN:** ***low**/high/unclear*
Rationale of applicability rating: Image + basic anthropometrics feasible incl. smartphone PoC.	

**DOMAIN 3: Outcome**	
**A. Quality**	
CKD per KDIGO 2012 (eGFR + albuminuria, confirmed ≥2 visits ≥3 mo apart), eGFR also regressed	
Or	**Y/ PY/ PN/ N/ NI**
**3.1 Were outcomes defined and assessed appropriately?**	Y
**3.2 Were outcomes defined and assessed in a similar way for all participants?**	Y
**3.3 Were outcome assessments made without use or knowledge of predictor data?**	Y
**3.4 Was the time interval between predictor assessment and outcome assessment appropriate?**	Y
**Concern regarding quality of the outcome or its determination**	**QUALITYCONCERN:** ***low**/high/unclear*
Guideline-based, multi-visit confirmation reduces misclassification.	
**B. Applicability**	
Outcomes determined at routine health-check visits with multi-visit confirmation, composite CKD label combines eGFR<60 and albuminuria.	
**Concern that the outcome, its definition, assessment, or timing of assessment do not match the review question or the assessor's intended use**	**APPLICABILITY CONCERN:** ***low**/high/unclear*
KDIGO definitions match intended use.	

**DOMAIN 4: Analysis**
**A. Quality**
60,244 train / 8614 tune / 17,454 test. External 1: 16,118; external 2: 6162. Longitudinal 8157+2112+3376.
ResNet-50 transfer learning, 4-model ensemble, eye-level/patient-level averaging, regression calibration, integrated gradients.

AUROC, Sn/Sp/PPV/NPV at high-Sn/Sp threshold, PCC/R²/MAE, ICC, C-index, time-dependent AUROC, Kaplan–Meier, HR.
Missing data: QC-failed images and unverified follow-up excluded.

	Y/ PY/ PN/ N/ NI
**4.1 Was there evidence that the sample size was reasonable?**	Y
**4.2 Were continuous and categorical predictors handled appropriately?**	Y
**4.3 Were participants with missing or censored data handled appropriately in the analysis?**	Y
**4.4 If methods to address class imbalance were used, was the model or the model predictions recalibrated?***	NI
**4.5 Were methods used to address potential model overfitting?**	Y
**Concern regarding quality of the analysis**	**QUALITYCONCERN:** ***low/**high/unclear*
Large sample, ensembling, separate tuning/test, calibrated regression, longitudinal validation.	

Model evaluation

Domain 1,2 and 3 same analysis as model development

**DOMAIN 4: Analysis**			
**Risk of bias**			
Internal 8727, external 1 8059, external 2 3081, longitudinal: 1685/80 events (internal), 1884/66 (external)			
AUROC, Sn/Sp/PPV/NPV at high-Sn/Sp threshold, PCC/R²/MAE, ICC, C-index, time-dependent AUROC, Kaplan–Meier, HR.			
Describe any participants who were excluded from the analysis: Exclusion same as development			
Describe missing data on predictors and outcomes as well as methods used for handling these missing data: Same as development			
	**Y/ PY/ PN/ N/ NI/ NA**		
**4.1 Was model evaluation based on only apparent performance avoided?**	Y		
	**A**	**I**	**E**
**4.2 Was there evidence that the sample size was reasonable?**	NA	Y	Y
**4.3 Were participants with missing or censored data handled appropriately in the analysis?**	NA	Y	Y
**4.4 If methods to address class imbalance were used, was the evaluation done in a dataset without imbalance correction?***	NA	NA	NA
**4.5 If data splitting was done to create training and test datasets, was there evidence that data leakage was avoided?***	**NA**	Y	**NA**
**4.6 If resampling methods were used to evaluate model performance, were all model development steps replicated in the resampling process?***	**NA**	Y	**NA**
**4.7 Was the predictive performance of the model evaluated appropriately, e.g., calibration, discrimination, and net benefit?**	NA	Y	PY

Risk of bias introduced by the analysis	RISK OF BIAS: ***low***/*high/unclear*
Multiple independent externals incl. smartphone, consistent discrimination, longitudinal validation.	

Step 4 - Assess the overall concerns regarding quality, risk of bias and applicability of the prediction model

OVERALL JUDGEMENT OF PREDICTION MODEL	
**Overall judgement of quality (development)**	**QUALITY CONCERN:** ***low***/*high/unclear*
All domains low.	
**Overall judgement of risk of bias (evaluation)**	**RISK OF BIAS:** ***low***/*high/unclear*
2 cross-sectional + 2 longitudinal externals, calibrated regression.	
**Overall judgement of applicability (development)**	**APPLICABILITY CONCERN:** ***low***/*high/unclear*
Multi-source Chinese cohorts incl. smartphone PoC.	
**Overall judgement of applicability (evaluation)**	**APPLICABILITY CONCERN:** ***low***/*high/unclear*
Matches community screening use.	

Dong et al. (57)

Step 1 - PICOTS

**Population**	T2DM-CKD adults undergoing renal biopsy at 5 Chinese hospitals.
**Index model(s)**	Trans-MUF: ImgLesion subnet, factor subnet, diagnosis subnet
**Comparator model(s)**	DenseNet-121, AlexNet, VGG-16, DAFT, reader study by 3 nephrologists with vs without AI visualisation.
**Outcome(s)**	Biopsy-confirmed DN vs NDRD by 3 blinded nephropathologists
**Timing**	Cross-sectional for detection; longitudinal for incidence.
**Setting and intended use of the prediction model**	Tertiary nephrology, intended to avoid unnecessary biopsy in T2DM-CKD.

Step 2 – Classify the type of prediction model assessment

Classify the assessment based on its aim

Type of prediction study	***Explanation***	***PROBAST+AI boxes to complete***	***Tick as appropriate***
**Development only**	**Prediction model development only, i.e., without evaluation of its performance.**	**Model development**	
**Evaluation only**	**External validation of one or more existing models in new data**	**Model evaluation**	
**Combination**	**Prediction model development combined in the same study (publication) with the evaluation of its apparent performance, internal validation performance, or external validation performance.**	**Model development and evaluation**	**X**

Step 3 - Assess quality and applicability or risk of bias and applicability

**Publication reference**	Dong ZY, Wang XF, Pan S, Weng TH, Chen XN, Jiang SS, et al. A multimodal transformer system for noninvasive diabetic nephropathy diagnosis via retinal imaging. Npj Digital Medicine. 2025;8(1).
**Models of interest**	Trans-MUF: ImgLesion subnet, factor subnet, diagnosis subnet
**Outcome of interest**	Biopsy-confirmed DN vs NDRD by 3 blinded nephropathologists

Model development

**DOMAIN 1: Participants and data sources**	
**A. Quality**	
Retina-DKD: 934 images / 246 patients at PLA General Hospital (DN/NDRD 96/106), patient-wise 8/2: train 202/ test 44, prospective: 41/136, multicentre external: 28/55, non-standard-angle: 16/42.	
	**Y/ PY/ PN/ N/ NI**
**1.1 Were appropriate data sources used?**	PY
**1.2 Was an appropriate study design used?**	Y
**1.3 Did the in- and exclusions of study participants result in a representative dataset?**	PY
**Concern regarding quality of selection of participants and data sources**	**QUALITYCONCERN:** ***low****/high/unclear*
Rationale of quality rating: Biopsy-confirmed labels, multicentre design, small external limits power.	
**B. Applicability**	
5 Chinese tertiary hospitals, mean age ~52 y, mean eGFR ~60, mean HbA1c 7.5%, 50–80% male.	

Concern that the (data of the) included participants do not match the review question or the assessor's intended use of the prediction model	APPLICABILITY CONCERN: ***low**/high/unclear*
Rationale of applicability rating: Biopsy-confirmed T2DM-CKD matches intended use, only Chinese, small external.	

DOMAIN 2: Predictors	
A. Quality	
Macula-centred fundus, U-Net lesion-segmentation, 5 clinical risk factors selected by univariate logistic regression (P<0.05).	
	**Y/ PY/ PN/ N/ NI**
**2.1 Were predictors defined and assessed in a similar way for all participants?**	Y
**2.2 Was any pre-processing of predictors similar for all participants?**	Y
**2.3 Were predictor assessments made without knowledge of outcome data?**	Y
**2.4 Were the predictors included in the model available at the time the model was intended to be used?**	Y
**Concern regarding quality of selection of participants and data sources**	**QUALITYCONCERN:** ***low**/high/unclear*
Standardised pipeline, clinical factors routinely measured.	
**B. Applicability**	
**Concern that the definition, pre-processing, assessment, or timing of assessment of the predictors in the model do not match the review question or the assessor's intended use**	**APPLICABILITY CONCERN:** ***low**/high/unclear*
Rationale of applicability rating: Obtainable in any tertiary nephrology / endocrine clinic.	

DOMAIN 3: Outcome	
A. Quality	
Renal biopsy with LM + IF + EM, consensus diagnosis by 3 blinded pathologists, RPS criteria.	
Or	**Y/ PY/ PN/ N/ NI**
**3.1 Were outcomes defined and assessed appropriately?**	Y
**3.2 Were outcomes defined and assessed in a similar way for all participants?**	Y
**3.3 Were outcome assessments made without use or knowledge of predictor data?**	Y
**3.4 Was the time interval between predictor assessment and outcome assessment appropriate?**	Y
**Concern regarding quality of the outcome or its determination**	**QUALITYCONCERN:** ***low**/high/unclear*
Histological gold standard, blinded, uniform criteria.	

B. Applicability	
Outcome determined at biopsy obtained around the time of fundus imaging, binary DN vs NDRD, NDRD is heterogeneous but reported as a single combined non-DN class.	
**Concern that the outcome, its definition, assessment, or timing of assessment do not match the review question or the assessor's intended use**	**APPLICABILITY CONCERN:** ***low***/*high/unclear*
Biopsy DN vs NDRD is the clinical reference for the intended biopsy-avoidance use.	

DOMAIN 4: Analysis	
A. Quality	
Train 841/202, internal test 93/44, prospective 136/41, multicentre external 55/28, non-standard-angle 42/16, 5 clinical predictors, 17-class lesion annotation in 529 images.	
U-Net segmentation, WAM-ResNet, Transformer + SE attention, weighted cross-entropy, integrated gradients / Grad-CAM.	
Accuracy, Sn, F1, AUC with 100-resample bootstrap Cis, image- and patient-level evaluation, reader study.	
Missing data: QC-failed images and missing biochemistry excluded.	
	**Y/ PY/ PN/ N/ NI**
**4.1 Was there evidence that the sample size was reasonable?**	PY
**4.2 Were continuous and categorical predictors handled appropriately?**	Y
**4.3 Were participants with missing or censored data handled appropriately in the analysis?**	Y
**4.4 If methods to address class imbalance were used, was the model or the model predictions recalibrated?***	Y
**4.5 Were methods used to address potential model overfitting?**	Y
**Concern regarding quality of the analysis**	**QUALITYCONCERN:** ***low***/*high/unclear*
Class-weighted loss, modality ablations, reader study, modest 202-patient training for a transformer.	

Model evaluation

Domain 1,2 and 3 same analysis as model development

DOMAIN 4: Analysis
**Risk of bias**
Internal 93/44, prospective 136/41, external 55/28, non-standard 42/16.
Accuracy, Sn, F1, AUC with 100-resample bootstrap Cis, image- and patient-level evaluation, reader study.
Describe any participants who were excluded from the analysis:<br>Exclusion same as development

Describe missing data on predictors and outcomes as well as methods used for handling these missing data:<br>Same as development			
	**Y/ PY/ PN/ N/ NI/ NA**		
**4.1 Was model evaluation based on only apparent performance avoided?**	Y		
	**A**	**I**	**E**
**4.2 Was there evidence that the sample size was reasonable?**	NA	PY	PN
**4.3 Were participants with missing or censored data handled appropriately in the analysis?**	NA	Y	Y
**4.4 If methods to address class imbalance were used, was the evaluation done in<br>a dataset without imbalance correction?***	NA	PY	PY
**4.5 If data splitting was done to create training and test datasets, was there evidence that data leakage was avoided?***	**NA**	Y	**NA**
**4.6 If resampling methods were used to evaluate model performance, were all<br>model development steps replicated in the resampling process?***	**NA**	Y	**NA**
**4.7 Was the predictive performance of the model evaluated appropriately, e.g., calibration, discrimination, and net benefit?**	NA	PY	PY
**Risk of bias introduced by the analysis**	**RISK OF BIAS:**<br>*low/**high**/unclear*		
Excellent discrimination across cohorts and convincing reader-study uplift, external cohort only 28 patients, only Chinese centres, no calibration plot / DCA, AUC drops at non-standard angles.			

Step 4 - Assess the overall concerns regarding quality, risk of bias and applicability of the prediction model

**OVERALL JUDGEMENT OF PREDICTION MODEL**	
**Overall judgement of quality (development)**	**QUALITY CONCERN:**<br>***low**/high/unclear*
All domains low.	
**Overall judgement of risk of bias (evaluation)**	**RISK OF BIAS:**<br>*low/**high**/unclear*
Strong AUC but small external sample, no calibration, angle sensitivity.	
**Overall judgement of applicability (development)**	**APPLICABILITY CONCERN:**<br>***low**/high/unclear*
Multicentre Chinese T2DM-CKD biopsy population, small external testing, Chinese only.	
**Overall judgement of applicability (evaluation)**	**APPLICABILITY CONCERN:**<br>***low**/high/unclear*
Chinese tertiary nephrology, non-standard angles fragile.	

Bhak et al. (58)

Step 1 - PICOTS

**Population**	Korean adults 20–79 y at two health-promotion centres.
**Index model(s)**	WRN-28-4 multimodal: 256-D image features with 12-D vector into FC layers with sigmoid.
**Comparator model(s)**	eGFR-RIDL (image-only WRN), eGFR-UDLR (urine LR), subgroup analyses by age <65/≥65, diabetes, hypertension, proteinuria.
**Outcome(s)**	eGFR <60 mL/min/1.73 m² by CKD-EPI 2009.
**Timing**	Cross-sectional
**Setting and intended use of the prediction model**	Health-promotion/annual check-up, intended for non-invasive CKD screening.

Step 2 – Classify the type of prediction model assessment

**Classify the assessment based on its aim**			
Type of prediction study	***Explanation***	***PROBAST+AI boxes to complete***	***Tick as appropriate***
**Development only**	**Prediction model development only, i.e., without evaluation of its performance.**	**Model development**	
**Evaluation only**	**External validation of one or more existing models in new data**	**Model evaluation**	
**Combination**	**Prediction model development combined in the same study (publication) with the evaluation of its apparent performance, internal validation performance, or external validation performance.**	**Model development and evaluation**	**X**

Step 3 - Assess quality and applicability or risk of bias and applicability

**Publication reference**	Bhak Y, Lee YH, Kim J, Lee K, Lee D, Jang EC, et al. Diagnosis of Chronic Kidney Disease Using Retinal Imaging and Urine Dipstick Data: Multimodal Deep Learning Approach. JMIR Med Inform. 2025;13:e55825.
**Models of interest**	WRN-28-4 multimodal: 256-D image features with 12-D vector into FC layers with sigmoid.
**Outcome of interest**	eGFR <60 mL/min/1.73 m^2 by CKD-EPI 2009.

Model development

DOMAIN 1: Participants and data sources	
**A. Quality**	
65,082 patients / 128,938 images; train 72,204 / val 18,066 / test 38,668. External set: 58,284 patients/ 113,279 images.	
	**Y/ PY/ PN/ N/ NI**
**1.1 Were appropriate data sources used?**	Y
**1.2 Was an appropriate study design used?**	Y
**1.3 Did the in- and exclusions of study participants result in a representative dataset?**	PY
**Concern regarding quality of selection of participants and data sources**	**QUALITYCONCERN:** ***low***/*high/unclear*
Rationale of quality rating: Two large health-check cohorts; clear QC; overlap window 35 d.	
**B. Applicability**	
Two Korean health-promotion centres, mean age 46 y, 54% male, diabetes 14.2%, HTN 18.6%, CKD 1.7%. Severance Checkup: mean age 48 y, 52% male, diabetes 5%, HTN 14.1%, CKD 0.8%.	
**Concern that the (data of the) included participants do not match the review question or the assessor's intended use of the prediction model**	**APPLICABILITY CONCERN:** ***low***/*high/unclear*
Rationale of applicability rating: Health-check populations match intended use, different cameras strengthen transportability, only Koreans tested.	

DOMAIN 2: Predictors	
**A. Quality**	
Macula-centred fundus + age + sex + 10 dipstick variables (, numerical variables standardized, 256-D image features concatenated with 12-D numerical vector.	
	**Y/ PY/ PN/ N/ NI**
**2.1 Were predictors defined and assessed in a similar way for all participants?**	Y
**2.2 Was any pre-processing of predictors similar for all participants?**	Y
**2.3 Were predictor assessments made without knowledge of outcome data?**	Y
**2.4 Were the predictors included in the model available at the time the model was intended to be used?**	Y
**Concern regarding quality of selection of participants and data sources**	**QUALITYCONCERN:** ***low***/*high/unclear*
Standardised acquisition, dipstick is routine PoC assay.	
**B. Applicability**	
**Concern that the definition, pre-processing, assessment, or timing of assessment of the predictors in the model do not match the review question or the assessor's intended use**	**APPLICABILITY CONCERN:** ***low***/*high/unclear*

Rationale of applicability rating: All inputs obtainable at any health check.

**DOMAIN 3: Outcome**	
**A. Quality**	
CKD = eGFR <60 by CKD-EPI 2009 from single-visit creatinine, 95% CIs by 1000 bootstrap.	
Or	**Y/ PY/ PN/ N/ NI**
**3.1 Were outcomes defined and assessed appropriately?**	Y
**3.2 Were outcomes defined and assessed in a similar way for all participants?**	Y
**3.3 Were outcome assessments made without use or knowledge of predictor data?**	Y
**3.4 Was the time interval between predictor assessment and outcome assessment appropriate?**	Y
**Concern regarding quality of the outcome or its determination**	**QUALITYCONCERN:** ***low**/high/unclear*
Standard CKD-EPI, single-visit may misclassify borderline cases.	
**B. Applicability**	
Outcome determined at the health-check visit, single-visit eGFR threshold without KDIGO multi-visit confirmation, very low CKD prevalence in external set (0.8%).	
**Concern that the outcome, its definition, assessment, or timing of assessment do not match the review question or the assessor's intended use**	**APPLICABILITY CONCERN:** ***low**/high/unclear*
Binary eGFR <60 matches screening threshold.	

**DOMAIN 4: Analysis**	
**A. Quality**	
Training 72,204 / validation 18,066 / testing 38,668 images, CKD prevalence ~1.7% dev / 0.8% external, severe imbalance.	
WRN-28-4, BCE scaled by class ratio, early stopping, per-eye averaging, saliency maps, DeLong test with Bonferroni.	
AUROC, Sn/Sp at Index-of-Union threshold, saliency, sensitivity analysis on numerical features.	
Missing data: Patients without urinalysis or with low-quality images or outside 20–79 y excluded.	
	**Y/ PY/ PN/ N/ NI**
**4.1 Was there evidence that the sample size was reasonable?**	Y
**4.2 Were continuous and categorical predictors handled appropriately?**	Y
**4.3 Were participants with missing or censored data handled appropriately in the analysis?**	Y

**4.4 If methods to address class imbalance were used, was the model or the model predictions recalibrated?***	Y
**4.5 Were methods used to address potential model overfitting?**	Y
**Concern regarding quality of the analysis**	**QUALITYCONCERN:** ***low/****high/unclear*
Large sample, weighted loss for imbalance, early stopping, no calibration plot.	

Model evaluation

Domain 1,2 and 3 same analysis as model development

**DOMAIN 4: Analysis**			
**Risk of bias**			
Internal 38,668/19,529 (1.7% CKD); external 113,279/58,284 (0.8% CKD).			
AUROC, Sn/Sp at Index-of-Union threshold, saliency, sensitivity analysis on numerical features.			
Describe any participants who were excluded from the analysis: Exclusion same as development			
Describe missing data on predictors and outcomes as well as methods used for handling these missing data: Same as development			
	**Y/ PY/ PN/ N/ NI/ NA**		
**4.1 Was model evaluation based on only apparent performance avoided?**	Y		
	**A**	**I**	**E**
**4.2 Was there evidence that the sample size was reasonable?**	NA	Y	Y
**4.3 Were participants with missing or censored data handled appropriately in the analysis?**	NA	Y	Y
**4.4 If methods to address class imbalance were used, was the evaluation done in a dataset without imbalance correction?***	NA	PY	PY
**4.5 If data splitting was done to create training and test datasets, was there evidence that data leakage was avoided?***	**NA**	Y	**NA**
**4.6 If resampling methods were used to evaluate model performance, were all model development steps replicated in the resampling process?***	**NA**	Y	**NA**
**4.7 Was the predictive performance of the model evaluated appropriately, e.g., calibration, discrimination, and net benefit?**	NA	PY	PY
**Risk of bias introduced by the analysis**	**RISK OF BIAS:** ***low****/high/unclear*		
Independent external validation with different cameras, consistent discrimination, transparent subgroups, calibration plot not reported.			

Step 4 - Assess the overall concerns regarding quality, risk of bias and applicability of the prediction model

OVERALL JUDGEMENT OF PREDICTION MODEL	
**Overall judgement of quality (development)**	**QUALITY CONCERN:** ***low***/*high*/*unclear*
All domains low.	
**Overall judgement of risk of bias (evaluation)**	**RISK OF BIAS:** ***low***/*high*/*unclear*
External validation with different cameras, calibration not reported.	
**Overall judgement of applicability (development)**	**APPLICABILITY CONCERN:** ***low***/*high*/*unclear*
Korean only, ≥65 y subgroup underperforms.	
**Overall judgement of applicability (evaluation)**	**APPLICABILITY CONCERN:** ***low***/*high*/*unclear*
Two Korean centres.	

An et al. (59)

Step 1 - PICOTS

**Population**	UK Biobank adults 20–79 y, mostly European, fundus images and biomarkers.
**Index model(s)**	Two ensembles (one per eGFR definition) of 5 models: ResNet-50, EfficientNetV2S, ResNet-50+metadata, Gaussian-blur, lightness-CLAHE.
**Comparator model(s)**	Single- vs two-factor eGFR labels, cross-evaluation on ICD10 N18 labels.
**Outcome(s)**	CKD = eGFR <60 vs ≥90 (CKD-EPI 2021), ICD10 N18 ever reported.
**Timing**	Cross-sectional
**Setting and intended use of the prediction model**	Population-based research cohort, intended for community CKD screening.

Step 2 – Classify the type of prediction model assessment

Classify the assessment based on its aim			
Type of prediction study	***Explanation***	***PROBAST+AI boxes to complete***	***Tick as appropriate***
**Development only**	**Prediction model development only, i.e., without evaluation of its performance.**	**Model development**	
**Evaluation only**	**External validation of one or more existing models in new data**	**Model evaluation**	

**Combination**	**Prediction model development combined in the same study (publication) with the evaluation of its apparent performance, internal validation performance, or external validation performance.**	**Model development and evaluation**	**X**

Step 3 - Assess quality and applicability or risk of bias and applicability

**Publication reference**	An S, Vaghefi E, Yang S, Xie L, Squirrell D. Examination of alternative eGFR definitions on the performance of deep learning models for detection of chronic kidney disease from fundus photographs. PLoS One. 2023;18(11):e0295073.
**Models of interest**	Two ensembles (one per eGFR definition) of 5 models: ResNet-50, EfficientNetV2S, ResNet-50+metadata, Gaussian-blur, lightness-CLAHE.
**Outcome of interest**	CKD = eGFR <60 vs ≥90 (CKD-EPI 2021), ICD10 N18 ever reported.

Model development

**DOMAIN 1: Participants and data sources**	
**A. Quality**	
175,788 images / 85,707 patients screened; down-sampled to 1/8 case/control yielding 14,040 / 12,636. 80/20 dev/test split, eGFR 60–89 excluded from training.	
	**Y/ PY/ PN/ N/ NI**
**1.1 Were appropriate data sources used?**	Y
**1.2 Was an appropriate study design used?**	Y
**1.3 Did the in- and exclusions of study participants result in a representative dataset?**	PN
**Concern regarding quality of selection of participants and data sources**	**QUALITYCONCERN:** *low/**high**/unclear*
Rationale of quality rating: Aggressive down-sampling and exclusion of borderline eGFR distort population, no external set.	
**B. Applicability**	
UK Biobank European adults (mean age 57 y; 46% male; BMI 27; diabetes 5.5%; eGFR <60 in 6.7%); Topcon 3D OCT 1000 Mk2 non-mydriatic 45° fundus camera across UK assessment centres.	
**Concern that the (data of the) included participants do not match the review question or the assessor's intended use of the prediction model**	**APPLICABILITY CONCERN:** *low/**high**/unclear*
Rationale of applicability rating: Low systemic-disease prevalence and single camera differ from intended primary-care DR-screening context, no external generalisability test.	

**DOMAIN 2: Predictors**

A. Quality	
Macula-centred 45° non-mydriatic images, optional Gaussian-blur or CLAHE enhancement; age, sex, ethnicity for metadata model.	
	**Y/ PY/ PN/ N/ NI**
**2.1 Were predictors defined and assessed in a similar way for all participants?**	Y
**2.2 Was any pre-processing of predictors similar for all participants?**	Y
**2.3 Were predictor assessments made without knowledge of outcome data?**	Y
**2.4 Were the predictors included in the model available at the time the model was intended to be used?**	Y
**Concern regarding quality of selection of participants and data sources**	**QUALITYCONCERN:** ***low***/*high*/*unclear*
Uniform UK Biobank pipeline.	
**B. Applicability**	
**Concern that the definition, pre-processing, assessment, or timing of assessment of the predictors in the model do not match the review question or the assessor's intended use**	**APPLICABILITY CONCERN:** ***low***/*high*/*unclear*
Rationale of applicability rating: Topcon 3D OCT device less common in primary-care DR screening.	

DOMAIN 3: Outcome	
**A. Quality**	
eGFR <60 by CKD-EPI 2021 (single-factor or two-factor), ICD10 N18 alternative outcome.	
Or	**Y/ PY/ PN/ N/ NI**
**3.1 Were outcomes defined and assessed appropriately?**	PY
**3.2 Were outcomes defined and assessed in a similar way for all participants?**	Y
**3.3 Were outcome assessments made without use or knowledge of predictor data?**	Y
**3.4 Was the time interval between predictor assessment and outcome assessment appropriate?**	PY
**Concern regarding quality of the outcome or its determination**	**QUALITYCONCERN:** ***low***/*high*/*unclear*
Standard CKD-EPI, single-visit biomarkers may misclassify borderline cases.	
**B. Applicability**	
Outcome determined at UK Biobank baseline visit and via linked-records ICD10 N18, composite single-visit eGFR threshold without multi-visit KDIGO confirmation.	
**Concern that the outcome, its definition, assessment, or timing of assessment do not match the review question or the assessor's intended use**	**APPLICABILITY CONCERN:** ***low***/*high*/*unclear*
eGFR threshold is clinical standard, ICD10 captures late/symptomatic disease.	

DOMAIN 4: Analysis	
A. Quality	
14,040 images / 12,636 patients, dev 11,232 / test 2808, cases at eGFR<60: 1249 dev / 311 test.	
ResNet-50 / EfficientNetV2S backbones + dropout (0.3) + 4-FC head + sigmoid, eGFR 60–90 excluded from training, Youden threshold, pairwise t-tests	
AUROC, Sn, Sp, F1 against eGFR labels and ICD10.	
Missing data: Patients with missing biomarkers excluded.	
	**Y/ PY/ PN/ N/ NI**
**4.1 Was there evidence that the sample size was reasonable?**	PY
**4.2 Were continuous and categorical predictors handled appropriately?**	Y
**4.3 Were participants with missing or censored data handled appropriately in the analysis?**	PY
**4.4 If methods to address class imbalance were used, was the model or the model predictions recalibrated?***	PY
**4.5 Were methods used to address potential model overfitting?**	Y
**Concern regarding quality of the analysis**	**QUALITYCONCERN:** ***low****/high/unclear*
Standard transfer learning, regularisation, hold-out, Youden threshold, sample adequate.	

Model evaluation

Domain 1,2 and 3 same analysis as model development

DOMAIN 4: Analysis			
Risk of bias			
Test 2808 images / 2750 patients, ICD10 N18 prevalence lower.			
AUROC, Sn, Sp, F1 against eGFR labels and ICD10.			
Describe any participants who were excluded from the analysis: Exclusion same as development			
Describe missing data on predictors and outcomes as well as methods used for handling these missing data: Same as development			
	**Y/ PY/ PN/ N/ NI/ NA**		
**4.1 Was model evaluation based on only apparent performance avoided?**	Y		
	**A**	**I**	**E**
**4.2 Was there evidence that the sample size was reasonable?**	NA	PY	NA
**4.3 Were participants with missing or censored data handled appropriately in the analysis?**	NA	PY	NA

**4.4 If methods to address class imbalance were used, was the evaluation done in a dataset without imbalance correction?***	NA	NA	NA
**4.5 If data splitting was done to create training and test datasets, was there evidence that data leakage was avoided?***	**NA**	Y	**NA**
**4.6 If resampling methods were used to evaluate model performance, were all model development steps replicated in the resampling process?***	**NA**	PY	**NA**
**4.7 Was the predictive performance of the model evaluated appropriately, e.g., calibration, discrimination, and net benefit?**	NA	PN	NA
**Risk of bias introduced by the analysis**	**RISK OF BIAS:** *low/**high**/unclear*		
Internal only, modest AUROC, two-factor gain not robust to ICD10, no calibration.			

Step 4 - Assess the overall concerns regarding quality, risk of bias and applicability of the prediction model

**OVERALL JUDGEMENT OF PREDICTION MODEL**	
**Overall judgement of quality (development)**	**QUALITY CONCERN:** *low/**high**/unclear*
D1 high	
**Overall judgement of risk of bias (evaluation)**	**RISK OF BIAS:** ***low**/high/unclear*
Internal hold-out only, modest AUROC, not robust to clinical labels.	
**Overall judgement of applicability (development)**	**APPLICABILITY CONCERN:** *low/**high**/unclear*
UK Biobank European cohort, low disease prevalence, single device.	
**Overall judgement of applicability (evaluation)**	**APPLICABILITY CONCERN:** ***low**/high/unclear*
No transportability shown.	

Xiao et al. (60)

Step 1 - PICOTS

**Population**	551 pathologically-confirmed CKD outpatients with 24-h urine protein, Chinese hospital
**Index model(s)**	logistic regression (LR), Elastic Net, Lasso, Ridge, SVM (RBF), random forest (RF), XGBoost, neural network (NN), k-nearest neighbour (NN).
**Comparator model(s)**	Models compared pairwise, clustered hierarchically by FP/FN profiles.
**Outcome(s)**	Binary classification of 24-h urinary protein: mild (≤1 g/24 h) vs moderate/severe (>1 g/24 h).
**Timing**	Cross-sectional

**Setting and intended use of the prediction model**	Outpatient nephrology follow-up, intended as a quick screening tool to avoid 24-h urine collection.

Step 2 – Classify the type of prediction model assessment

Classify the assessment based on its aim			
Type of prediction study	***Explanation***	***PROBAST+AI boxes to complete***	***Tick as appropriate***
**Development only**	**Prediction model development only, i.e., without evaluation of its performance.**	**Model development**	
**Evaluation only**	**External validation of one or more existing models in new data**	**Model evaluation**	
**Combination**	**Prediction model development combined in the same study (publication) with the evaluation of its apparent performance, internal validation performance, or external validation performance.**	**Model development and evaluation**	**X**

Step 3 - Assess quality and applicability or risk of bias and applicability

**Publication reference**	Xiao J, Ding R, Xu X, Guan H, Feng X, Sun T, et al. Comparison and development of ma-chine learning tools in the prediction of chronic kidney disease progression. J Transl Med. 2019;17(1):119.
**Models of interest**	LR, Elastic Net, Lasso, Ridge, SVM, RF, XGBoost, NN, KNN using 13 blood biochemistry + 5 demographic predictors.
**Outcome of interest**	Urine protein >1 g/24 h (moderate/severe) vs ≤1 g/24 h (mild).

Model development

DOMAIN 1: Participants and data sources	
**A. Quality**	
551 CKD outpatients (330 mild, 221 moderate/severe), single tertiary nephrology centre, power analysis indicated adequate sample.	
	**Y/ PY/ PN/ N/ NI**
**1.1 Were appropriate data sources used?**	Y
**1.2 Was an appropriate study design used?**	PY
**1.3 Did the in- and exclusions of study participants result in a representative**	PN

**dataset?**	
**Concern regarding quality of selection of participants and data sources**	**QUALITYCONCERN:** *low/**high**/unclear*
Rationale of quality rating: Single-centre tertiary cohort with restrictive exclusions limits representativeness, sample size modest for nine-model comparison.	
**B. Applicability**	
Chinese tertiary-hospital nephrology outpatients, mean age ~58 y, 51% male, CKD severity confirmed pathologically, comorbidities excluded, atypical of routine CKD population.	
**Concern that the (data of the) included participants do not match the review question or the assessor's intended use of the prediction model**	**APPLICABILITY CONCERN:** *low/**high**/unclear*
Rationale of applicability rating: Cohort excludes common CKD comorbidities (MetS, CVD), limits applicability to general outpatient CKD follow-up despite tool's intended broad use.	

**DOMAIN 2: Predictors**	
**A. Quality**	
13 routine blood biochemistry tests 5 demographics, standardised by Z-score, categorical variables converted to dummies	
	**Y/ PY/ PN/ N/ NI**
**2.1 Were predictors defined and assessed in a similar way for all participants?**	Y
**2.2 Was any pre-processing of predictors similar for all participants?**	Y
**2.3 Were predictor assessments made without knowledge of outcome data?**	PY
**2.4 Were the predictors included in the model available at the time the model was intended to be used?**	Y
**Concern regarding quality of selection of participants and data sources**	**QUALITYCONCERN:** ***low**/high/unclear*
Standard, routinely-available blood and demographic variables uniformly assessed and preprocessed.	
**B. Applicability**	
**Concern that the definition, pre-processing, assessment, or timing of assessment of the predictors in the model do not match the review question or the assessor's intended use**	**APPLICABILITY CONCERN:** ***low**/high/unclear*
Rationale of applicability rating: Standard, routinely-available blood and demographic variables uniformly assessed and preprocessed.	

**DOMAIN 3: Outcome**	
**A. Quality**	
24-h urinary protein dichotomised at 1 g/24 h. Gold-standard 24-h urine collection, same-visit ascertainment of predictors and outcome.	
Or	**Y/ PY/ PN/ N/ NI**
**3.1 Were outcomes defined and assessed appropriately?**	Y

**3.2 Were outcomes defined and assessed in a similar way for all participants?**	Y
**3.3 Were outcome assessments made without use or knowledge of predictor data?**	PY
**3.4 Was the time interval between predictor assessment and outcome assessment appropriate?**	Y
**Concern regarding quality of the outcome or its determination**	**QUALITYCONCERN:** ***low***/*high*/*unclear*
Gold-standard outcome measured uniformly at the same visit.	
**B. Applicability**	
Outcome determined at the same outpatient follow-up visit as predictors, single binary outcome, class distribution 330 mild (60%) / 221 moderate-severe (40%).	
**Concern that the outcome, its definition, assessment, or timing of assessment do not match the review question or the assessor's intended use**	**APPLICABILITY CONCERN:** ***low***/*high*/*unclear*
1 g/24 h threshold is clinically relevant for CKD severity stratification.	

**DOMAIN 4: Analysis**	
**A. Quality**	
551 patients (440 train / 111 validation), 18 candidate predictors, 221 events	
Nine algorithms tuned by grid search with 10-fold CV, 20-iteration resampling, feature importance via model coefficients, native importance, and mean-decrease accuracy (for KNN/SVM/NN).	
discrimination only (AUC, PR-AUC, F1), no calibration plot or decision-curve analysis.	
Missing data: Z-score scaling, minor missingness, handling of missing values not explicitly described.	
	**Y/ PY/ PN/ N/ NI**
**4.1 Was there evidence that the sample size was reasonable?**	PN
**4.2 Were continuous and categorical predictors handled appropriately?**	Y
**4.3 Were participants with missing or censored data handled appropriately in the analysis?**	NI
**4.4 If methods to address class imbalance were used, was the model or the model predictions recalibrated?***	NI
**4.5 Were methods used to address potential model overfitting?**	PY
**Concern regarding quality of the analysis**	**QUALITYCONCERN:** *low*/***high***/*unclear*
Modest sample for nine-model comparison, missing-data handling unclear, no calibration or net-benefit assessment.	

Model evaluation

Domain 1,2 and 3 same analysis as model development

**DOMAIN 4: Analysis**

Risk of bias			
111 validation cases per iteration			
discrimination only (AUC, PR-AUC, F1), no calibration plot or decision-curve analysis.			
Describe any participants who were excluded from the analysis:<br>Exclusion same as development			
Describe missing data on predictors and outcomes as well as methods used for handling these missing data:<br>Same as development			
	**Y/ PY/ PN/ N/ NI/ NA**		
**4.1 Was model evaluation based on only apparent performance avoided?**	Y		
	**A**	**I**	**E**
**4.2 Was there evidence that the sample size was reasonable?**	NA	PN	NA
**4.3 Were participants with missing or censored data handled appropriately in the analysis?**	NA	NI	NA
**4.4 If methods to address class imbalance were used, was the evaluation done in a dataset without imbalance correction?***	NA	NA	NA
**4.5 If data splitting was done to create training and test datasets, was there evidence that data leakage was avoided?***	**NA**	PY	**NA**
**4.6 If resampling methods were used to evaluate model performance, were all model development steps replicated in the resampling process?***	**NA**	PY	**NA**
**4.7 Was the predictive performance of the model evaluated appropriately, e.g., calibration, discrimination, and net benefit?**	NA	PN	NA
**Risk of bias introduced by the analysis**	**RISK OF BIAS:**<br>*low/**high**/unclear*		
No external validation, no calibration or decision-curve analysis, small validation slices.			

Step 4 - Assess the overall concerns regarding quality, risk of bias and applicability of the prediction model

OVERALL JUDGEMENT OF PREDICTION MODEL	
**Overall judgement of quality (development)**	**QUALITY CONCERN:**<br>*low/**high**/unclear*
Single-centre, restrictive exclusions, modest sample for nine-model comparison, no calibration.	
**Overall judgement of risk of bias (evaluation)**	**RISK OF BIAS:**<br>*low/**high**/unclear*
Internal validation only, discrimination-only metrics, small held-out slices.	
**Overall judgement of applicability (development)**	**APPLICABILITY CONCERN:**<br>*low/**high**/unclear*

Restrictive exclusions (MetS, CVD, cancer) limit applicability to typical CKD outpatients.

**Overall judgement of applicability (evaluation)**	**APPLICABILITY CONCERN:** ***low***/*high*/*unclear*
Single Chinese tertiary cohort, external generalisability unproven.	

Lu et al. (61)

Step 1 - PICOTS

**Population**	1358 pathologically-confirmed CKD patients treated at Chinese hospital
**Index model(s)**	Soft-voting ensemble combining the two best single learners (logistic regression + XGBoost) after RFE-LR feature selection, SHAP for interpretation.
**Comparator model(s)**	XGBoost, Gaussian Naïve Bayes (GNB), neural network (NN), ridge regression, logistic regression and the single-marker baselines eGFR and serum creatinine.
**Outcome(s)**	Binary classification of 24-h urinary protein: mild (≤1 g/24 h) vs advanced (>1 g/24 h).
**Timing**	Cross-sectional
**Setting and intended use of the prediction model**	Tertiary nephrology inpatient/outpatient setting, intended to replace 24-h urine collection with routine blood biochemistry for outpatient follow-up risk stratification.

Step 2 – Classify the type of prediction model assessment

**Classify the assessment based on its aim**			
Type of prediction study	***Explanation***	***PROBAST+AI boxes to complete***	***Tick as appropriate***
**Development only**	**Prediction model development only, i.e., without evaluation of its performance.**	**Model development**	
**Evaluation only**	**External validation of one or more existing models in new data**	**Model evaluation**	
**Combination**	**Prediction model development combined in the same study (publication) with the evaluation of its apparent performance, internal validation performance, or external validation performance.**	**Model development and evaluation**	**X**

Step 3 - Assess quality and applicability or risk of bias and applicability

**Publication reference**	Lu YF, Ning YC, Li Y, Zhu BW, Zhang J, Yang Y, et al. Risk factor mining and prediction of urine protein progression in chronic kidney disease: a machine learning- based study. BMC Med Inform Decis Mak. 2023;23(1).
**Models of interest**	RFE-LR + soft-voting ensemble (LR + XGBoost), five individual ML classifiers and two single-marker baselines (eGFR, Scr).
**Outcome of interest**	24-h urinary protein >1 g (advanced) vs ≤1 g (mild).

Model development

**DOMAIN 1: Participants and data sources**	
**A. Quality**	
2172 CKD patients screened, 814 excluded, class imbalance corrected on training set via SMOTE, stratified 10-fold CV during model development.	
	**Y/ PY/ PN/ N/ NI**
**1.1 Were appropriate data sources used?**	Y
**1.2 Was an appropriate study design used?**	Y
**1.3 Did the in- and exclusions of study participants result in a representative dataset?**	PN
**Concern regarding quality of selection of participants and data sources**	**QUALITYCONCERN:** *low/**high**/unclear*
Rationale of quality rating: Single-centre tertiary cohort with extensive exclusions (transplantation, dialysis, multiple comorbidities), SMOTE applied without subsequent probability recalibration.	
**B. Applicability**	
Tertiary Chinese nephrology cohort , 67% male, mean age 51 y, broad blood biochemistry, immunology, lipid, vitamin, mineral and complete blood count panels collected during inpatient or outpatient admission.	
**Concern that the (data of the) included participants do not match the review question or the assessor's intended use of the prediction model**	**APPLICABILITY CONCERN:** *low/**high**/unclear*
Rationale of applicability rating: Extensive exclusions and tertiary case-mix limit applicability to general CKD outpatient follow-up.	

**DOMAIN 2: Predictors**	
**A. Quality**	
100 candidate predictors, RFE-LR reduced to 17, categorical features one-hot-encoded, continuous variables imputed via random forest.	
	**Y/ PY/ PN/ N/ NI**
**2.1 Were predictors defined and assessed in a similar way for all participants?**	Y
**2.2 Was any pre-processing of predictors similar for all participants?**	Y

**2.3 Were predictor assessments made without knowledge of outcome data?**	PN
**2.4 Were the predictors included in the model available at the time the model was intended to be used?**	Y
**Concern regarding quality of selection of participants and data sources**	**QUALITYCONCERN:** *low/**high**/unclear*
RFE feature selection performed using outcome labels and on the full dataset before splitting, random-forest imputation prior to splitting, likely data leakage.	
**B. Applicability**	
**Concern that the definition, pre-processing, assessment, or timing of assessment of the predictors in the model do not match the review question or the assessor's intended use**	**APPLICABILITY CONCERN:** ***low**/high/unclear*
Rationale of applicability rating: Selected predictors are largely routine biochemistry, immunology and vitamin measurements, feasible in hospital settings but some (lambda light chain, 25-OH vit D, retinol-binding protein) less common in primary care.	

**DOMAIN 3: Outcome**	
**A. Quality**	
24-h urinary protein dichotomised at 1 g/24 h, gold-standard 24-h urine collection, same-visit ascertainment.	
Or	**Y/ PY/ PN/ N/ NI**
**3.1 Were outcomes defined and assessed appropriately?**	Y
**3.2 Were outcomes defined and assessed in a similar way for all participants?**	Y
**3.3 Were outcome assessments made without use or knowledge of predictor data?**	PY
**3.4 Was the time interval between predictor assessment and outcome assessment appropriate?**	Y
**Concern regarding quality of the outcome or its determination**	**QUALITYCONCERN:** ***low**/high/unclear*
Standard 1 g/24 h cut-off applied uniformly, same-visit ascertainment removes interval bias.	
**B. Applicability**	
Outcome determined at the same hospital admission as predictors, single binary outcome, class distribution 906 advanced (67%) / 452 mild (33%).	
**Concern that the outcome, its definition, assessment, or timing of assessment do not match the review question or the assessor's intended use**	**APPLICABILITY CONCERN:** ***low**/high/unclear*
1 g/24 h threshold is clinically relevant for CKD risk stratification.	

**DOMAIN 4: Analysis**
**A. Quality**
1086 train / 272 test (after 80/20 split), SMOTE applied to training only, 725/725 balanced training set, 17 predictors after RFE-LR.

XGBoost, GNB, NN, ridge, LR, each tuned by grid search with 10-fold stratified CV, soft-voting ensemble, RFE-LR selects 17 features, SHAP on ensemble, plots for top features.
AUROC, PR curves, sensitivity, specificity, accuracy, AP, no calibration plot, no decision-curve analysis.
Missing data: <10% missing on most features, categorical imputed by mode, continuous by random-forest imputation.

	Y/ PY/ PN/ N/ NI
**4.1 Was there evidence that the sample size was reasonable?**	Y
**4.2 Were continuous and categorical predictors handled appropriately?**	Y
**4.3 Were participants with missing or censored data handled appropriately in the analysis?**	PN
**4.4 If methods to address class imbalance were used, was the model or the model predictions recalibrated?***	PN
**4.5 Were methods used to address potential model overfitting?**	PY
**Concern regarding quality of the analysis**	**QUALITYCONCERN:** *low/**high**/unclear*
RF imputation and RFE feature selection likely done before train/test split, SMOTE applied without probability recalibration, no calibration or decision-curve analysis.	

Model evaluation

Domain 1,2 and 3 same analysis as model development

**DOMAIN 4: Analysis**
**Risk of bias**
272 test (153 advanced, 119 mild).
AUROC, PR curves, sensitivity, specificity, accuracy, AP, no calibration plot, no decision-curve analysis.
Describe any participants who were excluded from the analysis: Exclusion same as development
Describe missing data on predictors and outcomes as well as methods used for handling these missing data: Same as development

	Y/ PY/ PN/ N/ NI/ NA		
**4.1 Was model evaluation based on only apparent performance avoided?**	Y		
	**A**	**I**	**E**
**4.2 Was there evidence that the sample size was reasonable?**	NA	Y	NA
**4.3 Were participants with missing or censored data handled appropriately in the analysis?**	NA	PN	NA
**4.4 If methods to address class imbalance were used, was the evaluation done in a dataset without imbalance correction?***	NA	PY	NA
**4.5 If data splitting was done to create training and test datasets, was there evidence that data leakage was avoided?***	**NA**	PN	**NA**
**4.6 If resampling methods were used to evaluate model performance, were all**	**NA**	PN	**NA**

**model development steps replicated in the resampling process?***			
**4.7 Was the predictive performance of the model evaluated appropriately, e.g., calibration, discrimination, and net benefit?**	NA	PN	NA
**Risk of bias introduced by the analysis**	**RISK OF BIAS:** *low/**high**/unclear*		
RFE feature selection and RF imputation likely performed pre-split, SMOTE on training without probability recalibration, no calibration / decision-curve analysis, no external validation.			

Step 4 - Assess the overall concerns regarding quality, risk of bias and applicability of the prediction model

**OVERALL JUDGEMENT OF PREDICTION MODEL**	
**Overall judgement of quality (development)**	**QUALITY CONCERN:** *low/**high**/unclear*
Single-centre, restrictive exclusions, methodological leakage, no calibration.	
**Overall judgement of risk of bias (evaluation)**	**RISK OF BIAS:** *low/**high**/unclear*
Internal hold-out only, data-leakage concerns, class-imbalance not addressed with recalibration.	
**Overall judgement of applicability (development)**	**APPLICABILITY CONCERN:** *low/**high**/unclear*
Tertiary case-mix and exclusions limit applicability to general outpatient CKD population.	
**Overall judgement of applicability (evaluation)**	**APPLICABILITY CONCERN:** *low/**high**/unclear*
Single Shanghai tertiary cohort.	

Reddy et al. (62)

Step 1 - PICOTS

**Population**	Australian tertiary-centre pathology cohort, external validation: Japanese CKD-ROUTE registry
**Index model(s)**	Decision tree (CART), RF, RNN
**Comparator model(s)**	Inter-model comparison, no external KFRE benchmark.
**Outcome(s)**	Kidney failure, defined as two eGFR readings <15 mL/min/1.73 m² ≥30 days apart.
**Timing**	prediction made from baseline eGFR trajectory; horizon is until first KF event
**Setting and intended use of the prediction model**	Australian tertiary pathology service / EHR-integrated risk prediction, intended for early

	identification of patients likely to progress to kidney failure

Step 2 – Classify the type of prediction model assessment

**Classify the assessment based on its aim**			
Type of prediction study	***Explanation***	***PROBAST+AI boxes to complete***	***Tick as appropriate***
**Development only**	**Prediction model development only, i.e., without evaluation of its performance.**	**Model development**	
**Evaluation only**	**External validation of one or more existing models in new data**	**Model evaluation**	
**Combination**	**Prediction model development combined in the same study (publication) with the evaluation of its apparent performance, internal validation performance, or external validation performance.**	**Model development and evaluation**	**X**

Step 3 - Assess quality and applicability or risk of bias and applicability

**Publication reference**	Reddy S, Roy S, Choy KW, Sharma S, Dwyer KM, Manapragada C, et al. Predicting chronic kidney disease progression using small pathology datasets and explainable machine learning models. Computer Methods and Programs in Biomedicine Update. 2024;6.
**Models of interest**	Decision tree, random forest, RNN with five-variable input.
**Outcome of interest**	Kidney failure (eGFR <15 confirmed ≥30 d apart).

Model development

**DOMAIN 1: Participants and data sources**	
**A. Quality**	
Australian cohort: final n=10,064 (353 KF / 9711 non-KF). Centroid-cluster (K-means) under-sampling of majority class to match minority, 353:353 balanced training sample.	
	**Y/ PY/ PN/ N/ NI**
**1.1 Were appropriate data sources used?**	Y
**1.2 Was an appropriate study design used?**	Y
**1.3 Did the in- and exclusions of study participants result in a representative dataset?**	PY

**Concern regarding quality of selection of participants and data sources**	**QUALITYCONCERN:** ***low****/high/unclear*
Rationale of quality rating: Large pathology data source spanning 5 y, clear inclusion/exclusion, under-sampling addresses class imbalance for training only, centroid-based under-sampling discards majority data and may bias decision boundaries.	
**B. Applicability**	
Australian cohort, age 77.5±12.0 y (eGFR mean 46.6±15.7), 50% male, KF prevalence 3.5%, Japanese cohort: mean age 71.5 y, eGFR ~36.6, KF prevalence 27%, more advanced CKD cohort .	
**Concern that the (data of the) included participants do not match the review question or the assessor's intended use of the prediction model**	**APPLICABILITY CONCERN:** ***low****/high/unclear*
Rationale of applicability rating: Two routinely-collected pathology cohorts from primary/tertiary services align well with intended pathology-data-driven referral use, case-mix differences between Australian and Japanese cohorts addressed via transfer learning.	

**DOMAIN 2: Predictors**	
**A. Quality**	
age, sex, most recent eGFR, eGFR slope (linear regression over time-series of eGFR), mean eGFR, Japanese eGFR re-computed from back-calculated SCr to harmonise MDRD/CKD-EPI.	
	**Y/ PY/ PN/ N/ NI**
**2.1 Were predictors defined and assessed in a similar way for all participants?**	Y
**2.2 Was any pre-processing of predictors similar for all participants?**	Y
**2.3 Were predictor assessments made without knowledge of outcome data?**	Y
**2.4 Were the predictors included in the model available at the time the model was intended to be used?**	Y
**Concern regarding quality of selection of participants and data sources**	**QUALITYCONCERN:** ***low****/high/unclear*
Standardised pathology-derived predictors, explicit equation-harmonisation between cohorts, predictors readily available pre-outcome.	
**B. Applicability**	
**Concern that the definition, pre-processing, assessment, or timing of assessment of the predictors in the model do not match the review question or the assessor's intended use**	**APPLICABILITY CONCERN:** ***low****/high/unclear*
Rationale of applicability rating: Five routine pathology-derived variables, universally available in any clinical chemistry workflow.	

**DOMAIN 3: Outcome**	
**A. Quality**	
Kidney failure = ≥2 eGFR <15 mL/min/1.73 m² with ≥30-day interval.	
Or	**Y/ PY/ PN/ N/ NI**
**3.1 Were outcomes defined and assessed appropriately?**	PY
**3.2 Were outcomes defined and assessed in a similar way for all participants?**	Y

**3.3 Were outcome assessments made without use or knowledge of predictor data?**	PY
**3.4 Was the time interval between predictor assessment and outcome assessment appropriate?**	PY
**Concern regarding quality of the outcome or its determination**	**QUALITYCONCERN:** ***low****/high/unclear*
Lab-based eGFR <15 criterion uniformly applied, sensible exclusion of likely dialysis-affected values, but no fixed prediction horizon.	
**B. Applicability**	
Outcome ascertained any time within the 5-year pathology window, no fixed prediction horizon. Single biochemical outcome, KF prevalence 3.5% vs 27%.	
**Concern that the outcome, its definition, assessment, or timing of assessment do not match the review question or the assessor's intended use**	**APPLICABILITY CONCERN:** ***low****/high/unclear*
eGFR-based KF is useful proxy for clinical kidney failure, ignores patients who started KRT with eGFR >15 and lacks time-to-event framing.	

**DOMAIN 4: Analysis**	
**A. Quality**	
Australian under-sampled training balance ~353/353 events, stratified 5-fold CV, Japanese 15% fine-tuning (n=89), 85% held-out testing (n=508).	
Decision tree, random forest, Simple RNN, for DT/RF only five tabular features used, RNN uses time-series eGFR with age/gender, SHAP, LIME and DiCE counterfactual analysis on the final decision tree.	
Accuracy, precision, recall, F1, specificity, AUROC, SHAP/LIME for local/global interpretability.	
Missing data: not explicitly described; mean ≥3 eGFR tests required (implicit minimum density).	
	**Y/ PY/ PN/ N/ NI**
**4.1 Was there evidence that the sample size was reasonable?**	PY
**4.2 Were continuous and categorical predictors handled appropriately?**	Y
**4.3 Were participants with missing or censored data handled appropriately in the analysis?**	NI
**4.4 If methods to address class imbalance were used, was the model or the model predictions recalibrated?***	PN
**4.5 Were methods used to address potential model overfitting?**	PY
**Concern regarding quality of the analysis**	**QUALITYCONCERN:** *low/****high****/unclear*
Centroid under-sampling applied without subsequent probability recalibration, only 5 predictors but no calibration/decision-curve analysis, missing-data handling not described, small fine-tuning subset for transfer learning may overfit.	

Model evaluation

Domain 1,2 and 3 same analysis as model development

**DOMAIN 4: Analysis**
**Risk of bias**

Australian, 5-fold CV ~141 train/35 test per fold, Japanese: 89 fine-tune, 508 test (with ~138 events).			
Accuracy, precision, recall, F1, specificity, AUROC, SHAP/LIME for local/global interpretability.			
Describe any participants who were excluded from the analysis: Exclusion same as development			
Describe missing data on predictors and outcomes as well as methods used for handling these missing data: Same as development			
	**Y/ PY/ PN/ N/ NI/ NA**		
**4.1 Was model evaluation based on only apparent performance avoided?**	Y		
	**A**	**I**	**E**
**4.2 Was there evidence that the sample size was reasonable?**	NA	Y	PY
**4.3 Were participants with missing or censored data handled appropriately in the analysis?**	NA	NI	NI
**4.4 If methods to address class imbalance were used, was the evaluation done in a dataset without imbalance correction?***	NA	PN	PY
**4.5 If data splitting was done to create training and test datasets, was there evidence that data leakage was avoided?***	**NA**	Y	**NA**
**4.6 If resampling methods were used to evaluate model performance, were all model development steps replicated in the resampling process?***	**NA**	Y	**NA**
**4.7 Was the predictive performance of the model evaluated appropriately, e.g., calibration, discrimination, and net benefit?**	NA	PN	PN
**Risk of bias introduced by the analysis**	**RISK OF BIAS:** *low/**high**/unclear*		
External transfer-learning validation, evaluation under-sampled balanced, no calibration plot or decision-curve analysis presented, AUROCs near 0.98 internally likely inflated by under-sampling.			

Step 4 - Assess the overall concerns regarding quality, risk of bias and applicability of the prediction model

OVERALL JUDGEMENT OF PREDICTION MODEL	
**Overall judgement of quality (development)**	**QUALITY CONCERN:** *low/**high**/unclear*
Five-feature pathology model with under-sampling but no probability recalibration, no calibration / DCA.	
**Overall judgement of risk of bias (evaluation)**	**RISK OF BIAS:** *low/**high**/unclear*
Internal AUCs inflated by under-sampling balance, no calibration, transfer-learning on small subset.	
**Overall judgement of applicability (development)**	**APPLICABILITY CONCERN:** ***low**/high/unclear*
Routine pathology variables align well with intended use as an EHR-integrated alert.	

**Overall judgement of applicability (evaluation)**	**APPLICABILITY CONCERN:** ***low****/high/unclear*
Two international cohorts (Australia + Japan) with eGFR harmonisation support cross-setting applicability.	

Shih et al. (63)

Step 1 - PICOTS

**Population**	50,174 individuals from centres in Taiwan
**Index model(s)**	CART or XGBoost classifier
**Comparator model(s)**	Vanilla CART, vanilla XGBoost.
**Outcome(s)**	Progression to CKD third stage (binary), defined by eGFR (MDRD) thresholds.
**Timing**	one-shot binary classification of cross-sectional, repeated examinations across the 5-year
**Setting and intended use of the prediction model**	Community chain-clinic and health-examination context in Taiwan, intended to support personalised early prevention.

Step 2 – Classify the type of prediction model assessment

**Classify the assessment based on its aim**			
Type of prediction study	***Explanation***	***PROBAST+AI boxes to complete***	***Tick as appropriate***
**Development only**	**Prediction model development only, i.e., without evaluation of its performance.**	**Model development**	
**Evaluation only**	**External validation of one or more existing models in new data**	**Model evaluation**	
**Combination**	**Prediction model development combined in the same study (publication) with the evaluation of its apparent performance, internal validation performance, or external validation performance.**	**Model development and evaluation**	**X**

Step 3 - Assess quality and applicability or risk of bias and applicability

**Publication reference**	Shih CC, Chen SH, Chen GD, Chang CC, Shih YL. Development of a Longitudinal Diag-nosis and Prognosis in Patients with Chronic

	Kidney Disease: Intelligent Clinical Deci-sion-Making Scheme. Int J Environ Res Public Health. 2021;18(23).
**Models of interest**	CART/XGBoost
**Outcome of interest**	Third-stage CKD progression.

Model development

**DOMAIN 1: Participants and data sources**	
**A. Quality**	
50,174 records from 32 chain clinics + 3 health-check centres in Taiwan, training 30,106 (34.69% positives), testing 20,068 (same prevalence).	
	**Y/ PY/ PN/ N/ NI**
**1.1 Were appropriate data sources used?**	PY
**1.2 Was an appropriate study design used?**	PN
**1.3 Did the in- and exclusions of study participants result in a representative dataset?**	NI
**Concern regarding quality of selection of participants and data sources**	**QUALITYCONCERN:** *low/**high**/unclear*
Rationale of quality rating: Inclusion/exclusion criteria not described, unclear whether records are patient-level or visit-level, "longitudinal" claim is not operationalised as time-to-event.	
**B. Applicability**	
Taiwanese community chain-clinic and health-check attendees, demographic, examination, blood biochemistry, and lifestyle (smoking) variables collected, ages and CKD-stage breakdown not reported in detail.	
**Concern that the (data of the) included participants do not match the review question or the assessor's intended use of the prediction model**	**APPLICABILITY CONCERN:** *low/**high**/unclear*
Rationale of applicability rating: Absence of clear cohort description limits assessment of fit to community-screening intended use.	

**DOMAIN 2: Predictors**	
**A. Quality**	
Examination date, sex, date of birth, height, weight, systolic / diastolic pressure, urine PCR, urine ACR, uric acid, serum creatinine, eGFR (MDRD), cholesterol, LDL, HbA1c, fasting glucose, hemoglobin A1c, CKD stage, comorbidity, smoking.	
	**Y/ PY/ PN/ N/ NI**
**2.1 Were predictors defined and assessed in a similar way for all participants?**	PY
**2.2 Was any pre-processing of predictors similar for all participants?**	Y
**2.3 Were predictor assessments made without knowledge of outcome data?**	NI
**2.4 Were the predictors included in the model available at the time the model was intended to be used?**	PY

**Concern regarding quality of selection of participants and data sources**	**QUALITYCONCERN:** *low/high/**uncle**ar*
Predictors are clinically standard but pre-processing strategies intermix predictor and outcome handling, CKD stage may overlap with outcome definition.	
**B. Applicability**	
**Concern that the definition, pre-processing, assessment, or timing of assessment of the predictors in the model do not match the review question or the assessor's intended use**	**APPLICABILITY CONCERN:** ***low**/high/unclear*
Rationale of applicability rating: Predictors readily available in community / chain-clinic settings.	

**DOMAIN 3: Outcome**	
**A. Quality**	
Binary "progression to third-stage CKD" derived from eGFR (MDRD), definition and timing not explicitly stated.	
Or	**Y/ PY/ PN/ N/ NI**
**3.1 Were outcomes defined and assessed appropriately?**	PN
**3.2 Were outcomes defined and assessed in a similar way for all participants?**	PY
**3.3 Were outcome assessments made without use or knowledge of predictor data?**	NI
**3.4 Was the time interval between predictor assessment and outcome assessment appropriate?**	NI
**Concern regarding quality of the outcome or its determination**	**QUALITYCONCERN:** *low/**high**/unclear*
Outcome ascertainment, follow-up interval and possible overlap with predictor "CKD stage" not adequately described, longitudinal claim not operationalised.	
**B. Applicability**	
Time point at which third-stage CKD progression was determined not reported, no fixed prediction horizon, single eGFR-derived outcome.	
**Concern that the outcome, its definition, assessment, or timing of assessment do not match the review question or the assessor's intended use**	**APPLICABILITY CONCERN:** *low/high/**unclear***
Stage-3 progression is clinically relevant but timing of progression is not defined.	

**DOMAIN 4: Analysis**
**A. Quality**
Training 30,106 (≈10,444 events), testing 20,068.
CART or XGBoost base classifier, main-effect plots used to choose final strategy combination; k-fold CV; grid + random search; bagged-tree imputation; oversampling for class balance; correspondence analysis and Lasso for feature handling.
Accuracy, balanced accuracy, sensitivity, specificity, precision, F1, AUROC.

Missing data: bagged regression-tree imputation when strategy chosen.	
	**Y/ PY/ PN/ N/ NI**
**4.1 Was there evidence that the sample size was reasonable?**	Y
**4.2 Were continuous and categorical predictors handled appropriately?**	PY
**4.3 Were participants with missing or censored data handled appropriately in the analysis?**	PY
**4.4 If methods to address class imbalance were used, was the model or the model predictions recalibrated?***	PN
**4.5 Were methods used to address potential model overfitting?**	PY
**Concern regarding quality of the analysis**	**QUALITYCONCERN:** *low/**high**/unclear*
Taguchi strategy selection done on training balanced accuracy, oversampling not followed by probability recalibration, no calibration plot, no decision-curve analysis, risk-factor "importance" generated for interaction terms with unclear interpretation.	

Model evaluation

Domain 1,2 and 3 same analysis as model development

**DOMAIN 4: Analysis**			
**Risk of bias**			
20,068 test (~6960 events).			
Accuracy, balanced accuracy, sensitivity, specificity, precision, F1, AUROC.			
Describe any participants who were excluded from the analysis: Exclusion same as development			
Describe missing data on predictors and outcomes as well as methods used for handling these missing data: Same as development			
	**Y/ PY/ PN/ N/ NI/ NA**		
**4.1 Was model evaluation based on only apparent performance avoided?**	Y		
	**A**	**I**	**E**
**4.2 Was there evidence that the sample size was reasonable?**	NA	Y	NA
**4.3 Were participants with missing or censored data handled appropriately in the analysis?**	NA	PY	NA
**4.4 If methods to address class imbalance were used, was the evaluation done in a dataset without imbalance correction?***	NA	PN	NA
**4.5 If data splitting was done to create training and test datasets, was there evidence that data leakage was avoided?***	**NA**	PN	**NA**
**4.6 If resampling methods were used to evaluate model performance, were all model development steps replicated in the resampling process?***	**NA**	PN	**NA**
**4.7 Was the predictive performance of the model evaluated appropriately, e.g., calibration, discrimination, and net benefit?**	NA	PN	NA
**Risk of bias introduced by the analysis**	**RISK OF BIAS:** *low/**high**/unclear*		

Strategy-selection on the same training data risks selection bias/data leakage, evaluation on oversampled data not clearly separated, no external validation, no calibration/ decision-curve assessment.

Step 4 - Assess the overall concerns regarding quality, risk of bias and applicability of the prediction model

OVERALL JUDGEMENT OF PREDICTION MODEL	
**Overall judgement of quality (development)**	**QUALITY CONCERN:** *low/**high**/unclear*
Outcome ascertainment, strategy-selection methodology, no calibration.	
**Overall judgement of risk of bias (evaluation)**	**RISK OF BIAS:** *low/**high**/unclear*
Internal hold-out only, possible data leakage via Taguchi strategy selection, no calibration.	
**Overall judgement of applicability (development)**	**APPLICABILITY CONCERN:** *low/**high**/unclear*
Cohort and outcome definitions not described in sufficient detail to confirm applicability to intended community screening.	
**Overall judgement of applicability (evaluation)**	**APPLICABILITY CONCERN:** *low/**high**/unclear*
Single-system Taiwanese dataset, no external validation.	

Liang et al. (64)

Step 1 - PICOTS

**Population**	1765 CKD patients followed from Chinese hospital.
**Index model(s)**	Four-layer fully-connected NN
**Comparator model(s)**	Logistic regression, LASSO, ridge-regression classification (RRC), SVM-RBF, SVM-Linear, RF, XGBoost.
**Outcome(s)**	ESRD onset within 3 years from baseline, defined as dialysis initiation, kidney transplantation, or eGFR reduced by ≥50% during follow-up.
**Timing**	3-year prediction horizon from baseline
**Setting and intended use of the prediction model**	Tertiary setting, intended to inform individualised prognosis and identify novel CKD progression markers.

Step 2 – Classify the type of prediction model assessment

**Classify the assessment based on its aim**			
Type of prediction study	***Explanation***	***PROBAST+AI boxes to complete***	***Tick as appropriate***

**Development only**	**Prediction model development only, i.e., without evaluation of its performance.**	**Model development**	
**Evaluation only**	**External validation of one or more existing models in new data**	**Model evaluation**	
**Combination**	**Prediction model development combined in the same study (publication) with the evaluation of its apparent performance, internal validation performance, or external validation performance.**	**Model development and evaluation**	**X**

Step 3 - Assess quality and applicability or risk of bias and applicability

**Publication reference**	Liang P, Yang J, Wang W, Yuan G, Han M, Zhang Q, et al. Deep Learning Identifies Intel-ligible Predictors of Poor Prognosis in Chronic Kidney Disease. IEEE J Biomed Health In-form. 2023;27(7):3677–85.
**Models of interest**	NN, LR, LASSO, RRC, SVM, RF, XGBoost
**Outcome of interest**	ESRD onset within 3 years.

Model development

**DOMAIN 1: Participants and data sources**	
**A. Quality**	
1765 patients, end-stage ratio overall 41%.	
	**Y/ PY/ PN/ N/ NI**
**1.1 Were appropriate data sources used?**	Y
**1.2 Was an appropriate study design used?**	PY
**1.3 Did the in- and exclusions of study participants result in a representative dataset?**	PN
**Concern regarding quality of selection of participants and data sources**	**QUALITYCONCERN:** *low/**high**/unclear*
Rationale of quality rating: Single-centre cohort with high ESRD prevalence (41%) reflects tertiary-care case-mix and over-samples late-stage CKD, limits representativeness.	
**B. Applicability**	
Tertiary-referral Chinese cohort, high prevalence of advanced CKD, predictors include broad blood biochemistry, urinary indicators, immunology and ICD-10 comorbidities.	
**Concern that the (data of the) included participants do not match the review question or the assessor's intended use of the prediction model**	**APPLICABILITY CONCERN:** *low/**high**/unclear*

Rationale of applicability rating: Late-stage / tertiary case-mix and high ESRD prevalence limit applicability to earlier-stage CKD outpatient care.

**DOMAIN 2: Predictors**	
**A. Quality**	
97 demographic, comorbidity and biochemistry variables categorical features dummy-encoded, continuous z-scored.	
	**Y/ PY/ PN/ N/ NI**
**2.1 Were predictors defined and assessed in a similar way for all participants?**	PY
**2.2 Was any pre-processing of predictors similar for all participants?**	Y
**2.3 Were predictor assessments made without knowledge of outcome data?**	PY
**2.4 Were the predictors included in the model available at the time the model was intended to be used?**	Y
**Concern regarding quality of selection of participants and data sources**	**QUALITYCONCERN:** ***low****/high/unclear*
Broad routine laboratory predictors collected consistently, missing values imputed within each CKD stage by mean.	
**B. Applicability**	
**Concern that the definition, pre-processing, assessment, or timing of assessment of the predictors in the model do not match the review question or the assessor's intended use**	**APPLICABILITY CONCERN:** ***low****/high/unclear*
Rationale of applicability rating: Predictors are standard-care biochemistry, broadly applicable.	

**DOMAIN 3: Outcome**	
**A. Quality**	
ESRD defined as dialysis, transplantation, or eGFR reduction ≥50% during the observation period.	
Or	**Y/ PY/ PN/ N/ NI**
**3.1 Were outcomes defined and assessed appropriately?**	PY
**3.2 Were outcomes defined and assessed in a similar way for all participants?**	Y
**3.3 Were outcome assessments made without use or knowledge of predictor data?**	PY
**3.4 Was the time interval between predictor assessment and outcome assessment appropriate?**	PY
**Concern regarding quality of the outcome or its determination**	**QUALITYCONCERN:** ***low****/high/unclear*
Composite outcome includes both hard endpoints (dialysis/transplant) and a 50% eGFR drop	
**B. Applicability**	
Outcome assessed any time during a 3-year follow-up, totalling ~33% positive overall, varying by stage.	

Concern that the outcome, its definition, assessment, or timing of assessment do not match the review question or the assessor's intended use	APPLICABILITY CONCERN: ***low***/*high*/*unclear*
3-year ESRD progression is a clinically meaningful endpoint.	

DOMAIN 4: Analysis	
A. Quality	
1235 train, 177 validation, 353 test; 97 predictors, ~5 events per variable in training£.	
DNN with BatchNorm + Dropout, ten retrainings with different random seeds.	
Accuracy, precision, recall, AUROC, PR-AUC, F, per-stage performance (stages 1–2, 3, 4, 5).	
Missing data: mean imputation within CKD stage.	
	**Y/ PY/ PN/ N/ NI**
**4.1 Was there evidence that the sample size was reasonable?**	PN
**4.2 Were continuous and categorical predictors handled appropriately?**	Y
**4.3 Were participants with missing or censored data handled appropriately in the analysis?**	PN
**4.4 If methods to address class imbalance were used, was the model or the model predictions recalibrated?***	NI
**4.5 Were methods used to address potential model overfitting?**	Y
**Concern regarding quality of the analysis**	**QUALITYCONCERN:** *low/**high**/unclear*
Mean imputation by CKD stage may introduce leakage, no calibration or decision-curve analysis, attribution algorithms not formally validated against ground truth.	

Model evaluation

Domain 1,2 and 3 same analysis as model development

DOMAIN 4: Analysis	
Risk of bias	
353 test (~145 events).	
Accuracy, precision, recall, AUROC, PR-AUC, F, per-stage performance (stages 1–2, 3, 4, 5).	
Describe any participants who were excluded from the analysis: Exclusion same as development	
Describe missing data on predictors and outcomes as well as methods used for handling these missing data: Same as development	
	**Y/ PY/ PN/ N/ NI/ NA**

**4.1 Was model evaluation based on only apparent performance avoided?**	Y		
	**A**	**I**	**E**
**4.2 Was there evidence that the sample size was reasonable?**	NA	PN	NA
**4.3 Were participants with missing or censored data handled appropriately in the analysis?**	NA	PN	NA
**4.4 If methods to address class imbalance were used, was the evaluation done in a dataset without imbalance correction?***	NA	NA	NA
**4.5 If data splitting was done to create training and test datasets, was there evidence that data leakage was avoided?***	**NA**	PY	**NA**
**4.6 If resampling methods were used to evaluate model performance, were all model development steps replicated in the resampling process?***	**NA**	PY	**NA**
**4.7 Was the predictive performance of the model evaluated appropriately, e.g., calibration, discrimination, and net benefit?**	NA	PN	NA
**Risk of bias introduced by the analysis**	**RISK OF BIAS:** *low/**high**/unclear*		
No external validation, no calibration / decision-curve analysis, CKD-stage-conditional mean imputation may have leaked outcome-correlated information into both training and test sets.			

Step 4 - Assess the overall concerns regarding quality, risk of bias and applicability of the prediction model

**OVERALL JUDGEMENT OF PREDICTION MODEL**	
**Overall judgement of quality (development)**	**QUALITY CONCERN:** *low/**high**/unclear*
Single-centre, stage-conditional mean imputation, no calibration.	
**Overall judgement of risk of bias (evaluation)**	**RISK OF BIAS:** *low/**high**/unclear*
Internal hold-out only, no calibration, potential imputation-driven leakage.	
**Overall judgement of applicability (development)**	**APPLICABILITY CONCERN:** *low/**high**/unclear*
Tertiary-care cohort with high baseline dialysis prevalence, limited generalisation to earlier-stage outpatient CKD.	
**Overall judgement of applicability (evaluation)**	**APPLICABILITY CONCERN:** *low/**high**/unclear*
No external validation, single Chinese tertiary cohort.	

Bellocchio et al. (65)

Step 1 - PICOTS

**Population**	Non-dialysis-dependent CKD stage 3–5 adults from 15 countries
**Index model(s)**	Two Naïve Bayes Classifiers, PROGRES-CKD-6 and PROGRES-CKD-24
**Comparator model(s)**	KFRE 4-variable (4VAR) and 6-variable (6VAR) equations recalibrated for European populations.

**Outcome(s)**	KRT initiation (hemodialysis, peritoneal dialysis or kidney transplant) within 6 or 24 months from index visit.
**Timing**	6-month and 24-month prediction horizons from a randomly-selected index visit.
**Setting and intended use of the prediction model**	Outpatient nephrology clinics, intended for clinician-facing prognostic reasoning and risk stratification for intensified prevention / vascular access planning.

Step 2 – Classify the type of prediction model assessment

**Classify the assessment based on its aim**			
Type of prediction study	***Explanation***	***PROBAST+AI boxes to complete***	***Tick as appropriate***
**Development only**	**Prediction model development only, i.e., without evaluation of its performance.**	**Model development**	
**Evaluation only**	**External validation of one or more existing models in new data**	**Model evaluation**	
**Combination**	**Prediction model development combined in the same study (publication) with the evaluation of its apparent performance, internal validation performance, or external validation performance.**	**Model development and evaluation**	**X**

Step 3 - Assess quality and applicability or risk of bias and applicability

**Publication reference**	Bellocchio F, Lonati C, Titapiccolo JI, Nadal J, Meiselbach H, Schmid M, et al. Validation of a Novel Predictive Algorithm for Kidney Failure in Patients Suffering from Chronic Kid-ney Disease: The Prognostic Reasoning System for Chronic Kidney Disease (PRO-GRES-CKD). Int J Environ Res Public Health. 2021;18(23).
**Models of interest**	PROGRES-CKD-6 (28 vars) and PROGRES-CKD-24 (34 vars), Naïve Bayes Classifiers.
**Outcome of interest**	KRT initiation within 6 / 24 months.

Model development

**DOMAIN 1: Participants and data sources**
**A. Quality**

22,535 stage 3–5 CKD outpatients from FMC NephroCare, randomly partitioned 70/30: development, validation . KRT events: 801 (6 m) and 1817 (24 m).	
	**Y/ PY/ PN/ N/ NI**
**1.1 Were appropriate data sources used?**	Y
**1.2 Was an appropriate study design used?**	Y
**1.3 Did the in- and exclusions of study participants result in a representative dataset?**	Y
**Concern regarding quality of selection of participants and data sources**	**QUALITYCONCERN:** ***low****/high/unclear*
Rationale of quality rating: Very large, multinational, prospectively-collected registry, clear inclusion of stage 3–5 outpatients with at least one outpatient visit and one creatinine value.	
**B. Applicability**	
Outpatients in 15 countries, mean age 72; 50% male; 53% stage 3, 36% stage 4, 11% stage 5; eGFR mean 31.9. Routine clinical data including comorbidities (ICD-10), kidney biomarkers, etiology of kidney disease.	
**Concern that the (data of the) included participants do not match the review question or the assessor's intended use of the prediction model**	**APPLICABILITY CONCERN:** ***low****/high/unclear*
Rationale of applicability rating: Real-world multinational outpatient nephrology cohort closely matches intended use.	

**DOMAIN 2: Predictors**	
**A. Quality**	
28 or 34 variables — demographics, kidney function, aetiology and comorbidities, lifetime comorbidity occurrence abstracted from HER, biomarkers averaged over 12 months pre-index.	
	**Y/ PY/ PN/ N/ NI**
**2.1 Were predictors defined and assessed in a similar way for all participants?**	Y
**2.2 Was any pre-processing of predictors similar for all participants?**	Y
**2.3 Were predictor assessments made without knowledge of outcome data?**	Y
**2.4 Were the predictors included in the model available at the time the model was intended to be used?**	Y
**Concern regarding quality of selection of participants and data sources**	**QUALITYCONCERN:** ***low****/high/uncle*ar
Standardised codified data, predictors collected before index date, NBC handles missing predictors natively.	
**B. Applicability**	
**Concern that the definition, pre-processing, assessment, or timing of assessment of the predictors in the model do not match the review question or the assessor's intended use**	**APPLICABILITY CONCERN:** ***low****/high/unclear*
Rationale of applicability rating: Routine clinical biomarkers and comorbidities readily available in nephrology outpatient practice.	

**DOMAIN 3: Outcome**

A. Quality	
KRT initiation (hemodialysis, peritoneal dialysis, transplant) within 6 or 24 months, acute / transient dialysis excluded, patients with no further s-creatinine and no dialysis note classified as lost to follow-up.	
Or	**Y/ PY/ PN/ N/ NI**
**3.1 Were outcomes defined and assessed appropriately?**	Y
**3.2 Were outcomes defined and assessed in a similar way for all participants?**	Y
**3.3 Were outcome assessments made without use or knowledge of predictor data?**	Y
**3.4 Was the time interval between predictor assessment and outcome assessment appropriate?**	Y
**Concern regarding quality of the outcome or its determination**	**QUALITYCONCERN:** ***low***/*high/unclear*
Clinically meaningful, unambiguous KRT endpoint with appropriate time horizons (6/24 months).	
**B. Applicability**	
Outcome assessed at 6 months and at 24 months from the index visit. Composite of three KRT modalities: hemodialysis, peritoneal dialysis, or kidney transplantation;	
**Concern that the outcome, its definition, assessment, or timing of assessment do not match the review question or the assessor's intended use**	**APPLICABILITY CONCERN:** ***low***/*high/unclear*
KRT onset is the canonical endpoint for CKD progression risk scores.	

DOMAIN 4: Analysis	
**A. Quality**	
development n=17,775 (618 6-m KRT, 1280 24-m KRT events), 28–34 candidate predictors.	
Naïve Bayes Classifier, missing data handled natively, value-of-information (VOI) metrics and Normalised Likelihood per predictor, calibration assessed by observed vs predicted across quintiles of risk.	
AUROC, calibration plots by quintiles, VOI, impact metrics non-inferiority vs KFRE pre-specified and superiority, impact / NNT simulation for intensified-prevention program.	
Missing data: NBC natively handles missing variables without imputation.	
	**Y/ PY/ PN/ N/ NI**
**4.1 Was there evidence that the sample size was reasonable?**	Y
**4.2 Were continuous and categorical predictors handled appropriately?**	Y
**4.3 Were participants with missing or censored data handled appropriately in the analysis?**	Y
**4.4 If methods to address class imbalance were used, was the model or the model predictions recalibrated?***	NA
**4.5 Were methods used to address potential model overfitting?**	Y
**Concern regarding quality of the analysis**	**QUALITYCONCERN:** ***low***/*high/unclear*

Large sample, calibration assessed, native missing-data handling, explicit non-inferiority/superiority testing vs KFRE.

Model evaluation

Domain 1,2 and 3 same analysis as model development

**DOMAIN 4: Analysis**			
**Risk of bias**			
validation 6760 (248 / 537 events), GCKD 4058 (11 / 80 events).			
AUROC, calibration plots by quintiles, VOI, impact metrics non-inferiority vs KFRE pre-specified and superiority, impact / NNT simulation for intensified-prevention program.			
Describe any participants who were excluded from the analysis: Exclusion same as development			
Describe missing data on predictors and outcomes as well as methods used for handling these missing data: Same as development			
	**Y/ PY/ PN/ N/ NI/ NA**		
**4.1 Was model evaluation based on only apparent performance avoided?**	Y		
	**A**	**I**	**E**
**4.2 Was there evidence that the sample size was reasonable?**	NA	Y	Y
**4.3 Were participants with missing or censored data handled appropriately in the analysis?**	NA	Y	Y
**4.4 If methods to address class imbalance were used, was the evaluation done in a dataset without imbalance correction?***	NA	NA	NA
**4.5 If data splitting was done to create training and test datasets, was there evidence that data leakage was avoided?***	**NA**	Y	**NA**
**4.6 If resampling methods were used to evaluate model performance, were all model development steps replicated in the resampling process?***	**NA**	NA	**NA**
**4.7 Was the predictive performance of the model evaluated appropriately, e.g., calibration, discrimination, and net benefit?**	NA	Y	Y
**Risk of bias introduced by the analysis**	**RISK OF BIAS:** ***low**/high/unclear*		
Independent external validation in GCKD, AUROC, calibration and impact / NNT assessed, non-inferiority vs KFRE formally tested with appropriate statistical methods.			

Step 4 - Assess the overall concerns regarding quality, risk of bias and applicability of the prediction model

**OVERALL JUDGEMENT OF PREDICTION MODEL**	
**Overall judgement of quality (development)**	**QUALITY CONCERN:** ***low**/high/unclear*
Large multinational cohort, NBC handles missingness natively, calibration and VOI reported.	
**Overall judgement of risk of bias (evaluation)**	**RISK OF BIAS:** ***low**/high/unclear*
Internal + independent external (GCKD) validation, non-inferior/superior to KFRE, calibration and impact simulation.	

**Overall judgement of applicability (development)**	**APPLICABILITY CONCERN:** ***low**/high/unclear*
Multinational stage 3–5 outpatient nephrology cohort closely matches intended clinical use.	
**Overall judgement of applicability (evaluation)**	**APPLICABILITY CONCERN:** ***low**/high/unclear*
GCKD cohort independently confirms applicability.	

Segal et al. (66)

Step 1 - PICOTS

**Population**	~550,000 adults aged ≥18 with CKD stages 1–4 from a large US commercial health-insurance database.
**Index model(s)**	XGBoost classifier with 240 manually-engineered predictor candidates.
**Comparator model(s)**	Logistic regression with L1 and L2 regularisation, RF, CatBoost, and a deep neural network.
**Outcome(s)**	ESRD onset within 6 months of index date, defined by ≥1 of 30 ICD-9/ICD-10 ESRD codes or a dialysis procedure.
**Timing**	6-month prediction horizon from index visit.
**Setting and intended use of the prediction model**	Health-insurance payer / integrated health system; intended to trigger electronic alert to physicians to initiate nephrology referral.

Step 2 – Classify the type of prediction model assessment

**Classify the assessment based on its aim**			
Type of prediction study	***Explanation***	***PROBAST+AI boxes to complete***	***Tick as appropriate***
**Development only**	**Prediction model development only, i.e., without evaluation of its performance.**	**Model development**	
**Evaluation only**	**External validation of one or more existing models in new data**	**Model evaluation**	
**Combination**	**Prediction model development combined in the same study (publication) with the evaluation of its apparent performance, internal validation performance, or**	**Model development and evaluation**	**X**

	**external validation performance.**		

Step 3 - Assess quality and applicability or risk of bias and applicability

**Publication reference**	Segal Z, Kalifa D, Radinsky K, Ehrenberg B, Elad G, Maor G, et al. Machine learning algo-rithm for early detection of end-stage renal disease. BMC Nephrol. 2020;21(1):518.
**Models of interest**	XGBoost with Word2Vec-embedded codes, comparators LR-L1/L2, RF, CatBoost, DNN.
**Outcome of interest**	ESRD onset within 6 months (ICD codes / dialysis procedure).

Model development

**DOMAIN 1: Participants and data sources**	
**A. Quality**	
Training n=19,657 (1147 ESRD), testing n=7334 (438 ESRD; 6.0%), inclusion: adults ≥18 with CKD stages 1–4 and ≥6 months of claims history.	
	**Y/ PY/ PN/ N/ NI**
**1.1 Were appropriate data sources used?**	PY
**1.2 Was an appropriate study design used?**	Y
**1.3 Did the in- and exclusions of study participants result in a representative dataset?**	PY
**Concern regarding quality of selection of participants and data sources**	**QUALITYCONCERN:** ***low**/high/unclear*
Rationale of quality rating: Large claims dataset spanning 12 years with clear and clinically-reasonable inclusion/exclusion criteria.	
**B. Applicability**	
US commercial health-insurance beneficiaries, age 70.7 (controls) / 70.0 (cases); 50% / 47% female; over half of cases were CKD stage 4 (52.6%) at index, hospital and EMR data not linked, coding-based diagnoses only.	
**Concern that the (data of the) included participants do not match the review question or the assessor's intended use of the prediction model**	**APPLICABILITY CONCERN:** ***low**/high/unclear*
Rationale of applicability rating: Insured US adults are a relevant target for payer-side referral interventions, lower applicability to uninsured / single-payer settings and to early-stage CKD patients.	

**DOMAIN 2: Predictors**
**A. Quality**

240 manually compiled features (demographics, CCW chronic-condition flags with immediate/recent/ever windows, diagnosis/procedure counts and trends, medication NDC→RxCUI codes, medical costs, episode counts) + Word2Vec embeddings of code sequences, highly-correlated features (r>0.9) removed via filter selection.	
	**Y/ PY/ PN/ N/ NI**
**2.1 Were predictors defined and assessed in a similar way for all participants?**	Y
**2.2 Was any pre-processing of predictors similar for all participants?**	Y
**2.3 Were predictor assessments made without knowledge of outcome data?**	Y
**2.4 Were the predictors included in the model available at the time the model was intended to be used?**	Y
**Concern regarding quality of selection of participants and data sources**	**QUALITYCONCERN:** ***low***/*high*/*uncle*ar
Standardised claims-derived predictors, reproducible CCW/Word2Vec pipeline, predictors available at point of decision.	
**B. Applicability**	
**Concern that the definition, pre-processing, assessment, or timing of assessment of the predictors in the model do not match the review question or the assessor's intended use**	**APPLICABILITY CONCERN:** ***low***/*high*/*unclear*
Rationale of applicability rating: Claims codes available in payer environments but different in EMR-only or non-US settings.	

**DOMAIN 3: Outcome**	
**A. Quality**	
ESRD onset defined by ≥1 of 30 ICD-9/10 ESRD codes or a dialysis procedure within 6 months of index. No chart adjudication.	
Or	**Y/ PY/ PN/ N/ NI**
**3.1 Were outcomes defined and assessed appropriately?**	PY
**3.2 Were outcomes defined and assessed in a similar way for all participants?**	Y
**3.3 Were outcome assessments made without use or knowledge of predictor data?**	PY
**3.4 Was the time interval between predictor assessment and outcome assessment appropriate?**	Y
**Concern regarding quality of the outcome or its determination**	**QUALITYCONCERN:** ***low***/*high*/*unclear*
Code-based definition is standard for claims studies, possible misclassification but applied uniformly.	
**B. Applicability**	
Outcome ascertained at any point within the 6-month window after the index date, composite of two pathways: ≥1 of 30 ICD-9/10 ESRD codes OR a dialysis procedure code, relative contribution of each not separately reported.	
**Concern that the outcome, its definition, assessment, or timing of assessment do not match the review question or the assessor's intended use**	**APPLICABILITY CONCERN:** ***low***/*high*/*unclear*
ESRD onset within 6 months matches intended use as an early-warning referral trigger.	

DOMAIN 4: Analysis	
**A. Quality**	
19,657 train (1147 events), 7334 test (438 events), 240 candidate predictors + 100-dim embeddings × 3 (diagnoses/procedures/medications).	
XGBoost with class weighting, 4-fold CV on training set, filter feature selection on r>0.9, subgroup analysis by CKD stage, age, sex.	
C-statistic, PR-AUC, F1, sensitivity, specificity, PPV, NPV, PPV at top 1%/5% percentiles, no calibration plot, no decision-curve analysis.	
Missing data: handled implicitly by XGBoost, z-score normalisation of normally-distributed features.	
	**Y/ PY/ PN/ N/ NI**
**4.1 Was there evidence that the sample size was reasonable?**	Y
**4.2 Were continuous and categorical predictors handled appropriately?**	Y
**4.3 Were participants with missing or censored data handled appropriately in the analysis?**	PY
**4.4 If methods to address class imbalance were used, was the model or the model predictions recalibrated?***	PN
**4.5 Were methods used to address potential model overfitting?**	PY
**Concern regarding quality of the analysis**	**QUALITYCONCERN:** *low/**high**/unclear*
Class-weighting was applied without recalibration of predicted probabilities, no calibration or decision-curve assessment, PPV reported only at extreme percentiles.	

Model evaluation

Domain 1,2 and 3 same analysis as model development

DOMAIN 4: Analysis			
**Risk of bias**			
7334 / 438 (6%).			
C-statistic, PR-AUC, F1, sensitivity, specificity, PPV, NPV, PPV at top 1%/5% percentiles, no calibration plot, no decision-curve analysis.			
Describe any participants who were excluded from the analysis: Exclusion same as development			
Describe missing data on predictors and outcomes as well as methods used for handling these missing data: Same as development			
	**Y/ PY/ PN/ N/ NI/ NA**		
**4.1 Was model evaluation based on only apparent performance avoided?**	Y		
	**A**	**I**	**E**
**4.2 Was there evidence that the sample size was reasonable?**	NA	Y	NA
**4.3 Were participants with missing or censored data handled appropriately in the analysis?**	NA	PY	NA

**4.4 If methods to address class imbalance were used, was the evaluation done in a dataset without imbalance correction?***	NA	Y	NA
**4.5 If data splitting was done to create training and test datasets, was there evidence that data leakage was avoided?***	**NA**	PY	**NA**
**4.6 If resampling methods were used to evaluate model performance, were all model development steps replicated in the resampling process?***	**NA**	PY	**NA**
**4.7 Was the predictive performance of the model evaluated appropriately, e.g., calibration, discrimination, and net benefit?**	NA	PN	NA
**Risk of bias introduced by the analysis**	**RISK OF BIAS:** ***low***/*high*/*unclear*		
Strong discrimination but no external validation, no calibration plot, no decision-curve analysis, class-weighting without recalibration may distort predicted probabilities used in the alert system.			

Step 4 - Assess the overall concerns regarding quality, risk of bias and applicability of the prediction model

**OVERALL JUDGEMENT OF PREDICTION MODEL**	
**Overall judgement of quality (development)**	**QUALITY CONCERN:** *low/**high**/unclear*
Large, well-structured claims-based development with sound modelling, analysis domain HIGH due to absence of calibration and uncorrected class-weighting probabilities.	
**Overall judgement of risk of bias (evaluation)**	**RISK OF BIAS:** *low/**high**/unclear*
Internal hold-out only, no calibration, class-weighting probabilities not recalibrated.	
**Overall judgement of applicability (development)**	**APPLICABILITY CONCERN:** ***low***/*high/unclear*
Applicable to US insured CKD populations within payer / integrated systems, transferability to other healthcare systems limited.	
**Overall judgement of applicability (evaluation)**	**APPLICABILITY CONCERN:** ***low***/*high/unclear*
Single-payer US dataset.	

Tangri et al. (67)

Step 1 - PICOTS

**Population**	14,464 adults with type-2 diabetes from the CANVAS Program and CREDENCE trials (across 34 countries), pre-specified inclusion criteria of the parent trials apply
**Index model(s)**	Klinrisk: random survival forest using demographics (age, sex) and routinely-collected lab data.
**Comparator model(s)**	KDIGO heatmap (eGFR G1–G5 × albuminuria A1–A3) and refitted 4-variable KFRE.
**Outcome(s)**	CKD progression: composite of sustained 40% eGFR decline (confirmed by second eGFR) or kidney failure (chronic dialysis, transplant, or eGFR <15 ml/min/1.73 m²).
**Timing**	6-month prediction horizon from index visit.
**Setting and intended use of the prediction model**	Multinational clinical-trial cohort of diabetic kidney disease T2DM patients, intended for risk-based care integration via electronic medical records.

Step 2 – Classify the type of prediction model assessment

**Classify the assessment based on its aim**			
Type of prediction study	***Explanation***	***PROBAST+AI boxes to complete***	***Tick as appropriate***
**Development only**	**Prediction model development only, i.e., without evaluation of its performance.**	**Model development**	
**Evaluation only**	**External validation of one or more existing models in new data**	**Model evaluation**	**X**
**Combination**	**Prediction model development combined in the same study (publication) with the evaluation of its apparent performance, internal validation performance, or external validation performance.**	**Model development and evaluation**	

Step 3 - Assess quality and applicability or risk of bias and applicability

**Publication reference**	Tangri N, Ferguson TW, Bamforth RJ, Leon SJ, Arnott C, Mahaffey KW, et al. Machine learning for prediction of chronic kidney disease progression: Validation of the Klinrisk model in the CANVAS Program and CREDENCE trial. Diabetes, obesity & metabolism. 2024;26(8):3371–80.
**Models of interest**	Klinrisk RF.
**Outcome of interest**	CKD progression: 40% eGFR decline or kidney failure at 1, 2, 3 years.

Model evaluation

**DOMAIN 1: Participants and data sources**	
**A. Quality**	
Pooled cohort of 14,464 T2DM patients from CANVAS (n=4,330), CANVAS-R (n=5,812), and CREDENCE (n=4,401) RCTs across 24–34 countries. CANVAS inclusion: T2DM with high CV risk, eGFR ≥30 ml/min/1.73 m²; CREDENCE inclusion: T2DM with diabetic kidney disease (eGFR 30–<90, urine ACR 300–5,000 mg/g).	
	**Y/ PY/ PN/ N/ NI**
**1.1 Were appropriate data sources used?**	Y
**1.2 Was an appropriate study design used?**	Y
**1.3 Did the in- and exclusions of study participants result in a representative dataset?**	PY
**Concern regarding quality of selection of participants and data sources**	**QUALITYCONCERN:** ***low***/*high*/*unclear*
Rationale of quality rating: Prospectively-collected rigorously-monitored RCT data across two large multinational trial programs, explicit inclusion/exclusion criteria, only 79 patients excluded for missing eGFR.	
**B. Applicability**	
Multinational (24–34 countries) T2DM trial population, CANVAS skews to preserved kidney function with high CV risk, CREDENCE captures established diabetic kidney disease	
**Concern that the (data of the) included participants do not match the review question or the assessor's intended use of the prediction model**	**APPLICABILITY CONCERN:** ***low***/*high*/*unclear*
Rationale of applicability rating: Trial-selected T2DM populations (high CV risk in CANVAS; established diabetic kidney disease in CREDENCE) are slightly narrower than the general T2DM population but well-aligned with the intended use of risk-based CKD-progression care in T2DM.	

**DOMAIN 2: Predictors**	
**A. Quality**	
Klinrisk predictors at trial baseline (day 1 visit), eGFR recalculated using CKD-EPI 2009 in CREDENCE (originally MDRD in CANVAS-R), glucose missing in CANVAS-R (40%) imputed by random-forest imputation embedded in the Klinrisk model, missingness <3% for all other labs, complete-case sensitivity analysis confirmed findings.	
	**Y/ PY/ PN/ N/ NI**
**2.1 Were predictors defined and assessed in a similar way for all participants?**	Y
**2.2 Was any pre-processing of predictors similar for all participants?**	Y
**2.3 Were predictor assessments made without knowledge of outcome data?**	Y

**2.4 Were the predictors included in the model available at the time the model was intended to be used?**	Y
**Concern regarding quality of selection of participants and data sources**	**QUALITYCONCERN:** ***low****/high/uncle*ar
Standardised trial laboratory measurements with quality assurance, uniform CKD-EPI 2009 recalculation, complete-case sensitivity confirms robustness.	
**B. Applicability**	
**Concern that the definition, pre-processing, assessment, or timing of assessment of the predictors in the model do not match the review question or the assessor's intended use**	**APPLICABILITY CONCERN:** ***low****/high/unclear*
Rationale of applicability rating: Klinrisk predictors are routinely-collected lab data widely available in any T2DM care setting, designed for EMR/laboratory-information-system integration.	

**DOMAIN 3: Outcome**	
**A. Quality**	
Composite CKD progression outcome: sustained 40% eGFR decline confirmed by a second eGFR following the first eGFR showing the decline, or kidney failure, pre-specified and blindly adjudicated in both parent RCTs, endpoints assessed at 1, 2, and 3 years from baseline.	
Or	**Y/ PY/ PN/ N/ NI**
**3.1 Were outcomes defined and assessed appropriately?**	Y
**3.2 Were outcomes defined and assessed in a similar way for all participants?**	Y
**3.3 Were outcome assessments made without use or knowledge of predictor data?**	Y
**3.4 Was the time interval between predictor assessment and outcome assessment appropriate?**	Y
**Concern regarding quality of the outcome or its determination**	**QUALITYCONCERN:** ***low****/high/unclear*
Pre-specified, blindly-adjudicated composite endpoint with confirmation requirement for the 40% eGFR-decline component, standard CKD-progression endpoint used in regulatory and KDIGO contexts.	
**B. Applicability**	
Outcome ascertained at any point within the 6-month window after the index date, composite of two pathways: ≥1 of 30 ICD-9/10 ESRD codes OR a dialysis procedure code, relative contribution of each not separately reported.	
**Concern that the outcome, its definition, assessment, or timing of assessment do not match the review question or the assessor's intended use**	**APPLICABILITY CONCERN:** ***low****/high/unclear*
40% eGFR decline + kidney failure is the canonical CKD-progression endpoint for risk prediction and matches model's intended use for risk-based care.	

**DOMAIN 4: Analysis**
**Risk of bias**
14,464 patients (CANVAS n=7,942; CREDENCE n=6,522), 696 with 40% eGFR decline at 2 years, 158 with kidney failure, mean baseline eGFR 71.8, median ACR 3.8 mg/mmol.

AUROC, Brier score, calibration plot, sensitivity analysis
Describe any participants who were excluded from the analysis:<br><3% for most labs.
Describe missing data on predictors and outcomes as well as methods used for handling these missing data:<br>Glucose missing in CANVAS-R (40%) imputed by random-forest imputation embedded in the Klinrisk model, complete-case sensitivity analysis confirmed findings

	Y/ PY/ PN/ N/ NI/ NA		
**4.1 Was model evaluation based on only apparent performance avoided?**	Y		
	**A**	**I**	**E**
**4.2 Was there evidence that the sample size was reasonable?**	NA	NA	Y
**4.3 Were participants with missing or censored data handled appropriately in the analysis?**	NA	NA	Y
**4.4 If methods to address class imbalance were used, was the evaluation done in a dataset without imbalance correction?***	NA	NA	NA
**4.5 If data splitting was done to create training and test datasets, was there evidence that data leakage was avoided?***	**NA**	NA	**NA**
**4.6 If resampling methods were used to evaluate model performance, were all model development steps replicated in the resampling process?***	**NA**	NA	**NA**
**4.7 Was the predictive performance of the model evaluated appropriately, e.g., calibration, discrimination, and net benefit?**	NA	NA	Y
**Risk of bias introduced by the analysis**	**RISK OF BIAS:**<br>***low**/high/unclear*		
Independent external validation in large pooled multinational trial cohort, calibration, discrimination, NRI, and clinical-relevance metrics (OR by risk strata, treatment-interaction test) all reported, complete-case sensitivity analysis confirms robustness to imputation, outperforms KDIGO heatmap and refitted KFRE.			

Step 4 - Assess the overall concerns regarding quality, risk of bias and applicability of the prediction model

**OVERALL JUDGEMENT OF PREDICTION MODEL**	
**Overall judgement of quality (development)**	**QUALITY CONCERN:**<br>*low/high/unclear*
Not assessed	
**Overall judgement of risk of bias (evaluation)**	**RISK OF BIAS:**<br>***low**/high/unclear*
Independent external multinational validation, comprehensive AUC/Brier/calibration/NRI assessment, outperforms standard-of-care KDIGO heatmap and refitted KFRE.	
**Overall judgement of applicability (development)**	**APPLICABILITY CONCERN:**<br>*low/high/unclear*
Not assessed	
**Overall judgement of applicability (evaluation)**	**APPLICABILITY CONCERN:**<br>***low**/high/unclear*
Pooled CANVAS/CREDENCE multinational T2DM cohort spans 34 countries, broad applicability to T2DM CKD progression risk prediction, limited to T2DM with elevated CV risk or diabetic kidney disease.	

Ou et al. (68)

Step 1 - PICOTS

**Population**	53,477 newly-diagnosed T2DM patients aged ≥20 y identified at Taiwanese hospital.
**Index model(s)**	XGBoost with grid-search hyperparameter tuning, SMOTE-Tomek for class imbalance, KNN imputation for missing data.
**Comparator model(s)**	LR, extra trees, RF, GBDT, LGBM.
**Outcome(s)**	Incident ESRD (eGFR <15 ml/min/1.73 m² or initiation of dialysis or kidney transplant) during follow-up.
**Timing**	Predictors at T2DM diagnosis (baseline), outcome ascertained over follow-up to Dec 2018 (median follow-up not stated, cohort assembled over 10 years).
**Setting and intended use of the prediction model**	Tertiary-care Taiwanese veterans hospital, intended as risk-assessment tool for early identification of T2DM patients at risk of ESRD to allow preventive interventions.

Step 2 – Classify the type of prediction model assessment

**Classify the assessment based on its aim**			
Type of prediction study	***Explanation***	***PROBAST+AI boxes to complete***	***Tick as appropriate***
**Development only**	**Prediction model development only, i.e., without evaluation of its performance.**	**Model development**	
**Evaluation only**	**External validation of one or more existing models in new data**	**Model evaluation**	
**Combination**	**Prediction model development combined in the same study (publication) with the evaluation of its apparent performance, internal validation performance, or external validation performance.**	**Model development and evaluation**	**X**

Step 3 - Assess quality and applicability or risk of bias and applicability

**Publication reference**	Ou SM, Tsai MT, Lee KH, Tseng WC, Yang CY, Chen TH, et al. Prediction of the risk of de-veloping end-stage renal diseases in newly diagnosed type 2 diabetes mellitus using arti-ficial intelligence algorithms. Biodata Mining. 2023;16(1).
**Models of interest**	XGBoost, LR, ET, RF, GBDT, LGBM.
**Outcome of interest**	Incident ESRD in newly-diagnosed T2DM patients.

Model development

**DOMAIN 1: Participants and data sources**	
**A. Quality**	
Final cohort 53,477 T2DM patients, 70/30 train/test split.	
	**Y/ PY/ PN/ N/ NI**
**1.1 Were appropriate data sources used?**	Y
**1.2 Was an appropriate study design used?**	Y
**1.3 Did the in- and exclusions of study participants result in a representative dataset?**	PY
**Concern regarding quality of selection of participants and data sources**	**QUALITYCONCERN:** ***low***/*high/unclear*
Rationale of quality rating: Very large EHR cohort spanning 10 years with explicit and clinically reasonable inclusion/exclusion criteria.	
**B. Applicability**	
Taiwanese veterans-hospital T2DM patients, predominantly older and male, high prevalence of cardiovascular comorbidities, ESRD incidence 8.9% over 10-year window.	
**Concern that the (data of the) included participants do not match the review question or the assessor's intended use of the prediction model**	**APPLICABILITY CONCERN:** ***low***/*high/unclear*
Rationale of applicability rating: Veterans hospital sample is older and more male than general T2DM populations, but the cohort spans full spectrum of newly-diagnosed T2DM with broad applicability to Taiwanese / East-Asian healthcare settings.	

**DOMAIN 2: Predictors**	
**A. Quality**	
78 features at T2DM diagnosis, 4 demographics, 22 ICD-9/10 comorbidities, 32 routine laboratory tests, and 20 concomitant medication classes, KNN imputation for missing values. Forward feature selection.	
	**Y/ PY/ PN/ N/ NI**
**2.1 Were predictors defined and assessed in a similar way for all participants?**	Y
**2.2 Was any pre-processing of predictors similar for all participants?**	Y
**2.3 Were predictor assessments made without knowledge of outcome data?**	Y
**2.4 Were the predictors included in the model available at the time the model was intended to be used?**	Y

**Concern regarding quality of selection of participants and data sources**	**QUALITYCONCERN:** ***low**/high/unclear*
Standardised EHR-derived predictors, KNN imputation applied uniformly, predictors available at point of decision.	
**B. Applicability**	
**Concern that the definition, pre-processing, assessment, or timing of assessment of the predictors in the model do not match the review question or the assessor's intended use**	**APPLICABILITY CONCERN:** ***low**/high/unclear*
Rationale of applicability rating: Routine EHR predictors broadly available, some features may not be measured at T2DM diagnosis in all settings.	

**DOMAIN 3: Outcome**	
**A. Quality**	
ESRD defined as eGFR <15 ml/min/1.73 m² (CKD-EPI) or receipt of maintenance dialysis or kidney transplant during follow-up, code-based ascertainment from EHR.	
Or	**Y/ PY/ PN/ N/ NI**
**3.1 Were outcomes defined and assessed appropriately?**	Y
**3.2 Were outcomes defined and assessed in a similar way for all participants?**	Y
**3.3 Were outcome assessments made without use or knowledge of predictor data?**	Y
**3.4 Was the time interval between predictor assessment and outcome assessment appropriate?**	Y
**Concern regarding quality of the outcome or its determination**	**QUALITYCONCERN:** ***low**/high/unclear*
Standard clinically-meaningful ESRD endpoint, uniformly applied.	
**B. Applicability**	
Over 10-year follow-up window from T2DM diagnosis, ESRD captured whenever occurred, composite of eGFR <15 OR maintenance dialysis OR transplant, distribution of contributing components not separately reported, eGFR-defined ESRD dominates over hard renal-replacement events in newly-diagnosed T2DM.	
**Concern that the outcome, its definition, assessment, or timing of assessment do not match the review question or the assessor's intended use**	**APPLICABILITY CONCERN:** ***low**/high/unclear*
ESRD is canonical endpoint for T2DM-CKD risk prediction, 10-year horizon clinically relevant for newly-diagnosed T2DM.	

**DOMAIN 4: Analysis**
**A. Quality**
37,433 training (3,334 ESRD events) and 16,044 testing (1,435 events),78 candidate predictors.
six ML models (LR, ET, RF, GBDT, XGBoost, LGBM), 5-fold CV on training set, SMOTE-Tomek balancing on training data only, forward feature selection, SHAP for interpretation.

AUC, accuracy, F1, precision, recall, average precision, log-loss, no calibration plot, no decision-curve analysis.
Missing data: K-nearest-neighbour imputation prior to modelling.

	**Y/ PY/ PN/ N/ NI**
**4.1 Was there evidence that the sample size was reasonable?**	Y
**4.2 Were continuous and categorical predictors handled appropriately?**	Y
**4.3 Were participants with missing or censored data handled appropriately in the analysis?**	PY
**4.4 If methods to address class imbalance were used, was the model or the model predictions recalibrated?***	PN
**4.5 Were methods used to address potential model overfitting?**	PY
**Concern regarding quality of the analysis**	**QUALITYCONCERN:** *low/**high**/unclear*
SMOTE-Tomek class balancing applied without subsequent recalibration of predicted probabilities, no calibration plot or DCA despite the model being framed as a clinical risk-assessment tool.	

Model evaluation

Domain 1,2 and 3 same analysis as model development

**DOMAIN 4: Analysis**
**Risk of bias**
test set 16,044 / 1,435 (8.9%).
AUC, accuracy, F1, precision, recall, average precision, log-loss, no calibration plot, no decision-curve analysis.
Describe any participants who were excluded from the analysis: Exclusion same as development
Describe missing data on predictors and outcomes as well as methods used for handling these missing data: Same as development

	**Y/ PY/ PN/ N/ NI/ NA**		
**4.1 Was model evaluation based on only apparent performance avoided?**	Y		
	**A**	**I**	**E**
**4.2 Was there evidence that the sample size was reasonable?**	NA	Y	NA
**4.3 Were participants with missing or censored data handled appropriately in the analysis?**	NA	PY	NA
**4.4 If methods to address class imbalance were used, was the evaluation done in a dataset without imbalance correction?***	NA	PN	NA
**4.5 If data splitting was done to create training and test datasets, was there evidence that data leakage was avoided?***	**NA**	PY	**NA**
**4.6 If resampling methods were used to evaluate model performance, were all model development steps replicated in the resampling process?***	**NA**	PY	**NA**
**4.7 Was the predictive performance of the model evaluated appropriately, e.g., calibration, discrimination, and net benefit?**	NA	PN	NA

Risk of bias introduced by the analysis	RISK OF BIAS: low/**high**/unclear
Reported AUROC but no external validation, no calibration plot or decision-curve analysis, reported precision/recall reflect SMOTE-balanced training, evaluated on imbalanced test data without adjustment.	

Step 4 - Assess the overall concerns regarding quality, risk of bias and applicability of the prediction model

OVERALL JUDGEMENT OF PREDICTION MODEL	
**Overall judgement of quality (development)**	**QUALITY CONCERN:** *low/**high**/unclear*
Large well-characterised 10-year cohort, SMOTE without recalibration and absent calibration assessment.	
**Overall judgement of risk of bias (evaluation)**	**RISK OF BIAS:** *low/**high**/unclear*
Internal hold-out only, SMOTE-Tomek probabilities not recalibrated, no calibration / DCA, no external validation.	
**Overall judgement of applicability (development)**	**APPLICABILITY CONCERN:** ***low**/high/unclear*
Veterans-hospital T2DM cohort is relevant but skewed male and elderly, broadly applicable to East-Asian T2DM populations.	
**Overall judgement of applicability (evaluation)**	**APPLICABILITY CONCERN:** ***low**/high/unclear*
Single Taiwanese veterans hospital, external validation in independent cohorts not performed.	

Chen et al. (69)

Step 1 - PICOTS

**Population**	2,056 biopsy-proven IgA nephropathy patients aged ≥18 from 18 kidney centres in China
**Index model(s)**	Interpretable multivariable LSTM (IMV-LSTM) with internal mixture attention.
**Comparator model(s)**	IMV-LSTM baseline (time-invariant only), IMV-LSTM time-variant only, Cox model (refitted KFRE), International IgA Nephropathy Risk Prediction Tool.
**Outcome(s)**	Composite kidney outcome: ≥50% reduction in eGFR or kidney failure (eGFR <15 ml/min/1.73 m^2 for ≥3 months, dialysis, or transplant), assessed within 5 years of the third visit in each window.
**Timing**	Predictors from three consecutive follow-up visits, outcome predicted within 5 years following the third visit.
**Setting and intended use of the prediction model**	Tertiary nephrology centres in China, intended to provide dynamic individualised risk prediction during routine follow-up and inform treatment decisions.

Step 2 – Classify the type of prediction model assessment

Classify the assessment based on its aim			
Type of prediction study	***Explanation***	***PROBAST+AI boxes to complete***	***Tick as appropriate***
**Development only**	**Prediction model development only, i.e., without evaluation of its performance.**	**Model development**	
**Evaluation only**	**External validation of one or more existing models in new data**	**Model evaluation**	
**Combination**	**Prediction model development combined in the same study (publication) with the evaluation of its apparent performance, internal validation performance, or external validation performance.**	**Model development and evaluation**	**X**

Step 3 - Assess quality and applicability or risk of bias and applicability

**Publication reference**	Chen T, Chen T, Xu W, Liang S, Xu F, Liang D, et al. Development and External Validation of a Multidimensional Deep Learning Model to Dynamically Predict Kidney Outcomes in IgA Nephropathy. Clinical Journal of the American Society of Nephrology. 2024;19(7):898–907.
**Models of interest**	Interpretable Multivariable LSTM (IMV-LSTM) full / baseline / time-variant models.
**Outcome of interest**	Composite: 50% eGFR decline or kidney failure within 5 years of the prediction window.

Model development

DOMAIN 1: Participants and data sources	
**A. Quality**	
1,031 patients of 3 consecutive visits (randomised 80/20 train/validation), test: 1,025 patients from 17 additional centres, median follow-up 5.5 y.	
	**Y/ PY/ PN/ N/ NI**
**1.1 Were appropriate data sources used?**	Y
**1.2 Was an appropriate study design used?**	Y
**1.3 Did the in- and exclusions of study participants result in a representative dataset?**	Y
**Concern regarding quality of selection of participants and data sources**	**QUALITYCONCERN:** ***low****/high/unclear*

Rationale of quality rating: Large multicentre biopsy-proven IgAN cohort with explicit inclusion criteria and substantial follow-up.	
**B. Applicability**	
Multicentre Chinese IgAN cohort spanning, baseline eGFR ≥30, proteinuria ≥0.5 g/d, mean age 35y, 51% female, mean eGFR 96.9, mean proteinuria 1.1 g/d, cohort representative of routine IgAN follow-up practice.	
**Concern that the (data of the) included participants do not match the review question or the assessor's intended use of the prediction model**	**APPLICABILITY CONCERN:** ***low****/high/unclear*
Rationale of applicability rating: Multicentre Chinese biopsy-proven IgAN cohort matches intended use for dynamic risk prediction during routine follow-up, ethnic generalisability beyond Asian populations not confirmed.	

**DOMAIN 2: Predictors**	
**A. Quality**	
5 time-invariant variables and 8 time-variant variables repeatedly measured at follow-up, baseline missing values imputed by chained equations, follow-up missing values by forward filling.	
	**Y/ PY/ PN/ N/ NI**
**2.1 Were predictors defined and assessed in a similar way for all participants?**	Y
**2.2 Was any pre-processing of predictors similar for all participants?**	Y
**2.3 Were predictor assessments made without knowledge of outcome data?**	Y
**2.4 Were the predictors included in the model available at the time the model was intended to be used?**	Y
**Concern regarding quality of selection of participants and data sources**	**QUALITYCONCERN:** ***low****/high/unclear*
Routine clinical and pathological predictors with standard ascertainment, explicit imputation strategy, predictors collected before outcome assessment.	
**B. Applicability**	
**Concern that the definition, pre-processing, assessment, or timing of assessment of the predictors in the model do not match the review question or the assessor's intended use**	**APPLICABILITY CONCERN:** ***low****/high/unclear*
Rationale of applicability rating: Predictors routinely measured during IgAN follow-up, biopsy-derived Oxford scores are standard in IgAN clinical practice.	

**DOMAIN 3: Outcome**	
**A. Quality**	
composite of ≥50% eGFR decline or kidney failure within 5 years following the third visit of each sliding window, eGFR by CKD-EPI.	
Or	**Y/ PY/ PN/ N/ NI**
**3.1 Were outcomes defined and assessed appropriately?**	Y
**3.2 Were outcomes defined and assessed in a similar way for all participants?**	Y
**3.3 Were outcome assessments made without use or knowledge of predictor data?**	Y

**3.4 Was the time interval between predictor assessment and outcome assessment appropriate?**	Y
**Concern regarding quality of the outcome or its determination**	**QUALITYCONCERN:** ***low****/high/unclear*
Objective, validated composite endpoint matching KDIGO surrogate / hard endpoints, uniform CKD-EPI assessment.	
**B. Applicability**	
Within 5 years of the third visit of each sliding windo, composite of ≥50% eGFR decline and kidney failure, exact split of components not separately in the publication.	
**Concern that the outcome, its definition, assessment, or timing of assessment do not match the review question or the assessor's intended use**	**APPLICABILITY CONCERN:** ***low****/high/unclear*
Composite of validated surrogate (50% eGFR decline) and hard kidney-failure endpoint matches KDIGO-endorsed CKD-progression endpoints, 5-year horizon clinically meaningful for IgAN risk stratification.	

**DOMAIN 4: Analysis**	
**A. Quality**	
12,369 training and 3,093 validation windows ,13 predictors, events not separately reported per window.	
IMV-LSTM window length of 3 visits, comparison with baseline-only and time-variant-only LSTM, latent class mixed models for trajectory analysis, Cox proportional hazards for HRs across trajectory groups.	
C-statistic, calibration plot, Hosmer–Lemeshow test, net reclassification improvement (NRI), Kaplan–Meier across risk subgroups, secondary endpoints.	
Missing data: baseline imputed by chained equations; follow-up by forward-filling; missing rates explicitly tabulated	
	**Y/ PY/ PN/ N/ NI**
**4.1 Was there evidence that the sample size was reasonable?**	Y
**4.2 Were continuous and categorical predictors handled appropriately?**	Y
**4.3 Were participants with missing or censored data handled appropriately in the analysis?**	Y
**4.4 If methods to address class imbalance were used, was the model or the model predictions recalibrated?***	NA
**4.5 Were methods used to address potential model overfitting?**	Y
**Concern regarding quality of the analysis**	**QUALITYCONCERN:** ***low****/high/unclear*
Large training/validation set, explicit missing-data handling, calibration and NRI assessed, appropriate ranking loss for censored data, interpretable mixture-attention architecture.	

Model evaluation

Domain 1,2 and 3 same analysis as model development

**DOMAIN 4: Analysis**
**Risk of bias**
external test 8,797 windows from 1,025 patients in 17 centres, per-window event counts not separately reported.

C-statistic, calibration plot, Hosmer–Lemeshow test, net reclassification improvement (NRI), Kaplan–Meier across risk subgroups, secondary endpoints.			
Describe any participants who were excluded from the analysis: Exclusion same as development			
Describe missing data on predictors and outcomes as well as methods used for handling these missing data: Same as development			
	**Y/ PY/ PN/ N/ NI/ NA**		
**4.1 Was model evaluation based on only apparent performance avoided?**	Y		
	**A**	**I**	**E**
**4.2 Was there evidence that the sample size was reasonable?**	NA	Y	Y
**4.3 Were participants with missing or censored data handled appropriately in the analysis?**	NA	Y	Y
**4.4 If methods to address class imbalance were used, was the evaluation done in a dataset without imbalance correction?***	NA	NA	NA
**4.5 If data splitting was done to create training and test datasets, was there evidence that data leakage was avoided?***	**NA**	Y	**NA**
**4.6 If resampling methods were used to evaluate model performance, were all model development steps replicated in the resampling process?***	**NA**	Y	**NA**
**4.7 Was the predictive performance of the model evaluated appropriately, e.g., calibration, discrimination, and net benefit?**	NA	Y	Y
**Risk of bias introduced by the analysis**	**RISK OF BIAS:** ***low****/high/unclear*		
Robust external multicentre validation, calibration plots, Hosmer–Lemeshow test, NRI, and trajectory analyses all performed.			

Step 4 - Assess the overall concerns regarding quality, risk of bias and applicability of the prediction model

**OVERALL JUDGEMENT OF PREDICTION MODEL**	
**Overall judgement of quality (development)**	**QUALITY CONCERN:** ***low****/high/unclear*
Large multicentre IgAN cohort, longitudinal LSTM model with appropriate handling of censoring, missingness, calibration, and interpretation.	
**Overall judgement of risk of bias (evaluation)**	**RISK OF BIAS:** ***low****/high/unclear*
Independent external multicentre validation in 17 centres, comprehensive evaluation including calibration, NRI, and subgroup analyses.	
**Overall judgement of applicability (development)**	**APPLICABILITY CONCERN:** ***low****/high/unclear*
Multicentre Chinese biopsy-proven IgAN cohort closely matches intended-use population.	
**Overall judgement of applicability (evaluation)**	**APPLICABILITY CONCERN:** ***low****/high/unclear*
17-centre external Chinese cohort confirms applicability across IgAN nephrology practice in China.	

Chang et al. (70)

Step 1 - PICOTS

**Population**	1,965 advanced CKD outpatients aged ≥20 years from Chinese hospital.
**Index model(s)**	XGBoost binary classifier, KNN imputation of missing values.
**Comparator model(s)**	Multivariable logistic regression, two practicing nephrologists in a head-to-head human–machine competition.
**Outcome(s)**	Hyperkalemia (K+ >5.5 mEq/L) at the next clinic visit.
**Timing**	Predictors at clinic visit t, outcome at next visit t+1.
**Setting and intended use of the prediction model**	Single-centre outpatient nephrology program in Taiwan, intended as a clinical decision-support alert tool for the CKD team.

Step 2 – Classify the type of prediction model assessment

**Classify the assessment based on its aim**			
Type of prediction study	***Explanation***	***PROBAST+AI boxes to complete***	***Tick as appropriate***
**Development only**	**Prediction model development only, i.e., without evaluation of its performance.**	**Model development**	
**Evaluation only**	**External validation of one or more existing models in new data**	**Model evaluation**	
**Combination**	**Prediction model development combined in the same study (publication) with the evaluation of its apparent performance, internal validation performance, or external validation performance.**	**Model development and evaluation**	**X**

Step 3 - Assess quality and applicability or risk of bias and applicability

**Publication reference**	Chang HH, Chiang JH, Tsai CC, Chiu PF. Predicting hyperkalemia in patients with ad-vanced chronic kidney disease using the XGBoost model. BMC Nephrol. 2023;24(1).
**Models of interest**	XGBoost, comparator logistic regression, two nephrologists.
**Outcome of interest**	Hyperkalemia (K+ >5.5 mEq/L) at next clinic visit.

Model development

DOMAIN 1: Participants and data sources	
**A. Quality**	
1,965 patients, inclusion: ≥20 y, eGFR <30, ≥2 outpatient visits within 3 months.	
	**Y/ PY/ PN/ N/ NI**
**1.1 Were appropriate data sources used?**	PY
**1.2 Was an appropriate study design used?**	PY
**1.3 Did the in- and exclusions of study participants result in a representative dataset?**	PY
**Concern regarding quality of selection of participants and data sources**	**QUALITYCONCERN:** *low/**high**/unclear*
Rationale of quality rating: Single-centre retrospective cohort, baseline characteristics significantly differ between training and testing sets, non-random temporal split despite the stated random allocation.	
**B. Applicability**	
Single Taiwanese tertiary-care pre-ESRD program, mean age 69.4 y, 49% female, advanced CKD only.	
**Concern that the (data of the) included participants do not match the review question or the assessor's intended use of the prediction model**	**APPLICABILITY CONCERN:** *low/**high**/unclear*
Rationale of applicability rating: Single-centre Taiwanese pre-ESRD-program cohort with country-specific care pathways limits transportability.	

DOMAIN 2: Predictors	
**A. Quality**	
Demographics, 9 laboratory variables, 7 comorbidities by ICD-9/10 and 16 medication classes, missing laboratory values imputed via K-nearest neighbours separately in train/test.	
	**Y/ PY/ PN/ N/ NI**
**2.1 Were predictors defined and assessed in a similar way for all participants?**	Y
**2.2 Was any pre-processing of predictors similar for all participants?**	Y
**2.3 Were predictor assessments made without knowledge of outcome data?**	PY
**2.4 Were the predictors included in the model available at the time the model was intended to be used?**	Y
**Concern regarding quality of selection of participants and data sources**	**QUALITYCONCERN:** ***low**/high/unclear*
Routine, standardised lab and prescription data uniformly captured in the pre-ESRD registry, predictors available at the index visit.	
**B. Applicability**	
**Concern that the definition, pre-processing, assessment, or timing of assessment of the predictors in the model do not match the review question or the assessor's intended use**	**APPLICABILITY CONCERN:** ***low**/high/unclear*

Rationale of applicability rating: All predictors are routine laboratory/clinical data widely available in any nephrology outpatient setting.

**DOMAIN 3: Outcome**	
**A. Quality**	
Hyperkalemia defined as serum potassium >5.5 mEq/L at the next scheduled clinic visit, measurement performed in a College-of-American-Pathologists-accredited laboratory.	
Or	**Y/ PY/ PN/ N/ NI**
**3.1 Were outcomes defined and assessed appropriately?**	Y
**3.2 Were outcomes defined and assessed in a similar way for all participants?**	Y
**3.3 Were outcome assessments made without use or knowledge of predictor data?**	Y
**3.4 Was the time interval between predictor assessment and outcome assessment appropriate?**	Y
**Concern regarding quality of the outcome or its determination**	**QUALITYCONCERN:** ***low**/high/unclear*
Objective laboratory-measured outcome, standard clinical threshold, uniform ascertainment.	
**B. Applicability**	
Next outpatient visit (typically 3 months after index), single binary outcome (K+ >5.5 mEq/L), not a composite.	
**Concern that the outcome, its definition, assessment, or timing of assessment do not match the review question or the assessor's intended use**	**APPLICABILITY CONCERN:** ***low**/high/unclear*
K+ >5.5 mEq/L is a clinically actionable hyperkalemia threshold, next-visit prediction horizon matches intended decision-support use.	

**DOMAIN 4: Analysis**	
**A. Quality**	
6,949 training visits with 460 hyperkalemia events and 2,054 test visits with 141 events.	
XGBoost binary classifier, logistic regression as comparator, decision-curve analysis (DCA) and clinical impact curve (CIC), SHAP for feature importance, DeLong test for AUC comparison.	
AUROC, sensitivity, specificity, PPV, NPV, accuracy, DCA and CIC reported, no calibration plot.	
Missing data: K-nearest-neighbours imputation applied separately to training and testing sets.	
	**Y/ PY/ PN/ N/ NI**
**4.1 Was there evidence that the sample size was reasonable?**	PY
**4.2 Were continuous and categorical predictors handled appropriately?**	Y
**4.3 Were participants with missing or censored data handled appropriately in the analysis?**	PY

**4.4 If methods to address class imbalance were used, was the model or the model predictions recalibrated?***	NA
**4.5 Were methods used to address potential model overfitting?**	Y
**Concern regarding quality of the analysis**	**QUALITYCONCERN:** *low/**high**/unclear*
Severe class imbalance yields very low sensitivity at default threshold, no calibration plot, class imbalance not formally addressed.	

Model evaluation

Domain 1,2 and 3 same analysis as model development

**DOMAIN 4: Analysis**			
**Risk of bias**			
2,054 test visits / 141 hyperkalemia events.			
AUROC, sensitivity, specificity, PPV, NPV, accuracy, DCA and CIC reported, no calibration plot.			
Describe any participants who were excluded from the analysis: Exclusion same as development			
Describe missing data on predictors and outcomes as well as methods used for handling these missing data: Same as development			
	**Y/ PY/ PN/ N/ NI/ NA**		
**4.1 Was model evaluation based on only apparent performance avoided?**	Y		
	**A**	**I**	**E**
**4.2 Was there evidence that the sample size was reasonable?**	NA	PY	NA
**4.3 Were participants with missing or censored data handled appropriately in the analysis?**	NA	PY	NA
**4.4 If methods to address class imbalance were used, was the evaluation done in a dataset without imbalance correction?***	NA	NA	NA
**4.5 If data splitting was done to create training and test datasets, was there evidence that data leakage was avoided?***	**NA**	PY	**NA**
**4.6 If resampling methods were used to evaluate model performance, were all model development steps replicated in the resampling process?***	**NA**	PY	**NA**
**4.7 Was the predictive performance of the model evaluated appropriately, e.g., calibration, discrimination, and net benefit?**	NA	PN	NA
**Risk of bias introduced by the analysis**	**RISK OF BIAS:** *low/**high**/unclear*		
No external validation, no calibration plot despite model being intended for probability-based clinical alerts, very low sensitivity at the operating point chosen.			

Step 4 - Assess the overall concerns regarding quality, risk of bias and applicability of the prediction model

**OVERALL JUDGEMENT OF PREDICTION MODEL**	
**Overall judgement of quality (development)**	**QUALITY CONCERN:** *low/**high**/unclear*

Single-centre Taiwanese cohort with between-set baseline imbalance, no calibration, low sensitivity at chosen threshold.	
**Overall judgement of risk of bias (evaluation)**	**RISK OF BIAS:**<br>*low/**high**/unclear*
Internal hold-out only, no calibration assessment, no external validation.	
**Overall judgement of applicability (development)**	**APPLICABILITY CONCERN:**<br>*low/**high**/unclear*
Country-specific pre-ESRD program, advanced-CKD only, transportability uncertain.	
**Overall judgement of applicability (evaluation)**	**APPLICABILITY CONCERN:**<br>*low/**high**/unclear*
Single Taiwanese centre limits external applicability.	

Lu et al. (71)

Step 1 - PICOTS

**Population**	1,092 adults aged ≥45 y with self-reported physician-diagnosed CKD from the China Health and Retirement Longitudinal Study (CHARLS) 2015 wave.
**Index model(s)**	Logistic-regression nomogram with 6 predictors selected by LASSO, Bayesian-optimised gradient boosting machine (GBM) as best ML model.
**Comparator model(s)**	Regularized discriminant analysis (RDA), RF, SVM, KDIGO/AWGS-defined sarcopenia status as outcome.
**Outcome(s)**	Sarcopenia (AWGS 2019)
**Timing**	Cross-sectional
**Setting and intended use of the prediction model**	Chinese community-dwelling middle-aged and older adults with self-reported CKD, intended for primary-care sarcopenia risk stratification.

Step 2 – Classify the type of prediction model assessment

**Classify the assessment based on its aim**			
Type of prediction study	***Explanation***	***PROBAST+AI boxes to complete***	***Tick as appropriate***
**Development only**	**Prediction model development only, i.e., without evaluation of its performance.**	**Model development**	
**Evaluation only**	**External validation of one or more existing models in new data**	**Model evaluation**	

**Combination**	**Prediction model development combined in the same study (publication) with the evaluation of its apparent performance, internal validation performance, or external validation performance.**	**Model development and evaluation**	**X**

Step 3 - Assess quality and applicability or risk of bias and applicability

**Publication reference**	Lu R, Wang S, Chen P, Li F, Li P, Chen Q, et al. Predictive model for sarcopenia in chronic kidney disease: a nomogram and machine learning approach using CHARLS data. Front Med (Lausanne). 2025;12:1546988.
**Models of interest**	Nomogram (logistic regression) + Bayesian-optimised GBM.
**Outcome of interest**	Sarcopenia (AWGS 2019 criteria with anthropometric-equation-estimated ASM) in adults ≥45 y with self-reported CKD.

Model development

**DOMAIN 1: Participants and data sources**	
**A. Quality**	
1,092 CKD patients selected from 21,095 CHARLS 2015 participants, 70/30 train/test split, 231 with sarcopenia, mean age 65.4y, 50.5% female.	
	**Y/ PY/ PN/ N/ NI**
**1.1 Were appropriate data sources used?**	PY
**1.2 Was an appropriate study design used?**	PY
**1.3 Did the in- and exclusions of study participants result in a representative dataset?**	PY
**Concern regarding quality of selection of participants and data sources**	**QUALITYCONCERN:** *low/**high**/unclear*
Rationale of quality rating: CKD ascertainment by self-report only, substantial exclusion of 827 patients with missing key variables, cross-sectional design limits causal inference, modest sample size after exclusions.	
**B. Applicability**	
Chinese community-dwelling adults ≥45 y from CHARLS 2015 wave, self-reported CKD only (no eGFR-based confirmation in the cohort), diverse comorbidity profile.	
**Concern that the (data of the) included participants do not match the review question or the assessor's intended use of the prediction model**	**APPLICABILITY CONCERN:** *low/**high**/unclear*
Rationale of applicability rating: Self-reported CKD and Chinese community-dwelling middle-aged/older population limit applicability to biochemically-confirmed CKD populations and to non-Chinese settings, rural-majority sample further restricts generalisability.	

**DOMAIN 2: Predictors**
**A. Quality**

From 49 candidate variables, LASSO selected 6 for the nomogram: age, waist circumference, LDL-C, HDL-C, triglycerides, diastolic blood pressure.	
	**Y/ PY/ PN/ N/ NI**
**2.1 Were predictors defined and assessed in a similar way for all participants?**	Y
**2.2 Was any pre-processing of predictors similar for all participants?**	Y
**2.3 Were predictor assessments made without knowledge of outcome data?**	Y
**2.4 Were the predictors included in the model available at the time the model was intended to be used?**	Y
**Concern regarding quality of selection of participants and data sources**	**QUALITYCONCERN:** ***low****/high/unclear*
Standard demographic, anthropometric and lipid predictors uniformly assessed in CHARLS, LASSO performed on training set only.	
**B. Applicability**	
**Concern that the definition, pre-processing, assessment, or timing of assessment of the predictors in the model do not match the review question or the assessor's intended use**	**APPLICABILITY CONCERN:** ***low****/high/unclear*
Rationale of applicability rating: All six final predictors (age, waist circumference, LDL-C, HDL-C, triglycerides, DBP) are routinely measured in community / primary-care settings.	

**DOMAIN 3: Outcome**	
**A. Quality**	
Sarcopenia per AWGS 2019.	
Or	**Y/ PY/ PN/ N/ NI**
**3.1 Were outcomes defined and assessed appropriately?**	PN
**3.2 Were outcomes defined and assessed in a similar way for all participants?**	Y
**3.3 Were outcome assessments made without use or knowledge of predictor data?**	Y
**3.4 Was the time interval between predictor assessment and outcome assessment appropriate?**	Y
**Concern regarding quality of the outcome or its determination**	**QUALITYCONCERN:** *low/****high****/unclear*
ASM estimated by anthropometric equation rather than DXA / BIA, the AWGS gold standards.	
**B. Applicability**	
Cross-sectional, composite operational definition of sarcopenia requires low ASM AND low grip strength OR low physical performance, distribution of contributing combinations not separately reported.	
**Concern that the outcome, its definition, assessment, or timing of assessment do not match the review question or the assessor's intended use**	**APPLICABILITY CONCERN:** *low/****high****/unclear*
Anthropometric-equation-based ASM and percentile-derived cutoffs deviate from the AWGS DXA/BIA reference and absolute thresholds, limiting comparability with standard sarcopenia diagnoses in clinical or research settings.	

DOMAIN 4: Analysis	
A. Quality	
Training n=764, test n=328, 49 candidate predictors, 6 final predictors	
LASSO for predictor selection, logistic-regression nomogram, four ML models (GBM, RDA, RF, SVM).	
AUROC, calibration plots, Hosmer–Lemeshow test, decision-curve analysis, AUC nomogram	
Missing data: 827 patients with missing key variables excluded (complete-case analysis), no imputation reported.	
	**Y/ PY/ PN/ N/ NI**
**4.1 Was there evidence that the sample size was reasonable?**	PN
**4.2 Were continuous and categorical predictors handled appropriately?**	Y
**4.3 Were participants with missing or censored data handled appropriately in the analysis?**	PN
**4.4 If methods to address class imbalance were used, was the model or the model predictions recalibrated?***	NA
**4.5 Were methods used to address potential model overfitting?**	Y
**Concern regarding quality of the analysis**	**QUALITYCONCERN:** *low/**high**/unclear*
Modest sample size, complete-case analysis excludes 827 patients without imputation, potential bias from missing-not-at-random data, no external validation.	

Model evaluation

Domain 1,2 and 3 same analysis as model development

DOMAIN 4: Analysis			
Risk of bias			
internal test n=328 / 69 events.			
LASSO for predictor selection, logistic-regression nomogram, four ML models (GBM, RDA, RF, SVM).			
Describe any participants who were excluded from the analysis: Exclusion same as development			
Describe missing data on predictors and outcomes as well as methods used for handling these missing data: Same as development			
	**Y/ PY/ PN/ N/ NI/ NA**		
**4.1 Was model evaluation based on only apparent performance avoided?**	Y		
	**A**	**I**	**E**
**4.2 Was there evidence that the sample size was reasonable?**	NA	PN	NA
**4.3 Were participants with missing or censored data handled appropriately in the analysis?**	NA	PN	NA
**4.4 If methods to address class imbalance were used, was the evaluation done in**	NA	NA	NA

**a dataset without imbalance correction?***			
**4.5 If data splitting was done to create training and test datasets, was there evidence that data leakage was avoided?***	**NA**	PY	**NA**
**4.6 If resampling methods were used to evaluate model performance, were all model development steps replicated in the resampling process?***	**NA**	PY	**NA**
**4.7 Was the predictive performance of the model evaluated appropriately, e.g., calibration, discrimination, and net benefit?**	NA	PY	NA
**Risk of bias introduced by the analysis**	**RISK OF BIAS:** *low/**high**/unclear*		
Internal hold-out only, modest test-set size with few events, RF train AUC 1.000 suggests overfitting risk, no external validation, complete-case analysis without imputation.			

Step 4 - Assess the overall concerns regarding quality, risk of bias and applicability of the prediction model

**OVERALL JUDGEMENT OF PREDICTION MODEL**	
**Overall judgement of quality (development)**	**QUALITY CONCERN:** *low/**high**/unclear*
Self-reported CKD ascertainment, anthropometric-equation surrogate sarcopenia outcome, complete-case exclusions, modest sample, no external validation.	
**Overall judgement of risk of bias (evaluation)**	**RISK OF BIAS:** *low/**high**/unclear*
Internal hold-out only, small validation event count, RF overfitting signal, no external validation.	
**Overall judgement of applicability (development)**	**APPLICABILITY CONCERN:** *low/**high**/unclear*
Self-reported CKD and percentile-derived ASM cutoffs limit applicability to biochemically-confirmed CKD populations and to standard AWGS-defined sarcopenia.	
**Overall judgement of applicability (evaluation)**	**APPLICABILITY CONCERN:** *low/**high**/unclear*
CHARLS Chinese community sample only, external generalisability unproven.	

Hsu et al. (72)

Step 1 - PICOTS

**Population**	6,614 adult CKD stage 3–5 and ESKD patients at Taiwanese hospital.
**Index model(s)**	Artificial neural network
**Comparator model(s)**	LR, XGBoost, LightGBM, CatBoost, SVM, DT, RF, KNN.
**Outcome(s)**	Osteoporosis defined as DXA T-score ≤−2.5 at lumbar spine, femoral neck, or total hip.
**Timing**	Cross-sectional
**Setting and intended use of the prediction model**	Single tertiary Taiwanese veterans hospital with CKD-MBD specialty clinic, intended to screen CKD/ESKD patients for high osteoporosis risk.

Step 2 – Classify the type of prediction model assessment

**Classify the assessment based on its aim**			
Type of prediction study	***Explanation***	***PROBAST+AI boxes to complete***	***Tick as appropriate***
**Development only**	**Prediction model development only, i.e., without evaluation of its performance.**	**Model development**	
**Evaluation only**	**External validation of one or more existing models in new data**	**Model evaluation**	
**Combination**	**Prediction model development combined in the same study (publication) with the evaluation of its apparent performance, internal validation performance, or external validation performance.**	**Model development and evaluation**	**X**

Step 3 - Assess quality and applicability or risk of bias and applicability

**Publication reference**	Hsu CT, Huang CY, Chen CH, Deng YL, Lin SY, Wu MJ. Machine learning models to pre-dict osteoporosis in patients with chronic kidney disease stage 3-5 and end-stage kidney disease. Sci Rep. 2025;15(1):11391.
**Models of interest**	Artificial neural network, 8 comparator ML models.
**Outcome of interest**	Osteoporosis (DXA T-score ≤−2.5) in CKD stage 3–5 and ESKD patients.

Model development

**DOMAIN 1: Participants and data sources**	
**A. Quality**	
6,614 CKD stage 3–5 / ESKD adults selected from 35,670 osteoporosis-database DXA scans at single Taiwanese tertiary hospital. Osteoporosis prevalence 68.4%, control 31.6%, stratified random 80/20 train/test split, train further split 80/20 to train/validation, 5-fold CV during model selection.	
	**Y/ PY/ PN/ N/ NI**
**1.1 Were appropriate data sources used?**	Y
**1.2 Was an appropriate study design used?**	PY
**1.3 Did the in- and exclusions of study participants result in a representative dataset?**	PN
**Concern regarding quality of selection of participants and data sources**	**QUALITYCONCERN:** *low/**high**/unclear*
Rationale of quality rating: Single-centre cohort with substantial, osteoporosis prevalence of 68.4% is much higher than typical population, reflecting referral bias.	

B. Applicability	
Single Taiwanese tertiary veterans hospital CKD-MBD clinic cohort, mean age 75.3 y, 70% female, advanced CKD stage 3–5 / ESKD, 51% with previous fracture history.	
**Concern that the (data of the) included participants do not match the review question or the assessor's intended use of the prediction model**	**APPLICABILITY CONCERN:** *low/**high**/unclear*
Rationale of applicability rating: Single-centre Taiwanese veterans hospital DXA-referred cohort with very high osteoporosis prevalence, transportability to general CKD outpatient populations limited, population skewed elderly, female, and high-risk.	

DOMAIN 2: Predictors	
**A. Quality**	
16 predictors after recursive feature elimination with cross-validation, finally 8 model features.	
	**Y/ PY/ PN/ N/ NI**
**2.1 Were predictors defined and assessed in a similar way for all participants?**	Y
**2.2 Was any pre-processing of predictors similar for all participants?**	Y
**2.3 Were predictor assessments made without knowledge of outcome data?**	Y
**2.4 Were the predictors included in the model available at the time the model was intended to be used?**	Y
**Concern regarding quality of selection of participants and data sources**	**QUALITYCONCERN:** ***low**/high/unclear*
Routine clinical and biochemical predictors, multiple imputation applied uniformly, recursive feature elimination performed in CV.	
**B. Applicability**	
**Concern that the definition, pre-processing, assessment, or timing of assessment of the predictors in the model do not match the review question or the assessor's intended use**	**APPLICABILITY CONCERN:** ***low**/high/unclear*
Rationale of applicability rating: All final predictors (age, i-PTH, weight, height, hemoglobin, glucose, creatinine, albumin) are routinely measured in CKD-MBD clinical practice worldwide.	

DOMAIN 3: Outcome	
**A. Quality**	
Osteoporosis defined as DXA T-score ≤−2.5 at lumbar spine, femoral neck, or total hip; DXA scans performed at the same tertiary centre on standardised equipment.	
Or	**Y/ PY/ PN/ N/ NI**
**3.1 Were outcomes defined and assessed appropriately?**	Y
**3.2 Were outcomes defined and assessed in a similar way for all participants?**	Y
**3.3 Were outcome assessments made without use or knowledge of predictor data?**	Y
**3.4 Was the time interval between predictor assessment and outcome assessment appropriate?**	Y

Concern regarding quality of the outcome or its determination	QUALITYCONCERN: ***low***/*high/unclear*
Gold-standard WHO-defined DXA T-score outcome uniformly applied.	
**B. Applicability**	
Cross-sectional, osteoporosis status assessed at a single time point, single binary outcome, not a composite in time-to-event sense.	
**Concern that the outcome, its definition, assessment, or timing of assessment do not match the review question or the assessor's intended use**	**APPLICABILITY CONCERN:** ***low***/*high/unclear*
WHO operational definition matches the intended use as a DXA-screening triage tool, lumbar/femoral/hip sites are all standard DXA measurement locations.	

**DOMAIN 4: Analysis**	
**A. Quality**	
5,291 training (80%) and 1,323 test (20%), osteoporosis events: 4,526 overall; 8 final predictors	
9 ML models (LR, XGBoost, LightGBM, CatBoost, SVM, DT, RF, KNN, ANN) with 5-fold CV	
AUROC, accuracy, precision, recall, F1, calibration curve, decision-curve analysis, ROC, confusion matrix, predictive-probability histograms, SHAP for feature importance, Kaplan–Meier curves for eGFR, albumin, tumor size.	
Missing data: multiple imputation by chained equations (sensitivity analyses with mean, KNN, iterative imputation, and complete-case all reported similar performance).	
	**Y/ PY/ PN/ N/ NI**
**4.1 Was there evidence that the sample size was reasonable?**	Y
**4.2 Were continuous and categorical predictors handled appropriately?**	Y
**4.3 Were participants with missing or censored data handled appropriately in the analysis?**	Y
**4.4 If methods to address class imbalance were used, was the model or the model predictions recalibrated?***	NA
**4.5 Were methods used to address potential model overfitting?**	Y
**Concern regarding quality of the analysis**	**QUALITYCONCERN:** ***low***/*high/unclear*
Large sample with many events per variable, multiple-imputation sensitivity analyses confirm robustness, calibration curve and DCA reported.	

Model evaluation

Domain 1,2 and 3 same analysis as model development

**DOMAIN 4: Analysis**
**Risk of bias**
Test 1,323 / ~905 osteoporosis events (68.4% prevalence).

AUROC, accuracy, precision, recall, F1, calibration curve, decision-curve analysis, ROC, confusion matrix, predictive-probability histograms, SHAP for feature importance, Kaplan–Meier curves for eGFR, albumin, tumor size.			
Describe any participants who were excluded from the analysis:<br>Exclusion same as development			
Describe missing data on predictors and outcomes as well as methods used for handling these missing data:<br>Same as development			
	**Y/ PY/ PN/ N/ NI/ NA**		
**4.1 Was model evaluation based on only apparent performance avoided?**	Y		
	**A**	**I**	**E**
**4.2 Was there evidence that the sample size was reasonable?**	NA	Y	NA
**4.3 Were participants with missing or censored data handled appropriately in the analysis?**	NA	Y	NA
**4.4 If methods to address class imbalance were used, was the evaluation done in a dataset without imbalance correction?***	NA	NA	NA
**4.5 If data splitting was done to create training and test datasets, was there evidence that data leakage was avoided?***	**NA**	Y	**NA**
**4.6 If resampling methods were used to evaluate model performance, were all model development steps replicated in the resampling process?***	**NA**	Y	**NA**
**4.7 Was the predictive performance of the model evaluated appropriately, e.g., calibration, discrimination, and net benefit?**	NA	Y	NA
**Risk of bias introduced by the analysis**	**RISK OF BIAS:**<br>*low/**high**/unclear*		
Comprehensive internal evaluation, absence of external validation in a cohort, high event prevalence in test set may inflate apparent performance.			

Step 4 - Assess the overall concerns regarding quality, risk of bias and applicability of the prediction model

**OVERALL JUDGEMENT OF PREDICTION MODEL**	
**Overall judgement of quality (development)**	**QUALITY CONCERN:**<br>*low/**high**/unclear*
Single-centre Taiwanese DXA-referred cohort with 68.4% osteoporosis prevalence reflects strong referral selection, otherwise rigorous analysis with calibration and DCA.	
**Overall judgement of risk of bias (evaluation)**	**RISK OF BIAS:**<br>*low/**high**/unclear*
Internal hold-out only, no external validation, high event prevalence may inflate AUROC.	
**Overall judgement of applicability (development)**	**APPLICABILITY CONCERN:**<br>*low/**high**/unclear*
Taiwanese DXA-referral cohort restricted by national coverage criteria limits applicability to general CKD populations.	
**Overall judgement of applicability (evaluation)**	**APPLICABILITY CONCERN:**<br>*low/**high**/unclear*
Single Taiwanese DXA-referral cohort, generalisability untested.	

Oh et al. (73)

Step 1 - PICOTS

**Population**	Korean adults with RCC undergoing partial or radical nephrectomy.
**Index model(s)**	Gradient Boosting classifier on 12 perioperative variables.
**Comparator model(s)**	SVM, LR, DT, KNN, RF, AdaBoost, XGBoost and LightGBM
**Outcome(s)**	Post-operative CKD defined as post-nephrectomy eGFR <60 mL/min/1.73 m² (CKD-EPI).
**Timing**	Cross-sectional
**Setting and intended use of the prediction model**	Korean tertiary unephrology practice, intended as a postoperative-follow-up risk-stratification tool to identify RCC nephrectomy patients at elevated risk of CKD.

Step 2 – Classify the type of prediction model assessment

**Classify the assessment based on its aim**			
Type of prediction study	***Explanation***	***PROBAST+AI boxes to complete***	***Tick as appropriate***
**Development only**	**Prediction model development only, i.e., without evaluation of its performance.**	**Model development**	
**Evaluation only**	**External validation of one or more existing models in new data**	**Model evaluation**	
**Combination**	**Prediction model development combined in the same study (publication) with the evaluation of its apparent performance, internal validation performance, or external validation performance.**	**Model development and evaluation**	**X**

Step 3 - Assess quality and applicability or risk of bias and applicability

**Publication reference**	Oh SW, Byun SS, Kim JK, Jeong CW, Kwak C, Hwang EC, et al. Machine learning models for predicting the onset of chronic kidney disease after surgery in patients with renal cell carcinoma. BMC Med Inform Decis Mak. 2024;24(1):85.
**Models of interest**	Gradient Boosting compared with SVM, LR, DT, KNN, RF, AdaBoost, XGBoost and LightGBM on 12 perioperative variables
**Outcome of interest**	Post-operative CKD = eGFR <60 mL/min/1.73 m² (CKD-EPI) following partial or radical nephrectomy.

Model development

DOMAIN 1: Participants and data sources	
A. Quality	
From 9598 KORCC patients (214 variables), final cohort n=4389 (3313 no post-CKD, 1076 post-CKD), 7:3 train/test split, SMOTE applied on training set to balance to 1:1.	
	**Y/ PY/ PN/ N/ NI**
**1.1 Were appropriate data sources used?**	Y
**1.2 Was an appropriate study design used?**	Y
**1.3 Did the in- and exclusions of study participants result in a representative dataset?**	PY
**Concern regarding quality of selection of participants and data sources**	**QUALITYCONCERN:** ***low***/*high/unclear*
Rationale of quality rating: Multicentre Korean RCC database with detailed peri-operative information, appropriate retrospective cohort design, exclusions justified but without imputation.	
B. Applicability	
Multicentre Korean RCC cohort, 4% male in the post-CKD group vs 68.2% in the no-CKD group, mean pre-operative eGFR 94.7 (no-CKD) vs 77.7 (post-CKD), mean tumour size 38.6 cm vs 52.4 cm.	
**Concern that the (data of the) included participants do not match the review question or the assessor's intended use of the prediction model**	**APPLICABILITY CONCERN:** *low/**high**/unclear*
Rationale of applicability rating: Korean RCC nephrectomy population matches intended postoperative-management use, generalisability to non-Korean populations and non-tertiary settings not demonstrated.	

DOMAIN 2: Predictors	
A. Quality	
12 predictors, includes sex, smoking, diabetes, hypertension, ECOG score (0–4), haemoglobin, creatinine, albumin, calcium, preoperative eGFR, tumour size, tumour location, all available pre-/peri-operatively.	
	**Y/ PY/ PN/ N/ NI**
**2.1 Were predictors defined and assessed in a similar way for all participants?**	Y
**2.2 Was any pre-processing of predictors similar for all participants?**	Y
**2.3 Were predictor assessments made without knowledge of outcome data?**	Y
**2.4 Were the predictors included in the model available at the time the model was intended to be used?**	Y
**Concern regarding quality of selection of participants and data sources**	**QUALITYCONCERN:** ***low***/*high/unclear*
Predictors are routinely available pre-operatively, transparent literature-driven selection, standardised across 8 tertiary centres.	
B. Applicability	
**Concern that the definition, pre-processing, assessment, or timing of assessment of the predictors in the model do not match the review question or the assessor's intended use**	**APPLICABILITY CONCERN:** ***low***/*high/unclear*

Rationale of applicability rating: All 12 variables are routinely collected during RCC surgical work-up.

**DOMAIN 3: Outcome**	
**A. Quality**	
Post-operative CKD = eGFR <60 mL/min/1.73 m² calculated by the CKD-EPI equation from post-operative serum creatinine, patients with pre-existing CKD or low pre-operative eGFR excluded	
Or	**Y/ PY/ PN/ N/ NI**
**3.1 Were outcomes defined and assessed appropriately?**	Y
**3.2 Were outcomes defined and assessed in a similar way for all participants?**	Y
**3.3 Were outcome assessments made without use or knowledge of predictor data?**	Y
**3.4 Was the time interval between predictor assessment and outcome assessment appropriate?**	PY
**Concern regarding quality of the outcome or its determination**	**QUALITYCONCERN:** ***low****/high/unclear*
Standard CKD-EPI definition, same reference threshold across all participants, reliance on a single post-operative eGFR, transient post-surgical AKI may be misclassified as CKD.	
**B. Applicability**	
Outcome determined at a single post-operative time-point, simple binary endpoint, post-operative CKD = eGFR <60 mL/min/1.73 m² by CKD-EPI.	
**Concern that the outcome, its definition, assessment, or timing of assessment do not match the review question or the assessor's intended use**	**APPLICABILITY CONCERN:** ***low****/high/unclear*
eGFR <60 is clinically used threshold for post-nephrectomy CKD surveillance matching intended use.	

**DOMAIN 4: Analysis**	
**A. Quality**	
4389 patients (3313 no post-CKD / 1076 post-CKD) split 7/3, SMOTE on training to 2319:2319 (1:1).	
9 ML algorithms, SMOTE on training only, SHAP for feature importance, Kaplan-Meier survival analyses for the top features.	
Sensitivity, specificity, accuracy, AUROC and F1.	
Missing data: 972 patients with missing data excluded (complete-case), no imputation.	
	**Y/ PY/ PN/ N/ NI**
**4.1 Was there evidence that the sample size was reasonable?**	Y
**4.2 Were continuous and categorical predictors handled appropriately?**	Y
**4.3 Were participants with missing or censored data handled appropriately in the analysis?**	PN

**4.4 If methods to address class imbalance were used, was the model or the model predictions recalibrated?***	PN
**4.5 Were methods used to address potential model overfitting?**	Y
**Concern regarding quality of the analysis**	**QUALITYCONCERN:** *low/**high**/unclear*
Adequate sample, grid-search tuning, hold-out testing and SHAP interpretability, complete-case handling and use of SMOTE without subsequent recalibration of predicted probabilities.	

Model evaluation

Domain 1,2 and 3 same analysis as model development

**DOMAIN 4: Analysis**			
**Risk of bias**			
Test set n=1317 (994 no-CKD / 323 post-CKD), natural-prevalence (not SMOTE-corrected) evaluation set.			
Sensitivity, specificity, accuracy, AUROC and F1.			
Describe any participants who were excluded from the analysis: Exclusion same as development			
Describe missing data on predictors and outcomes as well as methods used for handling these missing data: Same as development			
	**Y/ PY/ PN/ N/ NI/ NA**		
**4.1 Was model evaluation based on only apparent performance avoided?**	Y		
	**A**	**I**	**E**
**4.2 Was there evidence that the sample size was reasonable?**	NA	Y	NA
**4.3 Were participants with missing or censored data handled appropriately in the analysis?**	NA	PN	NA
**4.4 If methods to address class imbalance were used, was the evaluation done in a dataset without imbalance correction?***	NA	Y	NA
**4.5 If data splitting was done to create training and test datasets, was there evidence that data leakage was avoided?***	**NA**	Y	**NA**
**4.6 If resampling methods were used to evaluate model performance, were all model development steps replicated in the resampling process?***	**NA**	Y	**NA**
**4.7 Was the predictive performance of the model evaluated appropriately, e.g., calibration, discrimination, and net benefit?**	NA	PN	NA
**Risk of bias introduced by the analysis**	**RISK OF BIAS:** *low/**high**/unclear*		
Hold-out testing at natural prevalence, only internal validation in single cohort, no calibration plot or decision-curve analysis, SHAP-derived probabilities not recalibrated after SMOTE.			

Step 4 - Assess the overall concerns regarding quality, risk of bias and applicability of the prediction model

**OVERALL JUDGEMENT OF PREDICTION MODEL**	
**Overall judgement of quality (development)**	**QUALITY CONCERN:** *low/**high**/unclear*

D4 high, complete-case exclusion of 972 patients and SMOTE without probability recalibration.	
**Overall judgement of risk of bias (evaluation)**	**RISK OF BIAS:**<br>*low/**high**/unclear*
Internal hold-out only, no calibration, no decision-curve analysis, missing-data exclusion biases evaluation, modest sensitivity in the natural-prevalence test set.	
**Overall judgement of applicability (development)**	**APPLICABILITY CONCERN:**<br>***low**/high/unclear*
Multicentre Korean tertiary-RCC nephrectomy cohort, generalisability to other ethnicities and non-tertiary settings unproven.	
**Overall judgement of applicability (evaluation)**	**APPLICABILITY CONCERN:**<br>***low**/high/unclear*
Same KORCC tertiary-Korean cohort as development.	

Zhu et al. (74)

Step 1 - PICOTS

**Population**	Adult inpatients with CKD hospitalised in the Nephrology Department of a single tertiary centre in a Chinese hospital.
**Index model(s)**	XGBoost classifier on 8 LASSO-selected variables.
**Comparator model(s)**	SVM, LR, naïve Bayes, KNN, RF and NN on same 8 features, SHAP for interpretation.
**Outcome(s)**	Composite cardiovascular event = coronary heart disease (CAD, MI, angina, coronary revascularisation), cerebrovascular disease (haemorrhagic or ischaemic stroke), death (cardiovascular, non-cardiovascular or unknown), congestive heart failure, or peripheral artery disease.
**Timing**	Retrospective
**Setting and intended use of the prediction model**	Chinese tertiary nephrology inpatient practice, intended as a clinical-decision-support tool to identify CKD patients at high risk of CVD events.

Step 2 – Classify the type of prediction model assessment

**Classify the assessment based on its aim**			
Type of prediction study	***Explanation***	***PROBAST+AI boxes to complete***	***Tick as appropriate***
**Development only**	**Prediction model development only, i.e., without evaluation of its performance.**	**Model development**	
**Evaluation only**	**External validation of one or more existing models in new data**	**Model evaluation**	

**Combination**	**Prediction model development combined in the same study (publication) with the evaluation of its apparent performance, internal validation performance, or external validation performance.**	**Model development and evaluation**	**X**

Step 3 - Assess quality and applicability or risk of bias and applicability

**Publication reference**	Zhu H, Qiao S, Zhao D, Wang K, Wang B, Niu Y, et al. Machine learning model for cardi-ovascular disease prediction in patients with chronic kidney disease. Front Endocrinol (Lausanne). 2024;15:1390729.
**Models of interest**	XGBoost, SVM, LR, naïve Bayes, KNN, RF and NN on same 8 features, SHAP for interpretation.
**Outcome of interest**	Composite CVD event (coronary heart disease, cerebrovascular disease, all-cause death, congestive heart failure, peripheral artery disease).

Model development

**DOMAIN 1: Participants and data sources**	
**A. Quality**	
8894 CKD inpatients, SMOTE-balanced to 13280 (6640 non-CVD / 6640 CVD), 8:2 split: train n=10624, test n=2656.	
	**Y/ PY/ PN/ N/ NI**
**1.1 Were appropriate data sources used?**	Y
**1.2 Was an appropriate study design used?**	PY
**1.3 Did the in- and exclusions of study participants result in a representative dataset?**	PN
**Concern regarding quality of selection of participants and data sources**	**QUALITYCONCERN:** *low/**high**/unclear*
Rationale of quality rating: Single-centre tertiary CKD inpatient sample, SMOTE augmentation inflates effective sample size and case:control balance compared with real prevalence, generalisability to outpatient or community CKD populations not demonstrated.	
**B. Applicability**	
Single-centre Chinese tertiary nephrology inpatient cohort, median age 52, 69.1% male, mean eGFR 44.6, hypertension 74.3%, intended use is risk-stratification of hospitalised CKD patients for targeted CVD prevention.	
**Concern that the (data of the) included participants do not match the review question or the assessor's intended use of the prediction model**	**APPLICABILITY CONCERN:** ***low**/high/unclear*
Rationale of applicability rating: Chinese tertiary nephrology inpatients align with intended clinical decision-support use in same setting, model has not been tested in outpatient, primary-care or non-Chinese CKD populations.	

DOMAIN 2: Predictors	
**A. Quality**	
LASSO 5-fold-CV selected 8 features: age, history of hypertension, sex, antiplatelet drug use, HDL, sodium, 24-h urinary protein and eGFR. All available at admission.	
	**Y/ PY/ PN/ N/ NI**
**2.1 Were predictors defined and assessed in a similar way for all participants?**	Y
**2.2 Was any pre-processing of predictors similar for all participants?**	Y
**2.3 Were predictor assessments made without knowledge of outcome data?**	Y
**2.4 Were the predictors included in the model available at the time the model was intended to be used?**	Y
**Concern regarding quality of selection of participants and data sources**	**QUALITYCONCERN:** ***low****/high/unclear*
Predictors are routinely measured at admission, standardised laboratory and clinical methods within a single centre, principled LASSO selection minimises overfitting.	
**B. Applicability**	
**Concern that the definition, pre-processing, assessment, or timing of assessment of the predictors in the model do not match the review question or the assessor's intended use**	**APPLICABILITY CONCERN:** ***low****/high/unclear*
Rationale of applicability rating: All 8 predictors are routinely available in nephrology inpatient practice, 24-h urinary protein less practical in outpatient or primary-care settings.	

DOMAIN 3: Outcome	
**A. Quality**	
Composite cardiovascular event ascertained by manual review of electronic medical records: CHD (coronary artery disease, MI, angina pectoris, coronary revascularisation), cerebrovascular disease (haemorrhagic or ischaemic stroke), all-cause death (CV / non-CV / unknown), congestive heart failure, peripheral artery disease (aortic aneurysm, aortic or other peripheral arterial revascularisation).	
Or	**Y/ PY/ PN/ N/ NI**
**3.1 Were outcomes defined and assessed appropriately?**	PY
**3.2 Were outcomes defined and assessed in a similar way for all participants?**	Y
**3.3 Were outcome assessments made without use or knowledge of predictor data?**	PY
**3.4 Was the time interval between predictor assessment and outcome assessment appropriate?**	PY
**Concern regarding quality of the outcome or its determination**	**QUALITYCONCERN:** ***low****/high/unclear*
Standard composite CVD outcome aggregating CHD, cerebrovascular, death, CHF and PAD, inclusion of non-CV and unknown-cause deaths within the composite, reliance on manual EMR review rather than adjudication blinded to predictors.	
**B. Applicability**	
Outcome determined retrospectively from electronic medical records over the study, variable follow-up per patient, composite endpoint with 2304/8894 events, individual frequencies of 5 contributing components not separately reported.	
**Concern that the outcome, its definition, assessment, or timing of assessment do not match the review question or the assessor's intended use**	**APPLICABILITY CONCERN:** ***low****/high/unclear*

Composite CVD endpoint is clinically meaningful for CKD risk-stratification, aggregation of non-CV death and absence of per-component frequencies dilute the cardiovascular interpretation.

DOMAIN 4: Analysis	
A. Quality	
8894 patients (6640 non-CVD / 2304 CVD) augmented by SMOTE to 13280 (6640/6640), 8/2 train/test split (10624 / 2656), 8 final predictors.	
LASSO regression with 5-fold CV for feature, 7 ML classifiers and compared, XGBoost selected on AUROC, SHAP used for interpretation.	
AUC, accuracy, sensitivity, specificity, F1 on the test set, confusion matrix; SHAP summary and dependence plots.	
Missing data: Variables with >25% missing excluded; remainder imputed by KNN, complete-case otherwise.	
	**Y/ PY/ PN/ N/ NI**
**4.1 Was there evidence that the sample size was reasonable?**	Y
**4.2 Were continuous and categorical predictors handled appropriately?**	Y
**4.3 Were participants with missing or censored data handled appropriately in the analysis?**	PY
**4.4 If methods to address class imbalance were used, was the model or the model predictions recalibrated?***	PN
**4.5 Were methods used to address potential model overfitting?**	Y
**Concern regarding quality of the analysis**	**QUALITYCONCERN:** *low/**high**/unclear*
Large balanced sample, LASSO selection, 7-model comparison, KNN imputation and SHAP interpretation, application of SMOTE before splitting, absence of probability recalibration.	

Model evaluation

Domain 1,2 and 3 same analysis as model development

DOMAIN 4: Analysis			
**Risk of bias**			
Test set 2656 (synthetic + real, class-balanced), confusion matrix in the test set.			
AUC, accuracy, sensitivity, specificity, F1 on the test set, confusion matrix; SHAP summary and dependence plots.			
Describe any participants who were excluded from the analysis: Exclusion same as development			
Describe missing data on predictors and outcomes as well as methods used for handling these missing data: Same as development			
	**Y/ PY/ PN/ N/ NI/ NA**		
**4.1 Was model evaluation based on only apparent performance avoided?**	Y		
	**A**	**I**	**E**

**4.2 Was there evidence that the sample size was reasonable?**	NA	Y	NA
**4.3 Were participants with missing or censored data handled appropriately in the analysis?**	NA	PY	NA
**4.4 If methods to address class imbalance were used, was the evaluation done in a dataset without imbalance correction?***	NA	PN	NA
**4.5 If data splitting was done to create training and test datasets, was there evidence that data leakage was avoided?***	**NA**	PN	**NA**
**4.6 If resampling methods were used to evaluate model performance, were all model development steps replicated in the resampling process?***	**NA**	PY	**NA**
**4.7 Was the predictive performance of the model evaluated appropriately, e.g., calibration, discrimination, and net benefit?**	NA	PN	NA
**Risk of bias introduced by the analysis**	**RISK OF BIAS:** *low/**high**/unclear*		
SMOTE applied before the train/test split, test set is no longer at natural, no calibration plot or decision-curve analysis, no external validation, explicit limitation acknowledged by authors.			

Step 4 - Assess the overall concerns regarding quality, risk of bias and applicability of the prediction model

**OVERALL JUDGEMENT OF PREDICTION MODEL**	
**Overall judgement of quality (development)**	**QUALITY CONCERN:** *low/**high**/unclear*
D1 and D4 high, LASSO selection, 7-model comparison, SHAP interpretation.	
**Overall judgement of risk of bias (evaluation)**	**RISK OF BIAS:** *low/**high**/unclear*
Internal hold-out only with SMOTE applied before splitting, no calibration, no decision-curve analysis, no external validation.	
**Overall judgement of applicability (development)**	**APPLICABILITY CONCERN:** ***low**/high/unclear*
Single Chinese tertiary nephrology inpatient cohort, matches intended-use setting but generalisability to other CKD populations unproven.	
**Overall judgement of applicability (evaluation)**	**APPLICABILITY CONCERN:** ***low**/high/unclear*
Same single-centre cohort.	

Tran et al. (75)

Step 1 - PICOTS

**Population**	Non-dialysis stage 4 or 5 CKD adults from French national multicentre prospective cohort.
**Index model(s)**	Naïve Bayesian network, 7 selected variables.
**Comparator model(s)**	LR, NN and RF built on the same 26 baseline variables, performance compared by 10-fold CV before final model selection.
**Outcome(s)**	2-year all-cause mortality

**Timing**	Baseline variables collected at study inclusion, prediction horizon 2 years.
**Setting and intended use of the prediction model**	French nephrology outpatient clinics, intended as a research-purpose individualised prognostic tool for stage 4–5 CKD patients.

Step 2 – Classify the type of prediction model assessment

**Classify the assessment based on its aim**			
Type of prediction study	***Explanation***	***PROBAST+AI boxes to complete***	***Tick as appropriate***
**Development only**	**Prediction model development only, i.e., without evaluation of its performance.**	**Model development**	
**Evaluation only**	**External validation of one or more existing models in new data**	**Model evaluation**	
**Combination**	**Prediction model development combined in the same study (publication) with the evaluation of its apparent performance, internal validation performance, or external validation performance.**	**Model development and evaluation**	**X**

Step 3 - Assess quality and applicability or risk of bias and applicability

**Publication reference**	Tran DNT, Ducher M, Fouque D, Fauvel JP. External validation of a 2-year all-cause mor-tality prediction tool developed using machine learning in patients with stage 4-5 chronic kidney disease. Journal of nephrology. 2024;37(8):2267–74.
**Models of interest**	Naïve Bayesian network (7 variables) selected after comparison with LR, NN and RF.
**Outcome of interest**	2-year all-cause mortality (binary).

Model development

**DOMAIN 1: Participants and data sources**	
**A. Quality**	
975 non-dialysis stage 4–5 CKD patients, 441 excluded, final analytic cohort n=534 (400 survivors, 134 deceased).	
	**Y/ PY/ PN/ N/ NI**
**1.1 Were appropriate data sources used?**	Y

**1.2 Was an appropriate study design used?**	Y
**1.3 Did the in- and exclusions of study participants result in a representative dataset?**	PY
**Concern regarding quality of selection of participants and data sources**	**QUALITYCONCERN:** ***low****/high/unclear*
Rationale of quality rating: Large prospective national multicentre cohort, clear inclusion of stage 4–5 CKD, exclusions justified.	
**B. Applicability**	
French nationwide prospective cohort, mean age 72.0, 55% male, mean eGFR 21.0 mL/min/1.73 m², 54.6% with CV history, intended use is individualised prognosis in stage 4–5 CKD outpatients in routine nephrology practice.	
**Concern that the (data of the) included participants do not match the review question or the assessor's intended use of the prediction model**	**APPLICABILITY CONCERN:** ***low****/high/unclear*
Rationale of applicability rating: Multicentre French stage 4–5 CKD outpatient cohort matches intended-use population, generalisability to non-French / non-European CKD populations not demonstrated.	

**DOMAIN 2: Predictors**	
**A. Quality**	
26 baseline candidate predictors collected at inclusion, final 7 predictors: age, ESA use, CV history, smoking status, 25-OH vitamin D, PTH, ferritin, routinely available at the prediction time-point.	
	**Y/ PY/ PN/ N/ NI**
**2.1 Were predictors defined and assessed in a similar way for all participants?**	Y
**2.2 Was any pre-processing of predictors similar for all participants?**	Y
**2.3 Were predictor assessments made without knowledge of outcome data?**	Y
**2.4 Were the predictors included in the model available at the time the model was intended to be used?**	Y
**Concern regarding quality of selection of participants and data sources**	**QUALITYCONCERN:** ***low****/high/unclear*
Standardised data collection across the 70 centre, baseline measurement before outcome, predictors routinely obtained in nephrology practice.	
**B. Applicability**	
**Concern that the definition, pre-processing, assessment, or timing of assessment of the predictors in the model do not match the review question or the assessor's intended use**	**APPLICABILITY CONCERN:** ***low****/high/unclear*
Rationale of applicability rating: All 7 predictors are routinely collected in stage 4–5 CKD follow-up.	

**DOMAIN 3: Outcome**	
**A. Quality**	
2-year all-cause mortality (binary), ascertained by the local nephrologist during scheduled follow-up, patients with <2-year follow-up, who changed centre, or who received transplant during follow-up were excluded.	
Or	**Y/ PY/ PN/ N/ NI**

**3.1 Were outcomes defined and assessed appropriately?**	Y
**3.2 Were outcomes defined and assessed in a similar way for all participants?**	Y
**3.3 Were outcome assessments made without use or knowledge of predictor data?**	PY
**3.4 Was the time interval between predictor assessment and outcome assessment appropriate?**	Y
**Concern regarding quality of the outcome or its determination**	**QUALITYCONCERN:** ***low***/*high*/*unclear*
Hard, unambiguous endpoint, uniform 2-year prediction horizon, censoring rules clearly defined.	
**B. Applicability**	
Outcome determined at a fixed 2-year horizon after baseline inclusion, simple (non-composite) binary endpoint, patients with <2-year follow-up, who changed centre or who received a transplant during follow-up were excluded rather than censored.	
**Concern that the outcome, its definition, assessment, or timing of assessment do not match the review question or the assessor's intended use**	**APPLICABILITY CONCERN:** ***low***/*high*/*unclear*
All-cause mortality at 2 years is clinically meaningful endpoint for intended individual prognosis use.	

**DOMAIN 4: Analysis**	
**A. Quality**	
534 patients / 134 deaths / 26 candidate predictors, 7 in final model.	
4 models built on the 26 variables and compared by 10-fold CV, Bayesian network chosen on performance + qualitative advantages, variable selection by ranked % of variance, 7 variables retained.	
AUROC, accuracy, sensitivity, specificity, PPV, NPV, 10-fold CV, no calibration plot, no decision-curve analysis.	
Missing data: 13% overall, imputed by each model's native missing-data handler	
	**Y/ PY/ PN/ N/ NI**
**4.1 Was there evidence that the sample size was reasonable?**	PY
**4.2 Were continuous and categorical predictors handled appropriately?**	Y
**4.3 Were participants with missing or censored data handled appropriately in the analysis?**	Y
**4.4 If methods to address class imbalance were used, was the model or the model predictions recalibrated?***	PY
**4.5 Were methods used to address potential model overfitting?**	Y
**Concern regarding quality of the analysis**	**QUALITYCONCERN:** ***low***/*high*/*unclear*
10-fold CV, principled variable selection, Bayesian imputation of missing data and synthetic-data augmentation, arbitrary fixed choice of 7 variables, absence of calibration and decision-curve analysis.	

Model evaluation

Domain 1,2 and 3 same analysis as model development

DOMAIN 4: Analysis			
**Risk of bias**			
534 patients / 134 deaths assessed by 10-fold CV, no held-out external sample.			
AUROC, accuracy, sensitivity, specificity, PPV, NPV, 10-fold CV, no calibration plot, no decision-curve analysis.			
Describe any participants who were excluded from the analysis: Exclusion same as development			
Describe missing data on predictors and outcomes as well as methods used for handling these missing data: Same as development			
	**Y/ PY/ PN/ N/ NI/ NA**		
**4.1 Was model evaluation based on only apparent performance avoided?**	Y		
	**A**	**I**	**E**
**4.2 Was there evidence that the sample size was reasonable?**	NA	PY	NA
**4.3 Were participants with missing or censored data handled appropriately in the analysis?**	NA	Y	NA
**4.4 If methods to address class imbalance were used, was the evaluation done in a dataset without imbalance correction?***	NA	PN	NA
**4.5 If data splitting was done to create training and test datasets, was there evidence that data leakage was avoided?***	**NA**	PN	**NA**
**4.6 If resampling methods were used to evaluate model performance, were all model development steps replicated in the resampling process?***	**NA**	PY	**NA**
**4.7 Was the predictive performance of the model evaluated appropriately, e.g., calibration, discrimination, and net benefit?**	NA	PN	NA
**Risk of bias introduced by the analysis**	**RISK OF BIAS:** *low/**high**/unclear*		
Internal validation only, synthetic-data augmentation (1:1 ratio), inflating apparent sensitivity/PPV, no calibration or decision-curve analysis, no external validation.			

Step 4 - Assess the overall concerns regarding quality, risk of bias and applicability of the prediction model

OVERALL JUDGEMENT OF PREDICTION MODEL	
**Overall judgement of quality (development)**	**QUALITY CONCERN:** ***low**/high/unclear*
10-fold CV, native missing-data handling and synthetic augmentation, no calibration / decision-curve analysis, arbitrary fixed 7-variable cut-off.	
**Overall judgement of risk of bias (evaluation)**	**RISK OF BIAS:** *low/**high**/unclear*
Internal cross-validation only on class-balanced synthetic dataset, no calibration, decision-curve analysis or external validation.	
**Overall judgement of applicability (development)**	**APPLICABILITY CONCERN:** ***low**/high/unclear*
French multicentre stage 4–5 CKD cohort, routinely available predictors match intended individualised-prognosis use.	

**Overall judgement of applicability (evaluation)**	**APPLICABILITY CONCERN:**<br>***low***/*high*/*unclear*
Same cohort as development.	
